# Symmetry of three-center, four-electron bonds†‡

**DOI:** 10.1039/d0sc02076a

**Published:** 2020-06-30

**Authors:** Ann Christin Reiersølmoen, Stefano Battaglia, Sigurd Øien-Ødegaard, Arvind Kumar Gupta, Anne Fiksdahl, Roland Lindh, Máté Erdélyi

**Affiliations:** Department of Chemistry – BMC, Uppsala University Husargatan 3 Uppsala 752 37 Sweden mate.erdelyi@kemi.uu.se; Department of Chemistry, Norwegian University of Science and Technology Høgskoleringen 5 Trondheim 7491 Norway; Centre for Material Sciences and Nanotechnology, University of Oslo Sem Sælands vei 26 0371 Oslo Norway; Department of Chemistry – Ångström Laboratory, Uppsala University Lägerhyddsvägen 1 751 20 Uppsala Sweden

## Abstract

Three-center, four-electron bonds provide unusually strong interactions; however, their nature remains ununderstood. Investigations of the strength, symmetry and the covalent *versus* electrostatic character of three-center hydrogen bonds have vastly contributed to the understanding of chemical bonding, whereas the assessments of the analogous three-center halogen, chalcogen, tetrel and metallic σ̂-type long bonding are still lagging behind. Herein, we disclose the X-ray crystallographic, NMR spectroscopic and computational investigation of three-center, four-electron [D–X–D]^+^ bonding for a variety of cations (X^+^ = H^+^, Li^+^, Na^+^, F^+^, Cl^+^, Br^+^, I^+^, Ag^+^ and Au^+^) using a benchmark bidentate model system. Formation of a three-center bond, [D–X–D]^+^ is accompanied by an at least 30% shortening of the D–X bonds. We introduce a numerical index that correlates symmetry to the ionic size and the electron affinity of the central cation, X^+^. Providing an improved understanding of the fundamental factors determining bond symmetry on a comprehensive level is expected to facilitate future developments and applications of secondary bonding and hypervalent chemistry.

## Abstract 2

The factors determining the symmetry and the fundamental nature of the three-center, four-electron bonds are assessed.

## Graphical abstract

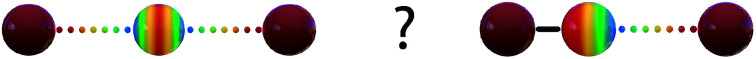

## Introduction

**Fig. 1:**
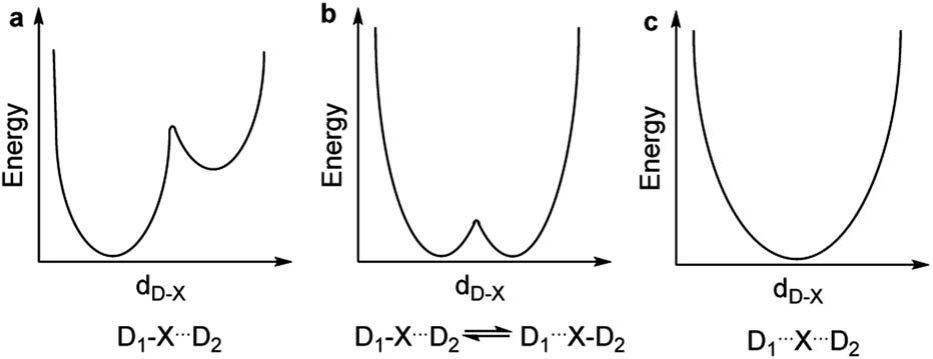
The energy potentials for the motion of an electrophilic atom, X, between two Lewis bases, D_1_ and D_2_, may follow (a) an asymmetric double-well, (b) an isoenergetic double-well, or (c) a single-well.

**Fig. 2:**
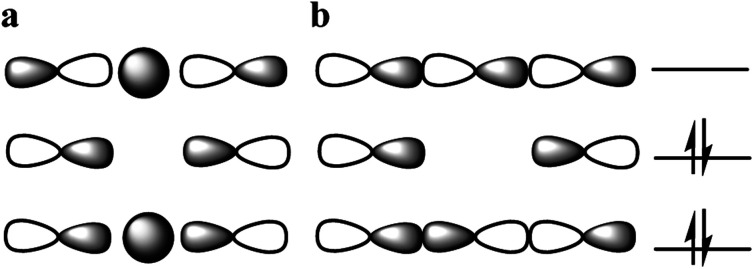
(a) Schematic of molecular orbitals for the three-center, four-electron bond [D⋯X⋯D]^+^, for hydrogen-, gold- and silver-centered systems, for example, and (b) for most main-group-element- and metal-centered systems, such as halogen-centered ones. In the former, the central orbital is of s-character, whereas in the latter it is of p-character. Two electrons are in the bonding, and two in the non-bonding orbitals. The electrons in the non-bonding orbitals are predominantly located on the terminal electron donor atoms, leaving the central atom electron deficient.

The nature of chemical bonding has fascinated scientists from as far back as the 12^th^ century.^1^ Accordingly, the hydrogen bond has remained one of the most studied topics of chemistry^2^ since it was first reported 150 years ago. There has been a firm interest in the exploration of weak chemical forces—reflected by the recent IUPAC projects to define hydrogen,^3^ halogen,^4^ chalcogen,^5^ pnictogen, and tetrel bonding.^6^ A common feature of these interactions is that they involve the donation of lone pair electrons into a suitable empty orbital (often σ*), which typically results in an electrophilic atom, X, being shared between two Lewis bases, D. These lone pair donors and the electrophile may either form a static asymmetric complex (Fig. 1a), a rapidly interconverting mixture of asymmetric complexes (Fig. 1b), or a static symmetric complex (Fig. 1c). The static asymmetric complex is most common for weak interactions involving electron donors with vastly different Lewis basicity. In this case, the central electrophile remains covalently bound to one donor atom and weakly bound to the other, thereby forming a complex with an asymmetric double-well energy potential (Fig. 1a). Alternatively, the electrophile may move from one donor to the other, producing a dynamic mixture of asymmetric isomers with the two bonds remaining different in character (Fig. 1b). An isoenergetic double-well describes the motion of the electrophile in these dynamic systems, which is typically named tautomerism, or prototropy for hydrogen bonding and halotropy for halogen bonding. As a third possibility, the two bonds to the electrophile may become equal in energy and distance, forming a static and symmetric complex (Fig. 1c), in which the motion of the electrophile between the electron donors is best described by a single-well energy potential. Notably, this symmetric arrangement results in an unusually strong noncovalent bond. Accordingly, “short, strong” (SSHB) or “low barrier” (LBHB) hydrogen bonds have been the topic of interest for more than half a century,^2,7–9^ and the symmetry of halogen^10^ and tetrel^11^ bonds has recently been assessed. The symmetric [F⋯H⋯F]^−^ bond was estimated at 240 kJ mol^−1^,^8^ whereas the dissociation energies of [I⋯I⋯I]^−^ and [N⋯C⋯N]^+^ bonds were found to be 180 kJ mol^−1^ and 50 kJ mol^−1^, respectively—significantly higher than typical noncovalent interactions. These strong noncovalent bonds have attracted interest for their possible role in stabilizing intermediates or even transition states.^3,5,8,11–13^ Their electronic structure has been interpreted in terms of the Rundle^14,15^ and Pimentel^16^ three-center, four-electron (3c4e) model,^10,17^ and as charge transfer, hypervalent or hypercoordinate bonds.^18^ For transition metals, this bonding situation has been described as 3c4e σ̂-type long bonding.^19^ Thus, the same bonding phenomenon has been discussed in context-dependent terms, with a common key aspect: an empty orbital of a formal cation, X^+^, simultaneously accepts electron pairs from two Lewis bases. Consequently, three atomic orbitals combine into three molecular orbitals that hold four electrons, and thus act as a three-center, four-electron system (Fig. 2).

**Fig. 3:**
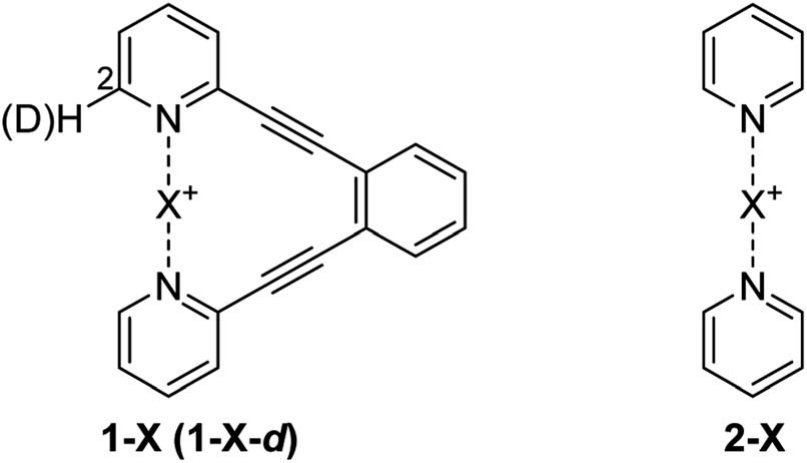
Bis(pyridine)-type systems used to study the nature and geometry of the three-center, four-electron [N–X–N]^+^ bonds of H^+^ (**1-H**), Li^+^ (**1-Li**), Na^+^ (**1-Na**), Br^+^ (**1-Br**), I^+^ (**1-I**), Ag^+^ (**1-Ag**) and Au^+^ (**1-Au**). As the [N–X–N]^+^ complexes of F^+^ and Cl^+^ are highly reactive and hence unstable, **1-Cl** and **1-F** were unsuitable to synthesize. The [N–Cl–N]^+^ and [N–F–N]^+^ bonds were therefore studied in the [bis(pyridine)fluorine(i)]^+^ (**2-F**) and [bis(pyridine)chlorine(i)]^+^ (**2-Cl**) complexes, at low temperature. A mixture of **1-X** and its mono-deuterated isotopologue **1-X-d** was used in isotopic perturbation of equilibrium (IPE) NMR experiments to differentiate between static [N⋯X⋯N]^+^ and dynamic [N⋯X–N]^+^ ⇄ [N–X⋯N]^+^ solution geometries.

The consequence of symmetry on the strength, length and reactivity of the three-center, four-electron bond has been the topic of debates in various research fields.^2,7–9,11–13,15–17,20–23^ However, the fundamental factors determining bond symmetry have not been comprehensively assessed. Herein, we evaluate the origin of symmetry in three-center, four-electron bonds with various central elements, and characterize their covalent *versus* electrostatic character using a benchmark system^10,24^ (Fig. 3).

## Results and discussion

Non-deuterated and deuterated **1-X** (**1-X-d**) and **2-X** complexes were synthesized following a literature protocol.^20^ Details of NMR spectroscopy, computation and of the X-ray data analyses are given in the ESI.‡

### The [N–H⋯N]^+^ bond

Single crystals were obtained for **1-H**, however, X-ray crystallographic analyses does not allow for reliable determination of the position of the N–H⋯N proton. Reported X-ray^24^ and neutron diffraction^24,26^ data for **2-H**, displays an asymmetric [N–H⋯N]^+^ geometry with comparable N–N distance (*d*_N–N_ = 2.737 Å) to that of **1-H** (2.851 Å, Table 1). Based on this observation, we presumed that similar to **2-H** (*d*_N–N_ = 2.737; *d*_N–H_ = 1.086 Å and 1.658 Å, neutron diffraction)^24,26^ also **1-H** possesses asymmetric [N–H⋯N]^+^ hydrogen bond in the solid state (Table 1). The asymmetry of **1-H** is thus intrinsic for the [N–H⋯N]^+^ hydrogen bond of bis(pyridine) complexes, and is not a consequence of the strain introduced by the diethynylbenzene backbone. The nitrogen–nitrogen distance of **1** is astonishingly shortened by ∼40%, from 4.685 Å (ref. 20 and 23) to 2.851 Å, upon formation of the [N–H⋯N]^+^ hydrogen bond. The nitrogen–hydrogen bond length, *d*_N–X(2)_ (1.919 Å, Table 1), is 30% shorter than the sum of the van der Waals radii^27^ of the involved atoms (1.20 Å + 1.55 Å = 2.75 Å), suggesting the formation of a strong bond. The energy of this bond was estimated (**1** + X^+^ → **1-X**^(+)^) to be −100.4 kJ mol^−1^ in gas phase and −51.19 kJ mol^−1^ in CH_2_Cl_2_ solution by DFT calculations. This three-center bond is capable of enforcing the strain necessary for distortion of the diethynylbenzene backbone to allow a nitrogen–nitrogen distance that is close to optimal for the [N–H⋯N]^+^ interaction, as reported for the unrestrained **2-H** complex (2.73 Å).^24,26,28–30^ The energy necessary for deformation of the diethynylbenzene backbone was estimated to be 41.5 kJ mol^−1^ (Table 2), by comparison of the energy of **1** when locked into its optimized geometry in **1-H**, with H^+^ removed, to the energy of its geometry as fully relaxed free ligand. The geometry of **1** is nonplanar due to the electrostatic repulsion of the nitrogen lone pairs, in contrast to the complexes **1-X**.

**Table 1:** Experimentally determined symmetry of the **1-X** complexes in solution and solid state, X-ray crystallographically determined N–X and N–N bond lengths, and ^15^N NMR chemical shifts. Computed quantities are shown in italic

Complex	Symmetry^a^	*d*_N–X covalent_ f [Å]	*d*_N–X_(1) [Å]	*d*_N–X_(2) [Å]	*d*_N–N_ [Å]	*δ* ^15^N_complex_ [ppm]	*δ* ^15^N_ligand_ [ppm]	Δ*δ*^15^N_coord_ [ppm]
**1-H**	A/A	1.020	0.943^b^	1.919^b^	2.851	−137.9	−64.5	−73.4
	*A*		*1.050*	*1.856*	*2.881*	*−146.3*	*−70.5*	*−75.8*
**1-Li**	S/n.a.	2.090	n.a^c^	n.a^c^	n.a^c^	−91.3	−64.5	−26.8
	*S*		*2.077*	*2.077*	*4.101*	*−110.4*	*−70.5*	*−39.9*
**1-Na**	S/S	2.290	2.447	2.434	4.831	−83.2	−64.5	−18.7
	*S*		*2.452* d	*2.452* d	*4.750* d	*−99.5* d	*−70.5* d	*−28.9* d
**1-F**	n.a^e^	1.460	n.a^e^	n.a^e^	n.a^e^	n.a^d^	−64.5	n.a^d^
	*A*		*1.357*	*2.916*	*4.272*	*−129.2/−70.0*	*−70.5*	*−58.7/+0.5*
**1-Cl**	n.a^e^	1.730	n.a^e^	n.a^e^	n.a^e^	n.a^e^	−64.5	n.a^e^
	*S*		*2.011*	*2.011*	*4.021*	*−119.3*	*−70.5*	*−48.8*
**1-Br**	S/n.a^g^	1.910	n.a^g^	n.a^g^	n.a^g^	−141.2	−64.5	−76.7
	*S*		*2.128* h	*2.128* h	*4.257* h	*−129.9*	*−70.5*	*−59.4*
**1-I**	S/S	2.100	2.174	2.177	4.352	−165.5	−64.5	−101.0
	*S*		*2.300*	*2.300*	*4.597*	*−139.8*	*−70.5*	*−69.3*
**1-Ag**	S/S	2.280	2.101	2.119	4.219	−116.0	−64.5	−51.5
	*S*		*2.144*	*2.144*	*4.288*	*−142.9*	*−70.5*	*−72.4*
**1-Au**	S/S	2.190	2.019	2.009	4.026	−150.1	−64.5	−85.6
	*S*		*2.050*	*2.050*	*4.100*	*−161.4*	*−70.5*	*−90.9*

a The symmetry in solution and solid state for the different complexes are indicated by A or S for the asymmetric or symmetric states, respectively. The first letter in each row indicates the symmetry in solution, whereas the second that in the solid state.

b The covalent N–H distance from XRD is constrained, and not freely refined.

c Data not available due to the low affinity of Li^+^ to **1** preventing crystallization despite a large number of attempts.

d Computed data is given for the 1 : 1 complex of **1** and Na^+^.

e Data not available due to instability of **1-F**, **1-Cl**.^22^

f These values are the sum of tabulated covalent radii.^27^ They agree with the covalent bond lengths calculated for the N–X bond of pyridine-X systems.

g Suitable crystals for X-ray analysis were not available for the **1-Br** complex.

h Calculated distances *d*_N–X_ and *d*_N–N_ for **2-Br** complex are shorter by 0.002 Å and 0.006 Å, respectively, than those of **1-Br**.

**Table 2:** Interaction energy (Δ*G*), deformation energy (Δ*E*_def_), and natural population analysis (NPA) charges for the atoms involved in the 3c4e bond (N_1_, N_2_ and X) in the equilibrium geometries of complexes **1-X**

X	Δ*G*^a^ [kJ mol^−1^]	Δ*E*_def_^b^ [kJ mol^−1^]	N_1_	N_2_	X
H^+^	−116.0	41.5	−0.44	−0.48	+0.47
Li^+^	+2.8	2.4	−0.52	−0.52	+0.90
Na^+^	+4.8	0.9	−0.50	−0.50	+0.95
F^+^	−314.4	26.1	−0.14	−0.44	+0.13
Cl^+^	−202.0	12.1	−0.36	−0.36	+0.17
Br^+^	−204.0	9.0	−0.42	−0.42	+0.29
I^+^	−174.6	7.0	−0.47	−0.47	+0.43
Ag^+^	−115.7	2.9	−0.51	−0.51	+0.73
Au^+^	−267.7	7.2	−0.48	−0.48	+0.52

a Defined as the energy change for **1** + XBF_4_ → **1-X** + BF_4_.

b The energy difference of the geometry **1** would possess if X was coordinated (**1-X**) and as free ligand. For **1-Na**, data is given for the 1 : 1 complex of **1** and Na^+^.

It should be emphasized that the asymmetry proposed here for **1-H** in the solid state is not based only on its X-ray data, but on the neutron diffraction data of the structurally closely related **2-H**,^26^ which shows comparable N–N distance. Whereas some of the X-ray data of **1-H** shows asymmetric electron density distribution (CCDC 1989941‡), that obtained for some other crystals (CCDC 19888175, Fig. S1 and S2, ESI‡) could easily be misinterpreted as a symmetric geometry, due to reflections of a twin domain that are overlapping with those of the main domain, yielding a structural shadow. Analogous challenges might be accountable for some of the past half century's intense debates on the symmetry of “short, strong hydrogen bonds”.^8,9^ Some of the literature data proposing a symmetric [N⋯H⋯N]^+^ geometry for **2-H**,^31^ for example, is not convincing, as the position of the proton could not be experimentally located, due to large noise or otherwise poor raw data indicated by the high *R* factor.^32^

In solution, the formation of the three-center hydrogen bond was indicated by large ^15^N coordination shifts, Δ*δ*^15^N_coord_ −73.4 ppm of **1-H** in CD_2_Cl_2_ solution (Table 1). The single set of ^15^N NMR signals of this complex is compatible either with a static, symmetric [N⋯H⋯N]^+^, or a rapidly equilibrating mixture of tautomers [N⋯X–N]^+^ ⇄ [N–X⋯N]^+^ possessing a low energy barrier for interconversion.

The isotopic perturbation of equilibrium (IPE) method,^33,34^ previously proven to be capable of distinguishing between static and rapidly equilibrating dynamic systems,^2,9,20,21,23,33,34^ was exploited to determine the nature of **1-H**. In short, IPE relies on vibrational energy changes upon selective isotope labeling, usually a hydrogen-to-deuterium substitution close to the molecular site of interest. The isotopologue mixture, here **1-H** and **1-H-d**, is analyzed by ^13^C NMR yielding two sets of signals, one originating from the selectively-deuterated molecule, and the second from the corresponding non-deuterated one. The chemical shift difference between these signals is called the isotope shift, ^*n*^*Δ*_obs_, where *n* denotes the number of bonds between the site of the ^1^H-to-^2^H substitution and the carbon of interest. The observed shift difference consists of the intrinsic isotope shift, ^*n*^*Δ*_0_, and the equilibrium isotope shift, ^*n*^*Δ*_eq_, as described by

1 ^*n*^*Δ*_obs_ = *δ*_C(D)_ − *δ*_C(H)_ = ^*n*^*Δ*_0_ + ^*n*^*Δ*_eq_.

Here *δ*_C(H)_ and *δ*_C(D)_ are the ^13^C NMR chemical shift of a carbon atom when it is bound to a hydrogen or to a deuterium, respectively. The intrinsic isotope effect, ^*n*^*Δ*_0_, is present in all molecular systems independent of their static or dynamic nature and is usually small and attenuates as *n* increases. In contrast, the equilibrium isotope shift, ^*n*^*Δ*_eq_, is only present in dynamic systems. The magnitude of the equilibrium isotope shift, ^*n*^*Δ*_eq_, depends on the equilibrium constant *K* of the dynamic process, and hence it is temperature dependent according to the van't Hoff equation.^35^ The magnitude of the observed isotope shift, ^*n*^*Δ*_obs_, does not always allow direct differentiation between static and dynamic systems, however, its temperature dependence has proven to be diagnostic.^20^ Herein, we studied the symmetry of the 3c4e [N–X–N]^+^ bonds using IPE NMR with ^13^C {^1^H, ^2^H} detection, using mixtures of the isotopologues **1-X** and **1-X-d** in CD_2_Cl_2_ solutions. Following the literature, the temperature dependence of the isotope shifts of the free ligand, 1,2-bis(2′-pyridylethynyl)-benzene (**1**), was used as reference for a static structure (^*n*^*Δ*_obs_ = ^*n*^*Δ*_0_). The large temperature dependence of the isotope shifts of the isotopologue mixture **1-H**/**1-H-d** (Σ|*Δ*_obs_| = 38.0, Table 3), as compared to the static reference (Σ|*Δ*_obs_| = 26.6) revealed **1-H** to be a dynamic mixture of rapidly interconverting tautomers (Fig. 1b).

**Table 3:** Temperature coefficients (ppm *x* K) of the ^13^C isotope shifts of **1-X** complexes and **1**, observed in CD_2_Cl_2_ solutions

Structure	C2 ^1^*Δ*_obs_	C3 ^2^*Δ*_obs_	C4 ^3^*Δ*_obs_	C5 ^4^*Δ*_obs_	C6 ^5^*Δ*_obs_	Σ|*Δ*_obs_|
**1**	−8.1	−9.1	−1.5	+3.4	−4.5	26.6^a^
**1-H**	−10.0	−11.0	−3.0	0	15	38.0^a^
**1-Li**	−8.0	−8.6	+1.0	+1.5	−3.6	22.7
**1-Na**	−9.3	−7.3	+1.7	−1.9	−3.1	23.3
**1-Br**	−7.0	−9.0	−3.0	0	—	19.0^a^
**1-I**	−8.9	−10.8	+0.7	0	−2.0	22.4^a^
**1-Ag**	−7.7	−5.8	+7.2	−0.9	−4.4	26.0
**1-Au**	−6.7	−10.3	+0.3	—	−3.9	21.2

a IPE data for **1**, **1-H**, **1-I** and **1-Br** are literature known.^20,23^

The DFT estimated low Gibbs free energy barrier of the symmetric transition state, 11.3 kJ mol^−1^ at 298.15 K, is in excellent agreement with the IPE indicated tautomerization in solution, confirming that **1-H** exists as a mixture of asymmetric tautomers in solution. The computed Δ*δ*^15^N_coord_, and the N–H and N–N distances are also in agreement with those obtained experimentally (Table 1). The interaction energy of **1-H**, defined as **1** + HBF_4_ → **1-H**^(+)^ + BF_4_^−^, amounts to −116.0 kJ mol^−1^ (Table 2), accounting for the sum of the energy released upon formation of the covalent N–H bond, the energy gained due to the noncovalent bond, minus the deformation energy.

### The [N⋯Li⋯N]^+^ bond

Despite repeated attempts, no **1-Li** crystals suitable for X-ray analysis were obtained. The Δ*δ*^15^N_coord_ −26.8 ppm of **1-Li** in CD_2_Cl_2_ solution (Table 1) suggests the formation of a very weak complex. This is comparable to the Δ*δ*^15^N_coord_ −26.3 ppm of pyridine in a 2 : 1 complex with Li^+^. In the investigation of these complexes, lithium tetrakis(pentafluorophenyl)borate ethyl etherate was used instead of the tetrafluoroborate salt, due to its better solubility in dichloromethane. The low interaction affinity of Li^+^ to **1** is confirmed by the DFT calculated negligible interaction energy (Table 2). DFT analysis of the minimum energy geometry indicated **1-Li** to be symmetric (Table 1), with significantly less N–N distance shortening than **1-H**, *i.e.* 13% rather than 40%, as compared to **1** (4.685 Å). The computed nitrogen–lithium distance, 2.077 Å, is 38% shorter than the sum of the van der Waals radii of the involved atoms (3.37 Å). Natural population analysis (Table 2) showed only a minor electron transfer from the nitrogen lone pairs to the Li^+^ 2s and 2p_*z*_ orbitals, revealing that the observed weak interaction is dominantly electrostatic. The observed low temperature dependence of the isotope shifts of **1-Li** (Table 3) is in agreement with the DFT-predicted static and symmetric [N⋯Li⋯N]^+^ geometry in solution (Fig. 1c).

### The [N⋯Na⋯N]^+^ bond

**Fig. 4:**
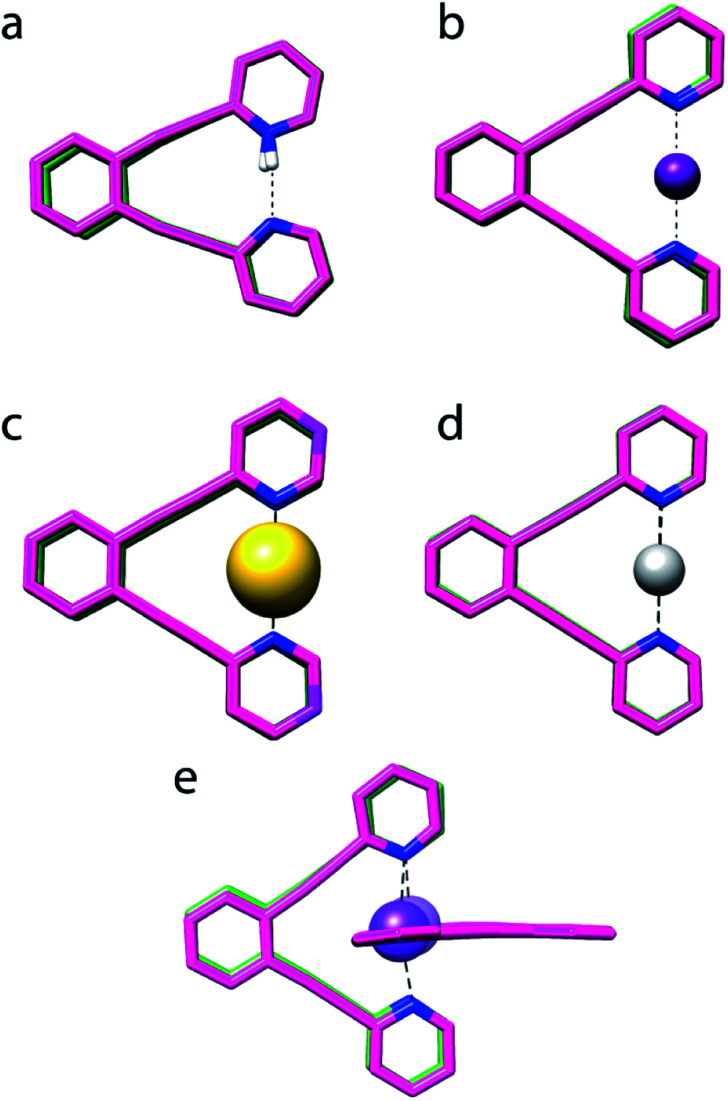
The overlaid X-ray determined (pink) and DFT optimized (green) geometries of (a) **1-H**, (b) **1-I**, (c) **1-Au**, (d) **1-Ag** and (e) **1-Na** (this is a 2 : 1 complex of **1** and Na^+^). Counter-ions and C–H protons are not shown. No X-ray data was obtained for **1-Li**, **1-F**, **1-Cl** and **1-Br**, and their computed structures are given in the ESI.‡

Single crystal X-ray analysis of **1-Na** indicated formation of a 2 : 1 complex of **1** and Na^+^, shown in Fig. 4e. Its N–Na^+^ distances (Table 1) differ by <1%, indicating a symmetric [N⋯Na⋯N]^+^ solid state geometry. To accommodate the Na^+^, the nitrogen–nitrogen distance of **1** is increased by 3%. The observed N–N and N–Na^+^ distances are in agreement with those computed by DFT (Table 1). The nitrogen–sodium distance, 2.434 Å, is 36% shorter than the sum of the van der Waals radii of the involved atoms (3.83 Å). Natural population analysis (Table 2) reveals that similar to **1-Li**, the electron transfer from the nitrogens to the Na^+^ 3s and 3p_*z*_ orbitals is insignificant. Thus, the N–Na^+^ interaction is primarily electrostatic. The weak and symmetric nature of the [N⋯Na⋯N]^+^ interaction is supported by the small Δ*δ*^15^N_coord_ and small temperature coefficients of the isotope effects of **1-Na** in solution (Tables 2 and 3), and by the negligible interaction energy of Na^+^ to **1** (Table 2). The similarity of the nature of the [N⋯Na/Li⋯N]^+^ interactions of **1-Na** and of **1-Li**, along with the X-ray structure obtained for **1-Na** (Fig. 4e) raises the question whether the interaction of alkali metals with **1** ought to be seen as purely electrostatic, not involving formation of a 3c4e bond that would result in the molecular orbital system shown in Fig. 2b.

### The [N–F⋯N]^+^ bond

The [N–F⋯N]^+^ bond has been previously studied using the [bis(pyridine)fluorine(i)] (**2-F**) model system^22,30,36^ and was reported to be highly reactive and thus only exist at low temperature, preventing its IPE NMR investigation. Due to the high reactivity of **1-F**, the NMR and X-ray analysis was unfeasible and therefore it was investigated with DFT computations only. DFT indicates that **1-F** prefers a static asymmetric [N–F⋯N]^+^ geometry (Fig. 1a) with a 95.15 kJ mol^−1^ (at the experimental temperature of −35 °C) energy barrier to the symmetric transition state, preventing halotropic interconversion analogous to the prototropy of **1-H**. The predicted Δ*δ*^15^N_coord_ shifts of **1-F** (Table 1) are in agreement with those reported for **2-F** at −35 °C for CD_3_CN solution (−55.2 ppm and −1.8 ppm, for the two nitrogens, respectively).^22^ Overall, the [N–F⋯N]^+^ bond of **1-F** is static asymmetric, corresponding to the potential energy curve shown in Fig. 1a. The noncovalent nitrogen–fluorine bond length is 3.4% shorter than the sum of the van der Waals radii of the involved atoms (3.02 Å). As expected for the weak halogen bond donor fluorine, the estimated halogen bond energy of **1-F** in the gas phase and in CH_2_Cl_2_ solution are merely −27.9 kJ mol^−1^ and −4.8 kJ mol^−1^, respectively, such that the large interaction energy, −314.4 kJ mol^−1^, reported in Table 2, is essentially due to the formation of the covalent N–F bond. This and the DFT-predicted small Δ*δ*^15^N_coord_ are in agreement with the expected low interaction energy of a fluorine-centered halogen bond in solution.^37^ The experimentally observed asymmetry of the unrestrained **2-F**^22^ suggests that the asymmetry of **1-F** is not induced by the strain due to the diethynylbenzene backbone.

### The [N⋯Cl⋯N]^+^ bond

Similar to **2-F**, **2-Cl** has been reported to be highly reactive and only exist below −80 °C in solution, preventing the detailed NMR spectroscopic and X-ray crystallographic investigation of the [N⋯Cl⋯N]^+^ bond geometry.^22^ Accordingly, spectroscopic investigation of **1-Cl** was seen as unfeasible. DFT predicts **1-Cl** to be static and symmetric (Fig. 1c), with a nitrogen–chlorine bond length 39% shorter than the sum of the van der Waals radii of the involved atoms (3.30 Å). The nitrogen–nitrogen distance of **1** is shortened by 14% in **1-Cl**. Natural population analysis assigns +0.17 charge to the chlorine, indicating substantial electron transfer from the nitrogen lone pairs. This is confirmed by the second order perturbation theory analysis of the Fock matrix (FPT2)^38^ that reveals the covalent character of this bond (Table 4), which has −202.0 kJ mol^−1^ estimated energy (Table 2). The predicted Δ*δ*^15^N_coord_ of **1-Cl** is small as compared to those of **1-Br** and of **1-I** (Table 1). The above suggest the [N⋯Cl⋯N]^+^ bond of **1-Cl** to be weak, static and symmetric, and to possess comparable N⋯Cl bond length (2.011 Å) and properties to that reported for **2-Cl** (*d*_N–Cl_ 2.025 Å).^22^

**Table 4:** FPT2 elements^38^ between the nitrogen lone pair (*n*_N_) and the central partially occupied *n*_s_ or *n*_p_ (*n*_(s,p)_) atomic orbital and their occupation number. Values 3c_b_ and 3c_nb_ correspond to occupation numbers of the second NBO set identified

X	*n*_N_ → *n*_(s,p)_^a^	*n*_N_ b	*n*_(s,p)_ b	3c_b_^b^	3c_nb_^b^
Li^+^	56.9^c^	1.91	0.08^c^	—d	—d
Na^+^^e^	21.3^c^	1.91	0.04^c^	—d	—d
Cl^+^	809.2	1.55	0.87	1.98	1.90
Br^+^	616.7	1.60	0.76	1.98	1.91
I^+^	454.0	1.66	0.63	1.97	1.92
Ag^+^	256.9	1.81	0.29	1.95	1.92
Au^+^	612.1	1.70	0.53	1.97	1.94

a This value is given in kJ mol^−1^, and corresponds to the contribution of a single nitrogen lone pair. There is another, exactly equal contribution from the second lone pair.

b NBO occupation numbers provide an estimate of the occupation of an orbital. They vary between 0 (completely empty orbital) and 2 (exactly doubly occupied orbital). Here, *n*_N_ and *n*_(s,p)_ belong to the NBO set consisting of isolated lone pairs and partially occupied central atomic orbitals, and refer to the nitrogen lone pair orbital and to the central s or p atomic orbital, respectively. The 3c_b_ and 3c_nb_ belong to the second NBO set describing the system as a 3c4e bond, and refer to the bonding and non-bonding orbitals, respectively.

c Combined value for both the 2s and 2p orbitals of lithium, and for the 3s and 3p orbitals of sodium.

d No NBO set with 3c4e bond orbitals was found.

e Computed data is given for the 1 : 1 complex of **1** and Na^+^.

### The [N⋯Br⋯N]^+^ bond

The geometry of the **1-Br** complex has been demonstrated to be static and symmetric in solution.^20^ As no suitable crystals for **1-Br** were available for us, the DFT predicted bond distances (Table 1) are here compared to those reported for the single crystals of the analogous unrestrained **2-Br** complex (*d*_N–Br_ 2.075 Å and 2.101 Å, *d*_N–N_ 4.182 Å).^25^ The latter 1.2% difference in the *d*_N–Br_ of **2-Br** is most likely due to crystal packing forces along with the pyridines not being covalently locked, and thus the complex possesses an overall symmetric [N⋯Br⋯N]^+^ three-center bond in the solid state. In line with the expectations, **1-Br** was computed to possess slightly longer *d*_N–Br_ (2.128 Å, Table 3) than **2-Br**, which is well-explained by the steric strain introduced by the diethynylbenzene backbone. Its nitrogen–bromine bond distance is 37% shorter than the sum of the van der Waals radii of the involved atoms (3.40 Å), comparable to that of **1-Cl**. The nitrogen–nitrogen distance of **1-Br** is shortened by 9% as compared to **1**, necessitating a total deformation energy of 9 kJ mol^−1^ (Table 2). The [N⋯Br⋯N]^+^ interaction energy is estimated to be −204.0 kJ mol^−1^, comparable to that of **1-Cl**. A larger, +0.29, positive charge is assigned to bromine by the NPA (Table 2), consequently increasing the electrostatic character and decreasing the covalent one of the bromine(i)-centered 3c4e bond as compared to the chlorine(i)-centered one. This is also reflected by the weaker interaction predicted by the FPT2 analysis^38^ (Table 4). The temperature dependence of the isotope shifts of **1-Br** (Table 3) are compatible with a static and symmetric solution geometry. Overall, **1-Br** has a weak, static and symmetric 3c4e bond in both the solid state and in solution.

### The [N–I–N]^+^ bond

The [N–I–N]^+^ bond of **1-I** has been studied in solution,^20^ however, its single crystal X-ray analysis is reported here for the first time (Fig. 4b). The analysis of the X-ray data of this complex has been highly challenging due to rotational disorder, upon various packing of the three-aromatic rings of **1** within the crystal (for details see the ESI‡). The nitrogen–iodine distance of **1-I** is 38% shorter than the sum of the van der Waals radii of the involved atoms (3.53 Å), and hence comparable to those computed for **1-Cl** and **1-Br**. The nitrogen–nitrogen distance of **1-I** is shortened by 7% as compared to **1**. IPE studies of **1-I** (Table 3) demonstrated it to be static and symmetric in solution,^20^ which was corroborated by DFT simulations (Table 1). The observed large Δ*δ*^15^N_coord_, that is consistent with previous findings,^13,20,21,23^ could be reasonably well reproduced by DFT calculations, the predicted values fitting the experimental trends. The discrepancy of the predicted and observed Δ*δ*^15^N_coord_ for **1-I** can be understood in terms of the longer *d*_N–X_ distances obtained with DFT decreasing the coordination strength of the iodonium ion, whereas, *e.g.* for **1-Br**, the correlation between the equilibrium geometry and the X-ray structure yields a consistency between the observed and computed coordination shifts. Natural population analysis predicts iodine to retain the largest positive charge among the three symmetric halogens, +0.43, which together with the FPT2 analysis (Table 4) suggest that the 3c4e bond of **1-I** has the most electrostatic and the least covalent character. This is corroborated by the lowest interaction energy, −174.6 kJ mol^−1^, among the three larger halogens.

It should be emphasized here that interaction energy (Table 2) does not necessarily parallels stability. Hence, the interaction energy is here defined as the energy change associated to the **1** + XBF_4_ → **1-X** + BF_4_ hypothetical process, which does not reflect the typical experimental decomposition route of **1-I**, **1-Br** and **1-Cl** in solution. Their decomposition is commonly moisture mediated, and may be described by the **1-X** + H_2_O → **1-H** + HOX reaction, for which the order of energy changes (54.9 kJ mol^−1^ (**1-I**), 48.9 kJ mol^−1^ (**1-Br**) and 35.0 kJ mol^−1^ (**1-Cl**)), follow the experimentally detected stability order **2-I** > **2-Br** > **2-Cl** (ESI‡).^20,22^

### The [N⋯Ag⋯N]^+^ bond

X-ray analysis of **1-Ag** revealed a symmetric geometry (Fig. 4d) with <1% asymmetry of the nitrogen–silver distances, which is most probably explained by crystal packing forces. The observed nitrogen–silver bond lengths are 35% shorter than the sum of the van der Waals radii of the involved atoms (3.27 Å), whereas its nitrogen–nitrogen distance is shortened by 10% as compared to **1**. Its Δ*δ*^15^N_coord_ of −51.5 ppm indicates the formation of a coordinative complex in solution, which is in agreement with the literature,^39^ and is reasonably reproduced by DFT. The observed N–Ag bond lengths are comparable to those predicted. In contrast to the halogen's 3c4e bond, that of **1-Ag** is formed by the overlap of the silver 5s orbital with the nitrogen 2p lone pairs (Fig. 2a). The natural bond orbital analysis supports this interpretation (see ESI for more details‡). This bond is similar to the molecular orbitals of **1-H**, and possesses similar strength, −115.7 kJ mol^−1^ (Table 2). IPE NMR investigation of **1-Ag**/**1-Ag-d** indicated static and symmetric [N⋯Ag⋯N]^+^ bond geometry in solution. Overall, the 3c4e bond of **1-Ag** is demonstrated to be static and symmetric in the solid state and in solution.

### The [N⋯Au⋯N]^+^ bond

X-ray analysis of the single crystals obtained for **1-Au** indicated a static and symmetric 3c4e bond geometry (Fig. 4c). The nitrogen–gold bond lengths were 37% shorter than the sum of the van der Waals radii of the involved atoms (3.21 Å), whereas the nitrogen–nitrogen distance shortened by 14% as compared to **1**. DFT corroborated the symmetric geometry found in the solid state, and estimated the [N⋯Au⋯N]^+^ bond to possess a comparable interaction energy, −267.7 kJ mol^−1^, to **1-I**, and higher than that of the corresponding bond of **1-Ag** (Table 2). It also has a higher covalent character than the 3c4e bond of **1-Ag** (Table 4). Natural population analysis confirmed the similarity of the three-center bonds of **1-Au** and **1-I**, with the only difference that the electron donation from the nitrogen lone pairs is accepted by the empty 6s atomic orbital of Au(i), instead of a 5p orbital as in **1-I** (*vide infra*). The predicted Δ*δ*^15^N_coord_ −90.9 ppm is in agreement with that observed for **1-Au** in CD_2_Cl_2_ solution (Table 1), confirming the formation of a strong complex in solution. The low temperature dependence observed for the isotope shifts of **1-Au** (Table 3) was in line with the DFT-predicted static and symmetric geometry in solution (Fig. 1c).

Overall, agreement between the crystallographic data and the DFT predicted geometries was obtained for the complexes (Fig. 4), even though the latter tends to overestimate the N–X^+^ and N–N distances by 0.03 Å to 0.1 Å. Importantly, the coordinating ion enforces a significant shortening of the nitrogen–nitrogen distance of **1** for all **1-X** complexes but **1-Na** (deformation energies are reported in Table 2), with **1-H** being the most extreme (40%). The larger extent of bond shortening correlates with the estimated interaction energy and thus reflects the formation of a strong three-center bond, independently of the nature of the central ion. The N–X bond shortening of the studied complexes with respect to the sum of the van der Waals radii of the involved atoms follows the order of **1-Cl** (39%) ∼ **1-I** (38%) ∼ **1-Li** (38%) ∼ **1-Br** (37%) ∼ **1-Au** (37%) ∼ **1-Na** (36%) ∼ **1-Ag** (35%) > **1-H** (30%) ≫ **1-F** (3%),^40^ and thus indicates that the formation of static, symmetric bonds results in a ∼35–39% bond shortening, that of a “resonance-stabilized” bond in *ca.* 30% shortening, whereas a weak, conventional secondary bond in <10% change in the bond length. It should be underlined that the 3c4e bonds of the halogens and transition metals possess a significant covalent character, whereas the bonds of **1-Li** and **1-Na** are dominantly electrostatic. Hence, vast reduction in the interatomic distances in comparison with the sum of the van der Waals radii of the involved atoms does not indicate formation of a 3c4e bond, but it differentiates asymmetric and symmetric systems.

The complexes of larger cations with **1** are static and symmetric both in solution and in the solid state. An important similarity between the **1-H** and **1-Li**, and the **1-F** and **1-Cl** pairs is that in both, the complex possessing the lighter ion is asymmetric (Fig. 1a and b), while the one with the heavier central ion is symmetric (Fig. 1c). As the analogous unrestrained bis(pyridine)-type **2-X** complexes show the same symmetry,^21,22,25,41^ this conclusion appears general, *i.e.* scaffold independent. For rationalization of the competition between the possible symmetric and asymmetric bond geometries, we introduce a numerical index derived from the size and the electron affinity of the central cation. The latter parameter reflects the ability of X^+^ to accept electron density. It is quantified by the electron affinity of the ion (EA(X^+^)), which is equivalent to the negative ionization energy of the atom X. The higher EA(X^+^) is, the more X^+^ is likely to form a covalent bond with the nitrogen, due to the favorable energetic outcome. The symmetry behavior of three-center bonds can be rationalized by the variation of these two independent quantities, as a function of the central species of the three-center bond:

2 *f*_sym_ = EA(X^+^)/4π*R*^2^.

This symmetry function, *f*_sym_, defines the electron affinity per surface area of X^+^, which we propose to be the determining factor for the symmetry properties of the 3c4e [N–X–N]^+^ bonds studied here. Here, the radii were taken from ref. 27, whereas the electron affinity of the central cations from ref. 42. The computed *f*_sym_ values are given in Table 5. Above the arbitrary threshold of 50 kJ mol^−1^ Å^−2^, a covalent N–X bond is formed yielding an asymmetric [N–X⋯N]^+^ structure. Ions for which the *f*_sym_ function is below this threshold form symmetric 3c4e [N⋯X⋯N]^+^ bond. The *f*_sym_ calculated for the 3c4e bond of the carbon-centered **1-C** system^11^ predicts the [N⋯C⋯N]^+^ bond to be symmetric (*f*_sym_ = 31.00 kJ mol^−1^ Å^−2^, where EA(X^+^) = 1086.5 kJ mol^−1^ and *R* = 1.67 Å). This is in excellent agreement with the experimental observations.^11^

**Table 5:** The electron affinity per surface area, *f*_sym_, is a function of the electron affinity of the ion, EA(X^+^), and the radius of the cation, *R*

X	Symmetry	EA(X^+^) [kJ mol^−1^]	*R* [Å]	*f*_sym_ [kJ mol^−1^ Å^−2^]
H^+^	A	1312.1	0.00	∞
F^+^	A	1681.1	1.44	64.51
Li^+^	S	520.2	0.98	43.10
Cl^+^	S	1251.2	1.88	28.17
Na^+^^a^	S	495.9	1.33	22.31
Br^+^	S	1139.9	2.01	22.45
Au^+^	S	890.1	1.99	17.89
I^+^	S	1008.4	2.21	16.43
Ag^+^	S	731.0	1.89	16.28

a Computed data is given for the 1 : 1 complex of **1** and Na^+^.

**Fig. 5:**
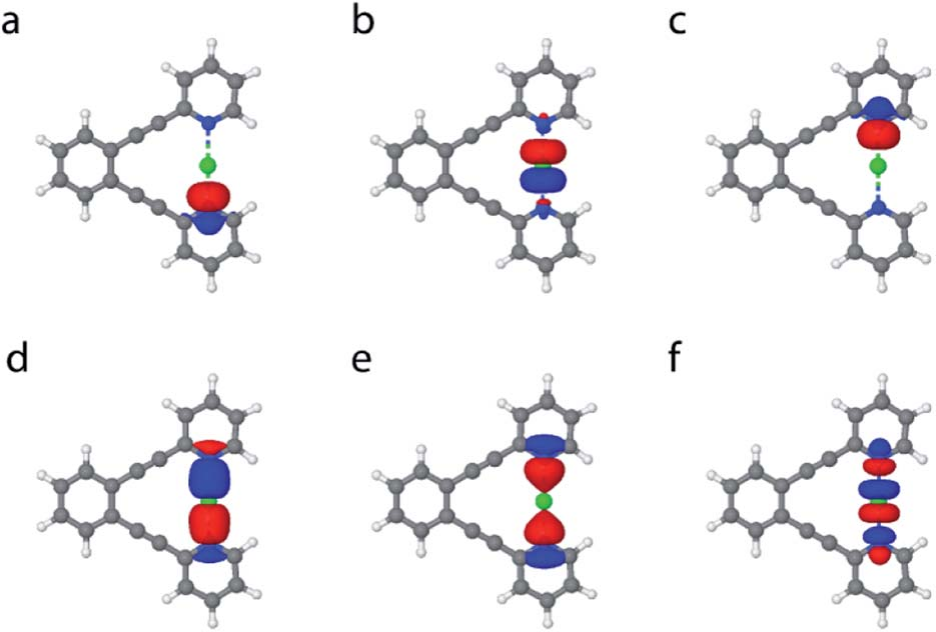
The NBOs involved in the 3c4e bond of **1-Cl**. According to one viable description, (a–c) the two nitrogen lone pairs have occupation numbers of approximately 1.55, while the chlorine p orbital 0.87 for a total of almost 4 electrons involved in the bonding. According to another viable description, (d) bonding, (e) non-bonding and (f) anti-bonding orbitals of a 3c4e bond are formed. Orbitals (d) and (e) are almost doubly occupied, whereas orbital (f) is essentially empty, for a total of almost 4 electrons.

In order to gain further understanding of the bonding situation of the studied complexes, and thus to identify their natural Lewis structure (NLS) in terms of molecular orbitals, we carried out natural bond orbital analyses. A set of NBOs is sought to assign the largest possible amount of electron density to this structure. The most plausible natural Lewis structure thus has the lowest amount of electron density that cannot be placed in its NBOs. For most of the symmetric **1-X** complexes studied here, two approximately equivalent descriptions (set of NBOs) were found (for details see the ESI‡). This is explained here on the example of **1-Cl**, for which a first NBO set containing two nitrogen lone pairs and a partially filled 3p_*z*_ chloronium orbital (Fig. 5a–c) accounts for 96.66% of the electron density, thus leaving out 5.41 electrons (3.34% of the total electron density) from orbitals consistent with the Lewis picture. A second NBO set formed by 3c4e bond molecular orbitals (Fig. 2b, and 5d and e) is also found, not accounting for 4.61 electrons, *i.e.* 2.85% of the total electron density. Thus, the assignment of 3c4e bond character to the [N⋯X⋯N]^+^ complexes is corroborated by the NBO analysis. A similar set of NBOs describes **1-Br** and **1-I**, both halogens hybridizing an empty p-atomic orbital (Fig. 2b), and also **1-Ag** and **1-Au** that hybridize an empty s atomic orbital (Fig. 2a) into the molecular orbital of the three-center bond. These NBOs are shown in Table S11 and Fig. S10–S23 in the ESI.‡ Even though the 3c4e bond orbitals (Fig. 5d–f) best describe the electron density in terms of Lewis structure, the isolated lone pairs NBO description (Fig. 5a–c) has the advantage of allowing a descriptive application of second-order perturbation theory of the Fock matrix.^38^ The latter estimates the interaction strength between different NBOs, and thus provides additional insights into the electronic structure of the ground state. Table 4 reports the interaction strengths between the lone pairs and the partially occupied s and p atomic orbitals of the central the ions for the symmetric **1-X** systems. The columns 3c_b_ and 3c_nb_ show that the 3c4e bond description is well captured by NBO analysis, whereby the bonding and non-bonding orbitals are almost perfectly doubly occupied. The second-order perturbation of the Fock matrix (Table 4) is a good indicator of the interaction energy, with the occupation numbers of the lone pairs quantifying the extent of covalent character of the bond, and the electron delocalization from the nitrogen lone pairs to the empty acceptor orbital of X. A larger occupation of the central atomic orbital (*n*_(s,p)_) can be understood as a bond having larger covalent character, which is also reflected by an associated larger interaction energy. This analysis reveals the dominantly electrostatic character of the weak [N⋯X⋯N]^+^ interaction of **1-Li** and **1-Na**, which may therefore not be seen to form the molecular orbital system typical for 3c4e bonds. The 3c4e bond of the halogen-centered complexes possesses significant covalent character, which increases with decreasing halogen size (Table 4). A less efficient charge transfer from the heavier halogens provides their bond a more electrostatic, and accordingly, more dative character. The opposite trend is observed for transition metals (Table 4), with the bond of the lighter **1-Ag** showing a markedly stronger ionic character than that of **1-Au**.

To further support the chemical bonding analysis, the Wiberg bond orders were computed in the natural atomic orbital basis during the NBO analysis (Table 6). The Wiberg bond orders are between 0.4 and 0.5 for **1-Cl**, **1-Br**, **1-I** and **1-Au**, supporting the three-center four-electron bond picture, whereby the doubly occupied bonding orbital is responsible for “half a bond” between the central atom and each nitrogen atom, whereas the non-bonding orbital does not contribute to the bond order. The bond order of 0.26 computed for **1-Ag** is in agreement with the smallest Δ*G* value (Table 2) among the symmetric three-center, four-electron systems. The bond orders for the complexes containing the two alkali metals are close to zero, confirming the very weak interaction observed by NMR as well as indicated by the Δ*G* and the NBO analysis. The asymmetric systems **1-H** and **1-F** show bond orders larger than 0.65 between the central atom and one nitrogen, and a close to zero bond order for the other nitrogen. This is consistent with the observation of a single covalent bond in an asymmetric geometry.

**Table 6:** Wiberg bond index expressed in the natural atomic orbital basis. The last column is the sum of the bond index between the central atom X and all other atoms of the molecule, thus not simply the sum of the X–N_1_ and X–N_2_ bond indices

X	X–N_1_	X–N_2_	Total X
H^+^	0.66	0.08	0.78
Li^+^	0.06	0.06	0.18
Na^+^	0.04	0.04	0.11
F^+^	0.95	0.01	1.11
Cl^+^	0.50	0.50	1.16
Br^+^	0.47	0.47	1.13
I^+^	0.42	0.42	1.02
Ag^+^	0.26	0.26	0.66
Au^+^	0.42	0.42	1.06

**Fig. 6:**
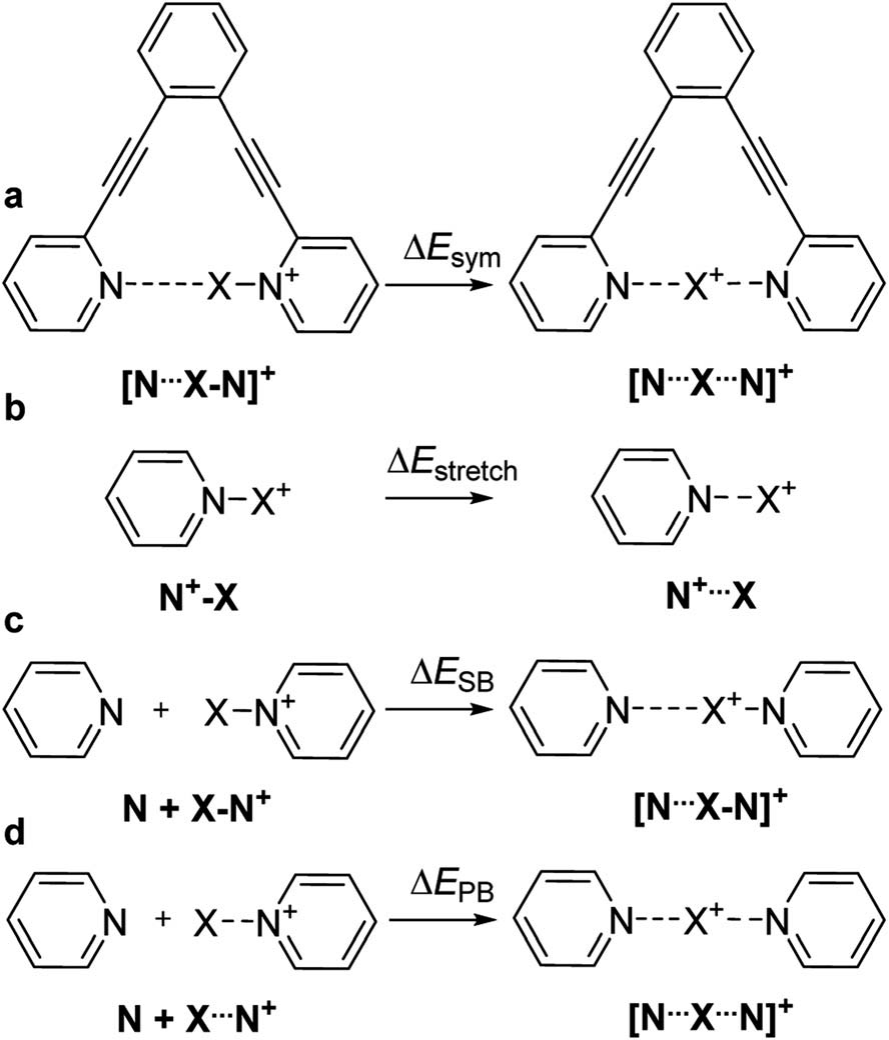
The energetic consequences of formation of a symmetric [N⋯X⋯N]^+^ bond may be characterized (a) by comparison of the electronic energies of the symmetric and asymmetric geometries of the complex **1-X** (Δ*E*_sym_). This energetic gain encompasses, among others, (b) the energy needed for stretching an N–X bond from its covalent bond length to that observed in the corresponding symmetric 3c4e complex (Δ*E*_stretch_) and the energy of a secondary bond, which can be estimated (c) by comparison of the energy of the asymmetric 3c4e [N⋯X–N]^+^ complex **2-X** with the sum of the energies of its constituents, pyridine and pyridinium-X, the latter possessing a covalent N–X bond (Δ*E*_SB_). Alternatively, (d) the energy of one N⋯X bond within a 3c4e [N⋯X⋯N]^+^ complex may be estimated by comparison of the energy of a 3c4e complex (**2-X**) and the sum of the energies of pyridine and pyridinium-X possessing an N⋯X bond stretched to its optimal bond length within a symmetric 3c4e system (Δ*E*_PB_).

We estimated the overall energetic consequence of the formation of a symmetric 3c4e bond, Δ*E*_sym_, by comparing the electronic energies of the symmetric and the asymmetric geometries of the **1-X** complexes (Fig. 6a). Here, the asymmetric geometry possesses a distinct covalent and a distinct noncovalent bond, whereas the symmetric one has two noncovalent bonds with considerable covalent character. Here, Δ*E*_sym_ encompasses several components, such as the energy gain upon establishment of a three-center bond, and the energy loss upon stretching a covalent bond to the bond length found in the symmetric **1-X** geometry (Δ*E*_stretch_, Fig. 6b). The estimated energies are given in Table 7. For all ions but Li^+^ and Na^+^, a stable pyridine-X^+^ geometry was computationally obtained. In agreement with the experiments, the formation of a symmetric three-center bond is overall endothermic for **1-H** and **1-F**, reflecting that their asymmetric geometry is favorable over the symmetric one. A significant part of this energy loss originates from bond stretching, Δ*E*_stretch_ and to a way lesser extent from backbone deformation of **1**, Δ*E*_def_ (Table 2). For the halogen bonded complexes **1-Cl**, **1-Br** and **1-I**, the formation of symmetric 3c4e geometries is associated with an energetic gain. The stretching of the N–X bond is more extensive for chlorine(i) (1.73 Å to 2.01 Å, 14%, Table 1) than for bromine(i) (1.91 Å to 2.13 Å, 10%) and iodine (2.10 Å to 2.30 Å, 9%), which is well reflected by Δ*E*_stretch_ decreasing with increasing halogen size. Whereas Δ*E*_def_ follows the same trend, the difference is smaller (Table 2). As a consequence, despite the somewhat larger interaction energy (Table 2), and the more extensive covalent character (Table 4) of the N–Cl and N–Br bonds as compared to the N–I bond, the overall energetic gain upon formation of a symmetric three-center bond, Δ*E*_sym_, is smallest for **1-Cl**, whereas is comparable for **1-Br** and **1-I** (Table 7). This trend is in agreement with the experimental observation of **2-Cl** only being stable at low temperature in solution, whereas **2-I** and **2-Br** being detectable at room temperature. As the 3c4e N⋯X bond lengths of the transition metals are ∼8% shorter than their covalent bonds (Table 1), the Δ*E*_stretch_ and Δ*E*_sym_ of **1-Au** and **1-Ag** are insignificant, and do not show dependence on the covalent character of the bond (Table 4).

**Table 7:** The energy gain upon forming a symmetric geometry from the corresponding asymmetric complexes (Δ*E*_sym_), and the energy required to stretch an N–X bond from its covalent bond length (*d*_N1–X_) to the bond length in a symmetric **1-X** complex (Δ*E*_stretch_). Δ*E*_SB_ represents the estimated noncovalent, secondary bond interaction energy, presuming X to be covalently bonded to one nitrogen at *d*_N1–X_ bond distance, and forming N⋯X noncovalent bond to the second nitrogen. Similarly, Δ*E*_PB_ is the estimated N⋯X interaction energy, when X is in the predicted equilibrium position. See Fig. 6

X	*d*_N1–X_ [Å]	*d*_N2–X_ [Å]	*d*_N–X_ a [Å]	Δ*E*_sym_ [kJ mol^−1^]	Δ*E*_stretch_ [kJ mol^−1^]	Δ*E*_SB_ [kJ mol^−1^]	Δ*E*_PB_ [kJ mol^−1^]
H^+^	1.050^b^	1.856	1.309^c^	20.6	104.6	−51.1	−51.1
Li^+^	n.a.^d^	n.a.^d^	2.077	n.a.^d^	n.a.^d^	n.a.^d^	−42.6
Na^+^	n.a.^d^	n.a.^d^	2.452	n.a.^d^	n.a.^d^	n.a.^d^	−33.6
F^+^	1.357^b^	2.916	1.793^c^	90.1	143.9	−4.8	−4.8
Cl^+^	1.717	2.421	2.011	−7.8	57.9	−45.7	−128.6
Br^+^	1.876	2.361	2.128	−16.9	38.7	−74.7	−140.1
I^+^	2.090	2.425	2.300	−14.7	23.6	−99.9	−142.6
Ag^+^	2.197	2.149	2.144	−0.6	1.0	−116.3	−118.4
Au^+^	2.034	2.051	2.050	−0.1	0.1	−213.6	−213.9

a Bond length in a symmetric [N⋯X⋯N]^+^ complex.

b Covalent bond lengths taken from the **1-X** complexes.

c Bond length corresponding to the transition state.

d No pyridine–Li/Na geometry with covalent N–Li/Na bond could be identified.

**Fig. 7:**
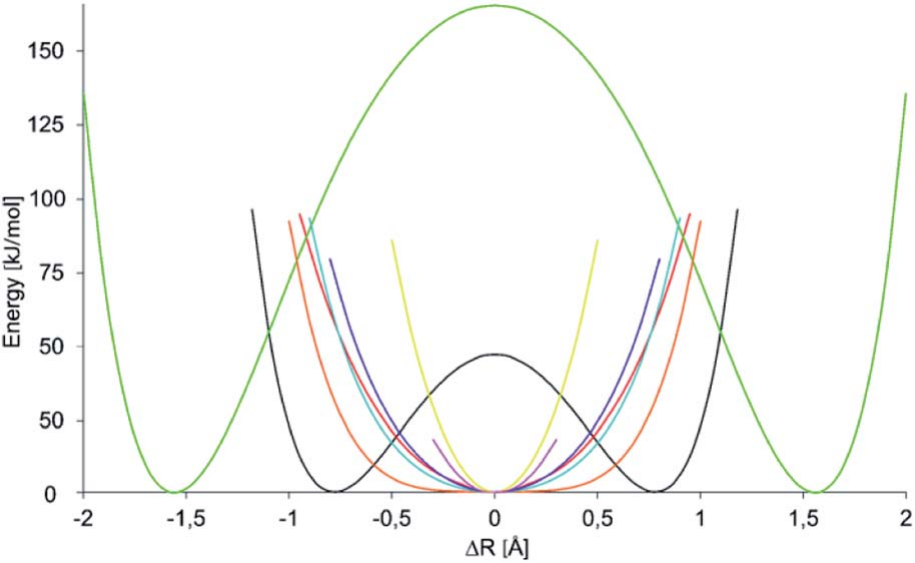
Variation of the electronic energy as a function of the position of atom X in an N–X–N bond. Here, the position of X is described by Δ*R*, the elongation of X from the geometrical midpoint of the donor–donor distance. Hence, at Δ*R* = 0 atom X is in the center, whereas at Δ*R* = 0.5 it is 0.25 Å closer to one of the nitrogens. The energy potential surfaces (PESs) are color coded as **1-H** (black), **1-Li** (red), **1-F** (green), **1-Cl** (orange), **1-Br** (blue), **1-I** (purple), **1-Ag** (pink), and **1-Au** (yellow). No energy potential surface is shown for **1-Na** as it was observed to prefer a different binding mode (Fig. 4), making the interpretation of the PES of a 1 : 1 complex ambiguous. For **1-Ag** only a limited number of points could be calculated. For details of the construction of PESs, see the ESI.‡

A crucial component of the overall energetic change upon formation of a symmetric 3c4e bond in **1-X**, Δ*E*_sym_ is the energetic gain due to formation of a new N⋯X bond. This can be estimated as the energetic consequence of the formation of a secondary bond between the σ-hole of pyridinium-X and the nitrogen of a second pyridine, leading to formation of **2-X** (Fig. 6c). This secondary bond interaction energy, Δ*E*_SB_, provides an estimate for the weak interaction present in the asymmetric **1-H** and **1-F**, which possess a distinct covalent and a distinct secondary bond. Due to the absence of the diethynylbenzene backbone, this estimate is not contaminated by Δ*E*_stretch_ and Δ*E*_def_, but shows the secondary bond energy of these systems. Nonetheless, Δ*E*_SB_ does not provide a sensible description for the interaction energy of an N⋯X bond within a symmetric 3c4e system, in which the central electrophile is bound to the two electron donors equally strongly, through secondary bonds with significant primary bond character. This interaction is better described by Δ*E*_PB_ (Fig. 6d and Table 7) that reflects the energy of one N⋯X bond within a 3c4e complex (**2-X**), without influence of Δ*E*_stretch_ and Δ*E*_def_. This term is vastly exothermic for the complexes of halogens and transition metals, reflecting the primary bond character of their 3c4e bonds. For the transition metal complexes **1-Au** and **1-Ag**, its magnitude correlates to the covalent character (Table 4) and to the interaction energy (Table 2) of the bond. For the halogens, the order of its magnitude qualitatively reflects the experimentally observed stability of **2-X** (and **1-X**) complexes,^22^ and is in agreement with the generally accepted strength order of halogen bonds, whereas it is inversely correlated to their covalent and directly correlated to their electrostatic character.^37^ The small Δ*E*_PB_ computed for **1-Na** and **1-Li** is in agreement with the weak, electrostatic nature of these interactions. This term is not interpretable for **1-H** and **1-F**, for which the symmetric geometry is expected to correspond to a high energy state (Fig. 1a and b). Upon computation of the potential energy surface (PES) of the movement of the central cation in a 3c4e bond, we observed that it depends on the identity of the central ion, X^+^, as demonstrated in Fig. 7. Among the halogens, the widest symmetrical potential energy surface belongs to **1-Cl** whereas the PES of **1-Br** and **1-I** are gradually tighter, following the order of halogen size and Δ*E*_PB_. In agreement with the experimental data, the PES of **1-F** reflects a static asymmetric geometry with a high energy barrier preventing interconversion. The PESs of transition metals are the tightest, suggesting strong symmetric complexes with low variability of the N–X bond lengths. The stronger bond of **1-Au** as compared to that of **1-Ag** is reflected by a narrower single-well potential. Whereas the PES of **1-H** shows double minima, similar to that of **1-F**, the energy barrier of interconversion of the former is low enough to allow tautomerization, corroborating the solution NMR observations. The width of the PES of **1-Li**, possessing a small and weakly bound cation, is comparable to that of the more strongly bound **1-I**, reflecting the complex dependence of the width of their potential well. Hence, among the halogen bonded complexes the width of the single-well PES geometry of **1-X** complexes on the ionic size and the electron affinity of the central electrophilic atom, X. Furthermore, the stability of the symmetric **1-X** complexes can be predicted by corresponds to the experimentally observed stability order **1-Cl** < **1-Br** < **1-I**. The width of the PES of **1-Li** corresponds to that of **1-Br**, and accordingly we detected the formation of both complexes in solution, but were unable to crystallize them. The transition metal complexes possessing tight PESs are vastly stable, which is well reflected by the applicability of **1-Au** as catalyst for organic transformations.^43^

We obtained statistical trends from solid state observations by collecting the available X-ray structures that possess an N–X–N motif involving heterocyclic Lewis bases from the Cambridge Structural Database (CSD, search June 2020). Out of the 57 structures with an [N–H–N]^+^ synthon (ESI Fig. S33‡) 66% have close to linear, 180 ± 10°, N–H–N bond angle. The strong correlation, *r*^2^ > 0.99, of the two N–H bond distances within the latter 34 [N–H–N]^+^ complexes (ESI Fig. S38‡) could ingenuously be interpreted as an indication of the exclusive prevalence of symmetric [N⋯H⋯N]^+^ hydrogen bonds. However, as X-ray data is intrinsically unreliable regarding the position of hydrogens, this observation of equal N–H distances does not reflect reality, but rather highlights an unjustified bias towards fitting hydrogen bonds symmetric within X-ray structures. Whereas no bis(pyridine)-type Li^+^ complexes have previously been deposited to CSD, all available [N⋯Li⋯N]^+^ complexes of other non-polymeric N-heterocycles are symmetric (*r*^2^ > 0.99, ESI Fig. S40‡), which is in agreement with the symmetric structure of **1-Li** proposed here. Whereas no previously reported Na^+^, F^+^ or Cl^+^-centered [N–X–N]^+^ complexes were found in CSD, those possessing a [N–Br–N]^+^ motif have 2.05–2.25 Å N–Br bond distances, comparable to that predicted for **1-Br** by DFT, and are linear (180 ± 5°, ESI Fig. S34‡) showing a maximum of up to 4% deviation from symmetry (ESI Fig. S42‡). The [N–I–N]^+^ three-center bond of the 70 [bis(pyridine)iodine(i)]^+^ complexes found in CSD show comparable bond distances (2.23–2.5 Å) to that of **1-I** (Table 1). Their [N–I–N]^+^ bonds are linear (ESI Fig. S35‡), symmetric with a maximum of 1.7% deviation from symmetry (Fig. S46‡), with the exception of the [N–I–N]^+^ bonds of nonplanar supramolecular helices. Apart from some bent supramolecular structures, the >500 [bis(pyridine)silver(i)]^+^ complexes available in CSD show <1% deviation from symmetry, and 2.08–2.28 Å N–Ag bond distances, which are in line with our observations for **1-Ag**. Some deviations from linearity (ESI Fig. S36‡) and centrosymmetry may be accounted to strong coordination of counteranions to silver(i), which is not possible to bromine(i) and iodine(i), yielding an *r*^2^ of 0.578 for the correlation of the two N–Ag bond lengths within the complexes (ESI Fig. S50‡). The correlation to symmetry is even stronger when [N–Ag–N]^+^ complexes of all N-heterocycles are included (*r*^2^ 0.828, ESI Fig. S52‡). [Bis(pyridine)gold(i)]^+^ complexes deposited to CSD are linear (ESI Fig. S37‡), and have a maximum of 1.3% deviation from bond symmetry (*r*^2^ > 0.97, ESI Fig. S49‡). The CSD deposited structures of the related bis(pyridine) complexes of Hg(ii), Cd(ii), Te(iii), Er(iii), Zn(ii), Gd(iii), Mn(ii), Fe(ii), Ni(ii), Cr(ii), and Rh(i) are also typically symmetric (*r*^2^ > 0.99, ESI Fig. S51‡), suggesting that the conclusion drawn from the spectroscopic, crystallographic and computational evaluation of selected examples of [N–X–N]^+^ systems is expectably generally valid for three-center, four-electron [N–X–N]^+^ bonds.

## Conclusions

We present the first assessment of the position of the central atom in three-center, four-electron bond [N–X–N]^+^ complexes, and the nature of the bond using NMR spectroscopic, X-ray crystallographic and computational techniques. Most complexes (Cl^+^, Br^+^, I^+^, Ag^+^, Au^+^, Na^+^, Li^+^) prefer static, symmetric and linear geometry in both solution and in solid state. In contrast, the systems with H^+^ and F^+^ as central ions form asymmetric complexes. The H^+^ complex equilibrates through tautomerisation [N⋯H–N]^+^ ⇄ [N–H⋯N]^+^, passing a low energy barrier, 11.3 kJ mol^−1^. A similar halotropic interconversion of the F^+^ complex is hindered by a 95.15 kJ mol^−1^ energy barrier, resulting in static and asymmetric N^+^–F⋯N geometry. This complex has a strong covalent and another weak halogen bond.

The symmetry of the three-center bond is found to depend simultaneously on the size and the electron affinity of the central ion. A high electron affinity of the central electrophile (F^+^ and H^+^; Table 5) promotes an asymmetric structure, whereas low electron affinity (Na^+^ and Li^+^) produces a weak electrostatic bond, and thus both are counterproductive for the formation of a strong, symmetric three-center, four-electron bond. Electrophiles with a large ionic radius are most likely to form complexes with the central atom equidistant from both Lewis bases. However, the ionic radius alone does not determine symmetry, as indicated by the ionic radius of F^+^ (*R*_F^+^_, Table 5; asymmetric complex) being larger than those of Na^+^ and Li^+^ (symmetric complexes).

Our observations do not support the formation of a short and symmetric hydrogen bond, as originally proposed for SSHBs; however, the data reveals a high energetic gain (Δ*G* = −116 kJ mol^−1^, Table 2) and a drastically shortened (∼40%) nitrogen–nitrogen distance upon formation of the [N⋯H–N]^+^ ⇄ [N–H⋯N]^+^ tautomeric mixture. Here, the complexes form asymmetric geometries with distinct covalent and noncovalent character. Neither alkali metals form true three-center, four-electron bonds with pronounced charge transfer character and corresponding molecular orbitals, but instead behave as weak electrostatic complexes. In contrast, formation of three-center, four-electron complexes was observed for halogens and transition metals. These have a balanced electron affinity and comparably large size, allowing efficient charge transfer from two Lewis bases simultaneously. These [N⋯X⋯N]^+^ complexes are static and symmetric. They have unusually strong N⋯X noncovalent bonds that possess significant covalent character.

In this work, we focused on the impact of the central electrophilic atom on the covalent *versus* electrostatic character as well as on the geometry of the three-center, four-electron bond, using a benchmark system that offers two nitrogen donor Lewis bases for the interaction. Future work ought to explore the influence of the Lewis base, by evaluating the geometry and bond characteristics of analogous complexes of ligands possessing S, O, and P donor ligands, for example.

Understanding the factors that govern the bonding, geometry and properties of three-center bonds is critical for their applications in a variety of fields. These studies are expected to improve the understanding of chemical bonding.^11^ As such, these results could improve understanding of reaction mechanisms, such as that of S_N_2.^44^ Furthermore, they will aid synthetic chemistry by improving the development of novel transition metal, hydrogen and halogen bond catalysts.^43,45,46^

## Conflicts of interest

There are no conflicts to declare.

## Supplementary Material

SC-011-D0SC02076A-s001

SC-011-D0SC02076A-s002
