# Supplementary material for: Symmetry of three-center, four-electron bonds
Source: Chem Sci. 2020 Jun 30;11(30):7979–90. doi: 10.1039/d0sc02076a (PMC8163095; doi:10.1039/d0sc02076a)
Supplement: SC-011-D0SC02076A-s001 [file SC-011-D0SC02076A-s001.pdf]

Electronic Supplementary Material (ESI) for Chemical Science.
This journal is © The Royal Society of Chemistry 2020

Supporting Information

Symmetry of Three-Center, Four-Electron Bonds

Ann Christin Reiersølmoen,[†,‖] Stefano Battaglia,[‡,‖] Sigurd Øien-Ødegaard,[§] Arvind Kumar Gupta,[¶] Anne Fiksdahl,[†] Roland Lindh,[‡] Máté Erdélyi[*,‡]

[†] Department of Chemistry, Norwegian University of Science and Technology, Høgskoleringen 5, 7491, Trondheim, Norway
[‡] Department of Chemistry – BMC Uppsala University, Husargatan 3, 752 37, Uppsala, Sweden
[§]Centre for Material Sciences and Nanotechnology, University of Oslo, Sem Sælands vei 26, 0371 Oslo, Norway
[¶] Department of Organic Chemistry – Ångström Laboratory, Uppsala University, Lägerhyddsvägen 1, 751 20, Uppsala, Sweden
[‖] These authors contributed equally to this work.

Table of Contents

1. Experimental Procedures S2
1.1 General Information S2
1.2 Preparation of complexes S2
2. Crystallographic data S4
3. IPE Measurements - ^{13}C NMR Chemical Shifts and Their Temperature Dependence S6
4. Computations S14
4.1. Predicted ^{13}C NMR Chemical Shifts S14
4.2. Bonding character S14
4.3. Computational details on the calculation of ΔE_{sym}, $\Delta E_{stretch}$, ΔE_{SB} and ΔE_{PB} S18
4.4. Potential energy surfaces S19
4.5. Free energy differences S19
5. Spectroscopic Data S20
6. Crystallographic Data Collected from CSD S25
7. References S79

1. Experimental Procedures

1.1 General Information

Commercial grade reagents were used as received. Dry solvents were collected from a solvent-purification system. ^{1}H, ^{13}C NMR, ^{1}H, ^{15}N NMR spectrum were recorded in CD_2Cl_2 or CD_3CN using a 500 or a 600 MHz Bruker Avance Neo spectrometer equipped with TCI or TXI cryogenic probe. Chemical shifts were determined by standard ^{1}H, ^{7}Li and ^{13}C NMR, and ^{1}H, ^{15}N HMBC pulse sequences. Chemical shifts are reported in ppm (δ), using the residual solvent signal, CH_3NO_2 (^{15}N) or LiCl (^{7}Li) as chemical shift reference. Data were collected on a Bruker D8 APEX-II equipped with a CCD camera using Mo Kα radiation (λ = 0.71073 Å)), or on a D8 Venture diffractometer equipped with a CMOS detector. Crystals were mounted on a fiber loop using Fomblin oil. Data reduction was performed with SAINT,[1] absorption corrections for the area detector were performed using SADABS.[2] Structures were solved by direct methods and refined by least squares methods on F2 using the SHELX[3-4] and the OLEX2 software suits.[5-7] The data for **1-H**, **1-Na**, **1-I**, **1-Ag** and **1-Au** were collected at 150(2) K. Non-hydrogen atoms were refined anisotropically, whereas hydrogen atoms were constrained in geometrical positions to their parent atoms. The X-ray structures (cif) of **1-H** (CCDC 1989941), **1-H** (twin, CCDC 19888175), **1-Na** (CCDC 1988176), **1-I** (CCDC 1988177), **1-Ag** (CCDC 1989942) and **1-Au** (CCDC 1988178) have been deposited with the Cambridge Crystallographic Data Centre, where it can be obtained free of charge via http://www.ccdc.cam.ac.uk/conts/retrieving.html, or from the Cambridge Crystallographic Data Centre, 12 Union Road, Cambridge CB2 1EZ, UK. Fax: +44 1223 336 033; or deposit@ccdc.cam.ac.uk.

1.2 Preparation of complexes

<u>(1,2-Bis(pyridin-2-ylethynyl)benzene)proton complex, **1-H**:</u>

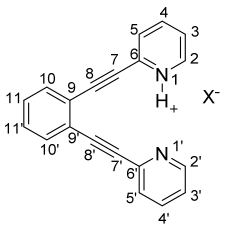

The proton complex of the clamp was prepared by mixing (1,2-Bis(pyridin-2-ylethynyl)benzene (8 mg, 0.029 mmol) and $AuBr_3$×$(H_2O)_x$ (12 mg, 0.029 mmol) in methanol (2 mL). As a result of the bottle with $AuBr_3$ being old and wet, the proton complex was formed, instead of gold coordination. The complex was dried under reduced pressure, before crystals for x-ray analysis were made by dissolving the complex in dichloromethane, followed by diffusion of *n*-pentane into the solution of the complex, CCDC ID: 1989941. Previously reported spectroscopic data for **1-H** with BF_4^- as counterion[8]: ^{1}H NMR (500 MHz, CD_2Cl_2) δ 8.94-8.96 (m, 2H, H2 and H2'), 8.19-8.24 (m, 3H, H4 and H4'), 7.96-8.01 (m, 3H, H5 and H5'), 7.74-7.79 (m, 6H, H10, H10', H3 and H3'), 7.52-7.58 (m, 3H, H11 and H11'); ^{13}C NMR (126 MHz, CD_2Cl_2) δ 147.03 (C2), 146.73 (C2'), 142.93 (C4'), 142.91 (C4), 138.79 (C6), 138.76 (C6'), 133.19 (C10 and C10'), 131.17 (C11 and C11'), 130.04 (C5 and C5'), 125.98 (C3), 125.84 (C3'), 124.83 (C9 and C9'), 95.27 (C7 and C7'), 89.06 (C8 and C8').

<u>[(1,2-Bis(pyridin-2-ylethynyl)benzene)lithium(I)] tetrakis(pentafluorophenyl)borate, **1-Li** :</u>

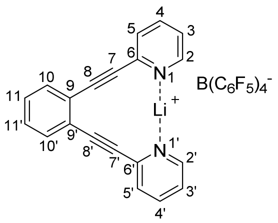

(1,2-Bis(pyridin-2-ylethynyl)benzene (1 equiv.) and lithium tetrakis(pentafluorophenyl)borate ethyl etherate (1 equiv.) were dried and dissolved in dichloromethane under an nitrogen atmosphere. Single crystal for x-ray analysis were attempted grown by slow diffusion of *n*-pentane into a solution of the complex in dichloromethane at room temperature, however no suitable crystals were obtained. ^{1}H NMR (600 MHz, CD_2Cl_2) δ = 8.59 (ddd, *J*=5.1, 1.8, 1.0, 2H, H2 and H2'), 7.85 (td, *J*=7.7, 1.7, 2H, H4 and H4'), 7.68 (ddd, *J*=9.1, 6.8, 2.2, 4H, H10, H10', H3 and H3'), 7.51 (dd, *J*=5.7, 3.3, 2H, H5 and H5'), 7.40 (ddd, *J*=7.6, 5.1, 1.2, 2H, H11 and H11')); ^{13}C NMR (151 MHz, CD_2Cl_2) δ = 149.3 (C2 and C2'), 148.9 (m, br, C-F), 147.3 (m, br, C-F), 141.9 (C4 and C4'), 139.0 (m, br, C-F), 138.5 (C6, C6'), 137.4 (m, br, C-F), 137.1 (m, br, C-F)), 135.5 (m, br, C-F), 132.2 (C10 and C10'), 130.0 (C11 and C11'), 128.2 (C5 and C5'), 124.4 (C3 and C3'), 124.2 (C9 and C9'), 91.5 (C7 and C7'), 89.7 (C8 and C8'); ^{15}N NMR (61 MHz, CD_2Cl_2) δ = -91.3.

<u>[(1,2-Bis(pyridin-2-ylethynyl)benzene)sodium(I)] tetrakis[3,5-bis(trifluoromethyl)phenyl]borate, **1-Na** :</u>

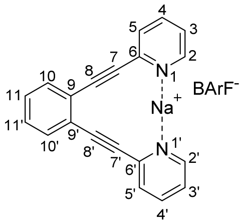

(1,2-Bis(pyridin-2-ylethynyl)benzene (1 equiv.) and LiBArF (1 equiv.) were dried and dissolved in dichloromethane under an nitrogen atmosphere. Single crystal for x-ray analysis were grown by slow diffusion of *n*-pentane into a solution of the complex in dichloromethane under a nitrogen atmosphere at room temperature. CCDC ID: 1988176 ^{1}H NMR (600 MHz, CD_2Cl_2) δ = 8.53 (dt, *J*=5.2, 1.2, 2H, H2 and H2'), 7.77-7,72 (m, 12H, H4, H4', H5, H5' and BArF), 7.62 (d, *J*=7.8, 2H, H3 and H3'), 7.57 (s, 4H, BArF), 7.51 (dd, *J*=5.8, 3.3, 2H, H10 and H10'), 7.29 (ddd, *J*=7.8, 5.0, 1.2, 2H, H11 and H11'); ^{13}C NMR (151 MHz, CD_2Cl_2) δ = 161.7 (dd, *J*=99.6, 49.8, BArF), 149.8 (C2 and C2'), 142.1 (C4 and C4'), 137.8 (C6 and C6'), 134.8 (C10 and C10'), 133.1 (C11 and C11'), 130.0 (C5 and C5'), 129.3 – 128.4 (m, BArF), 127.9 (C3 and C3'), 126.4 (q, *J*=272.9, CF_3-BArF), 124.2 (C9 and C9'), 123.6 (BArF), 117,5 (m, BArF), 91.6 (C7 and C7'), 88.5 (C8, and C8'); ^{15}N NMR (61 MHz, CD_2Cl_2) δ = -83.2.

<u>[(1,2-Bis(pyridin-2-ylethynyl)benzene)silver(I)] tetrafluoroborate, **1-Ag**</u>

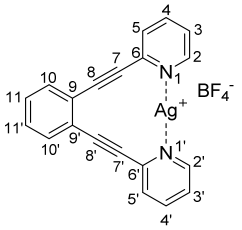

The **1-Ag** complex was prepared following previously reported method.[9] The spectroscopic data corresponds to the data previously reported. Single crystals for x-ray analysis were prepared by slow discussion of *n*-pentane into a dichloromethane solution of the complex at room temperature, CCDC ID 1989942. Previously reported spectroscopic data[9]: ^{1}H NMR (399.95 MHz, CD_2Cl_2) δ 8.92 (ddd, J = 5.4, 1.7, 0.9 Hz, 2H, H2 and H2'), 7.97 (td, J = 7.8, 1.7 Hz, 2H,

H4 and H4’), 7.80 (ddd, J = 7.9, 1.4, 0.9 Hz, 2H, H5 and H5’), 7.70 (dd, J = 5.8, 3.3 Hz, 2H, H10 and H10’), 7.58 (ddd, J = 7.7, 5.4, 1.4 Hz, 2H, H3 and H3’), 7.48 (dd, J = 5.8, 3.4 Hz, 2H, H11 and H11’); ^{13}C NMR (125.71 MHz, CD_2Cl_2) δ 153.6 (C2 and C2´) 143.8 (C6 and C6´), 140.0 (C4 and C4´), 133.6 (C10 and C10´), 130.7 (C11 and C11´), 128.9 (C5 and C5´), 125.8 (C3 and C3´), 124.0 (C9 and C9´), 92.5 (C7 and C7´), 91.2 (C8 and C8´).

[(1,2-Bis(pyridin-2-ylethynyl)benzene)gold(I)] tetrafluoroborate, **1-Au**

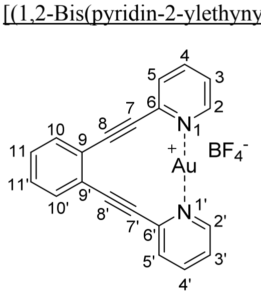

[(1,2-Bis(pyridin-2-ylethynyl)benzene)silver(I)] tetrafluoroborate, **1-Ag**, (8 mg, 0.0016 mmol) was dissolved in dichloromethane (3 mL) under a nitrogen atmosphere. Chloro(dimethylsulfide)gold(I) (5 mg, 0.0017 mmol) was added to the solution, before the reaction mixture was stirred for 1 hour. The reaction mixture was filtered and dried to give the **1-Au** as a pale solid, 6 mg (67%, 0.0011 mmol). Single crystals for X-ray analysis were prepared by slow diffusion of *n*-pentane into a dichloromethane solution of the complex at room temperature, CCDC ID: 1988178. ^{1}H NMR (600 MHz, CD_2Cl_2) δ = 8.87 (ddd, *J*=5.7, 1.6, 0.8, 2H, H2 and H2’), 8.14 (td, *J*=7.9, 1.6, 2H, H4 and H4’), 7.95 (ddd, *J*=8.0, 1.5, 0.8, 2H, H5 and H5’), 7.78 (dd, *J*=5.8, 3.3, 2H, H10 and H10’), 7.68 (ddd, *J*=7.5, 5.7, 1.5, 2H, H3 and H3’), 7.59 (dd, *J*=5.8, 3.3, 2H, H11 and H11’); ^{13}C NMR (151 MHz, CD_2Cl_2) δ = 154 (C2 and C2’), 144.7 (C6 and C6’), 141.3 (C4 and C4’), 133.6 (C10 and C10’), 131.2 (C11 and C11’), 130.4 (C5 and C5’), 126.7 (C3 and C3’), 124.0 (C9 and C9’), 94.5 (C7 and C7’), 90.6 (C8 and C8’); ^{15}N NMR (61 MHz, CD_2Cl_2) δ = -150.1.

[(1,2-Bis(pyridin-2-ylethynyl)benzene)Iodonium(I)] tetrafluoroborate, **1-I**

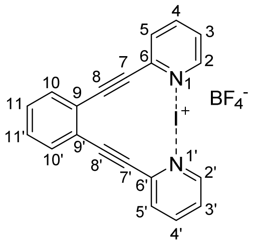

The I^+ complex was prepared following previously reported method.[9] The spectroscopic data corresponds to the data previously reported. Single crystals for X-ray analysis were prepared by slow diffusion of *n*-pentane into a dichloromethane solution of the complex at room temperature, CCDC ID: 1988177. Previously reported spectroscopic data[10]: ^{1}H NMR (399.94 MHz, CD_2Cl_2) δ 8.85 (ddd, J = 5.6, 1.6 and 0.8 Hz, 2H, H2 and H2´), 8.18 (app. td, J = 7.8 and 1.6 Hz, 2H, H4 and H4´), 7.91 (ddd, J = 8.0, 1.4 and 0.8 Hz, 2H, H5 and H5´), 7.76-7.81 (AA´ part of AA´BB´, 2H, H10 and H10´), 7.57-7.62 (BB´ part of AA´BB´, 2H, H11 and H11´), 7.50 (ddd, J = 7.7, 5.6 and 1.4 Hz, 2H, H3 and H3’); ^{13}C NMR (100.58 MHz, CD_2Cl_2) δ 151.27 (C2 and C2´), 143.17 (C6 and C6´), 142.72 (C4 and C4´), 134.89 (C10 and C10´), 131.51 (C11 and C11´), 130.88 (C5 and C5´), 127.05 (C3 and C3´), 124.69 (C9 and C9´), 99.07 (C8 and C8´), 91.10 (C7 and C7´); ^{15}N NMR (40.54 MHz, CD_2Cl_2) δ -165.5.

[(1,2-Bis(pyridin-2-ylethynyl)benzene)Bromonium(I)] triflate, **1-Br**

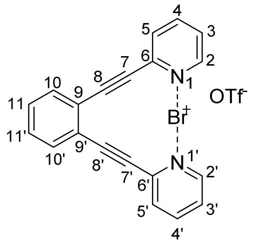

Previously reported spectroscopic data[8]: ^{1}H NMR (500 MHz, CD_2Cl_2) δ 8.88 (ddd, 2H, J = 5.7, 1.5 and 0.7 Hz, H2 and H2’), 8.17 (ddd, 2H, J = 7.9, 7.9, 1.5 Hz, H4 and H4’), 7.91 (ddd, 2H, J = 7.9, 1.4, 0.7 Hz, H5 and H5’), 7.73-7.79 (AA’ part of AA’BB’, 2H, H10, H10’), 7.63 (ddd, 2H, J = 7.9, 5.7, 1.4 Hz, H3 and H3’), 7.56-7.60 (BB’ part of AA’BB’, 2H, H11 and H11’), ^{13}C NMR (126 MHz, CD_2Cl_2) δ 148.48 (C2 and C2’), 142.46 (C4 and C4’), 140.52 (C6 and C6’), 134.24 (C10 and C10’), 131.56 (C11 and C11’), 131.31 (C5 and C5’), 126.85 (C3 and C3’), 124.96 (C9 and C9’), 121.46 (q, J = 319.9 Hz, CF_3), 98.39 (C8 and C8’), 89.03 (C7 and C7’); ^{19}F NMR (376 MHz, CD_2Cl_2) δ -76.78 (s, CF_3).

Pyridine *N*-fluoropyridinium tetrafluoroborate, **2-F**

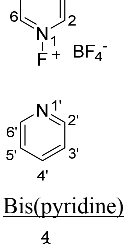

Previously reported spectroscopic data[11]: ^{1}H NMR (499.89 MHz, CD_3CN, -35 °C) δ 9.22 (m, 2H, H-2’), 8.65 (m, 1H, H-4’), 8.55 (m, 2H, H-2), 8.23 (m, 2H, H-3’), 7.75 (m, 1H, H-4), 7.34 (m, 2H, H-3). ^{13}C NMR (125.61 MHz, CD_3CN, -35 °C) δ 150.2 (C-2), 147.7 (C-4’), 137.1 (C4), 131.3 (C-3’), 136.9 (C-2’), 130.8 (C-3’), 124.7 (C-3); ^{19}F NMR (470.3 MHz, CD_3CN, -35 °C) δ 45.9 (N-F), -150.9 (BF_4^-); ^{15}N NMR (50.67 MHz, CD_3CN, -35 °C) δ-70.6 (N-1), -127.9 (N-1’).

Bis(pyridine)chloronium triflate, **2-Cl**

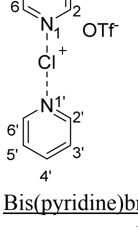

Previously reported spectroscopic data[11]: ^{1}H NMR (499.89 MHz, CD_2Cl_2, -80 °C) δ 8.87 (br s, 4H, H2), 8.46 (br s, 2H, H-4), 8.02 (br s, 4H, H-3); ^{13}C NMR (125.61 MHz, CD_2Cl_2, -80 °C) δ 145.7 (C-2), δ 144.8 (C-4), δ 129.4 (C-3).

Bis(pyridine)bromonium triflate, **2-Br**

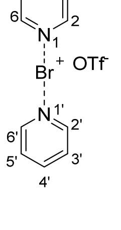

Previously reported spectroscopic data[12]: ^{1}H NMR (500 MHz, CD_2Cl_2) δ 8.73-8.76 (m, 3H, H–2, H–2’, H-6 and H-6’), 8.21-8.26 (m, 2H, H–4 and H–4’), 7.72-7.78 (m, 4H, H-3, H-3’, H–5 and H–5’); ^{13}C NMR (125 MHz, CD_2Cl_2) δ 146.9 (C–2, C–6 and C–6’), 146.6 (t, J = 29.3 Hz, C–6’), 142.7 (C–4 and C–4’), 128.2 (C–3, C–5 and C–5’), 128.1, (C–3’), 121.6 (q, J = 321.1 Hz, CF_3); ^{19}F NMR (376 MHz, CD_2Cl_2) δ -77.27 (s, CF_3)

2. Crystallographic data

Single crystals of **1-H, 1-Na, 1-I, 1-Ag** and **1-Au** were crystallized by slow diffusion of *n*-pentane into a dichloromethane solution of the complexes. Suitable crystals were selected and data for complexes **1-H, 1-Ag** and **1-Au** were collected on a Bruker D8 APEX-II diffractometer equipped with a CCD camera using Mo Kα radiation (λ = 0.71073 Å), whereas for complexes **1-Na** and **1-I** on a D8 Venture diffractometer equipped with a CMOS detector. Crystals were mounted on a fiber loop using Fomblin oil. Data reduction was performed with SAINT,[26] absorption corrections for the area detector were performed using SADABS,[27] or with CrysAlisPro. Structures were solved by direct methods and refined by least squares methods on F2 using the SHELX[28,29] and the OLEX2 software suits.[30-32] The data for **1-H**, **1-Ag** and **1-Au** were collected at 150 K, whereas the **1-Na**, **1-I** data were collected at 100 K. Non-hydrogen atoms were refined anisotropically, whereas hydrogen atoms were constrained in geometrical positions relative to their parent atoms. The X-ray structures (cif) of **1-H** (CCDC 1989941), **1-H** (twin, CCDC 19888175), **1-Na** (CCDC 1988176), **1-I** (CCDC 1988177), **1-Ag** (CCDC 1989942) and **1-Au** (CCDC 1988178) have been deposited with the Cambridge Crystallographic Data Centre.

Table S1. Crystallographic data for **1-H, 1-Na, 1-I, 1-Ag** and **1-Au**.

Compound	**1-H (**twin)	**1-Na**	**1-I**	**1-Au**	**1-H**	**1-Ag**
CCDC #	1988175	1988176	1988177	1988178	1989941	1989942
**Crystal data**						
Chemical formula	$AuBr_4·C_{20}H_{13}N_2$	$C_{40}H_{24}N_4Na·C_{32}H_{12}BF_{24}$	$2(C_{20}H_{12}IN_2)·1.5(CH_2Cl_2)·2(BF_4)$	$1(C_{19.84}H_{12}AuN_2)·0.72(CH_2Cl_2)·BF_4$	$AuBr_2·C_{20}H_{13}N_2$	$C_{20}H_{12}AgN_2·0.75(CH_2Cl_2)·BF_4$
M_r	797.93	1446.85	1115.44	624.28	638.11	537.58
Crystal system, space group	Triclinic, P$\bar{1}$	Orthorhombic, *Pbca*	Triclinic, P$\bar{1}$	Triclinic, P$\bar{1}$	Monoclinic, *P*2$_1$/*c*	Triclinic, P$\bar{1}$
Temperature (K)	150	100	100	150	150	150
a, *b*, *c* (Å)	7.2134 (2), 12.0436 (3), 13.6068 (4)	18.2569 (17), 25.817 (2), 27.739 (3)	7.0108 (4), 14.2732 (8), 21.1647 (12)	6.9430 (4), 14.1421 (9), 21.3969 (14)	7.8505 (11), 19.339 (3), 12.8209 (18)	6.987 (5), 13.924 (10), 21.312 (15)
α, β, γ (°)	113.303 (3), 94.230 (2), 100.284 (2)	90, 90, 90	86.903 (1), 87.804 (1), 77.898 (1)	85.536 (2), 86.970 (2), 78.487 (2)	90, 105.217 (3), 90	85.099 (15), 86.731 (15), 81.161 (15)
V (Å^3)	1054.50 (6)	13074 (2)	2067.0 (2)	2050.9 (2)	1878.2 (5)	2039 (2)
Z	2	8	2	4	4	4
Radiation type	Mo *K*α	Mo *K*α	Mo *K*α	Mo *K*α	Mo *K*α	Mo *K*α
μ (mm^{-1})	14,56	0,14	1,79	7,35	12,09	1,23
Crystal size (mm)	0.14 × 0.08 × 0.06	0.32 × 0.28 × 0.09	0.63 × 0.14 × 0.10	0.15 × 0.09 × 0.06	0.18 × 0.12 × 0.09	0.38 × 0.12 × 0.08

**Data collection**						
Diffractometer	Bruker *APEX*-II	Bruker D8 Venture	Bruker D8 Venture	Bruker *APEX*-II	Bruker *APEX*-II	Bruker *APEX*-II
Absorption correction	Multi-scan	Multi-scan	Multi-scan	Multi-scan	Multi-scan	Multi-scan
T_{min}, T_{max}	0.029, 0.065	0.600, 0.745	0.608, 0.746	0.543, 0.745	0.603, 0.745	0.450, 0.745
No. of measured, independent and observed [$I > 2\sigma(I)$] reflections	47154, 8013, 6509	72074, 8832, 6451	37838, 10247, 8677	75183, 7250, 5740	31369, 3320, 2345	19247, 4972, 3458
R_{int}	0,047	0,07	0,029	0,057	0,096	0,086
θ_{max} (°)	33,1	22,8	28,3	25	25	22
$(\sin \theta/\lambda)_{max}$ (Å^{-1})	0,769	0,545	0,668	0,595	0,595	0,526
**Refinement**						
$R[F^2 > 2\sigma(F^2)]$, $wR(F^2)$, S	0.041, 0.113, 1.03	0.110, 0.250, 1.16	0.045, 0.124, 1.04	0.034, 0.085, 1.06	0.047, 0.121, 1.05	0.083, 0.209, 1.04
No. of reflections	8013	8832	10247	7250	3320	4972
No. of parameters	245	984	620	573	226	643
No. of restraints	0	73	20	54	0	148
$\Delta\rho_{max}$, $\Delta\rho_{min}$ (e Å^{-3})	4.32, −1.04	0.78, −0.44	1.03, −1.32	1.51, −0.88	2.04, −1.18	0.94, −1.17

For **1-H**, the additional scattering (particularly from the electron-rich $AuBr_4^-$ counter-ion) of the twin gives a small systematic error (approximately 1-3%) in the structure factors, which yields increased noise and false peaks in the electron density map. This small but significant error is highly challenging to treat as the signal of the minor twin is too weak to integrate separately. Although the twin law for **1-H** was identified (see Figure S1), its signal could not be separated from that of the main component, neither during data reduction nor refinement. Due to the nature of the disorder, the false peaks appear in a region of the crystal structure where hydrogen placement is a key issue (Figure S1). Such small peaks of apparent electron density could easily be misinterpreted as a proton, or possibly a lithium ion. It should be emphasized that hydrogens cannot be precisely located by X-ray diffraction in structures that contain heavy atoms, such as gold, using standard independent atom model (IAM) refinements, due to the very weak scattering power of hydrogen, and the delocalized electron cloud. While there are recent examples of precise hydrogen position determination using Hirschfeldt atom refinement (HAR)[36] and early quantum crystallography examples,[37] these methods rely on accurate basis sets (HAR), or are under development (QCr). Thus, neutron diffraction remains currently the most reliable method for precisely locating hydrogens in coordination complexes involving heavy atoms.

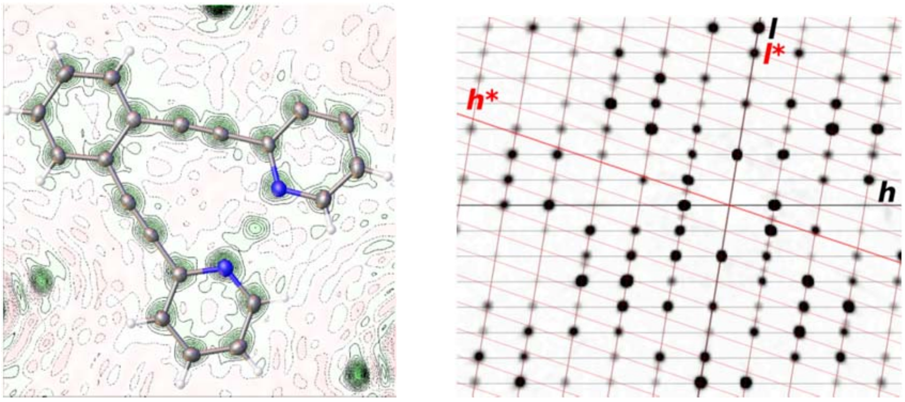

Figure S1. *Left*: Observed electron density map of **1-H** from X-ray diffraction. The accumulation of electron density seen between the two N atoms is an artifact from a minor twin component in the sample, and should not be interpreted as a real atom. *Right:* Precession image of the h0l zone of reflections from **1-H**. The main lattice is indicated by a black grid, the twin lattice by red. The twin law is a 180 degree rotation about the reciprocal c axis (perpendicular to the ab plane of the unit cell of the crystal). The twin domain reflections have approximately 1-3% of the intensity of the main domain.

3. IPE Measurements - ^{13}C NMR Chemical Shifts and Their Temperature Dependence

3.1. General Information

IPE NMR experiments of 2:1 mixtures of mono-deuterated/non-deuterated Ag^+, Au^+, Na^+ and Li^+ complexes were recorded on a 500 MHz Bruker Avance Neo spectrometer equipped with TCI cryogenic probe. The experiments were run with ^{13}C detection at 126 MHz using broadband ^{1}H and inverse-gated ^{2}H decoupling. ^{1}H and ^{13}C NMR spectra of mixtures were recorded for CD_2Cl_2 (δ_H 5.32, δ_C 54.00) in the temperature interval 35 °C to -10 or -35 °C, depending on the solubility of the complex. Upon cooling, the temperature was allowed to stabilize for at least 15 min. The data was then zero-filled to 262144 (256K) points using the software MestreNova V14.1.

3.2. 1-Ag

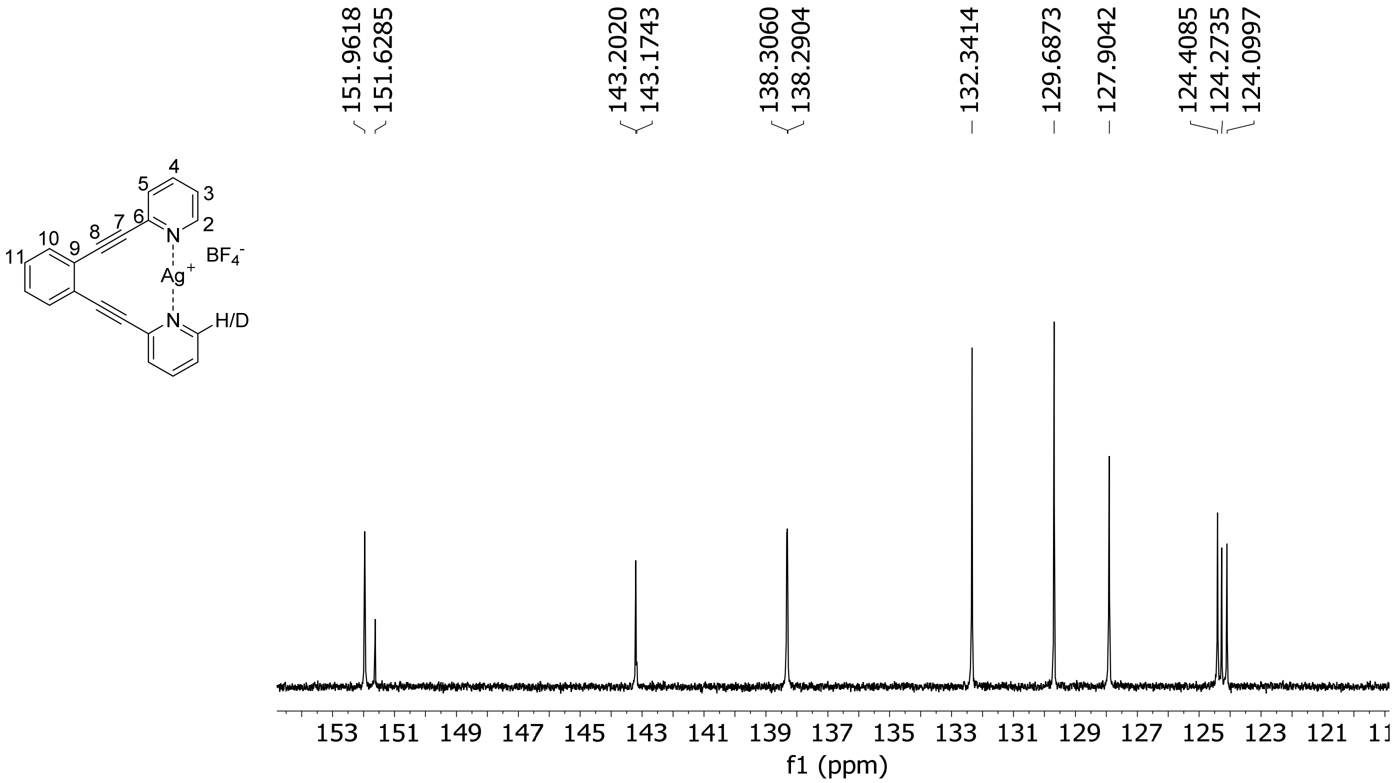

Figure S2. The ^{13}C{^{1}H, ^{2}H} spectrum of **1-Ag/1-Ag-*d*** acquired at 25 °C in CD_2Cl_2 at 126 MHz.

Table S2. The ^{13}C NMR Chemical Shifts of **1-Ag/1-Ag-*d*** given in ppm.

Temp (K)	δ (ppm) C2 (H)	δ (ppm) C2 (D)	δ (ppm) C3 (H)	δ (ppm) C3 (D)	δ (ppm) C4 (H)	δ (ppm) C4 (D)	δ (ppm) C6 (H)	δ (ppm) C6 (D)
303	151.9618	151.6285	124.4034	124.2735	138.3060	138.3260	143.2020	143.1743
293	151.9461	151.6114	124.4151	124.2845	138.3295	138.3509	143.1578	143.1294
283	151.9382	151.6033	124.4307	124.2953	138.3535	138.3752	143.1153	143.0867
273	151.9349	151.5988	124.4400	124.3041	-	-	143.0730	143.0436
263	151.9412	151.6038	124.4546	124.3166	-	-	-	-

Table S3. The temperature dependence of the isotope shifts observed for **1-Ag/1-Ag-*d*** given in ppm.

Temp. (K)	1/T (K^{-1})	$^{1}\Delta_{obs}$ (ppm) $\delta(C2_D\text{-}C2_H)$	$^{2}\Delta_{obs}$ (ppm) $\delta(C3_D\text{-}C3_H)$	$^{3}\Delta_{obs}$ (ppm) $\delta(C4_D\text{-}C4_H)$	$^{3}\Delta_{obs}$ (ppm) $\delta(C6_D\text{-}C6_H)$
303	0.003300	-0.3333	-0.1299	0.0200	-0.0277
293	0.003413	-0.3347	-0.1306	0.0214	-0.0284
283	0.003534	-0.3349	-0.1354	0.0217	-0.0286
273	0.003663	-0.3361	-0.1359	-	-0.0294
263	0.003802	-0.3374	-0.1380	-	-

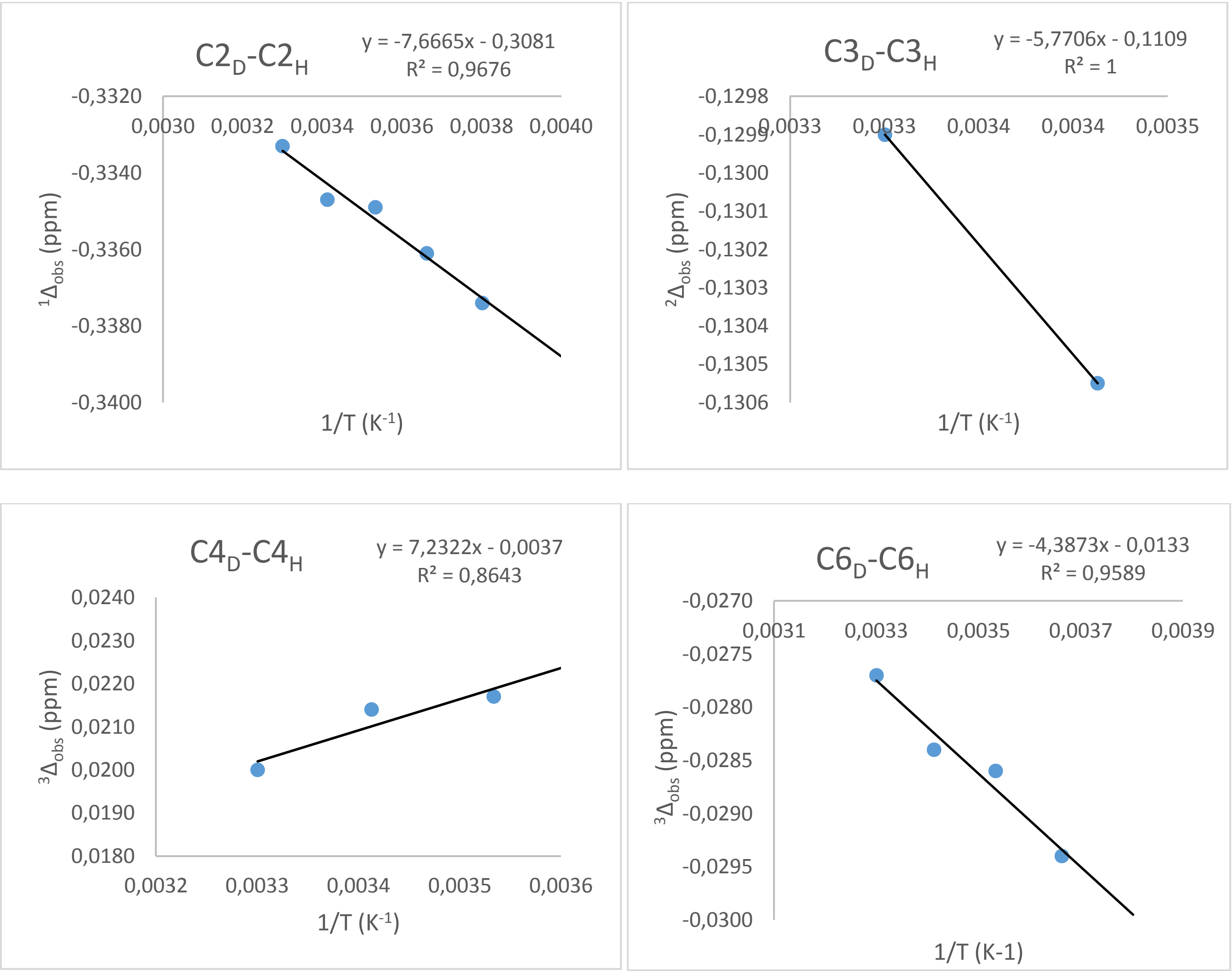

Figure S3. The temperature dependence of the isotope effects of complex **1-Ag/1-Ag-*d*** shown for each carbon separately.

1-Li

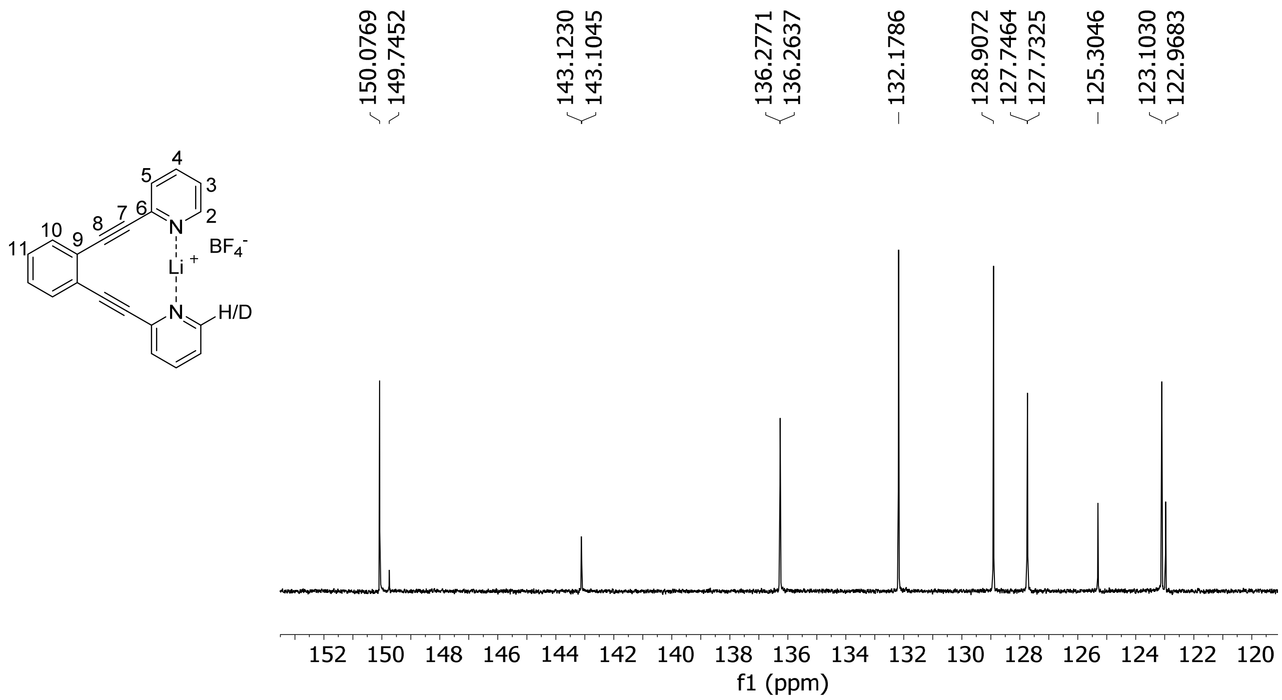

Figure S4. The $^{13}C\{^{1}H, ^{2}H\}$ spectrum of **1-Li/1-Li-*d*** acquired at 30 °C in CD_2Cl_2 at 126 MHz.

Table S4. The ^{13}C NMR Chemical Shifts of **1-Li/1-Li-*d*** given in ppm.

Temp (K)	δ (ppm) C2 (H)	δ (ppm) C2 (D)	δ (ppm) C3 (H)	δ (ppm) C3 (D)	δ (ppm) C4 (H)	δ (ppm) C4 (D)	δ (ppm) C5 (D)	δ (ppm) C5 (D)	δ (ppm) C6 (H)	δ (ppm) C6 (D)
303	150.0769	149.7452	123.1030	122.9683	136.2637	136.2771	127.7325	127.7464	143.1230	143.1045
293	150.0958	149.7630	123.1565	123.0207	136.3250	136.3384	127.7590	127.7731	143.0630	143.0441
283	150.1150	149.7808	123.2113	123.0745	136.3899	136.4036	127.7854	127.7997	143.0009	142.9816
273	150.1344	149.7996	123.2667	123.1287	136.4569	136.4707	127.8111	127.8257	142.9381	142.9184
263	150.1540	149.8181	123.3229	123.1839	136.5265	136.5400	127.8363	127.8511	142.8733	142.8531
253	150.1736	149.8366	123.3842	123.2442	136.6063	136.6202	127.8630	127.8777	142.7988	142.7780
238	150.1953	149.8570	123.4673	123.3262	136.7194	136.7338	127.8988	127.9142	142.6914	142.6696

Table S5. The temperature dependence of the isotope shifts observed for **1-Li/1-Li-*d*** given in ppm.

Temp. (K)	1/T (K^{-1})	$^{1}\Delta_{obs}$ (ppm) $\delta(C2_D-C2_H)$	$^{2}\Delta_{obs}$ (ppm) $\delta(C3_D-C3_H)$	$^{3}\Delta_{obs}$ (ppm) $\delta(C4_D-C4_H)$	$^{4}\Delta_{obs}$ (ppm) $\delta(C5_D-C5_H)$	$^{3}\Delta_{obs}$ (ppm) $\delta(C6_D-C6_H)$
303	0.003300	-0.3317	-0.1347	0.0134	0.0139	-0.0185
293	0.003413	-0.3328	-0.1358	0.0134	0.0141	-0.0189
283	0.003534	-0.3342	-0.1368	0.0137	0.0143	-0.0193
273	0.003663	-0.3348	-0.1380	0.0138	0.0146	-0.0197
263	0.003802	-0.3359	-0.1390	0.0135	0.0148	-0.0202
253	0.003953	-0.3370	-0.1400	0.0139	0.0147	-0.0208
238	0.004202	-0.3383	-0.1411	0.0144	0.0154	-0.0218

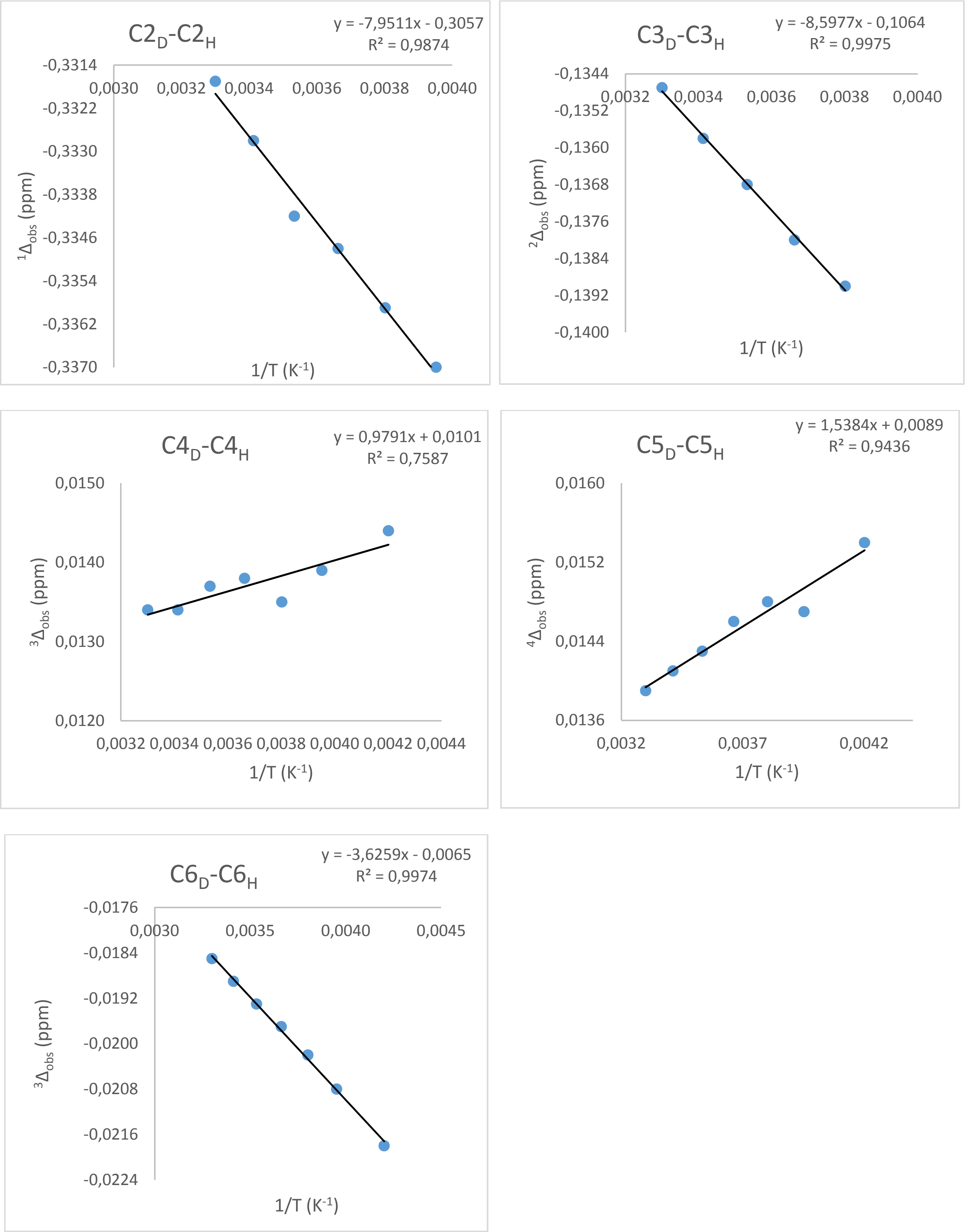

Figure S5. The temperature dependence of the isotope effects of complex **1-Li/1-Li-*d*** shown for each carbon separately.

1-Au

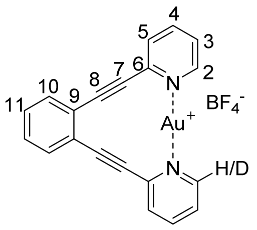

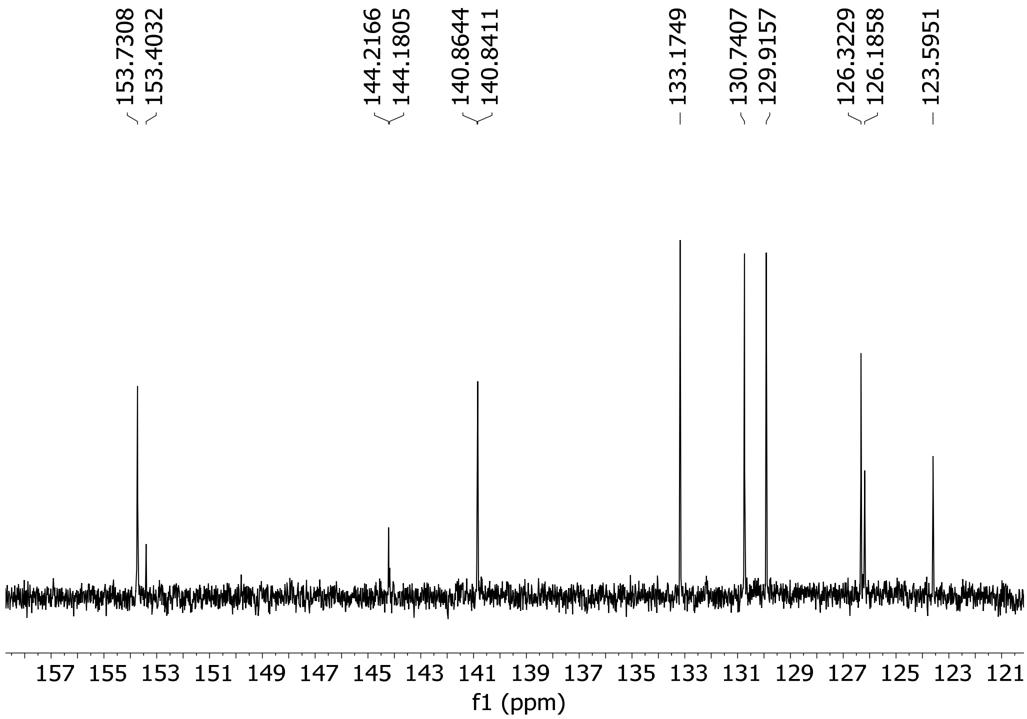

Figure S6. The $^{13}C\{^1H, ^2H\}$ spectrum of **1-Au/1-Au-*d*** acquired at 30 °C in CD_2Cl_2 at 126 MHz.

Table S6. The ^{13}C NMR Chemical Shifts of **1-Au/1-Au-*d*** given in ppm.

Temp (K)	δ (ppm) C2 (H)	δ (ppm) C2 (D)	δ (ppm) C3 (H)	δ (ppm) C3 (D)	δ (ppm) C4 (H)	δ (ppm) C4 (D)	δ (ppm) C6 (H)	δ (ppm) C6 (D)
303	153.7308	153.4032	126.3229	126.1858	140.8411	140.8644	144.2166	144.1805
293	153.6777	153.3492	126.3349	126.1963	140.8652	140.8883	144.1573	144.1195
283	153.6264	153.2976	126.3454	126.2052	140.8860	140.9092	144.0940	144.0562
273	153.5764	153.2462	126.3533	126.2125	140.9029	140.9262	144.0272	143.9894
263	153.5259	153.1961	126.3577	126.2152	140.9141	140.9383	143.9534	143.9148

Table S7. The temperature dependence of the isotope shifts observed for **1-Au/1-Au-*d*** given in ppm.

Temp. (K)	1/T (K^{-1})	$^1\Delta_{obs}$ (ppm) $\delta(C2_D-C2_H)$	$^2\Delta_{obs}$ (ppm) $\delta(C3_D-C3_H)$	$^3\Delta_{obs}$ (ppm) $\delta(C4_D-C4_H)$	$^3\Delta_{obs}$ (ppm) $\delta(C6_D-C6_H)$
303	0.003300	-0.3276	-0.1371	0.0233	-0.0361
293	0.003413	-0.3285	-0.1386	0.0231	-0.0378
283	0.003534	-0.3288	-0.1402	0.0232	-0.0378
273	0.003663	-0.3302	-0.1408	0.0233	-0.0378
263	0.003802	-0.3298	-0.1425	0.0242	-0.0386

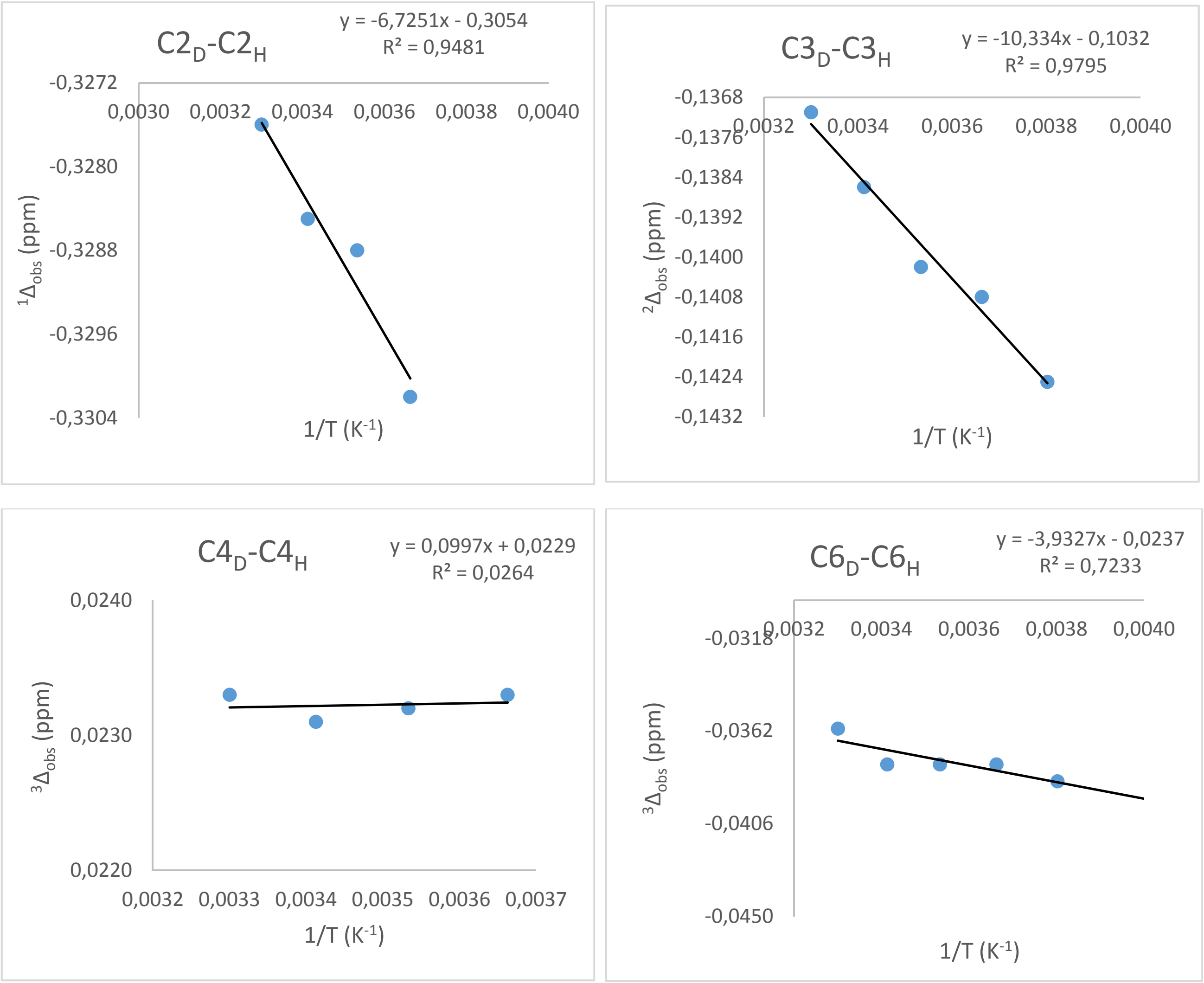

Figure S7. The temperature dependence of the isotope effects of complex **1-Au/1-Au-*d*** shown for each carbon separately.

1-Na

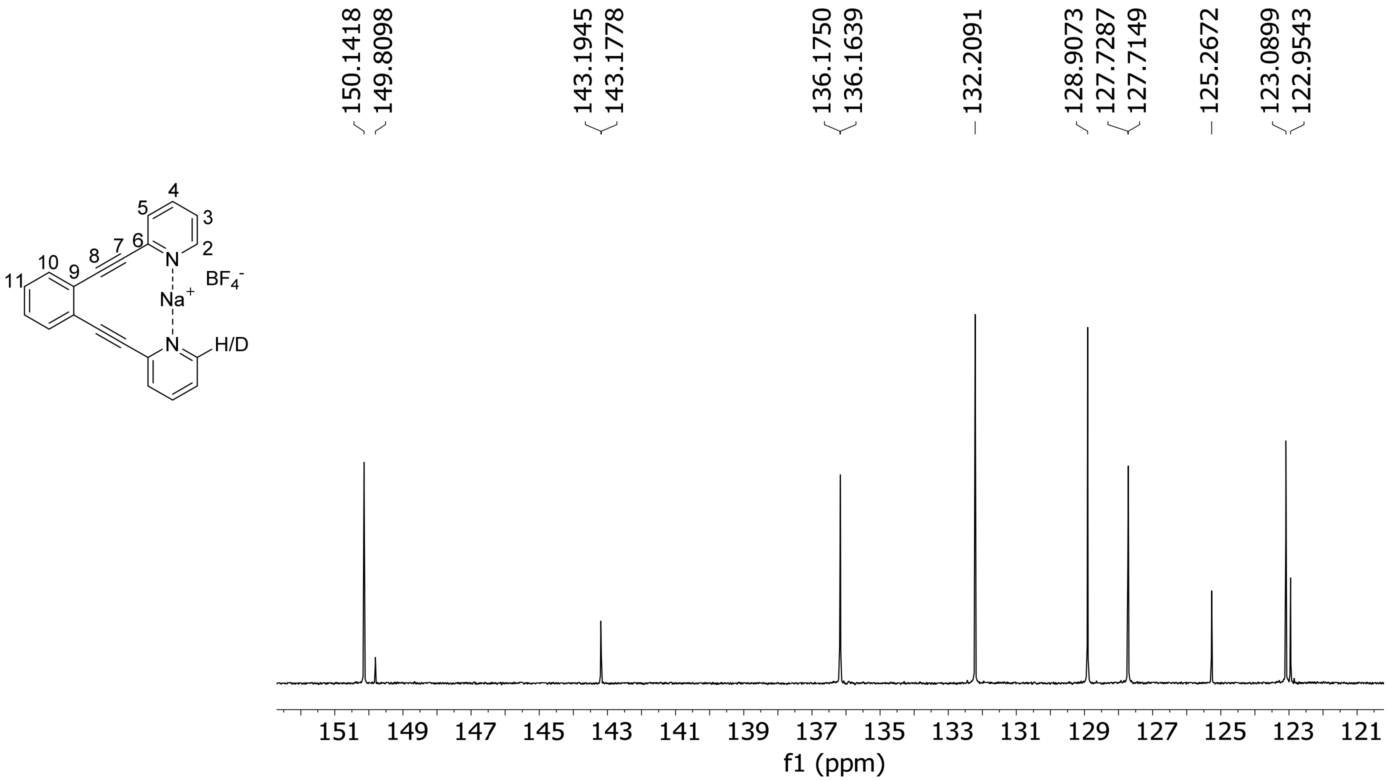

Figure S8. The $^{13}C\{^{1}H, ^{2}H\}$ spectrum of **1-Na/1-Na-*d*** acquired at 30 ºC in CD_2Cl_2 at 126 MHz.

Table S8. The ^{13}C NMR Chemical Shifts of **1-Na/1-Na-*d*** given in ppm.

Temp (K)	δ (ppm) C2 (H)	δ (ppm) C2 (D)	δ (ppm) C3 (H)	δ (ppm) C3 (D)	δ (ppm) C4 (H)	δ (ppm) C4 (D)	δ (ppm) C5 (H)	δ (ppm) C5 (D)	δ (ppm) C6 (H)	δ (ppm) C6 (D)
303	150.1418	149.8098	123.0899	122.9543	136.1639	136.1750	127.7287	127.7149	143.1945	143.1778
293	150.1619	149.8287	123.1425	123.0059	136.2258	136.2371	127.7531	127.7390	143.1352	143.1182
283	150.1830	149.8488	123.1961	123.0582	136.2898	136.3013	127.7783	127.7640	143.0756	143.0582
273	150.2046	149.8692	123.2507	123.1117	136.3559	136.3673	127.8040	127.7895	143.0156	142.9979
263	150.2262	149.8898	123.3064	123.1663	136.4248	136.4363	127.8305	127.8157	142.9548	142.9367
253	150.2487	149.9111	123.3673	123.2264	136.5019	136.5133	127.8598	127.8449	142.8868	142.8682
238	150.2786	149.9395	123.4555	123.3134	136.6165	136.6277	127.9039	127.8887	142.7890	142.7694

Table S9. The temperature dependence of the isotope shifts observed for **1-Na/1-Na-*d*** given in ppm.

Temp. (K)	1/T (K^{-1})	$^{1}\Delta_{obs}$ (ppm) δ($C2_D$-$C2_H$)	$^{2}\Delta_{obs}$ (ppm) δ($C3_D$-$C3_H$)	$^{3}\Delta_{obs}$ (ppm) δ($C4_D$-$C4_H$)	$^{4}\Delta_{obs}$ (ppm) δ($C5_D$-$C5_H$)	$^{3}\Delta_{obs}$ (ppm) δ($C6_D$-$C6_H$)
303	0.003300	-0.3320	-0.1356	0.0111	-0.0138	-0.0167
293	0.003413	-0.3332	-0.1366	0.0113	-0.0141	-0.0170
283	0.003534	-0.3342	-0.1379	0.0115	-0.0143	-0.0174
273	0.003663	-0.3354	-0.1390	0.0114	-0.0145	-0.0177
263	0.003802	-0.3364	-0.1401	0.0115	-0.0148	-0.0181
253	0.003953	-0.3376	-0.1409	0.0114	-0.0149	-0.0186
238	00.4202	-0.3391	-0.1421	0.0112	-0.0152	-0.0196

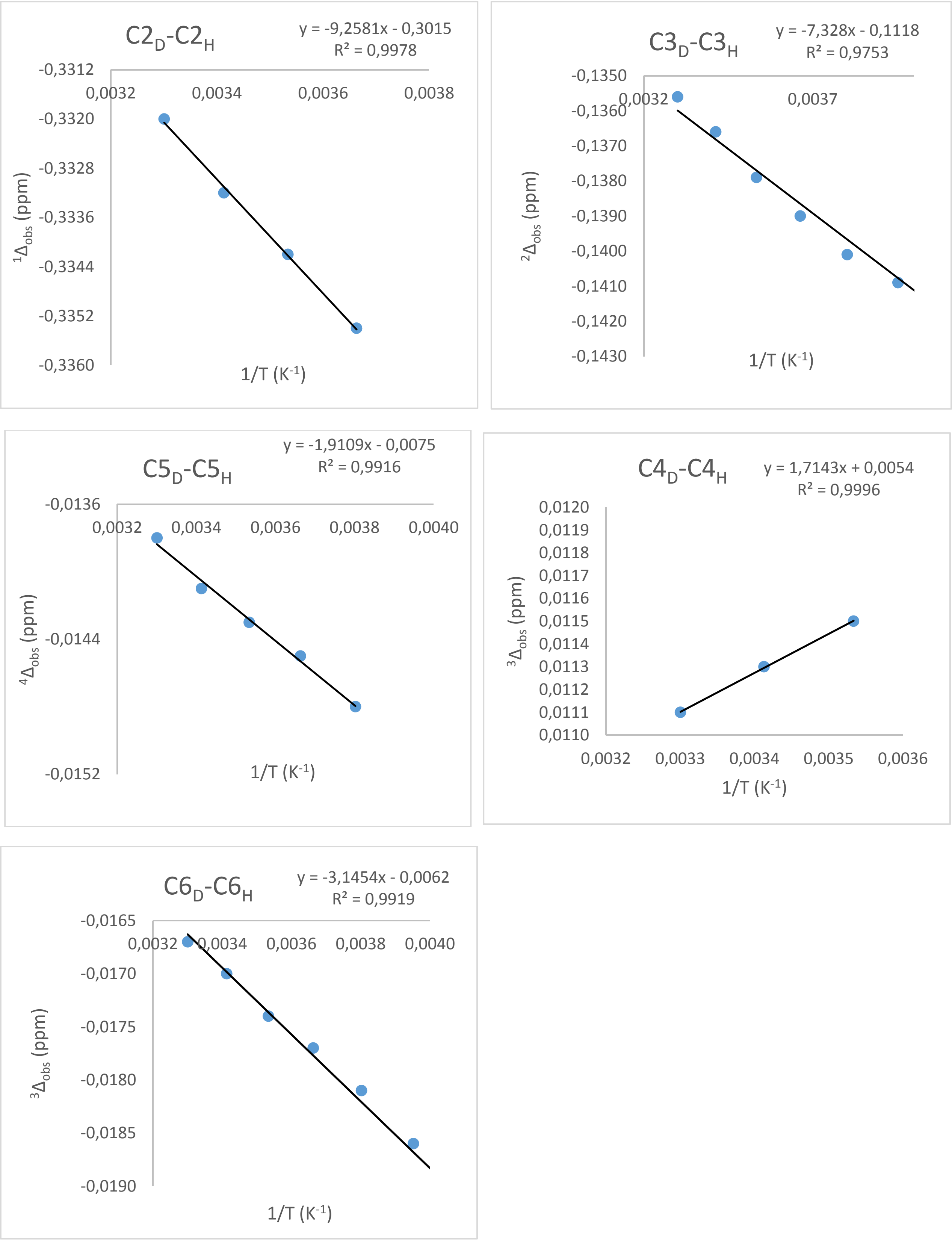

Figure S9. The temperature dependence of the isotope effects of complex **1-Na/1-Na-*d*** shown for each carbon separately.

4. Computations

To corroborate the experimental findings, density functional theory (DFT) calculations were performed. The equilibrium geometries were obtained with the B3LYP exchange and correlation functional[29-32] in conjunction with Dunning's cc-pVTZ correlation consistent basis set[33] on all carbon and hydrogen atoms and the aug-cc-pVTZ basis set[34,35] on the nitrogen atoms as well as the coordinating ions. The MDF[28] Stuttgart/Cologne effective core potential was used for iodine[36] and silver,[37] while the MDF60 was instead adopted for gold.[37] Correspondingly, modified aug-cc-pVTZ-PP basis sets were used for these three ions.[38-40] Solvent effects were taken into account by the polarizable continuum model (PCM)[41,42] using the integral equation formalism variant, with CH_2Cl_2 as a solvent ($\varepsilon = 8.93$) to mirror the experimental setup. The effects of dispersion interactions were considered through the Grimme D3 empirical dispersion correction.[43] The DFT- optimized structures were subsequently used in the calculation of ^{15}N NMR chemical shifts. Prediction of NMR chemical shifts was obtained using the Gauge-independent atomic orbital (GIAO) method.[44] Natural population analysis (NPA) and natural bond orbital (NBO) analysis were carried out on all systems using the NBO 3.1 program.[45]

All calculations were performed using the Gaussian 09 program package, revision d01, setting the integration grid to ultrafine..[46] This particular choice of computational parameters is supported by previous investigations on analogous systems, all in agreement with the experimental findings.[20-23]

4.1. Predicted ^{13}C NMR Chemical Shifts

For all systems considered, the ^{13}C NMR shifts were calculated using the GIAO method. The results are reported in Table S10.

Table S10. The predicted ^{13}C NMR Chemical Shifts of **1-X** complexes given in ppm.

Complex	δ (ppm) C2 (H)	δ (ppm) C2' (H)	δ (ppm) C3 (H)	δ (ppm) C3' (H)	δ (ppm) C4 (H)	δ (ppm) C4' (H)	δ (ppm) C5 (H)	δ (ppm) C5' (H)	δ (ppm) C6 (H)	δ (ppm) C6' (H)
**1**	158.09	158.09	128.32	128.32	142.28	142.28	134.91	134.91	151.30	151.30
**1-H**	149.11	157.34	130.75	130.51	153.06	145.47	137.46	136.18	141.93	148.97
**1-Li**	156.43	156.43	130.30	130.30	145.99	145.99	136.21	136.21	149.84	149.84
**1-Na**	157.14	157.14	130.00	130.00	144.93	144.93	136.19	136.19	150.37	150.37
**1-F**	141.84	157.88	131.06	129.65	151.27	143.03	140.52	136.17	137.92	150.03
**1-Cl**	151.80	151.80	131.10	131.10	148.69	148.69	139.00	139.00	145.23	145.23
**1-Br**	153.95	153.95	131.07	131.07	148.58	148.58	138.86	138.86	146.89	146.89
**1-I**	156.47	156.47	130.94	130.94	148.35	148.35	138.86	138.86	148.73	148.73
**1-Ag**	159.67	159.67	130.68	130.68	146.82	146.82	136.54	136.54	150.78	150.78
**1-Au**	159.43	159.43	130.91	130.91	147.23	147.23	137.15	137.15	150.30	150.30

4.2. Bonding character

To investigate the $[N\cdots X\cdots N]^+$ bonding character we have performed a natural bond orbitals analysis on the **1-X** complexes. For systems **1-Cl**, **1-Br**, **1-I**, **1-Ag** and **1-Au**, two valid sets of natural bond orbitals (NBOs) were found. For all symmetric complexes, the automatic search of the NBO 3.1 program identifies two partially occupied nitrogen lone pairs and a partially occupied atomic orbital on the central ion: the p_z orbital for the halogens, the s orbital for the metals and both the 2s and $2p_z$ orbitals for lithium and sodium. In Table S11 we summarize the important information of the analysis. For **1-H** and **1-F**, the NBO analysis confirms the covalent bond formed by hydrogen and fluorine to one of the pyridines. For **1-Li** and **1-Na**, the partial atomic charges suggest that the alkali metals essentially remain cations and thus only interact electrostatically with the nitrogen lone pairs. This interpretation is supported by the corresponding small ΔE_{PT2} value of the second-order perturbation of the Fock matrix. For **1-Cl**, **1-Br**, **1-I**, there is a clear trend: the lighter the halogen, the more electron density is donated by the nitrogen lone pairs to the halonium ion. This can be seen from the partial atomic charges, as well as from the ΔE_{PT2} value. Larger electron donation to **X** and larger second-order couplings mean a stronger covalent character of the 3c4e bond. For the transition metal complexes we observe the same behavior. More electron density donated to the metal corresponds to a stronger covalent character of the bond, agreeing with the ΔE_{PT2} values. Note that the percentage of Lewis-explained density increases with heavier **X** (even more than the "empty" clamp), but this is ascribed the total number of electrons being larger for heavier elements. Indeed, by looking at the amount of non-Lewis electrons, we notice that the empty clamp has the least of all. Note that almost all of the non-Lewis electron density occupies π* antibonding orbitals localized over the pyridines and the benzene rings and correspond to the resonance structures of the rings.

Table S11. Natural population and orbital analysis. The first three columns correspond to the atomic partial charges obtained by the natural population analysis. The fourth column corresponds to the amount of electron density (in %) that populates natural bond orbitals describing the natural Lewis structure. The fifth column corresponds to the amount of electron density (in number of electrons) that could not be placed in NBOs describing the natural Lewis structure. The last column corresponds to the second-order perturbation of the Fock matrix between the nitrogen lone pair and the accepting, partially occupied NBO of the central ion.

Complex	q_{N1}	q_{N2}	q_X	Lewis ρ (%)	Non-Lewis (e^-)	ΔE_{PT2} (kJ/mol)
**1**	-0.44	-0.44	-	96.94	4.46	-
**1-H**	-0.44	-0.48	+0.47	96.59	4.98	-

**1-Li**	-0.52	-0.52	+0.90	96.94	4.54	56.9[a]
**1-Na**	-0.50	-0.50	+0.95	97.11	4.50	21.3
**1-F**	-0.14	-0.44	+0.13	97.05	4.54	-
**1-Cl**	-0.36	-0.36	+0.17	96.66	5.41	809.2
**1-Br**	-0.42	-0.42	+0.29	97.07	5.28	616.7
**1-I**	-0.47	-0.47	+0.43	97.40	5.15	454.0
**1-Ag**	-0.51	-0.51	+0.73	97.49	4.82	256.9
**1-Au**	-0.48	-0.48	+0.52	97.73	5.09	612.1

[a] Total contribution for the interaction between one lone pair of nitrogen and both the 2s (3s) and $2p_z$ ($3p_z$) orbitals of lithium (sodium).

In Figures S10-S18 we report the natural bond orbitals localized around the $[N\cdots X\cdots N]^+$ bond from the "lone pair" set plotted for an isosurface value of 0.05 au, unless otherwise stated. Note that for the halogens, the accepting orbital is the p_z atomic orbital, while for the metals is the s atomic orbital.

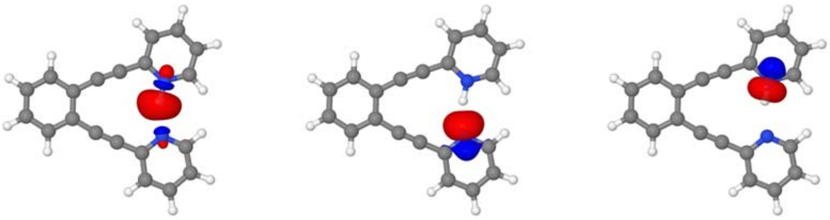

Figure S10. "Lone pair" NBOs for **1-H**. Note that this set of NBOs does not find a bonding orbital between the nitrogen and the hydrogen, but rather the third orbital is considered a pure nitrogen lone pair, donating to the hydrogen 1s orbital displayed in the first image.

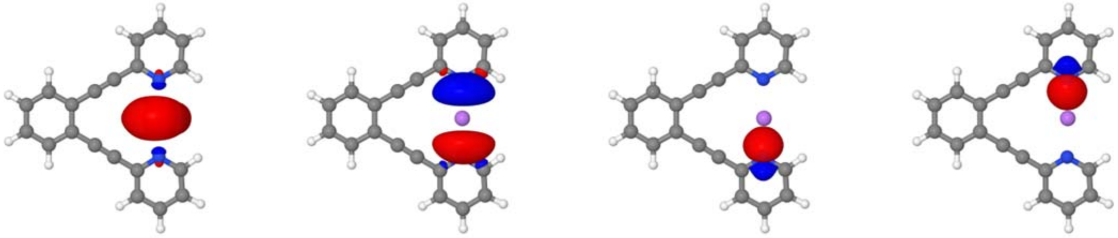

Figure S11. "Lone pair" NBOs for 1-Li. Note that usually only three orbitals are involved, but because the interaction is so weak and the 2s and $2p_z$ orbitals are almost equal in energy, they both receive approximately the same amount of electron density from the nitrogen lone pairs.

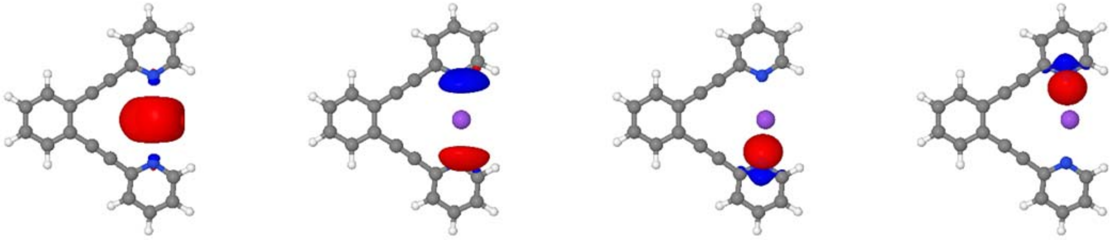

Figure S12. "Lone pair" NBOs for **1-Na**. Note that usually only three orbitals are involved, but because the interaction is so weak and the 3s and $3p_z$ orbitals are similar in energy, they both receive approximately the same amount of electron density from the nitrogen lone pairs.

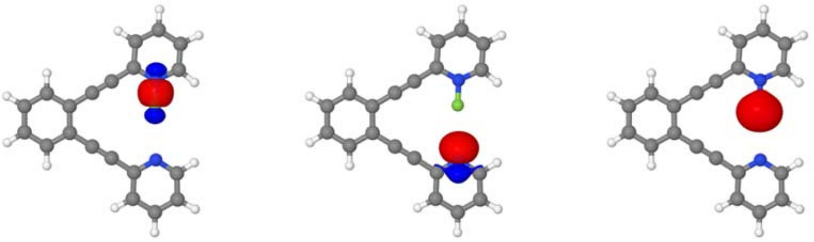

Figure S13. "Lone pair" NBOs for **1-F**.

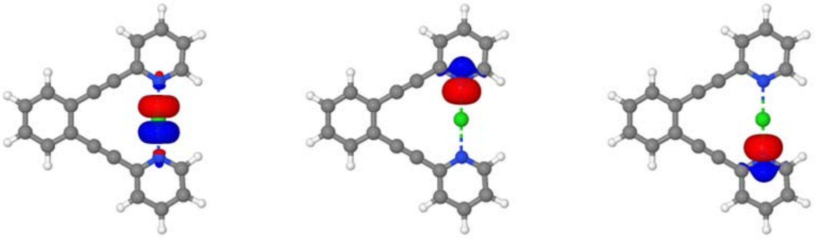

Figure S14. "Lone pair" NBOs for **1-Cl**.

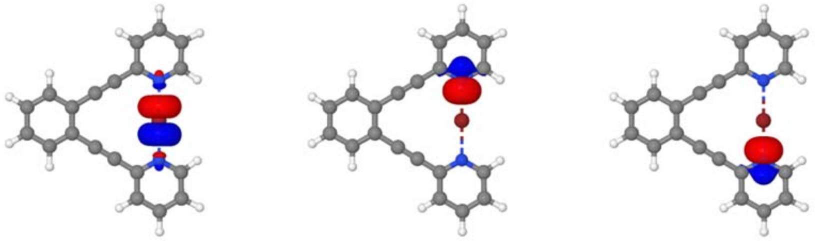

Figure S15. "Lone pair" NBOs for **1-Br**.

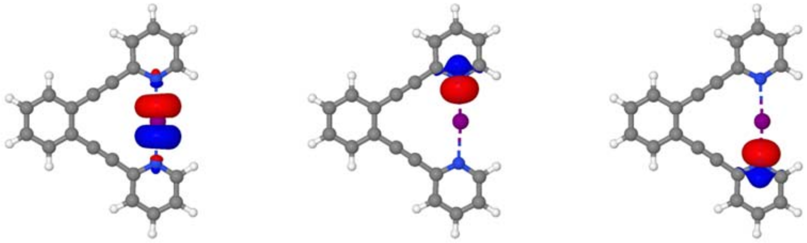

Figure S16. "Lone pair" NBOs for **1-I**.

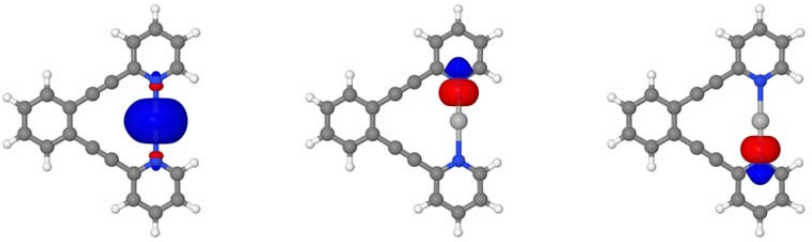

Figure S17. "Lone pair" NBOs for **1-Ag**.

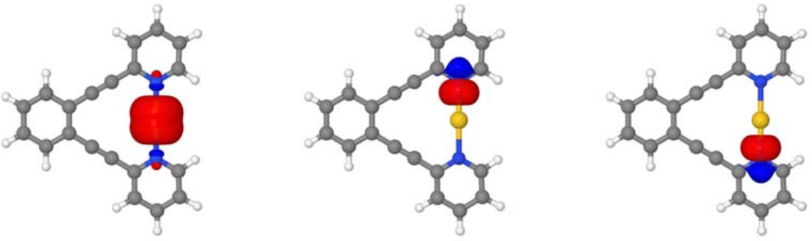

Figure S18. "Lone pair" NBOs for **1-Au** (cutoff 0.047 au).

For all symmetric systems other than **1-Li** and **1-Na**, a second set of NBOs reflecting the Pimentel-Rundle 3c4e-bond picture was identified. This second set was obtained by activating the three-center bond search in the NBO program with the "3CBOND" keyword as well as specifying the

expected Natural Lewis structure (that is, all two-center and three-center bonds as well as all lone pairs) for the various systems. Note that the non-bonding orbital is considered by the program as “non-Lewis”, which is consistent with a three-center, two-electron bond, however, within the three-center, four-electron context, the non-bonding orbital should contribute to the Lewis structure. Thus, the values reported in Table S12 for the electron density percentage in the Lewis structure and the amount of electron density that could not be placed by the NBO set count the non-bonding orbital as being part of the Natural Lewis structure. The relevant information and a comparison with the “lone pair” set is reported on Table S12. For **1-Cl**, **1-Br**, **1-I**, **1-Ag** and **1-Au**, the 3c4e-bond picture accounts for more electron density than the lone pair one. In case of **1-Li** and **1-Na**, no such set of orbitals could be found: further highlighting the weak electrostatic character of the interaction between the alkali metals and the clamp. Note that the natural population analysis does not depend on the natural bond orbitals and is thus the same for both sets. The “disadvantage” of the 3c4e bond with respect to the lone pair set is the loss of relevance of the second-order perturbation of the Fock matrix. Within the 3c4e NBO basis, the interaction between the nitrogens and the central ion is captured by the explicit formation of the 3c4e orbitals, rather than a coupling between the separate lone pairs and the accepting central atomic orbital.

Table S12. Natural population and orbital analysis. We report the electron density percentage in the Lewis structure and the amount of electron density that could not be placed for both sets.

Complex	“lone pair set” Lewis ρ (%)	“lone pair” Non-Lewis (e^-)	“3c4e set” Lewis ρ (%)	“3c4e” Non-Lewis (e^-)
**1**	96.94	4.46	-	-
**1-H**	96.59	4.98	-	-
**1-Li**	96.94	4.54	n.a.[a]	n.a.[a]
**1-Na**	97.11	4.50	n.a.[a]	n.a.[a]
**1-F**	97.05	4.54	-	-
**1-Cl**	96.66	5.41	97.15	4.61
**1-Br**	97.07	5.28	97.44	4.62
**1-I**	97.40	5.15	97.68	4.59
**1-Ag**	97.49	4.82	97.61	4.59
**1-Au**	97.73	5.09	97.94	4.61

[a] Even though **1-Li** and **1-Na** are symmetric, no reasonable NBO set with three-center orbitals was found.

In Figures S19-S23 we report the 3c4e NBOs for the complexes **1-Cl**, **1-Br**, **1-I**, **1-Ag** and **1-Au** plotted with a cutoff of 0.05 au. One can note that for the metals, the s atomic orbital forms the 3c4e NBOs, rather than the p_z in the case of the halogens. This is particularly emphasized for **1-Ag**, for which a less tight cutoff has been used in order to highlight the constructive overlap with the nitrogen lone pairs.

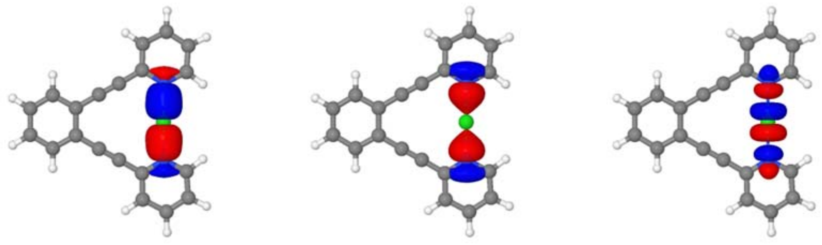

Figure S19. "3c4e” NBOs for **1-Cl**.

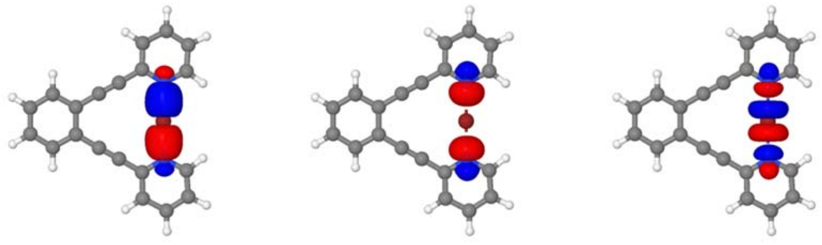

Figure S20. "3c4e” NBOs for **1-Br**.

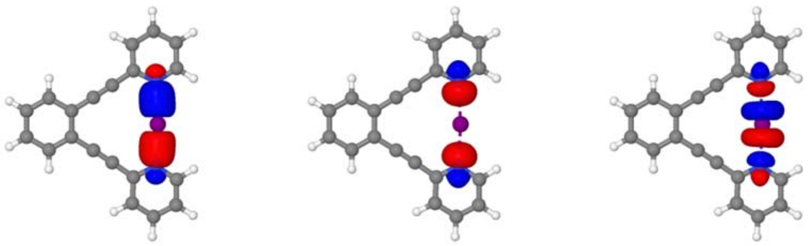

Figure S21. "3c4e" NBOs for **1-I**.

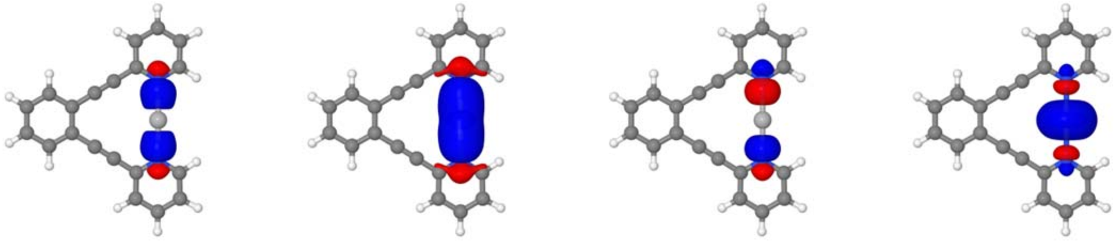

Figure S22. "3c4e" NBOs for **1-Ag**. The second picture is the same as the first one, just with a less tight cutoff of 0.03 instead of 0.05, to emphasize that the 3c4e bonding orbital is formed using the s atomic orbital rather than the p atomic orbital as it is for the halogens. This can also be seen from the orbitals phases.

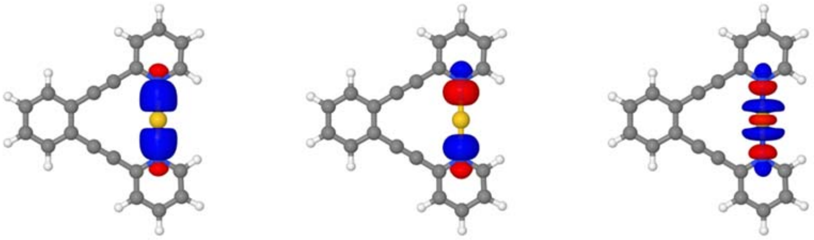

Figure S23. "3c4e" NBOs for **1-Au**.

4.3. Computational details on the calculation of ΔE_{sym}, $\Delta E_{stretch}$, ΔE_{SB} and ΔE_{PB}

To obtain a more quantitative estimation of the energy involved in the 3c4e bond, estimates of the energy required to move the coordinating ion from an asymmetric to a asymmetric position within the clamp, as well as approximate primary and secondary bond energies for the related **2-X** systems have been reported in the main text. This data is reported on Table 6 of the main text and refers to the reactions reported in Figure 7.
First, for all ions studied, an estimate of the "optimal" N-X covalent bond distance is calculated by optimizing the geometry of a *N*-X-pyridinium ionic system, labeled in the following as **pyr-X**. The values found in this way are reported in the d_{N1-X} column of Table 6 in the main text. Note that for **pyr-Li** and **pyr-Na**, no minimum of the potential energy surface corresponding to a covalently bonded system was identified. Furthermore, for the proton and the fluoronium ion, the values corresponding to the optimized **1-H** and **1-F** structures are used to obtain all energies discussed here and reported on Table 6 of the main text.

$\Delta E_{stretch}$, corresponding to Figure 7d of the main text, was calculated by computing the electronic energy difference between the **pyr-X** systems at the "symmetric" N-X bond distance and the optimal one. This value corresponds to the amount of energy required to pull X from its optimal covalent bond distance to the symmetric one found in the **1-X** complexes.
In a similar way, ΔE_{sym}, corresponding to Figure 7a of the main text, quantifies the energy gain/loss in pulling X from the covalent N-X bond distance to the symmetric one within the clamp, i.e. for the **1-X** complexes. Note that in this case, the energy difference is taken between the optimized structure of the **1-X** complexes and the optimized structure of the complexes where the N-X bond distance was kept frozen at the optimal covalent length during optimization, that we shall call **1-X'**. This value is negative for all symmetric **1-X** systems, whereas is positive for **1-H** and **1-F**, which prefer an asymmetric geometry.

ΔE_{SB} (Figure 7b) and ΔE_{PB} (Figure 7c) were obtain in the same way. First, the optimized geometries obtained for **1-X** and **1-X'** are taken, and the backbone connecting the pyridines is cut out. Two hydrogen atoms are used to saturate the dangling bonds and their bond distance is relaxed while keeping the remaining part of the (now) bis(pyridine)-X system frozen. At this point, the interaction energy between the two fragments is computed in the usual way: $E_{complex} – E_{pyr-X} – E_{pyr}$. The ΔE_{PB} values refer to the optimal starting structure, **1-X**, while ΔE_{SB} values refer to the asymmetric starting structures, **1-X'** (which are equal to the optimal one in the case of the proton and the fluoronium ion).
All interaction energies reported in Table 6 are corrected for basis set superposition error by the counterpoise scheme, estimated without accounting the solvent effect due to technical limitation of the software.

4.4. Potential energy surfaces

The potential energy surfaces reported on Figure 8 of the main text correspond to relaxed surface scan starting from the symmetric geometry and decreasing the N-X bond distance in step of 0.05 Å at a time. At each step, the shortened N-X bond is kept frozen during relaxation of the structure. Note that it was not possible to obtain the same amount of data points for all systems, as constraining the N-X bond to too short distances resulted in convergence issues of the geometry optimization.

4.5. Free energy differences

The free energy differences reported in Table 3 of the main manuscript are calculated according to the hypothetical reaction **1** + XBF_4 → [**1-X**]$^+$ + $[BF_4]^-$. First, the geometry of each fragment is relaxed to a minimum of the potential energy surface, confirmed by a subsequent frequency calculation (only exceptions are $BrBF_4$ and $AuBF_4$ for which a spurious imaginary frequency of 8 cm^{-1} and 5 cm^{-1}, respectively, is found, probably due to the approximation introduced by the Grimme dispersion model.) Note that several XBF_4 complexes relax to XF + BF_3.

Then, the Gibbs free energy of [**1-X**]$^+$ is added to that of BF_4^-, constituting one side of the equation. Similarly, the Gibbs free energy of **1** is added to that of XBF_4, constituting the other side of the equation. Finally, the values DG reported in Table 3 are simply obtained by taking the difference between the two sides of the equation. Note that in this way we assume that there is no intermolecular interaction between the fragments.

The reaction [**1-X**]$^+$ + H_2O → [**1-H**] + HOX is computed in a completely analogous fashion, again, neglecting the intermolecular interaction between the fragments.

All these calculations are performed using the same computational details as reported in the main text.

5. Spectroscopic Data

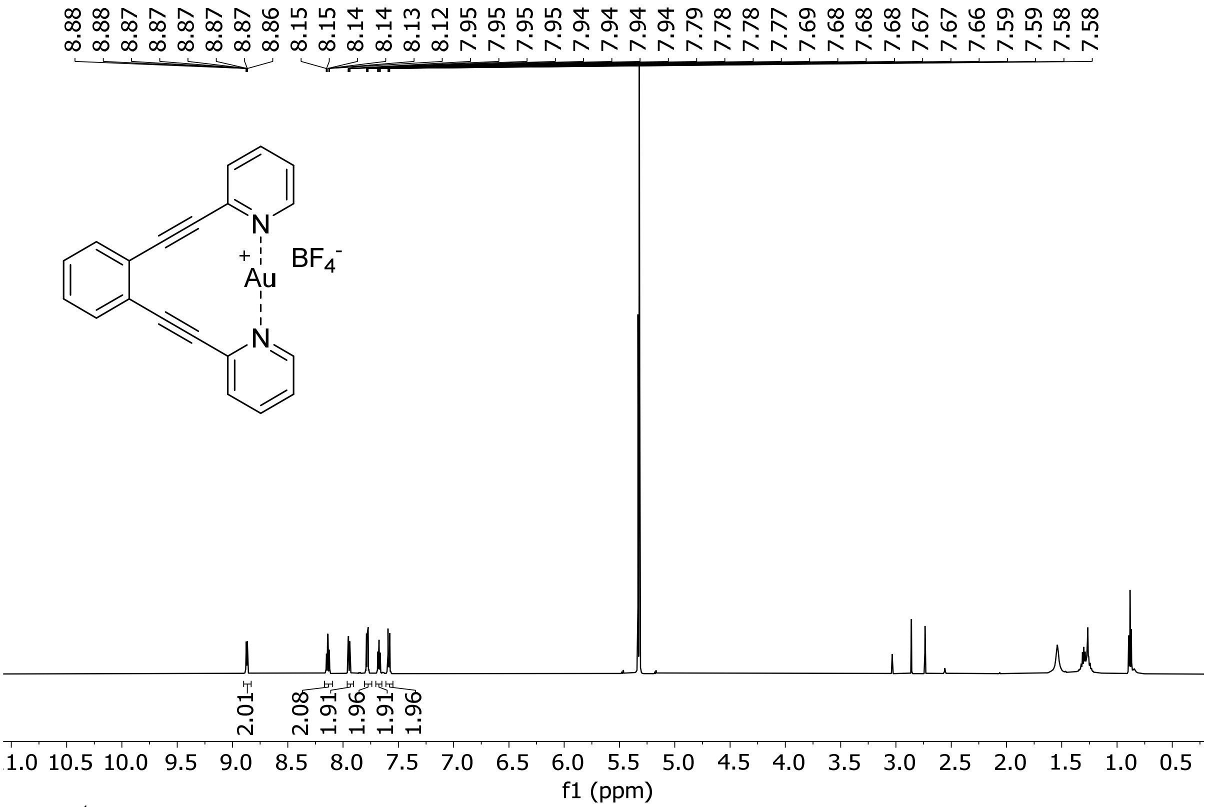

Figure S24. The ^{1}H NMR spectrum of **1**-Au acquired at 25 °C in CD_2Cl_2 at 600 MHz.

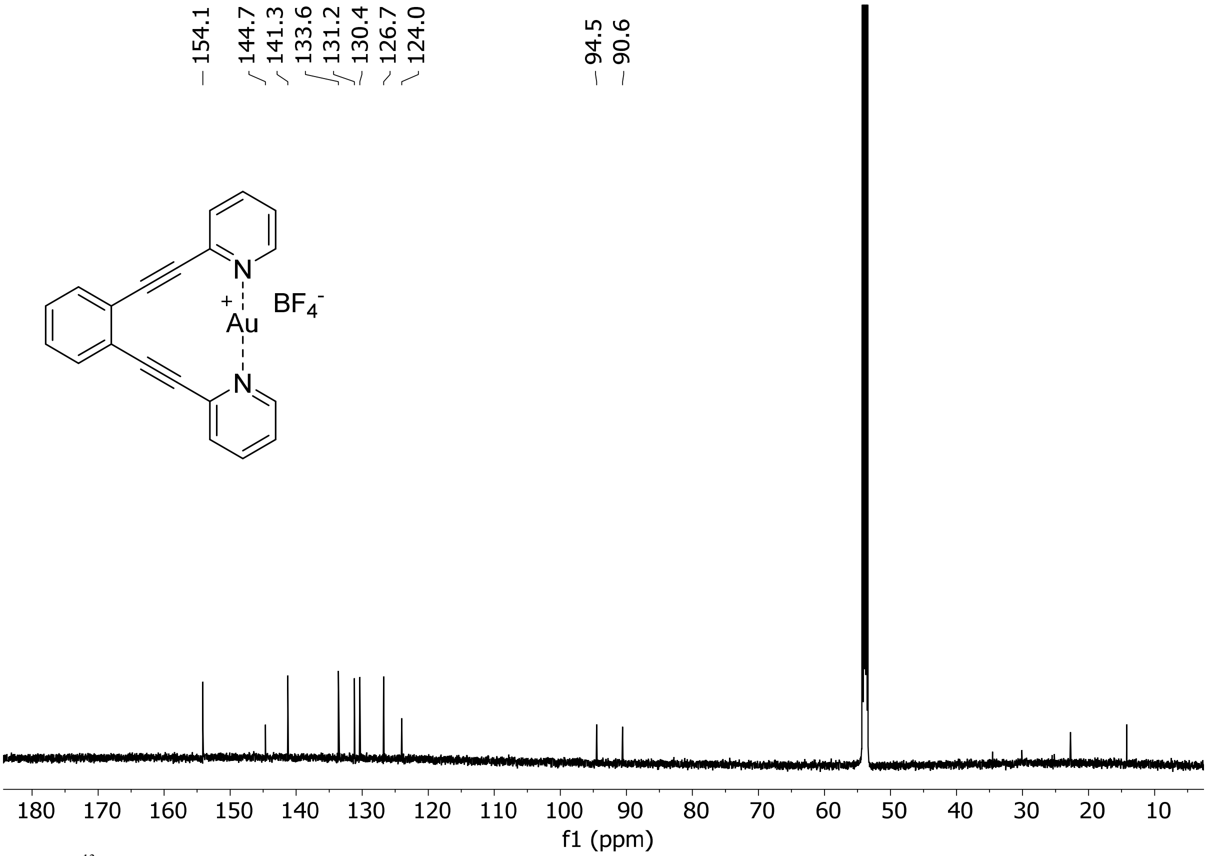

Figure S25. The ^{13}C NMR spectrum of **1**-Au acquired at 25 °C in CD_2Cl_2 at 151 MHz.

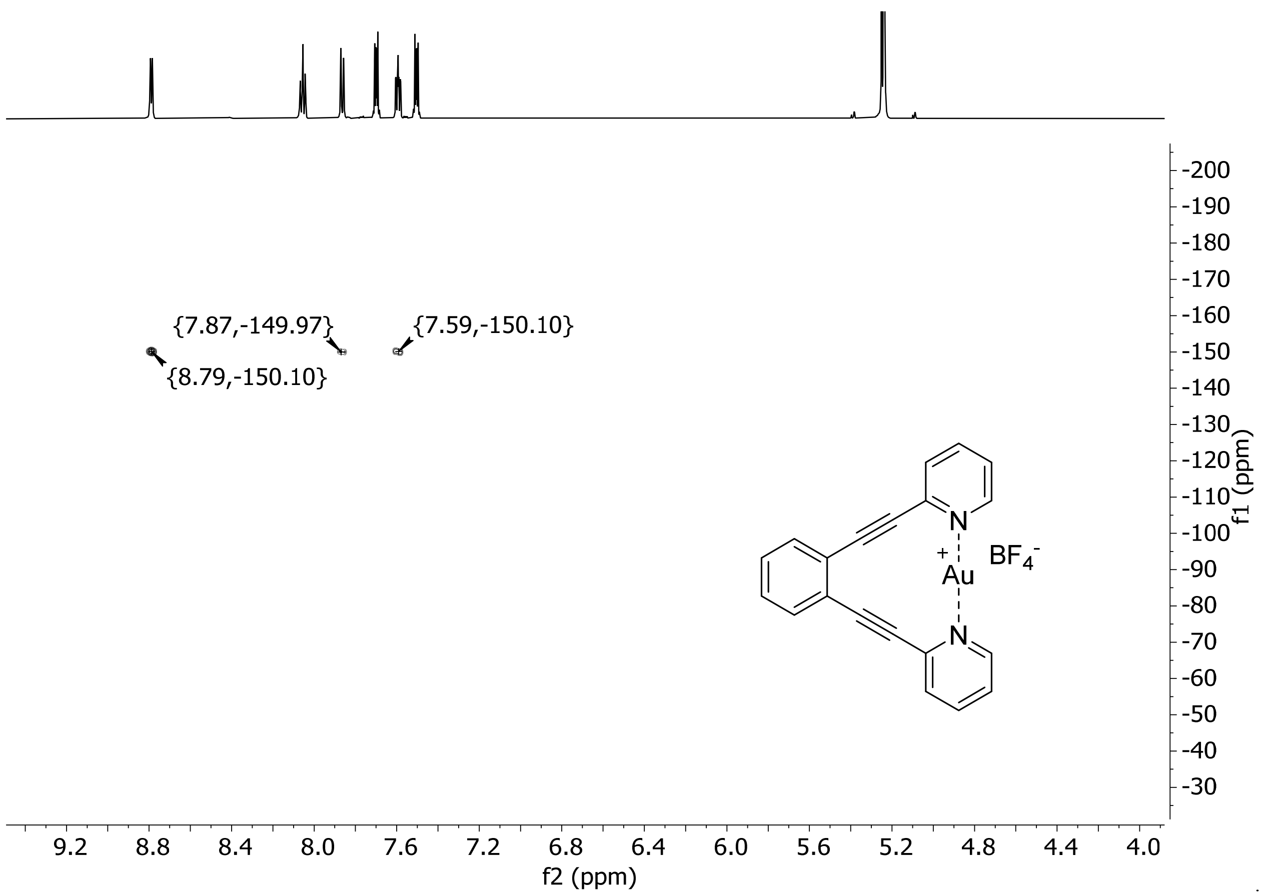

Figure S26. The ^{1}H, ^{15}N HMBC NMR spectrum of **1**-Au acquired at 25 °C in CD_2Cl_2 at 61 MHz.

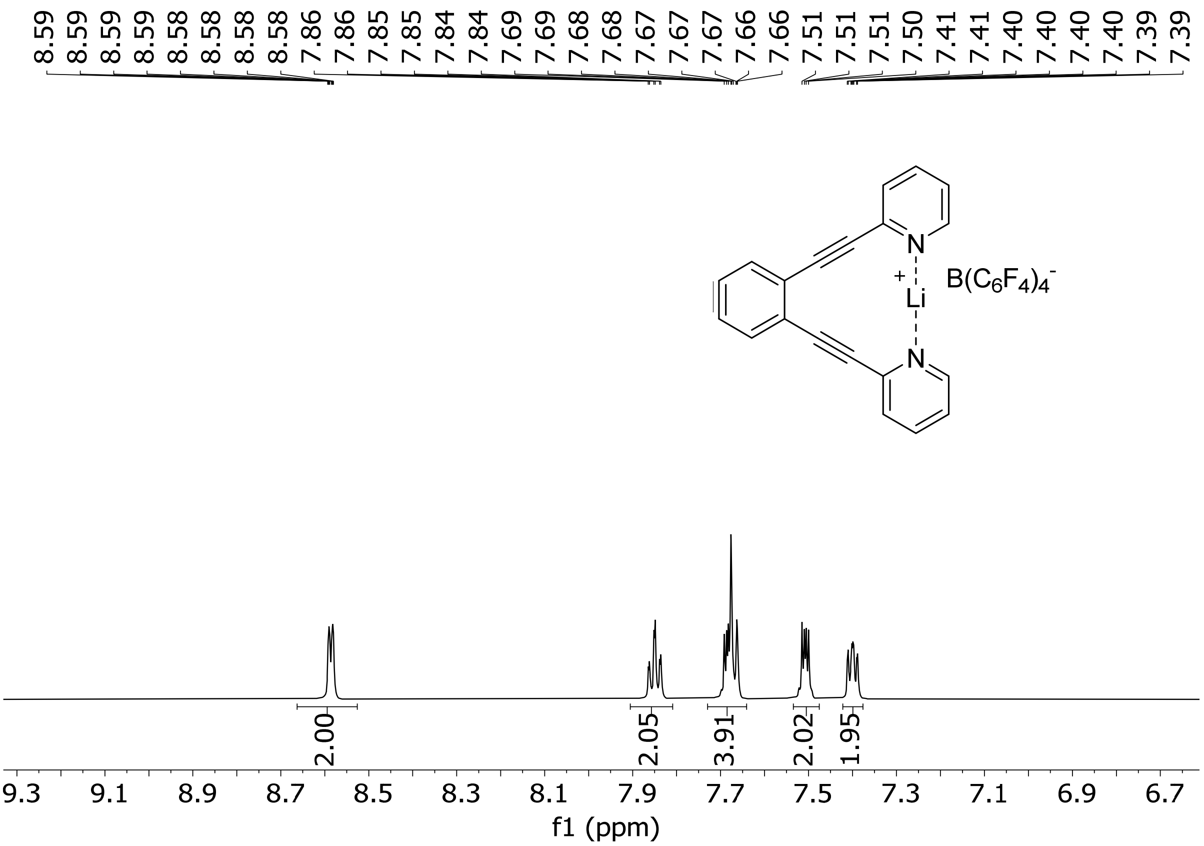

Figure S27. The ^{1}H NMR spectrum of **1**-Li acquired at 25 °C in CD_2Cl_2 at 600 MHz.

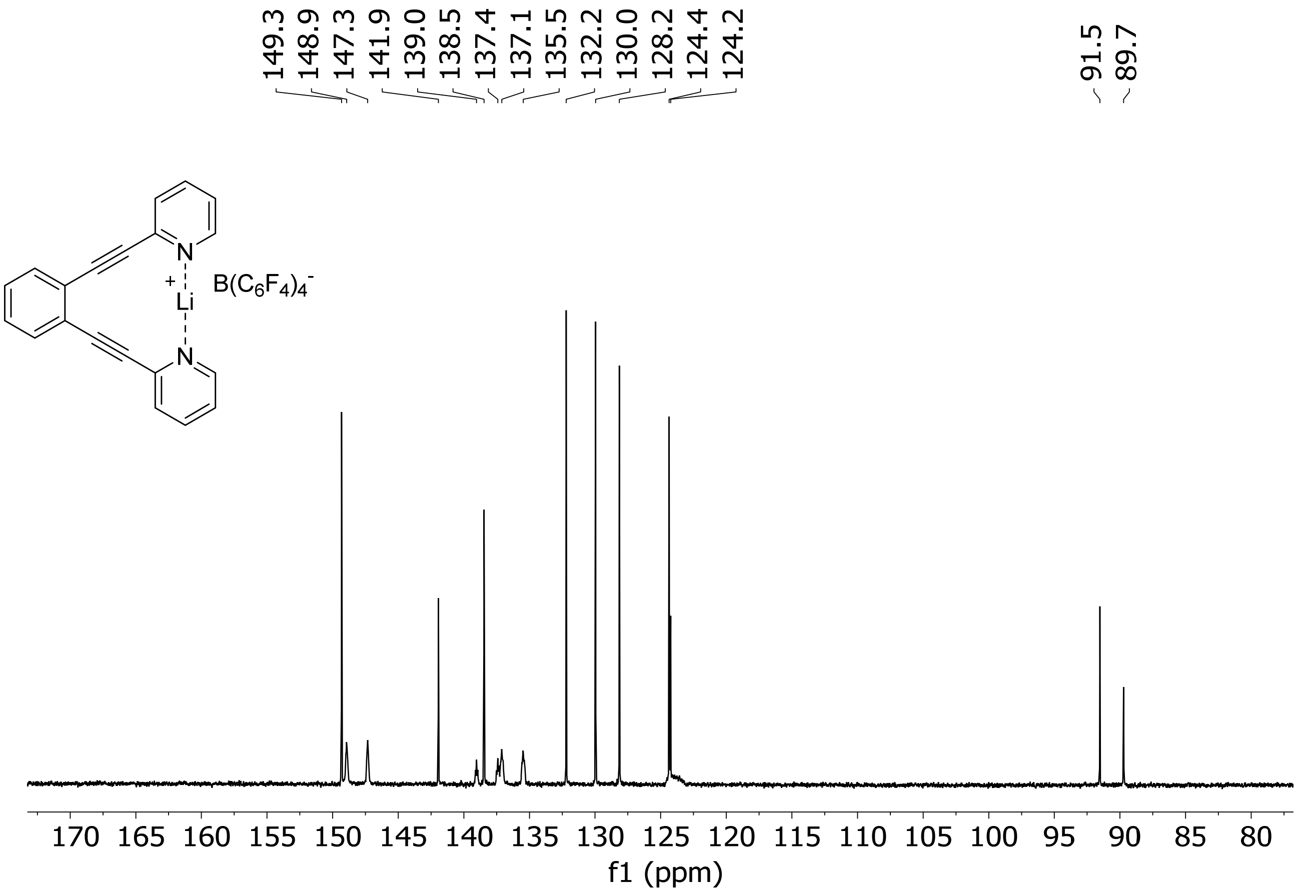

Figure S28. The ^{13}C NMR spectrum of **1**-Li acquired at 25 °C in CD_2Cl_2 at 151 MHz.

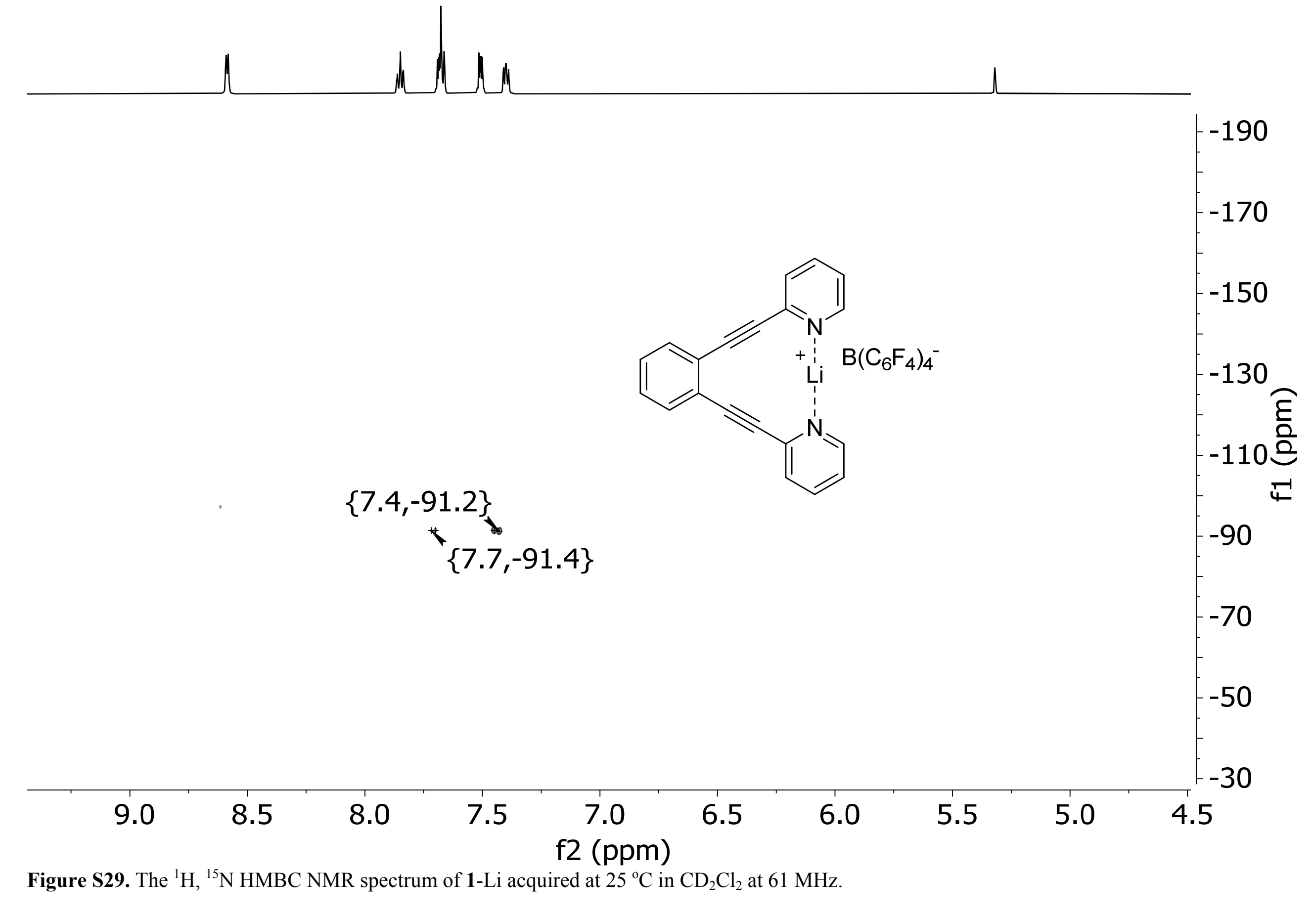

Figure S29. The ^{1}H, ^{15}N HMBC NMR spectrum of **1**-Li acquired at 25 °C in CD_2Cl_2 at 61 MHz.

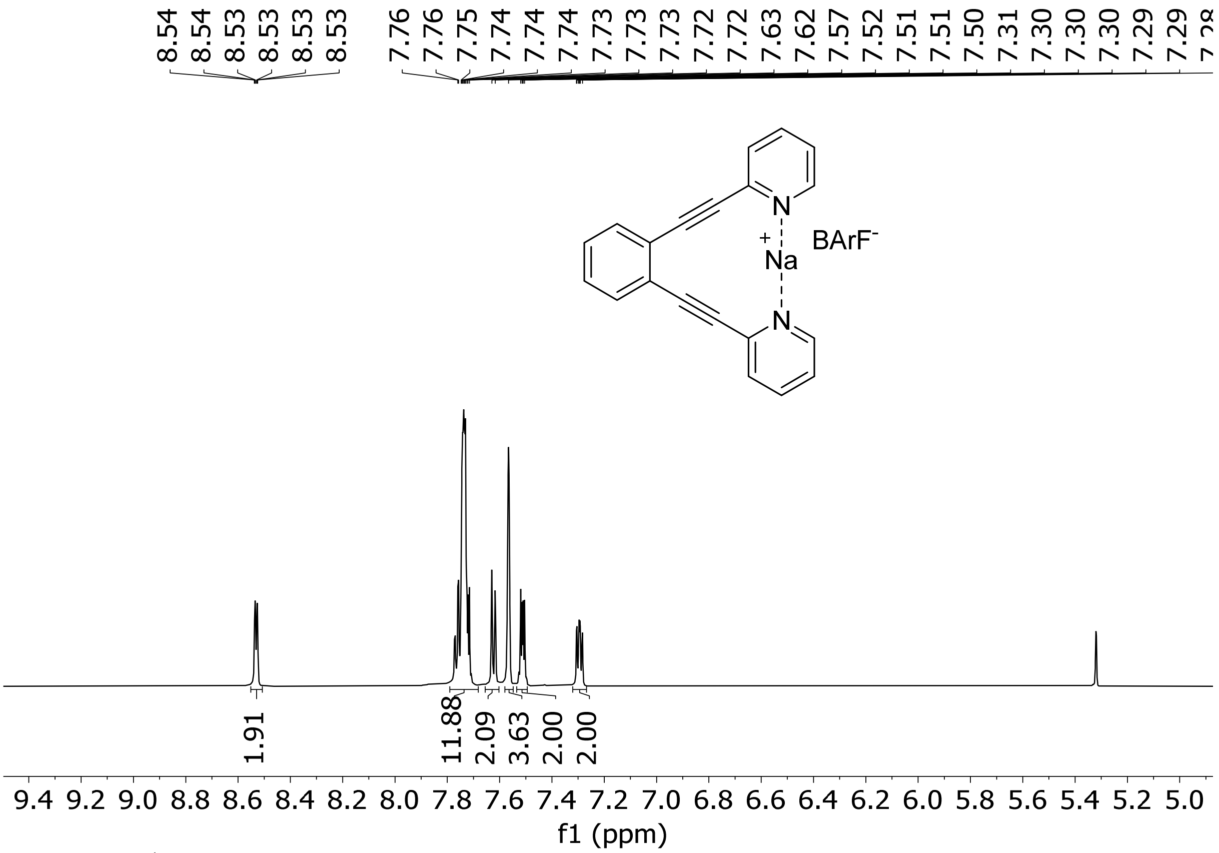

Figure S30. The ^{1}H NMR spectrum of **1**-Na acquired at 25 °C in CD_2Cl_2 at 600 MHz.

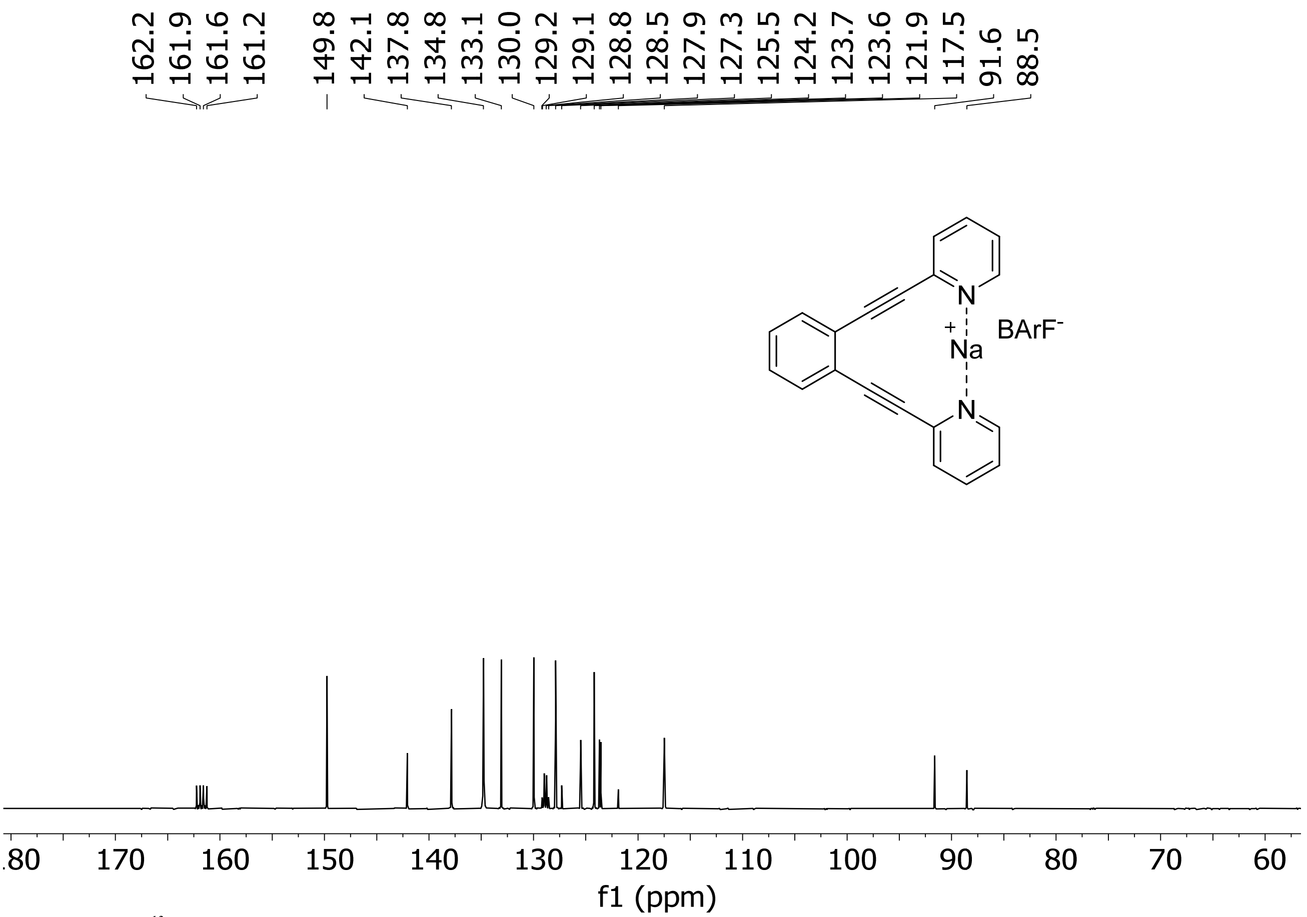

Figure S31. The ^{13}C NMR spectrum of **1**-Na acquired at 25 °C in CD_2Cl_2 at 151 MHz.

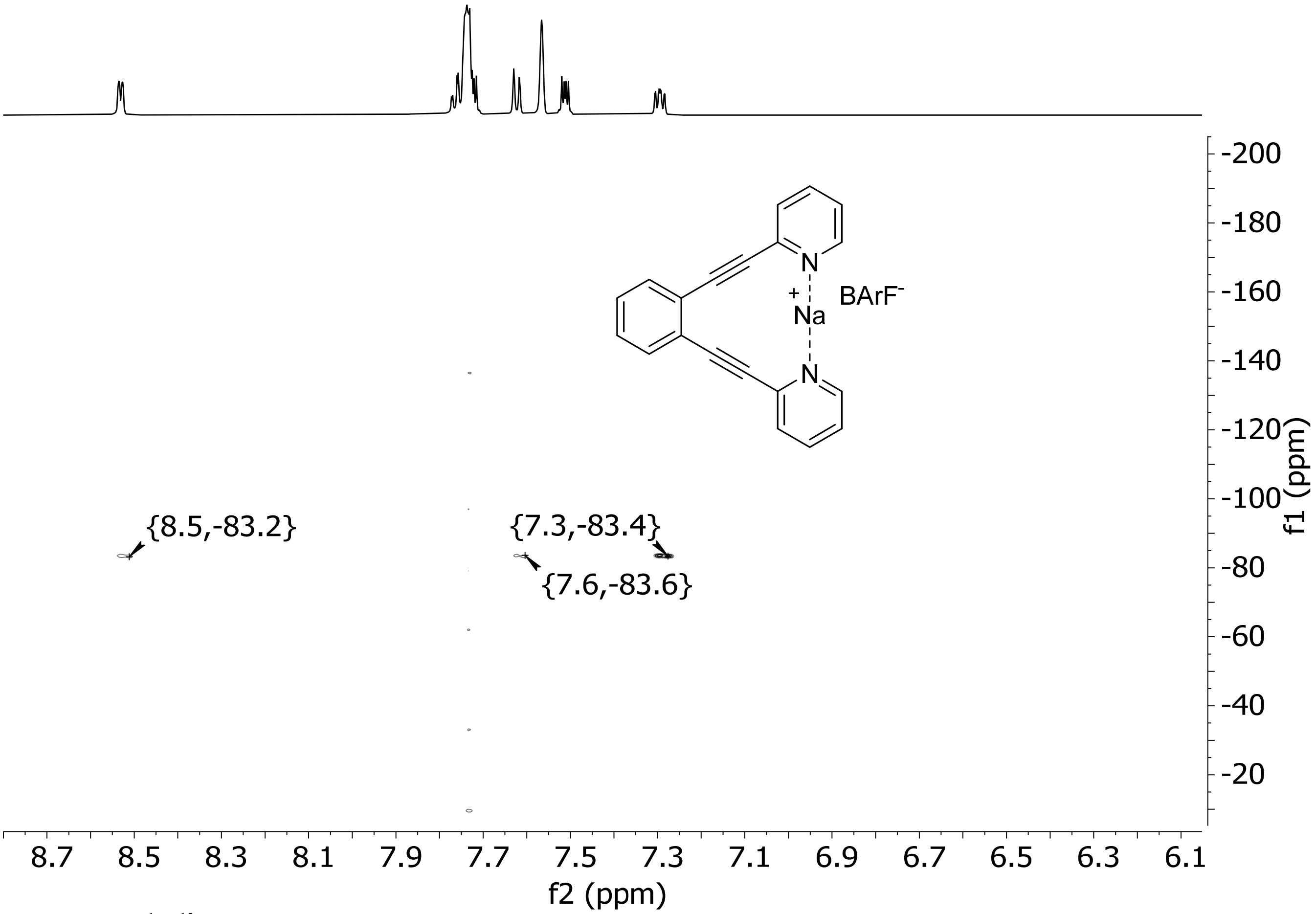

Figure S32. The ^{1}H, ^{15}N HMBC NMR spectrum of **1**-Na acquired at 25 °C in CD_2Cl_2 at 61 MHz.

6. Crystallographic Data Collected from CSD

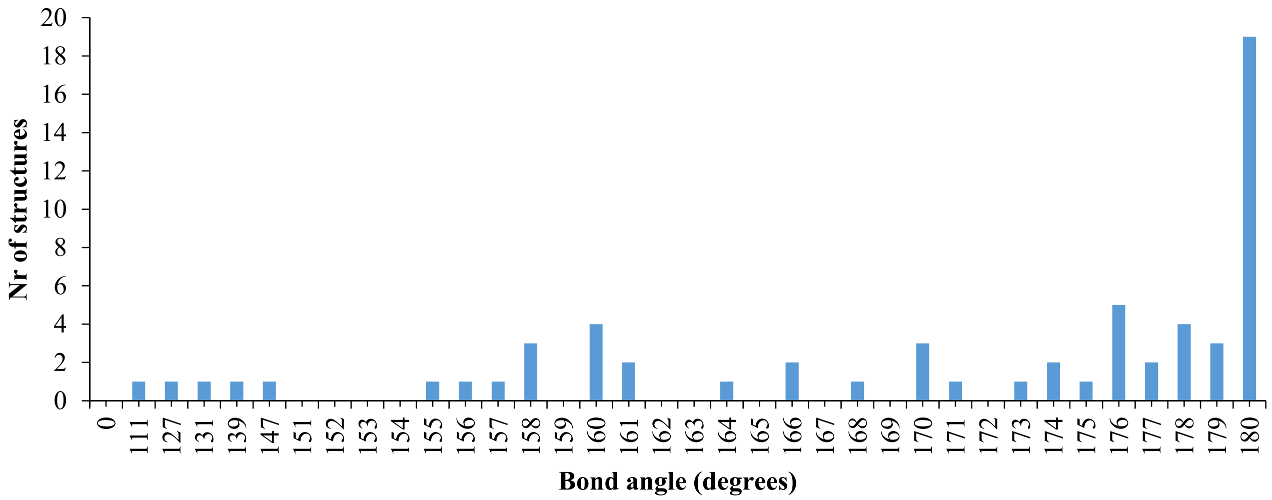

Figure S33. The number of X-ray structures as a function of their N-H-N dihedral angle, derived from the 57 X-ray structures with an N-H-N synthon available in the CSD. Out of the structures, 31% have a linear 180° N-H-N angle, whereas 55% 175-180°.

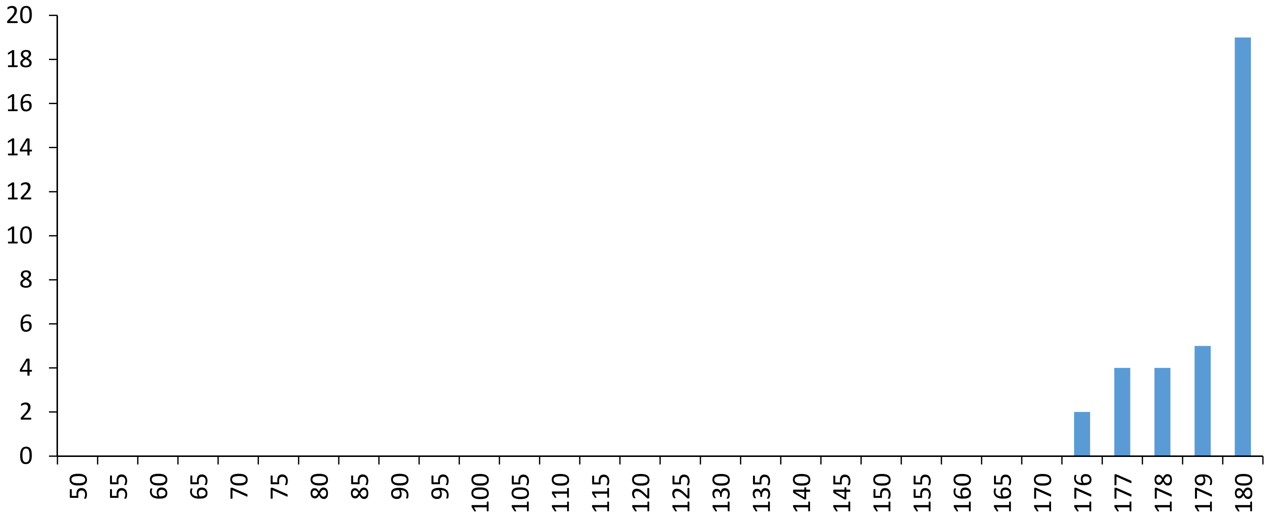

Figure S34. The number of X-ray structures as a function of their N-Br-N dihedral angle, derived from the 34 X-ray structures with an N-Br-N synthon available in the CSD. Out of the structures, 56% have a linear 180° N-Br-N angle, whereas 100% 175-180°.

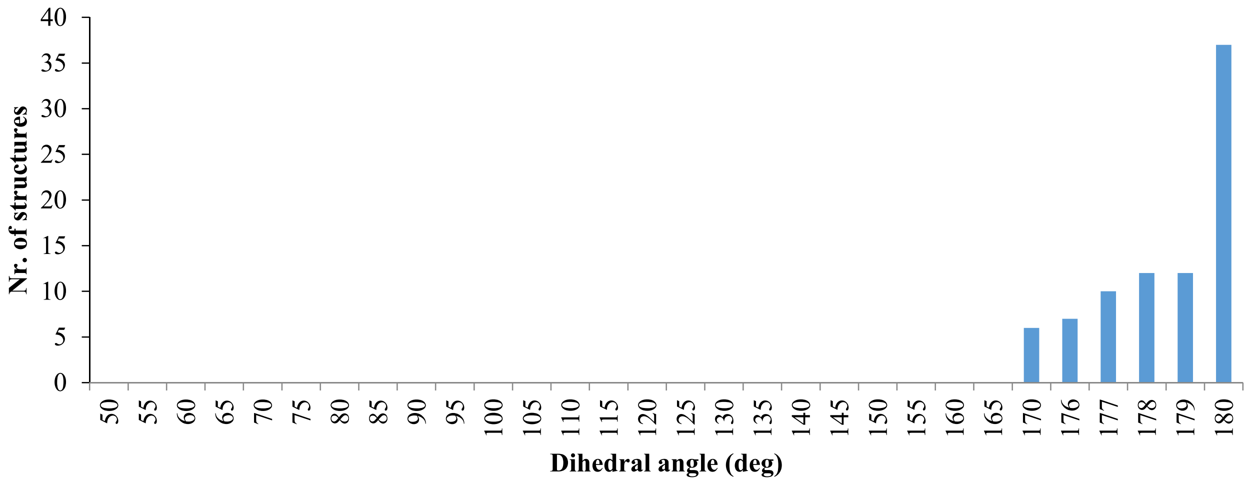

Figure S35. The number of X-ray structures as a function of their N-I-N dihedral angle, derived from the 85 X-ray structures with an N-I-N synthon available in the CSD. Out of the structures, 44% have a linear 180° N-I-N angle, whereas 99% 175-180°.

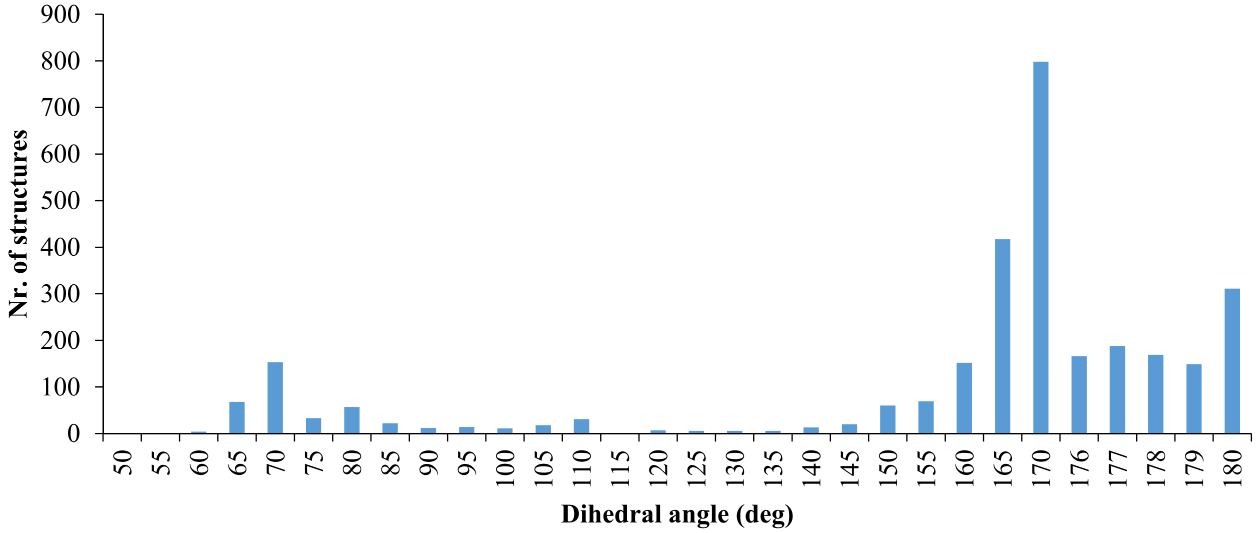

Figure S36. The number of X-ray structures as a function of their N-Ag-N dihedral angle, derived from the 2960 X-ray structures with an N-Ag-N synthon available in the CSD. Out of the structures, 11% have a linear 180° N-Ag-N angle, whereas 60% 175-180°.

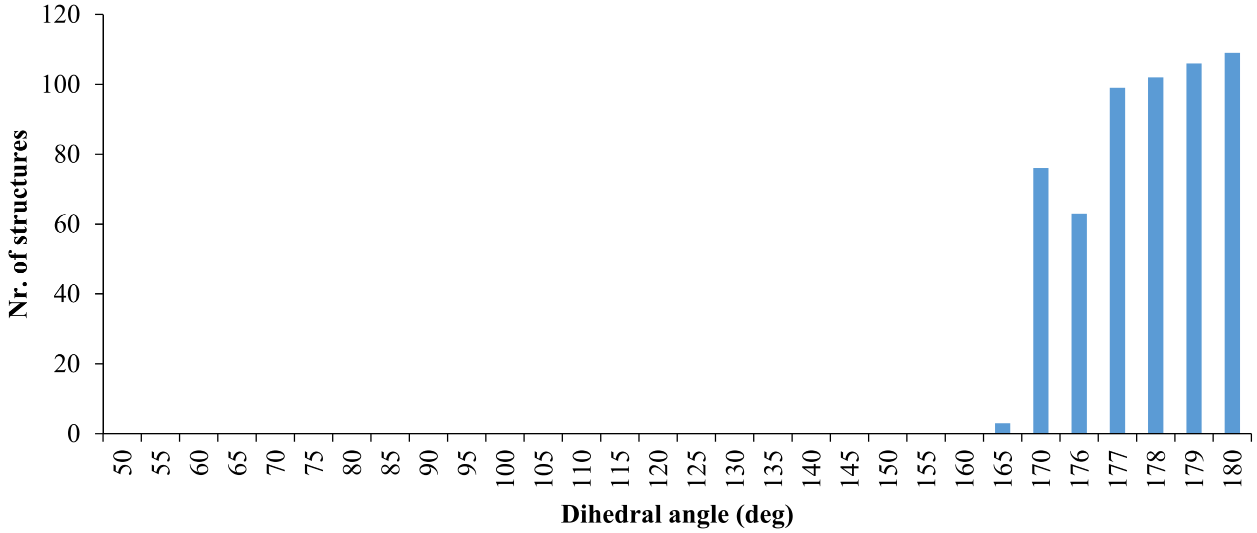

Figure S37. The number of X-ray structures as a function of their N-Au-N dihedral angle, derived from the 558 X-ray structures with an N-Au-N synthon available in the CSD. Out of the structures, 19% have a linear 180° N-Au-N angle, whereas 99% 175-180°.

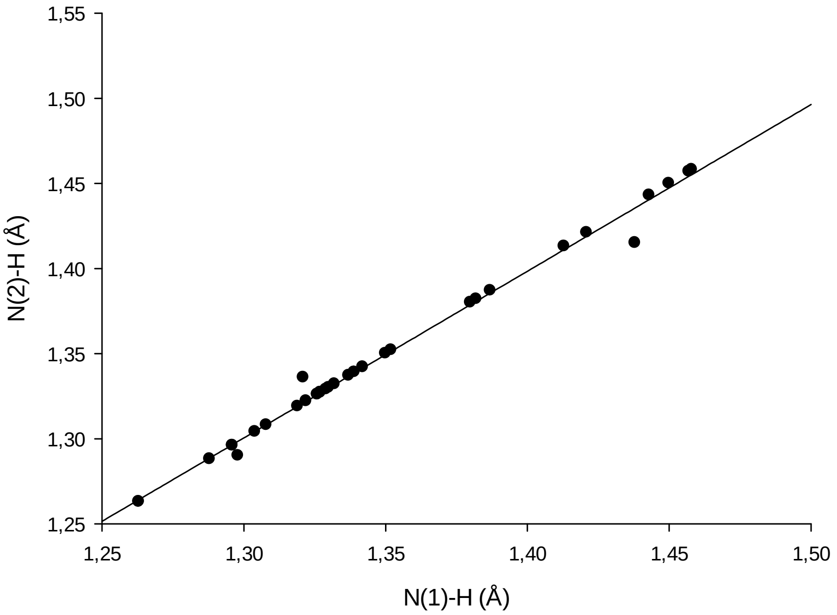

Figure S38. Scatterplot of the N-H distances within the N-H-N motif in the 34 X-ray structures available in the CSD, possessing a linear (180 ± 10°) N-H-N bond angle. The correlation has r^2 0.9923. Numerical parameters are given in Table S13.

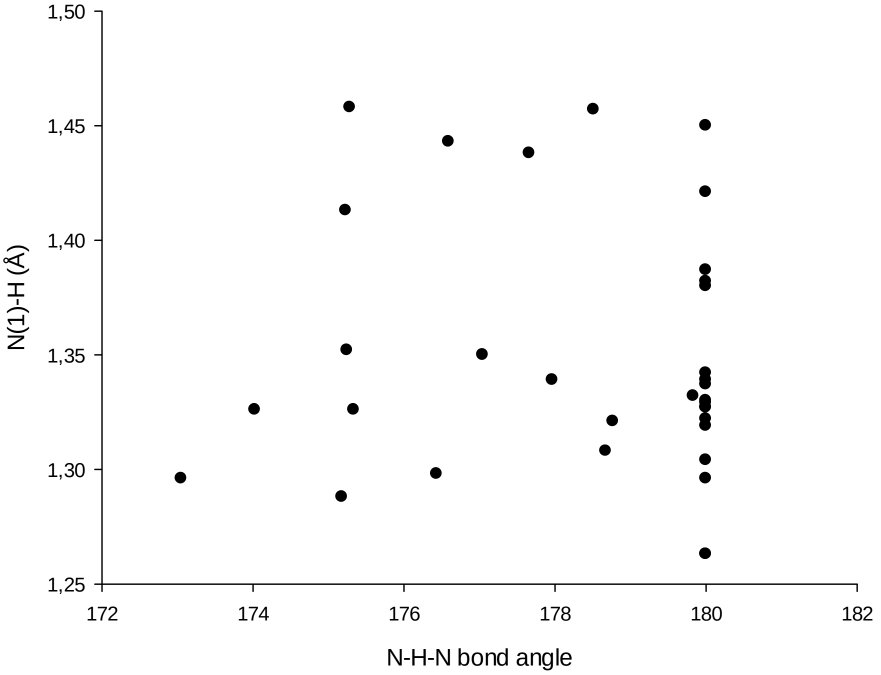

Figure S39. Scatterplot of the N-H bond distance as a function of the N-H-N bond angle within an N-H-N motif in the 34 X-ray structures available in the CSD, possessing a close to linear (180° ± 10°) N-H-N bond angle. Numerical parameters are given in Table S13.

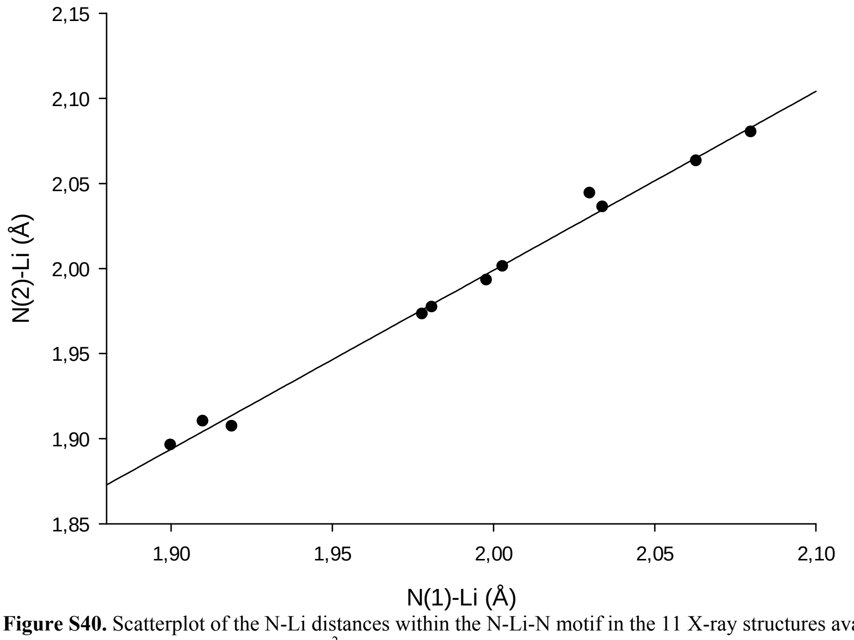

Figure S40. Scatterplot of the N-Li distances within the N-Li-N motif in the 11 X-ray structures available in the CSD, possessing a linear (180° ± 10°) N-Li-N bond angle. The correlation has r^2 0.9901. Structures containing additional cation were not considered. Numerical parameters are given in Table S14.

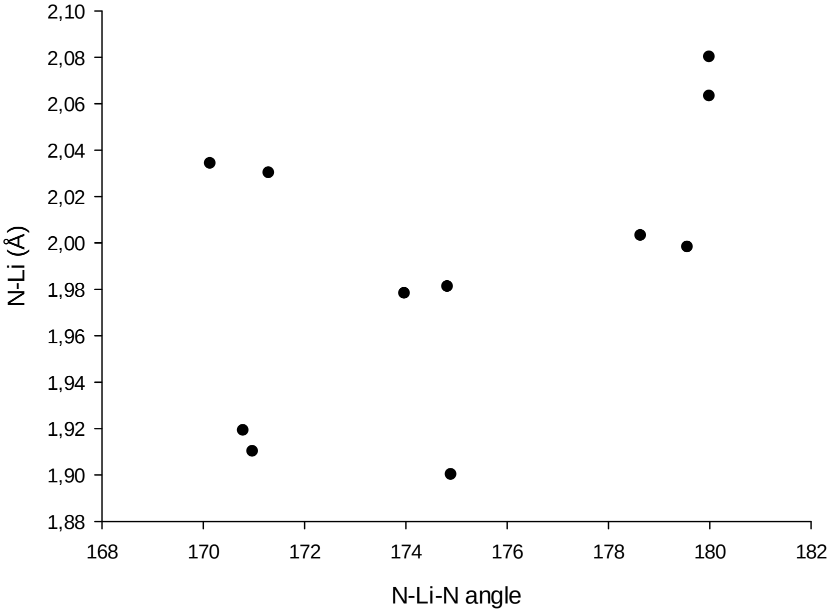

Figure S41. Scatterplot of the N-Li bond distance as a function of the N-Li-N bond angle within an N-Li-N motif in the 11 X-ray structures available in the CSD, possessing a close to linear (180° ± 10°) N-Li-N bond angle. Numerical parameters are given in Table S14.

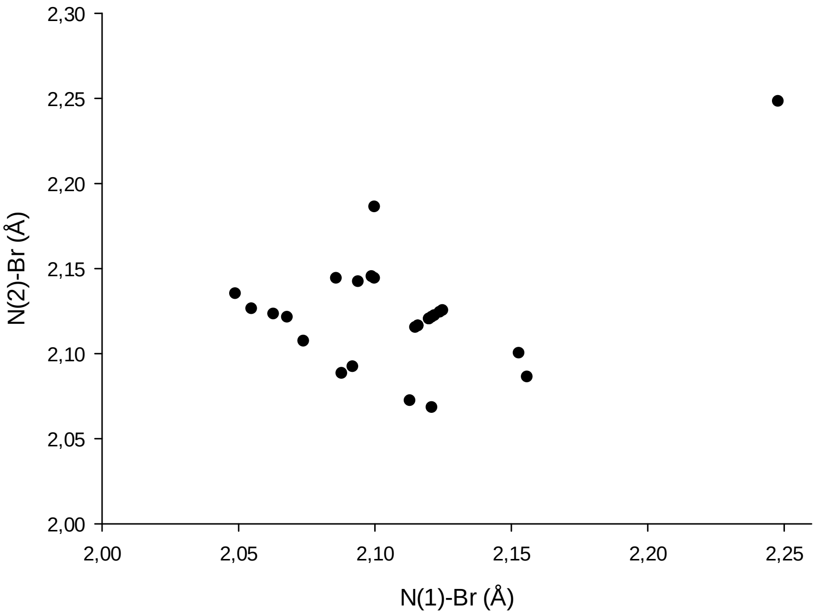

Figure S42. Scatterplot of the N-Br distances within the N-Br-N motif within [bis(pyridine)bromine(I)]$^+$ complexes in the 26 X-ray structures available in the CSD, possessing a linear (180° ± 10°) N-Br-N bond angle. The correlation has r^2 0.403. Structures containing additional cation were not considered. Numerical parameters are given in Table S15.

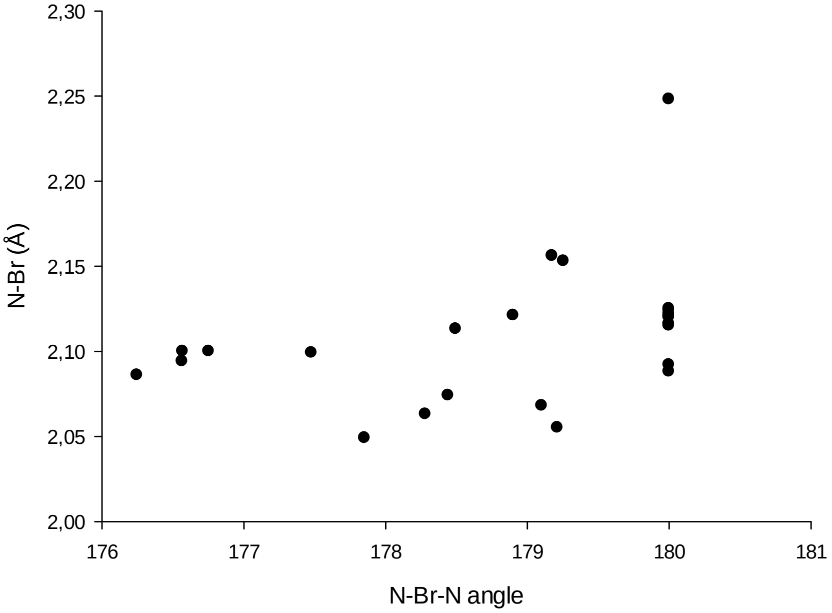

Figure S43. Scatterplot of the N-Br bond distance as a function of the N-Br-N bond angle within an N-Br-N motif in bis(pyridine) complexes, in the 26 X-ray structures available in the CSD, possessing a close to linear (180° ± 10°) N-Br-N bond angle. Numerical parameters are given in Table S15.

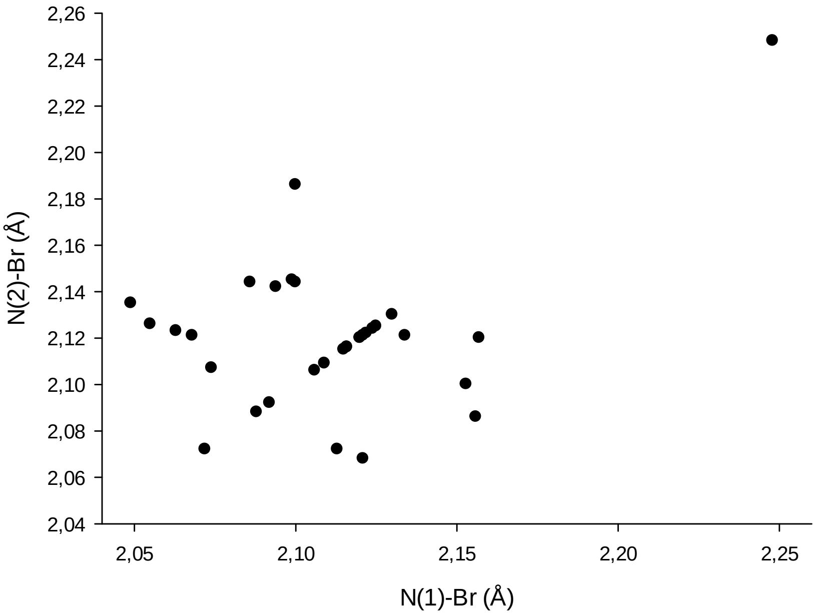

Figure S44. Scatterplot of the N-Br distances within the N-Br-N motif within in the 32 X-ray structures available in the CSD, possessing a linear (180° ± 10°) N-Br-N bond angle (with any *N*-donor heterocycles involved as Lewis base). The correlation has r^2 0.383. Structures containing additional cation were not considered. Numerical parameters are given in Table S15.The intrinsically asymmetric JUBNEZ01 and MUGDIC complexes were excluded.

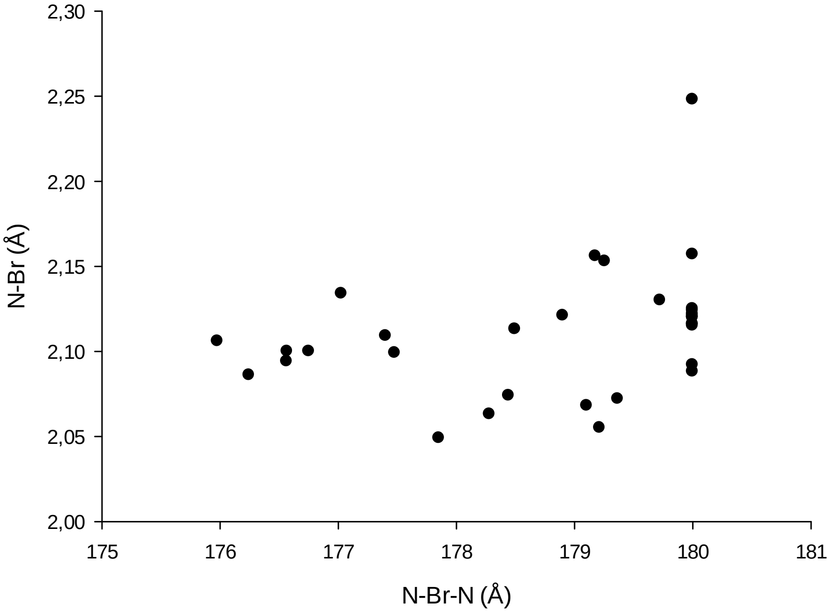

Figure S45. Scatterplot of the N-Br bond distance as a function of the N-Br-N bond angle within an N-Br-N in the 34 X-ray structures available in the CSD, possessing a close to linear (180° ± 10°) N-Br-N bond angle (with any *N*-donor heterocycles involved as Lewis base). Numerical parameters are given in Table S16. The intrinsically asymmetric JUBNEZ01 and MUGDIC complexes were excluded.

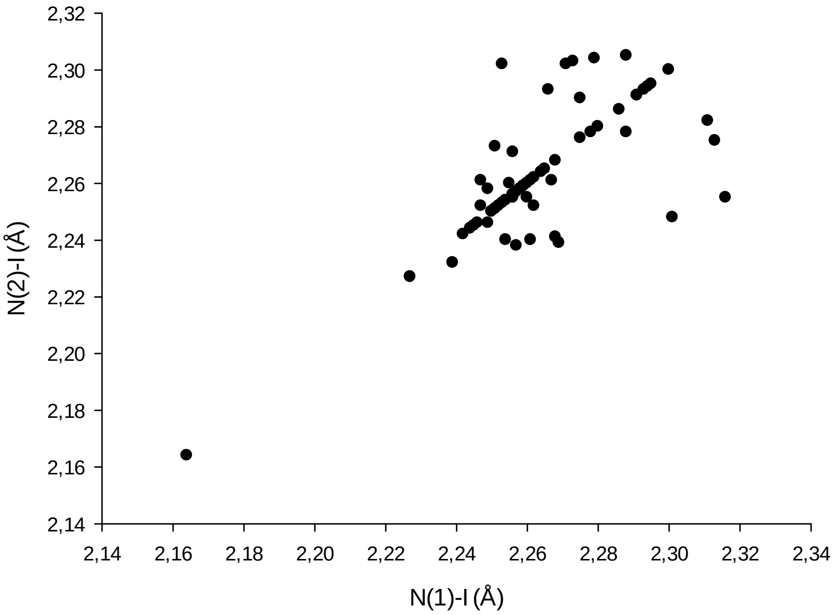

Figure S46. Scatterplot of the N-I distances within the N-I-N motif of the 70 X-ray [bis(pyridine)iodine(I)]$^+$ complexes available in the CSD, possessing a linear (180° ± 10°) N-I-N bond angle. The correlation has r^2 0.482. Structures containing additional cations were not considered. Numerical parameters are given in Table S16. The structures showing largest deviations possess nonlinear supramolecular complexes (NOMBOJ; NOMBUP; NOMCAW). Removing these 9 nonlinear structures, the r^2 increases to 0.72

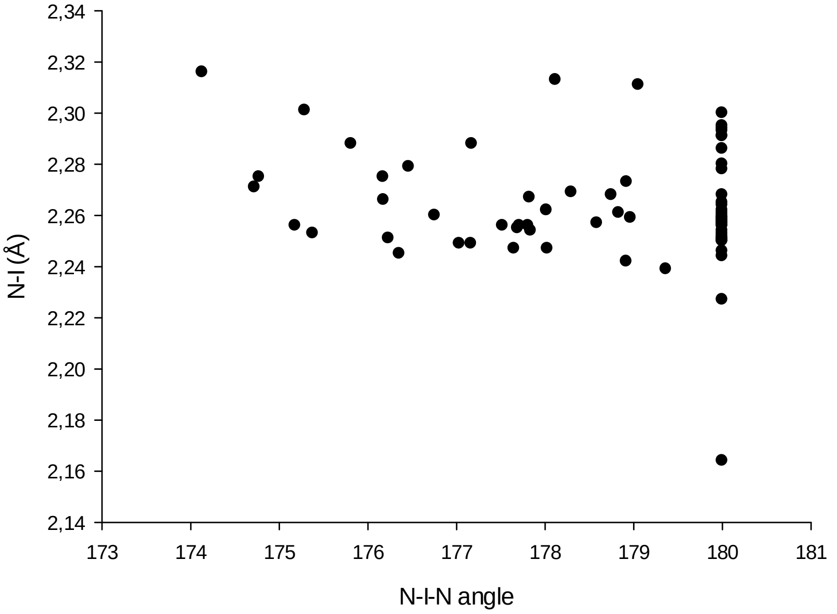

Figure S47. Scatterplot of the N-I bond distance as a function of the N-I-N bond angle within 70 X-ray derived [bis(pyridine)iodine(I)]$^+$ complexes available in the CSD, possessing a close to linear (180° ± 10°) N-I-N bond angle. Numerical parameters are given in Table S16.

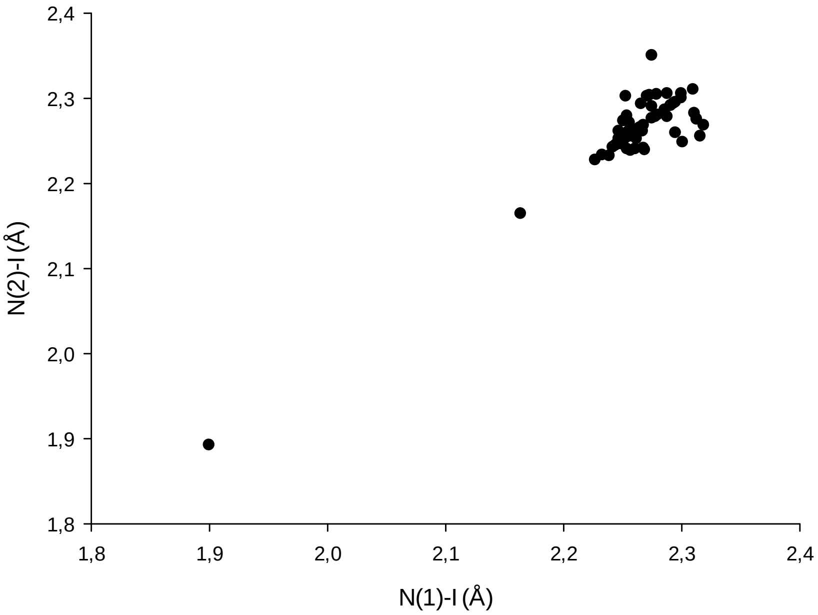

Figure S48. Scatterplot of the N-I distances within the N-I-N motif of the 80 complexes available in the CSD, possessing a linear (180° ± 10°) N-I-N bond angle (with any *N*-donor heterocycles involved as Lewis base). The correlation has r^2 0.389. Structures containing additional cations were not considered. Numerical parameters are given in Table S17. The structures showing largest deviations possess nonlinear supramolecular complexes (NOMBOJ; NOMBUP; NOMCAW).

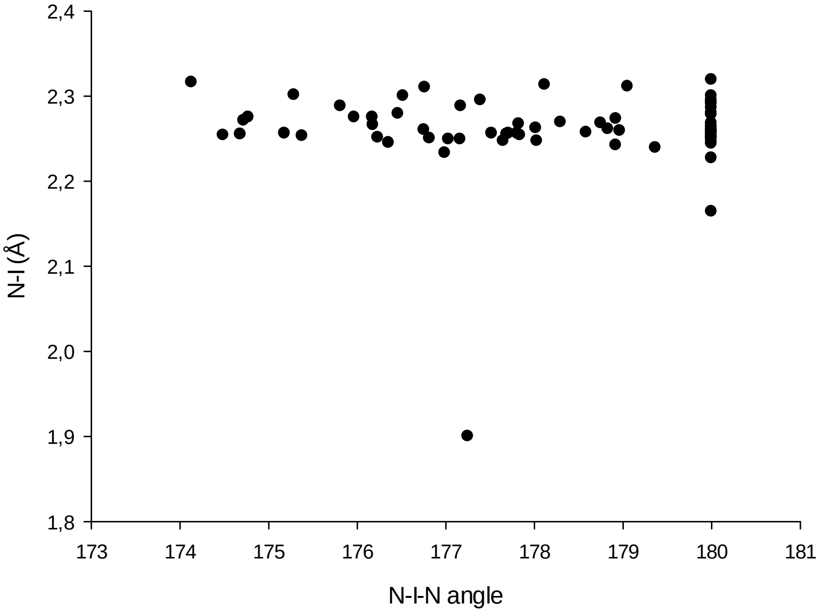

Figure S49. Scatterplot of the N-I bond distance as a function of the N-I-N bond angle within 80 related complexes available in the CSD, possessing a close to linear (180° ± 10°) N-I-N bond angle (with any *N*-donor heterocycles involved as Lewis base). Numerical parameters are given in Table S17.

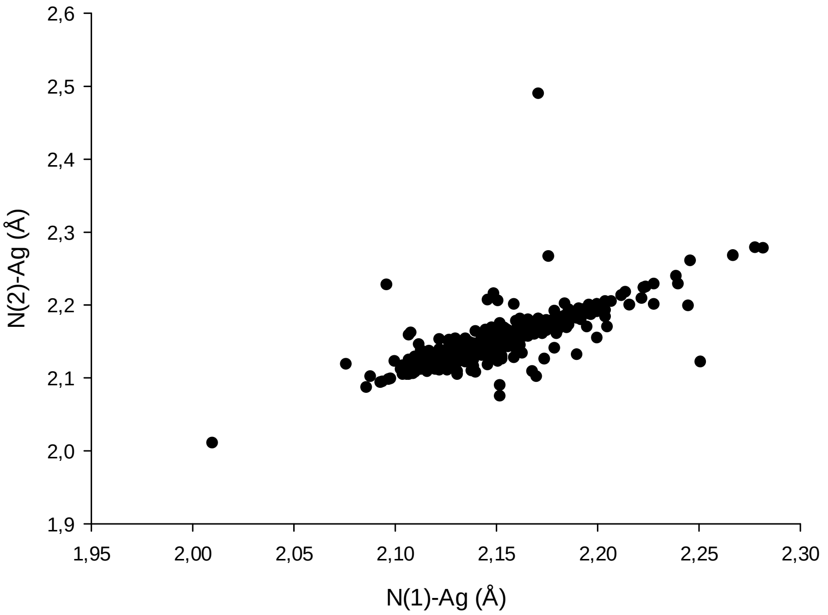

Figure S50. Scatterplot of the N-Ag distances within the N-Ag-N motif of the 537 [bis(pyridine)silver(I)]$^+$ complexes available in the CSD, possessing a linear (180° ± 10°) N-Ag-N bond angle. The correlation has r^2 0.578. Structures containing additional cations were not considered. Numerical parameters are given in Table S18. The structures showing largest deviations possess nonlinear supramolecular complexes (NEGXIJ, NEGYAC, CAHMUW).

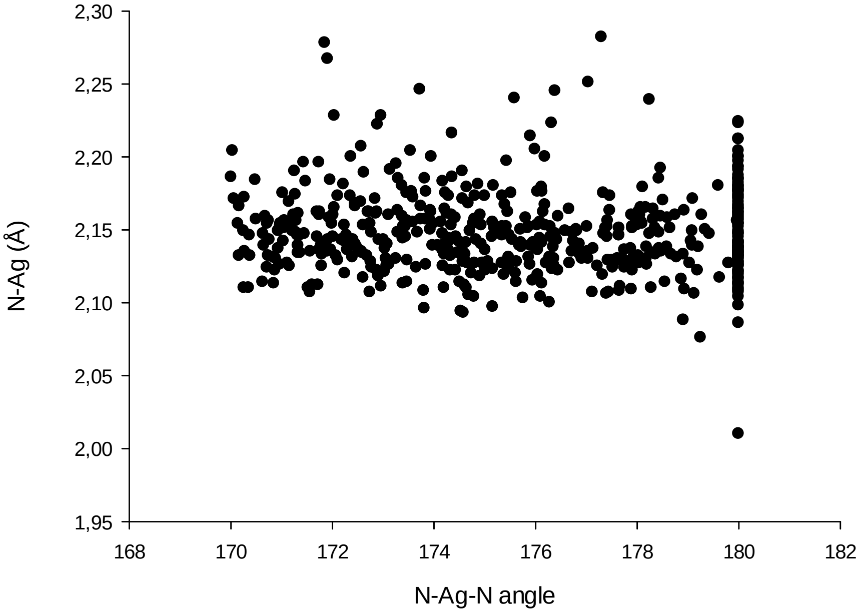

Figure S51. Scatterplot of the N-Ag bond distance as a function of the N-Ag-N bond angle within 537 related [bis(pyridine)silver(I)]$^+$ complexes available in the CSD, possessing a close to linear (180° ± 10°) N-I-N bond angle. Numerical parameters are given in Table S18.

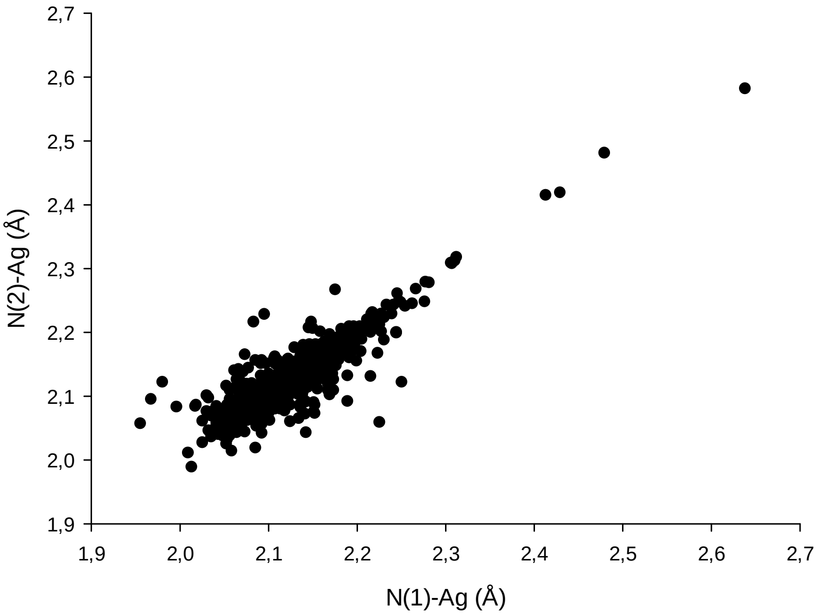

Figure S52. Scatterplot of the N-Ag distances within the N-Ag-N motif of the 1780 related complexes available in the CSD, possessing a linear (180° ± 10°) N-Ag-N bond angle (with any *N*-donor heterocycles involved as Lewis base). The correlation has r^2 0.828. Numerical parameters are given in Table S19.

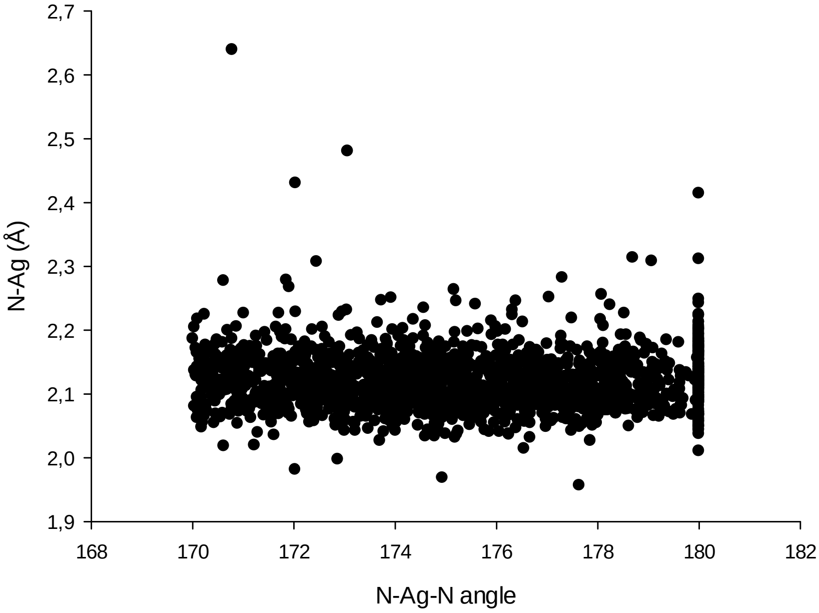

Figure S53. Scatterplot of the N-Ag bond distance as a function of the N-Ag-N bond angle within 1780 related complexes available in the CSD, possessing a close to linear (180° ± 10°) N-I-N bond angle (with any *N*-donor heterocycles involved as Lewis base). Numerical parameters are given in Table S19.

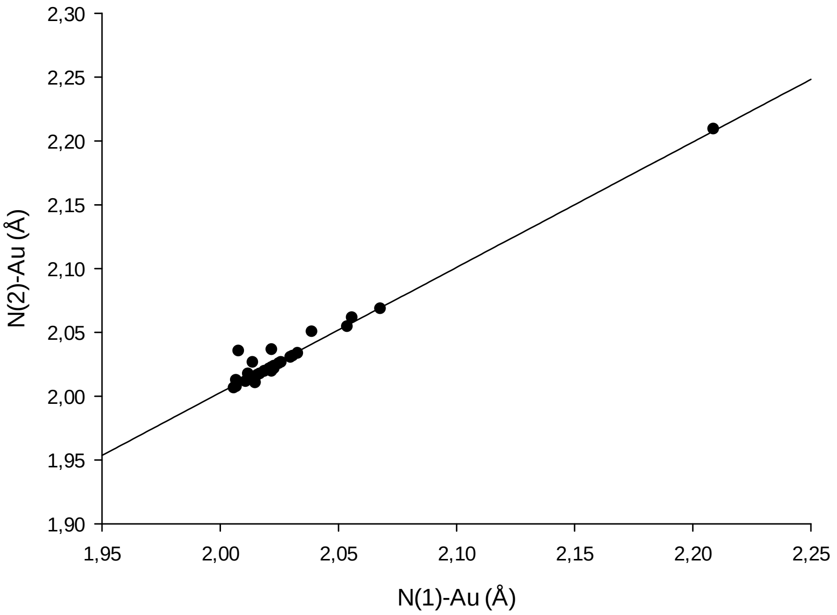

Figure S49. Scatterplot of the N-Au distances within the N-Ag-N motif of the 31 related [bis(pyridine)gold(I)]$^+$ complexes available in the CSD, possessing a linear (180° ± 10°) N-Au-N bond angle. The correlation has r^2 0.971. Numerical parameters are given in Table S20.

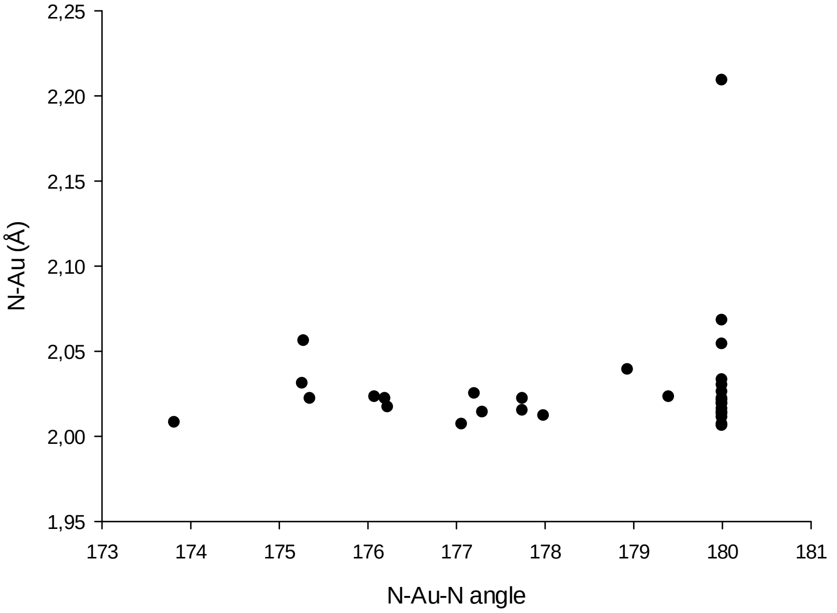

Figure S50. Scatterplot of the N-Au bond distance as a function of the N-Au-N bond angle within 31 related [bis(pyridine)gold(I)]$^+$ complexes available in the CSD, possessing a close to linear (180° ± 10°) N-I-N bond angle. Numerical parameters are given in Table S20.

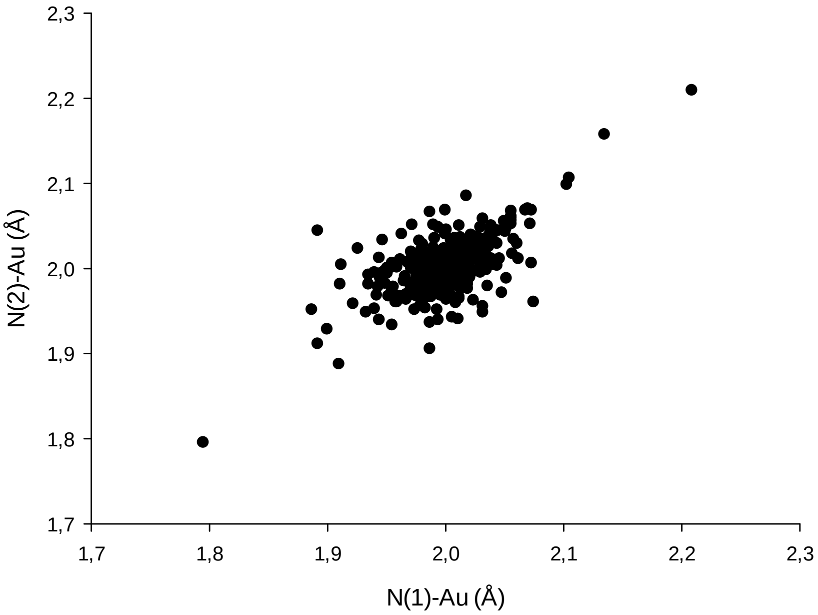

Figure S51. Scatterplot of the N-Au distances within the N-Ag-N motif of the 535 related complexes available in the CSD, possessing a linear (180° ± 10°) N-Au-N bond angle (with any *N*-donor heterocycles involved as Lewis base). The correlation has r^2 0.271. Numerical parameters are given in Table S21.

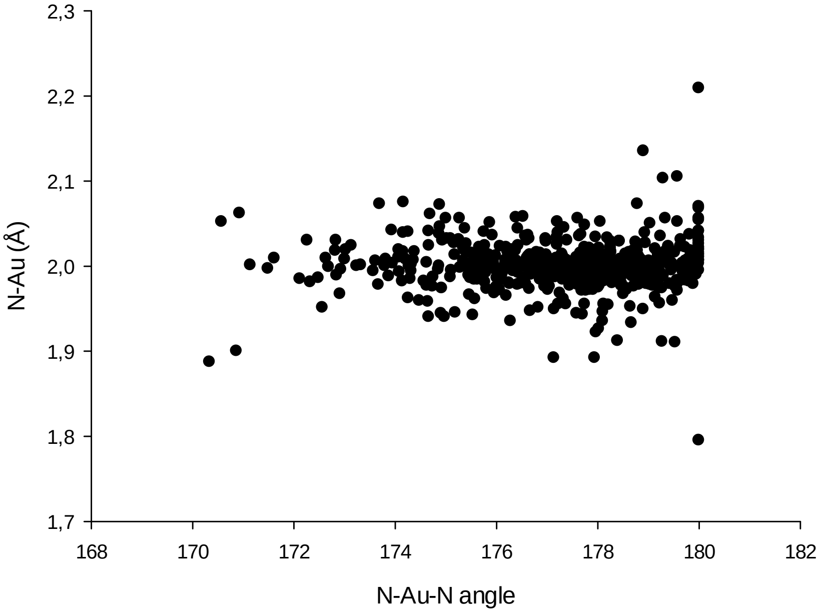

Figure S52. Scatterplot of the N-Au bond distance as a function of the N-Au-N bond angle within 535 related complexes available in the CSD, possessing a close to linear (180° ± 10°) N-I-N bond angle (with any *N*-donor heterocycles involved as Lewis base). Numerical parameters are given in Table S21.

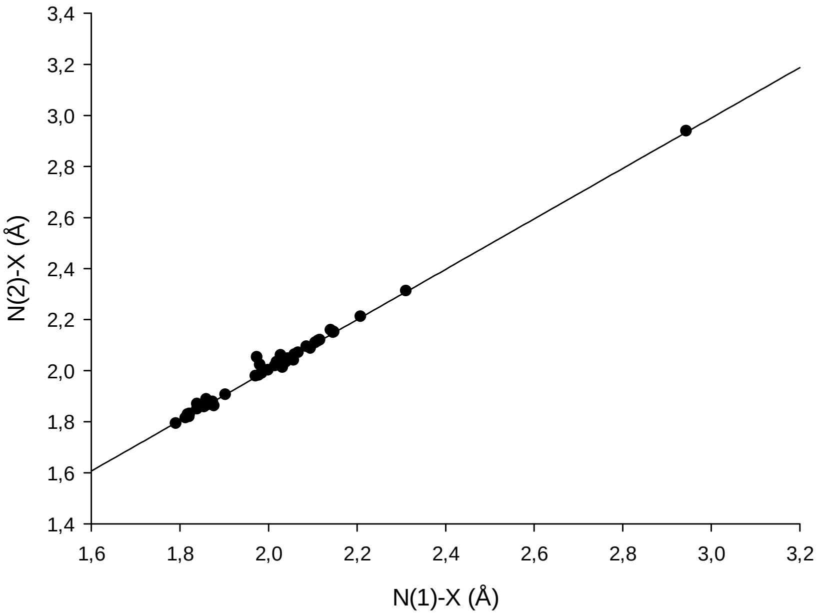

Figure S51. Scatterplot of the N-X distances within the N-X-N motif of the 54 related bis(pyridine) Hg(II), Cd(II), Te(III), Er(III), Zn(II), Gd(III), Mn(II), Fe(II), Ni(II), Cr(II), and Rh(I) complexes available in the CSD, possessing a linear (180° ± 10°) N-Au-N bond angle. The correlation has r^2 0.991. Numerical parameters are given in Table S22.

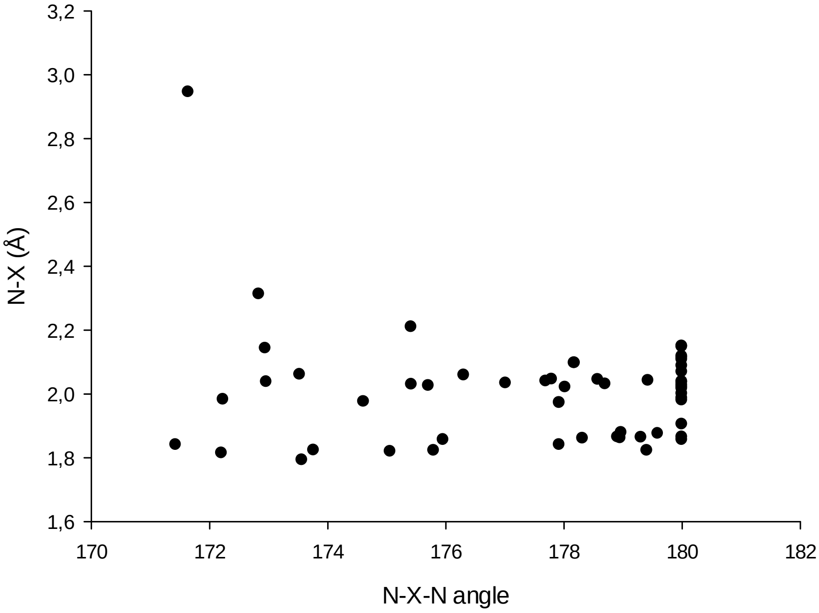

Figure S52. Scatterplot of the N-X bond distance as a function of the N-X-N bond angle within 54 related bis(pyridine)-type Hg(II), Cd(II), Te(III), Er(III), Zn(II), Gd(III), Mn(II), Fe(II), Ni(II), Cr(II), and Rh(I) complexes available in the CSD, possessing a close to linear (180° ± 10°) N-I-N bond angle. Numerical parameters are given in Table S22.

Table S13. N-H-N Bond angles and N-H bond distances in the X-ray structures available in the CSD. Only close to linear (180° ± 10°), nonpolymeric structures not having additional metal coordination are considered.

Ref. code	Angle	N(1)-H (Å)	N(2)-H (Å)	Δ(N-H)	%	R	SpGr	Temp	Pub. year
BOXYOF	178,7	1,3080	1,3080	0,0000	0,00	3,71	C2/c	90	2020
BAYBIN	180,0	1,3300	1,3300	0,0000	0,00	6,24	P-1	123	2003
BECHOG	180,0	1,3040	1,3040	0,0000	0,00	7,55	C2/c	295	1982
CABMOH	180,0	1,2630	1,2630	0,0000	0,00	3,10	P-31c	295	1983
CABMOH01	180,0	1,2630	1,2630	0,0000	0,00	3,10	P-31c	295	1984
CAFHAT01	175,2	1,3520	1,3520	0,0000	0,00	0,00	C2/c	296	2007
GOXCIF	180,0	1,3190	1,3190	0,0000	0,00	3,15	P-1	295	1998
IQIBAL01	180,0	1,3820	1,3820	0,0000	0,00	7,20	P-1	373	2004
IVONOY	180,0	1,2960	1,2960	0,0000	0,00	3,03	I41/a	100	2016
JETHAS	180,0	1,3800	1,3800	0,0000	0,00	5,75	C2/c	150	2007
JIMGOC	176,4	1,2980	1,2900	8,0000e-3	0,62	9,78	P-1	100	2007
KAHRIU	175,3	1,3260	1,3260	0,0000	0,00	6,40	P21212	295	1988
LADBIB01	178,5	1,4570	1,4570	0,0000	0,00	5,60	C2/c	295	1993
LADBIB01	176,6	1,4430	1,4430	0,0000	0,00	5,60	C2/c	295	1993
LEQHIY	180,0	1,3370	1,3370	0,0000	0,00	5,25	I2/m	295	1998
MEPHPY01	175,2	1,4130	1,4130	0,0000	0,00	8,00	C2/c	295	1975
MEPHPY01	177,7	1,4380	1,4150	0,0230	1,63	8,00	C2/c	295	1975
MEPHPY01	175,3	1,4580	1,4580	0,0000	0,00	8,00	C2/c	295	1975
NUSKIV	180,0	1,4500	1,4500	0,0000	0,00	4,07	P21/c	295	1998
ODICOU01	180,0	1,4210	1,4210	0,0000	0,00	6,46	P-1	9	2007
OWEMEJ	180,0	1,3270	1,3270	0,0000	0,00	6,14	C2/c	293	2011
QARQAC	180,0	1,3870	1,3870	0,0000	0,00	6,39	P21/c	295	2004
QUKXEZ	179,8	1,3320	1,3320	0,0000	0,00	3,49	C2/c	298	2004
RULFAF	180,0	1,3290	1,3290	0,0000	0,00	1,76	R32	295	1997
SAKKEU	173,1	1,2960	1,2960	0,0000	0,00	5,50	C2/c	295	1988
SAKKIY	175,2	1,2880	1,2880	0,0000	0,00	4,40	C2/c	295	1988
TOBGAU	174,0	1,3260	1,3260	0,0000	0,00	3,20	P21212	100	2014
WACMIY01	178,0	1,3390	1,3390	0,0000	0,00	3,40	C2/c	291	2017
WAJBEP	180,0	1,3220	1,3220	0,0000	0,00	3,55	P-1	120	2004
WEVHOX	178,8	1,3210	1,3360	-0,0150	-1,12	8,45	P-1	170	2018
WOFGII	180,0	1,3420	1,3420	0,0000	0,00	5,24	P-1	293	2008
XACFOW	180,0	1,3270	1,3270	0,0000	0,00	9,00	P-1	295	1999
XOHWAT	180,0	1,3390	1,3390	0,0000	0,00	5,04	P21/c	293	2008
YULTOO	177,0	1,3500	1,3500	0,0000	0,00	2,60	Pccn	295	1995

Table S14. N-Li-N Bond angles and N-Li bond distances in the X-ray structures available in the CSD. Only close to linear (180° ± 10°), nonpolymeric structures not having additional metal coordination are considered.

Ref. code	Angle	N(1)-Li (Å)	N(2)-Li (Å)	Δ(N-Li)	%	R	SpgGr	Temp	Pub. Year
AQIZEG	171,0	1,9100	1,9100	0,00	0,00	7,33	P41212	173	2011
EDORUK	180,0	2,0800	2,0800	0,00	0,00	4,58	P-1	293	2002
HATZAF	170,8	1,9190	1,9070	0,01	0,63	4,51	C2/c	150	2012
MIJHAR	170,1	2,0340	2,0360	-2,00e-3	-0,10	5,91	P-1	123	2018
MIJHAR	171,3	2,0300	2,0440	-0,01	-0,68	5,91	P-1	123	2018
MOLYER	174,9	1,9000	1,8960	4,00e-3	0,21	4,94	P21/n	200	2002
OYEVEU	180,0	2,0630	2,0630	0,00	0,00	8,54	P21/c	123	2011
VAJFES	179,6	1,9980	1,9930	5,00e-3	0,25	5,05	C2/c	123	2003
VAJFES	178,6	2,0030	2,0010	2,00e-3	0,10	5,05	C2/c	123	2003
ZALCUK	174,8	1,9810	1,9770	4,00e-3	0,20	3,80	C2	122	1995
ZALCUK	174,0	1,9780	1,9730	5,00e-3	0,25	3,80	C2	122	1995

Table S15. N-Br-N Bond angles and N-Br bond distances in the [bis(pyridine)bromine(I)]$^{+}$-type structures X-ray structures available in the CSD. Only close to linear (180° ± 10°), nonpolymeric structures not having additional metal coordination are considered.

Ref. code	Angle	N(1)-Br (Å)	N(2)-Br (Å)	Δ(N-Br)	%	R	SpGr	Temp	Pub. Year
AKOXON	176,2	2,0860	2,1440	-0,0580	-2,7052	6,62	C2/c	298	2003
AKOXON	180,0	2,1160	2,1160	0,0000	0,0000	6,62	C2/c	298	2003
AKOXON01	176,8	2,1000	2,1440	-0,0440	-2,0522	3,41	C2/c	300	2018
AKOXON01	180,0	2,1250	2,1250	0,0000	0,0000	3,41	C2/c	300	2018
AKOXON02	176,6	2,0940	2,1420	-0,0480	-2,2409	2,50	C2/c	100	2018
AKOXON02	180,0	2,1200	2,1200	0,0000	0,0000	2,50	C2/c	100	2018
AKOXON03	180,0	2,1160	2,1160	0,0000	0,0000	4,56	C2/c	300	2018
AKOXON03	180,0	2,1210	2,1210	0,0000	0,0000	4,56	C2/c	300	2018
AKOXON04	180,0	2,1200	2,1200	0,0000	0,0000	5,30	C2/c	100	2018
AKOXUT	178,4	2,0740	2,1070	-0,0330	-1,5662	4,90	P-1	296	2003
AKOXUT01	178,9	2,1210	2,0680	0,0530	2,5629	5,55	P-1	100	2014
AKOXUT02	178,5	2,1130	2,0720	0,0410	1,9788	3,57	P-1	300	2014
DOVZOF	178,3	2,0630	2,1230	-0,0600	-2,8262	3,91	P21/c	300	2014
DOVZOF01	177,9	2,0490	2,1350	-0,0860	-4,0281	3,14	P21/c	150	2014
DOWBAU	180,0	2,0920	2,0920	0,0000	0,0000	3,14	P21/c	300	2014
DOWBAU01	180,0	2,0880	2,0880	0,0000	0,0000	2,20	P21/c	150	2014
DOWBIC	179,1	2,0680	2,1210	-0,0530	-2,4988	3,86	C2/c	300	2014

DOWBIC01	179,2	2,0550	2,1260	-0,0710	-3,3396	2,48	C2/c	100	2014
ETOYET	180,0	2,2480	2,2480	0,0000	0,0000	8,74	P-1	295	2017
QUBRPC	177,5	2,0990	2,1450	-0,0460	-2,1445	7,50	C11a	295	1975
QUBRPC	176,6	2,1000	2,1860	-0,0860	-3,9341	7,50	C11a	295	1975
SIMTEQ	179,3	2,1530	2,1000	0,0530	2,5238	3,83	P-1	300	2018
SIMTEQ01	179,2	2,1560	2,0860	0,0700	3,3557	2,79	P-1	100	2018
SIMWET	180,0	2,1220	2,1220	0,0000	0,0000	3,45	C2/c	300	2018
SIMWET	180,0	2,1240	2,1240	0,0000	0,0000	3,45	C2/c	300	2018
SIMWET01	180,0	2,1150	2,1150	0,0000	0,0000	4,31	C2/c	100	2018

Table S16. N-I-N Bond angles and N-I bond distances in the 70 [bis(pyridine)iodine(I)]$^+$ X-ray structures available in the CSD. Only close to linear (180° ± 10°), nonpolymeric structures not having additional metal coordination are considered.

Ref. code	Angle	N(1)-I (Å)	N(2)-I (Å)	Δ(N-I)	%	R	SpGr	Temp	Pub. Year
AKOXON	176,2	2,0860	2,1440	-0,0580	-2,71	6,6200	C2/c	298	2003
AKOXON	180,0	2,1160	2,1160	0,0000	0,00	6,6200	C2/c	298	2003
AKOXON01	176,8	2,1000	2,1440	-0,0440	-2,05	3,4100	C2/c	300	2018
AKOXON01	180,0	2,1250	2,1250	0,0000	0,00	3,4100	C2/c	300	2018
AKOXON02	176,6	2,0940	2,1420	-0,0480	-2,24	2,5000	C2/c	100	2018
AKOXON02	180,0	2,1200	2,1200	0,0000	0,00	2,5000	C2/c	100	2018
AKOXON03	180,0	2,1160	2,1160	0,0000	0,00	4,5600	C2/c	300	2018
AKOXON03	180,0	2,1210	2,1210	0,0000	0,00	4,5600	C2/c	300	2018
AKOXON04	180,0	2,1200	2,1200	0,0000	0,00	5,3000	C2/c	100	2018
AKOXUT	178,4	2,0740	2,1070	-0,0330	-1,57	4,9000	P-1	296	2003
AKOXUT01	178,9	2,1210	2,0680	0,0530	2,56	5,5500	P-1	100	2014
AKOXUT02	178,5	2,1130	2,0720	0,0410	1,98	3,5700	P-1	300	2014
BUWMOV	180,0	2,1570	2,1200	0,0370	1,75	2,4000	P213	295	1983
CIGBIG	179,7	2,1300	2,1300	0,0000	0,00	2,6700	P3221	150	2018
DOVZOF	178,3	2,0630	2,1230	-0,0600	-2,83	3,9100	P21/c	300	2014
DOVZOF01	177,9	2,0490	2,1350	-0,0860	-4,03	3,1400	P21/c	150	2014
DOWBAU	180,0	2,0920	2,0920	0,0000	0,00	3,1400	P21/c	300	2014
DOWBAU01	180,0	2,0880	2,0880	0,0000	0,00	2,2000	P21/c	150	2014
DOWBIC	179,1	2,0680	2,1210	-0,0530	-2,50	3,8600	C2/c	300	2014
DOWBIC01	179,2	2,0550	2,1260	-0,0710	-3,34	2,4800	C2/c	100	2014
EDANAA	177,0	2,1340	2,1210	0,0130	0,61	7,1300	P21/c	100	2012
ETOYET	180,0	2,2480	2,2480	0,0000	0,00	8,7400	P-1	295	2017
JEJZED	177,4	2,1090	2,1090	0,0000	0,00	4,5000	Pnma	173	1990
JUBNEZ	176,0	2,1060	2,1060	0,0000	0,00	6,3000	Pccn	295	1992
QUBRPC	177,5	2,0990	2,1450	-0,0460	-2,14	7,5000	C11a	295	1975
QUBRPC	176,6	2,1000	2,1860	-0,0860	-3,93	7,5000	C11a	295	1975
SIMTEQ	179,3	2,1530	2,1000	0,0530	2,52	3,8300	P-1	300	2018
SIMTEQ01	179,2	2,1560	2,0860	0,0700	3,36	2,7900	P-1	100	2018
SIMWET	180,0	2,1220	2,1220	0,0000	0,00	3,4500	C2/c	300	2018
SIMWET	180,0	2,1240	2,1240	0,0000	0,00	3,4500	C2/c	300	2018
SIMWET01	180,0	2,1150	2,1150	0,0000	0,00	4,3100	C2/c	100	2018
XITPEY	179,4	2,0720	2,0720	0,0000	0,00	11,9400	C2/c	100	2018

Table S17. N-I-N Bond angles and N-I bond distances in the 80 related X-ray structures available in the CSD. Only close to linear (180° ± 10°), nonpolymeric structures not having additional metal coordination are considered.

Ref. code	Angle	N(1)-I (Å)	N(2)-I (Å)	Δ(N-I)	%	R	SpGr	Temp	Pub. Year
BAZNAR	177,1730	2,2880	2,3050			5,7000	C2/c	295	1982
BAZNAR	180,0000	2,2930	2,2930			5,7000	C2/c	295	1982
BAZNAR01	176,4620	2,2790	2,3040			2,9200	C2/c	300	2018
BAZNAR01	180,0000	2,2950	2,2950			2,9200	C2/c	300	2018
BAZNAR02	176,1780	2,2660	2,2930			3,7700	C2/c	100	2018
BAZNAR02	180,0000	2,2860	2,2860			3,7700	C2/c	100	2018
BAZNAR03	180,0000	2,2910	2,2910			3,4800	C2/c	300	2018
BAZNAR03	180,0000	2,2910	2,2910			3,4800	C2/c	300	2018
BAZNAR04	180,0000	2,2780	2,2780			3,5400	C2/c	100	2018
BAZNAR04	180,0000	2,2800	2,2800			3,5400	C2/c	100	2018
CICQIQ	178,9660	2,2590	2,2590			3,6200	C2	240	2013
CICQIQ01	180,0000	2,2590	2,2590			4,2100	C2/m	240	2014
CICQIQ02	180,0000	2,2540	2,2540			6,3600	C2/m	100	2014
CICQIQ03	180,0000	2,2680	2,2680			1,7200	C2/m	170	2015
CICQOW	178,9210	2,2730	2,3030			4,5000	P-1	240	2013
CICQOW01	179,0540	2,3110	2,2820			3,8100	P-1	300	2018
CICQOW02	178,1180	2,3130	2,2750			3,2300	P-1	100	2018
DEFXIW	180,0000	2,2940	2,2940			3,1000	P-1	120	2006
DIQWIM	176,8170	2,2500	2,2470			3,8300	P21/c	100	2018
DOVYOE	180,0000	2,2500	2,2500			7,8800	C2/n	300	2014
DOVYOE01	177,7100	2,2560	2,2560			2,7800	P21/n	170	2015
DOVYOE01	180,0000	2,2570	2,2570			2,7800	P21/n	170	2015
DOVYOE01	180,0000	2,2600	2,2600			2,7800	P21/n	170	2015
DOVYOE02	180,0000	2,2510	2,2510			7,6700	C2/c	250	2014
DOVYUK	180,0000	2,2500	2,2500			2,5700	P21/n	30	2014

DOVYUK	177,5210	2,2560	2,2550	2,5700	P21/n	30	2014
DOVYUK	180,0000	2,2620	2,2620	2,5700	P21/n	30	2014
DOVZAR	178,8320	2,2610	2,2400	4,7600	P-1	300	2014
DOVZAR01	178,5860	2,2570	2,2380	4,0600	P-1	100	2014
DOVZAR02	178,0270	2,2470	2,2610	5,0200	P-1	123	2015
GANXEZ	180,0000	2,3000	2,3000	1,5800	P-1	120	2005
GEXGOF	180,0000	2,3190	2,2680	2,9000	P213	295	1988
HMTITI	176,5170	2,3000	2,3050	3,9000	P21	295	1975
HUMMAD	177,6900	2,2550	2,2600	3,5900	P21/c	120	2002
HUMMAD	180,0000	2,2590	2,2590	3,5900	P21/c	120	2002
HUMMAD	180,0000	2,2610	2,2610	3,5900	P21/c	120	2002
HUMMAD01	180,0000	2,2460	2,2460	4,0400	P21/n	150	2010
HUMMAD01	180,0000	2,2560	2,2560	4,0400	P21/n	150	2010
HUMMAD01	177,8240	2,2670	2,2610	4,0400	P21/n	150	2010
HUMMAD02	180,0000	2,2570	2,2570	7,3000	C2/n	250	2010
HUMMAD03	180,0000	2,2640	2,2640	13,7400	C2/n	300	2014
HUMMAD04	177,8080	2,2560	2,2550	5,2600	P21/n	120	2014
HUMMAD04	180,0000	2,2560	2,2560	5,2600	P21/n	120	2014
HUMMAD04	180,0000	2,2580	2,2580	5,2600	P21/n	120	2014
HUMMAD05	180,0000	2,2580	2,2580	3,8600	C2/c	210	2014
LUKZOI	180,0000	2,2520	2,2520	2,1800	P-1	173	2015
LULBIF	178,7490	2,2680	2,2410	2,9700	P21/n	123	2015
LULCAY	180,0000	2,2500	2,2500	3,3200	P-1	173	2015
LULCAY	180,0000	2,2650	2,2650	3,3200	P-1	173	2015
MIYJIP	179,3650	2,2390	2,2320	6,8900	P-1	173	2013
MIYJIP	177,6500	2,2470	2,2520	6,8900	P-1	173	2013
MUGDAU	174,4890	2,2540	2,2790	8,7900	P21/a	293	2009
NOMBOJ	176,7550	2,2600	2,2550	3,2500	P65	150	2019
NOMBOJ	175,3790	2,2530	2,3020	3,2500	P65	150	2019
NOMBUP	174,7200	2,2710	2,3020	6,0100	P-1	150	2019
NOMBUP	176,1730	2,2750	2,2760	6,0100	P-1	150	2019
NOMBUP	174,7720	2,2750	2,2900	6,0100	P-1	150	2019
NOMBUP	175,8120	2,2880	2,2780	6,0100	P-1	150	2019
NOMBUP	174,1310	2,3160	2,2550	6,0100	P-1	150	2019
NOMCAW	175,2880	2,3010	2,2480	3,3800	P61	100	2019
NOMCAW	177,0320	2,2490	2,2580	3,3800	P61	100	2019
OVANAC	178,0180	2,2620	2,2520	2,9200	P21/c	123	2016
OVANEG	176,2320	2,2510	2,2730	2,5000	Pna21	123	2016
OVANEG	175,1790	2,2560	2,2710	2,5000	Pna21	123	2016
PYRIDI	180,0000	2,1640	2,1640	7,0000	P21/c	295	1961
PYRIDI01	180,0000	2,2510	2,2510	2,6000	P21/n	100	2014
SOPWUR	177,2500	1,9000	1,8920	11,0100	P-1	293	2015
WEHSUA	175,9670	2,2750	2,3500	11,7800	Pnma	123	2017
WEHSUA	177,3930	2,2950	2,2590	11,7800	Pnma	123	2017
WEHSUA	174,6820	2,2550	2,2720	11,7800	Pnma	123	2017
WEHSUA	176,7630	2,3100	2,3100	11,7800	Pnma	123	2017
WEHTEL	176,9880	2,2330	2,2330	4,4300	P-62c	120	2017
YOFKEM	178,2970	2,2690	2,2390	4,6700	P21/c	123	2019
YOFKIQ	177,1650	2,2490	2,2460	2,7000	I41/a	123	2019
YOFKIQ	180,0000	2,2530	2,2530	2,7000	I41/a	123	2019
YOFKIQ	177,8380	2,2540	2,2400	2,7000	I41/a	123	2019
YOFKOW	180,0000	2,2270	2,2270	5,4800	C2/m	123	2019
YOFKOW	178,9190	2,2420	2,2420	5,4800	C2/m	123	2019
YOFKOW01	176,3550	2,2450	2,2450	5,6500	Fddd	123	2019
YOFLAJ	180,0000	2,2440	2,2440	2,4600	P21/c	123	2019

Table S18. N-Ag-N Bond angles and N-Ag bond distances in the 537 related X-ray structures available in the CSD for [bis(pyridine)silver(I)]$^+$ complexes. Only close to linear (180° ± 10°), nonpolymeric structures not having additional metal coordination are considered.

Ref. code	Angle	N(1)-I (Å)	N(2)-I (Å)	Δ(N-I)	%	R	SpGr	Temp	Pub. Year
KOSTIY	178,3	2,1100	2,1120	-2,0000e-3	-0,09	6,9700	P21/n	115	2019
KOSTIY	178,9	2,1160	2,1080	8,0000e-3	0,38	6,9700	P21/n	115	2019
ROWCAK	175,5	2,1310	2,1040	0,0270	1,28	8,6900	P-1	250	2019
ROYSAC	173,8	2,1080	2,1610	-0,0530	-2,45	15,1100	I432	93	2019
VUCSUK	177,9	2,1520	2,1520	0,0000	0,00	4,5900	C2/c	150	2020
ABEMUR	171,8	2,1390	2,1370	2,0000e-3	0,09	6,5200	P-1	293	2011
ACAKUN	173,5	2,1750	2,1760	-1,0000e-3	-0,05	6,6000	P21/n	301	2017
ACALEY	174,9	2,1810	2,1760	5,0000e-3	0,23	6,4100	P21/n	301	2017
ACUREV	173,9	2,1560	2,1550	1,0000e-3	0,05	2,6200	P-1	293	2001
AGPYNO	172,7	2,1620	2,1620	0,0000	0,00	11,8000	C2/c	295	1970
AGPYNO02	173,9	2,1760	2,1760	0,0000	0,00	1,7900	I2/a	173	2015
AHIDIE	180,0	2,1730	2,1730	0,0000	0,00	4,8100	C2/c	223	2002
AHIDIE	173,1	2,1910	2,1940	-3,0000e-3	-0,14	4,8100	C2/c	223	2002
AJUKOH	180,0	2,1800	2,1800	0,0000	0,00	3,8900	P-1	298	2014
AKUCEO	180,0	2,1930	2,1930	0,0000	0,00	5,0000	C2/c	193	2003
AMOVIJ	180,0	2,1480	2,1480	0,0000	0,00	5,3600	Pbam	296	2016
AQAVOD	173,4	2,1450	2,1510	-6,0000e-3	-0,28	2,8200	P21/m	293	2004
ATEHOY	180,0	2,1490	2,1470	2,0000e-3	0,09	7,2400	C2/c	293	2016

ATEHOY	180,0	2,1850	2,1730	0,0120	0,55	7,2400	C2/c	293	2016
ATOLEA	173,5	2,1140	2,1180	-4,0000e-3	-0,19	3,3400	P21/n	293	2003
AYOGUQ	180,0	2,1250	2,1250	0,0000	0,00	2,9400	P-1	293	2004
AYOGUQ	180,0	2,1320	2,1320	0,0000	0,00	2,9400	P-1	293	2004
AYOHAX	180,0	2,1340	2,1340	0,0000	0,00	3,0500	P21/c	293	2004
BALHIH	180,0	2,1100	2,1100	0,0000	0,00	3,9200	C2/c	173	2011
BALJAB	180,0	2,1380	2,1380	0,0000	0,00	3,2200	C2/c	173	2011
BEWWUW	171,1	2,1520	2,1740	-0,0220	-1,01	4,6900	R-3	293	2004
BISTUU	171,8	2,1620	2,1680	-6,0000e-3	-0,28	3,4700	P-1	150	2014
BIXHAU	180,0	2,1410	2,1410	0,0000	0,00	12,9200	P-1	93	2018
BOCFIL	173,3	2,1300	2,1530	-0,0230	-1,07	6,6100	C2/c	100	2019
BOQVEK	180,0	2,1770	2,1770	0,0000	0,00	12,7700	P21/c	173	2014
BURVER	170,8	2,1310	2,1270	4,0000e-3	0,19	2,8000	C2/c	100	2015
BUTGAZ	177,8	2,1240	2,1340	-0,0100	-0,47	5,0800	P21/c	273	2009
CABMUO	180,0	2,1740	2,1740	0,0000	0,00	6,9100	P-1	293	2001
CAGWUF	178,3	2,1570	2,1570	0,0000	0,00	11,1900	Pnma	298	2017
CAGWUF	171,3	2,1460	2,1460	0,0000	0,00	11,1900	Pnma	298	2017
CAGWUF	175,8	2,1510	2,1330	0,0180	0,84	11,1900	Pnma	298	2017
CAGWUF	173,0	2,1370	2,1370	0,0000	0,00	11,1900	Pnma	298	2017
CAGXAM	179,1	2,1060	2,1060	0,0000	0,00	9,7000	Pnna	298	2017
CAHMUW	176,4	2,2450	2,1980	0,0470	2,14	12,7300	Pbcn	100	2016
CAHMUW	177,0	2,2510	2,1210	0,1300	6,13	12,7300	Pbcn	100	2016
CALHUT	175,1	2,1280	2,1240	4,0000e-3	0,19	5,5100	P-1	173	2002
CALJAB	172,7	2,1320	2,1300	2,0000e-3	0,09	4,5800	P-1	173	2002
CALJEF	177,8	2,1360	2,1350	1,0000e-3	0,05	6,0200	P-1	173	2002
CANTOB	176,9	2,1390	2,1390	0,0000	0,00	5,5300	C2221	293	2004
CANTOB	173,6	2,1760	2,1690	7,0000e-3	0,32	5,5300	C2221	293	2004
CASXAW	180,0	2,1540	2,1540	0,0000	0,00	4,9100	P-1	293	2005
CETJER	175,7	2,1500	2,1390	0,0110	0,51	4,4400	P212121	293	2005
CETJER	172,9	2,1180	2,1190	-1,0000e-3	-0,05	4,4400	P212121	293	2005
CEZPOO	175,9	2,1310	2,1330	-2,0000e-3	-0,09	6,3900	P-1	295	2013
CEZPUU	172,6	2,1530	2,1590	-6,0000e-3	-0,28	6,3700	C2/c	295	2013
CONQEC	178,5	2,1590	2,2000	-0,0410	-1,86	7,0800	P1	293	2009
CONQIG	177,7	2,1510	2,2050	-0,0540	-2,45	4,7000	P1	293	2009
CUTNEL	176,1	2,1410	2,1340	7,0000e-3	0,33	2,7400	P-1	296	2010
CUWNIS	178,9	2,1330	2,1400	-7,0000e-3	-0,33	5,8000	P21/c	223	2010
CUWNIS	173,4	2,1440	2,1440	0,0000	0,00	5,8000	P21/c	223	2010
CUWNOY	174,7	2,1790	2,1910	-0,0120	-0,55	4,2000	P-1	223	2010
CUXRES	174,4	2,1220	2,1220	0,0000	0,00	3,3100	C2/c	295	2000
CUXRES01	173,4	2,1130	2,1370	-0,0240	-1,12	5,4600	C2/c	173	2006
CUXRES01	173,1	2,1250	2,1250	0,0000	0,00	5,4600	C2/c	173	2006
CUYXIE	176,8	2,1500	2,1670	-0,0170	-0,78	3,6600	P21/c	298	2010
DACKAV	176,3	2,1230	2,1280	-5,0000e-3	-0,23	3,9000	P21/c	296	2011
DACKAV	177,8	2,1280	2,1280	0,0000	0,00	3,9000	P21/c	296	2011
DAPZEA	175,7	2,1390	2,1150	0,0240	1,13	6,0200	P21/n	293	2005
DATJOZ	180,0	2,1790	2,1790	0,0000	0,00	4,4300	P-1	296	2012
DATJOZ	174,3	2,1610	2,1590	2,0000e-3	0,09	4,4300	P-1	296	2012
DAXHIU	171,9	2,1580	2,1580	0,0000	0,00	3,0600	C2/c	273	2005
DAXZEK	171,0	2,1260	2,1320	-6,0000e-3	-0,28	7,5900	P-1	293	2016
DAXZIO	171,8	2,1250	2,1220	3,0000e-3	0,14	8,9000	P-1	293	2016
DAYZIM	180,0	2,1670	2,1670	0,0000	0,00	4,1000	I41/acd	295	1985
DEBGOI	179,2	2,1380	2,1360	2,0000e-3	0,09	9,2400	P21/c	293	2012
DEBGOI	179,2	2,1220	2,1520	-0,0300	-1,39	9,2400	P21/c	293	2012
DEWLUP	178,2	2,1650	2,1590	6,0000e-3	0,28	5,1700	P-1	293	2018
DEWLUP	180,0	2,1470	2,1470	0,0000	0,00	5,1700	P-1	293	2018
DITCEO	173,7	2,1570	2,1490	8,0000e-3	0,37	3,3000	I-4	295	1985
DOHKIX	180,0	2,2230	2,2230	0,0000	0,00	3,4600	P-1	296	2019
DOHXOP	170,3	2,1720	2,1690	3,0000e-3	0,14	5,9000	P21/n	293	2014
DOHXUV	172,6	2,1170	2,1270	-0,0100	-0,47	4,1700	C2/c	100	2014
DONMIF	174,3	2,1220	2,1310	-9,0000e-3	-0,42	3,8500	P-1	100	2019
DONMIF	174,3	2,1320	2,1320	0,0000	0,00	3,8500	P-1	100	2019
DOQHEY	174,8	2,1540	2,1680	-0,0140	-0,65	5,2900	P21/n	173	2015
DUHHIY	172,6	2,1690	2,1590	0,0100	0,46	6,0300	P-1	173	2009
DUQTOZ	173,4	2,1520	2,1460	6,0000e-3	0,28	2,5600	P-1	296	2010
ECOYOM	180,0	2,1780	2,1780	0,0000	0,00	4,6200	P-1	293	2012
EFIWUL	171,4	2,1960	2,1990	-3,0000e-3	-0,14	4,2500	P21/c	298	2002
EHIHEK	171,0	2,1420	2,1400	2,0000e-3	0,09	6,2700	P-1	293	2016
EHIHIO	170,4	2,1320	2,1370	-5,0000e-3	-0,23	5,0200	P-1	293	2016
EJIMUG	173,0	2,2280	2,2000	0,0280	1,27	6,7800	C2/c	296	2011
ELAVET	170,9	2,1220	2,1380	-0,0160	-0,75	6,7800	P-1	296	2011
ELAVET	170,8	2,1430	2,1390	4,0000e-3	0,19	6,7800	P-1	296	2011
ELEVEW	180,0	2,1340	2,1340	0,0000	0,00	3,4500	P21/c	200	2003
EMEJOW	177,3	2,1190	2,1270	-8,0000e-3	-0,38	5,8300	P-1	295	2010
EMEJOW	172,3	2,1360	2,1340	2,0000e-3	0,09	5,8300	P-1	295	2010
EMOVIM	173,9	2,1630	2,1620	1,0000e-3	0,05	3,8800	P-1	173	2011
ENEYEC	180,0	2,1770	2,1770	0,0000	0,00	8,4400	P-1	173	2010
ENEYOM	180,0	2,1370	2,1370	0,0000	0,00	4,2600	P21/n	173	2010
ESETOL	180,0	2,1620	2,1620	0,0000	0,00	3,3100	P-1	293	2004

ESEYIK	171,7	2,1600	2,1770	-0,0170	-0,78	8,6300	C2/c	298	2004
ESEYIK01	171,1	2,1560	2,1420	0,0140	0,65	5,0500	P-1	298	2005
ESEYIK01	173,3	2,1630	2,1760	-0,0130	-0,60	5,0500	P-1	298	2005
ESEYOQ	175,6	2,1200	2,1110	9,0000e-3	0,43	8,3100	C2/c	293	2004
ESEYUW	177,7	2,1460	2,1470	-1,0000e-3	-0,05	5,5600	P21/c	298	2004
ETOQIN	175,4	2,1970	2,1860	0,0110	0,50	7,3000	C2	293	2004
ETOQIN	176,3	2,2230	2,2230	0,0000	0,00	7,3000	C2	293	2004
EWIZAN	174,8	2,1040	2,1080	-4,0000e-3	-0,19	1,9400	P21/c	100	2016
EYAQAW	175,3	2,1270	2,1510	-0,0240	-1,12	6,7400	P-1	293	2004
EZARUT	171,7	2,1360	2,1360	0,0000	0,00	4,6300	Cmca	296	2011
EZASAA	174,2	2,1390	2,1460	-7,0000e-3	-0,33	4,3400	P21/n	296	2011
EZASEE	172,0	2,1450	2,1500	-5,0000e-3	-0,23	3,9600	P-1	296	2011
EZECAO	171,2	2,1250	2,1350	-0,0100	-0,47	2,8300	P21/n	173	2011
EZUKOZ	180,0	2,2120	2,2120	0,0000	0,00	5,6600	P-1	120	2004
EZUKOZ01	180,0	2,2120	2,2120	0,0000	0,00	5,6600	P-1	120	2005
EZUKUF	174,5	2,0940	2,0940	0,0000	0,00	3,0700	P-421c	150	2004
EZUKUF01	174,6	2,0930	2,0930	0,0000	0,00	2,8800	P-421c	150	2005
FARHUE	172,1	2,1730	2,1600	0,0130	0,60	4,5800	P21/n	301	2017
FEGFII	175,5	2,1750	2,1640	0,0110	0,51	2,7900	P21/c	200	2013
FELFOS	175,0	2,1270	2,1310	-4,0000e-3	-0,19	3,1600	C2/c	120	2005
FEPBUZ	180,0	2,1340	2,1340	0,0000	0,00	5,7800	C2/c	90	2013
FIDGEF	175,5	2,1460	2,1170	0,0290	1,37	3,9800	Fdd2	293	2005
FIDGEF	174,5	2,1420	2,1330	9,0000e-3	0,42	3,9800	Fdd2	293	2005
FIFYOJ	171,9	2,1350	2,1310	4,0000e-3	0,19	2,5600	C2/c	100	2005
FIGWID	180,0	2,1310	2,1310	0,0000	0,00	8,4700	P-1	293	2013
FIGWID	171,3	2,1390	2,1240	0,0150	0,71	8,4700	P-1	293	2013
FIGWOJ	170,7	2,1320	2,1390	-7,0000e-3	-0,33	9,1300	P-1	173	2013
FIGWOJ	180,0	2,1350	2,1350	0,0000	0,00	9,1300	P-1	173	2013
FIGWUP	180,0	2,1260	2,1260	0,0000	0,00	6,0400	P-1	173	2013
FINQOK	175,6	2,1430	2,1470	-4,0000e-3	-0,19	8,0300	P-1	298	2013
FINQUQ	174,3	2,1320	2,1260	6,0000e-3	0,28	3,0800	P-1	298	2013
FIWJAZ	180,0	2,1220	2,1220	0,0000	0,00	1,9700	P21/n	150	2019
FIZCIB	177,7	2,1300	2,1250	5,0000e-3	0,24	4,7300	P2/c	150	2005
FIZCOH	173,0	2,1430	2,1490	-6,0000e-3	-0,28	6,9400	C2/c	150	2005
FIZCOH	170,3	2,1100	2,1280	-0,0180	-0,85	6,9400	C2/c	150	2005
FOCGIO	176,1	2,1130	2,1300	-0,0170	-0,80	8,8200	C2/c	150	2005
FOCGIO	174,5	2,1140	2,1260	-0,0120	-0,56	8,8200	C2/c	150	2005
FONRUW	172,4	2,1730	2,1720	1,0000e-3	0,05	3,9800	P-1	293	2009
FONSAD	176,9	2,1300	2,1340	-4,0000e-3	-0,19	4,6600	P21/c	298	2009
FOPJUP	175,3	2,1460	2,1460	0,0000	0,00	4,1000	P-1	295	1987
FOPPIK	180,0	2,1420	2,1420	0,0000	0,00	6,6700	P-1	293	2009
FOPPIK	172,7	2,1070	2,1580	-0,0510	-2,36	6,6700	P-1	293	2009
FUFVOT	175,4	2,1670	2,1670	0,0000	0,00	9,6700	C2/c	296	2015
FUFVUZ	176,2	2,1670	2,1670	0,0000	0,00	5,4900	C2/c	293	2015
FUFWAG	176,5	2,1590	2,1590	0,0000	0,00	5,3100	C2/c	293	2015
FUFWEK	176,7	2,1640	2,1640	0,0000	0,00	5,4400	C2/c	293	2015
FUJLEC	177,4	2,1510	2,1460	5,0000e-3	0,23	10,7700	P2/c	150	2010
FUJLOM	180,0	2,1080	2,1080	0,0000	0,00	5,4600	P21/c	150	2010
GAKNEM	175,2	2,1550	2,1510	4,0000e-3	0,19	6,7200	P21/c	293	2004
GALCUT	174,1	2,1550	2,1550	0,0000	0,00	7,7100	Cmc21	296	2012
GEFDEB	177,5	2,1070	2,1240	-0,0170	-0,80	4,6300	C2/c	293	2006
GEXFOG	171,1	2,1520	2,0740	0,0780	3,76	7,2100	P-1	293	2013
GEXGAT	175,5	2,1230	2,1320	-9,0000e-3	-0,42	5,5600	C2/c	293	2013
GEXGAT	174,3	2,1360	2,1380	-2,0000e-3	-0,09	5,5600	C2/c	293	2013
GIHXOL	175,4	2,1520	2,1460	6,0000e-3	0,28	3,0400	P-1	173	2007
GIKJAN	174,6	2,1120	2,1210	-9,0000e-3	-0,42	4,2000	P-1	298	2013
GOBQEU01	180,0	2,1800	2,1800	0,0000	0,00	4,3400	P-1	153	2011
GOBQEU01	180,0	2,2000	2,2000	0,0000	0,00	4,3400	P-1	153	2011
GUCYIO	180,0	2,1070	2,1070	0,0000	0,00	4,6700	P-1	100	2015
GUCYIO	177,7	2,1110	2,1100	1,0000e-3	0,05	4,6700	P-1	100	2015
GUCYIO	180,0	2,1130	2,1130	0,0000	0,00	4,6700	P-1	100	2015
GUCYIO	178,6	2,1140	2,1130	1,0000e-3	0,05	4,6700	P-1	100	2015
GUCYIO	176,0	2,1150	2,1200	-5,0000e-3	-0,24	4,6700	P-1	100	2015
GUCZAH	174,2	2,1250	2,1150	0,0100	0,47	4,5900	P-1	100	2015
GUCZAH	175,9	2,1390	2,1260	0,0130	0,61	4,5900	P-1	100	2015
GUCZEL	172,8	2,1280	2,1240	4,0000e-3	0,19	2,7900	P-1	100	2015
GUCZEL	176,3	2,1520	2,0890	0,0630	3,02	2,7900	P-1	100	2015
GUCZIP	178,4	2,1480	2,1480	0,0000	0,00	2,9300	P-1	100	2015
GUCZIP	177,3	2,1750	2,1780	-3,0000e-3	-0,14	2,9300	P-1	100	2015
GUCZOV	175,7	2,1380	2,1090	0,0290	1,38	2,1500	C2/c	100	2015
GUKXOA	180,0	2,1840	2,1840	0,0000	0,00	8,4600	C2/m	293	2009
GUPPOW	173,0	2,1210	2,1170	4,0000e-3	0,19	3,5100	P1	295	2000
GUPPOW01	171,4	2,1340	2,1350	-1,0000e-3	-0,05	5,9300	P-1	294	2001
GUPPOW02	171,3	2,1340	2,1310	3,0000e-3	0,14	0,0000	P-1	295	2005
GUXTOJ	172,9	2,1610	2,1580	3,0000e-3	0,14	4,6500	P-1	150	2009
HEFFII	170,2	2,1320	2,1330	-1,0000e-3	-0,05	4,1200	P-1	120	2006
HEFFII	173,3	2,1480	2,1470	1,0000e-3	0,05	4,1200	P-1	120	2006
HEFFOO	178,9	2,1090	2,1140	-5,0000e-3	-0,24	5,8700	Cc	120	2006

HEFFOO	178,0	2,1320	2,1250	7,0000e-3	0,33	5,8700	Cc	120	2006
HEQTOO	174,8	2,1570	2,1520	5,0000e-3	0,23	2,7000	P21/n	143	2013
HETQEC	175,8	2,1030	2,1110	-8,0000e-3	-0,38	5,3000	P21/c	173	1998
HIQHIZ	179,1	2,1710	2,1730	-2,0000e-3	-0,09	3,1200	P21/n	294	2007
HIWHEB	176,3	2,1000	2,1220	-0,0220	-1,04	3,8200	P-1	193	2007
HOVJOT	173,6	2,1550	2,1470	8,0000e-3	0,37	4,0800	P-1	296	2014
HOYZEC	175,2	2,0970	2,0970	0,0000	0,00	9,5200	C2/c	190	2014
HOYZEC	177,4	2,1290	2,1390	-0,0100	-0,47	9,5200	C2/c	190	2014
HUTSOE	180,0	2,1620	2,1620	0,0000	0,00	4,4900	P-1	293	2003
HUTSUK	180,0	2,1710	2,1710	0,0000	0,00	4,3700	C2/c	298	2003
HUWCAE	173,0	2,1110	2,1170	-6,0000e-3	-0,28	3,7000	P21/c	223	2009
IBANUX	180,0	2,1540	2,1540	0,0000	0,00	1,8300	C2/c	133	2016
IBAXAN	170,4	2,1460	2,1430	3,0000e-3	0,14	3,0900	P-1	133	2016
IGOQEB	172,2	2,1530	2,1590	-6,0000e-3	-0,28	4,0200	P-1	100	2009
IGUPOP	172,5	2,1660	2,1790	-0,0130	-0,60	2,8700	P-1	168	2002
ILUJOO	177,0	2,1300	2,1380	-8,0000e-3	-0,37	6,9500	P21/n	120	2003
INAKUE	174,3	2,1730	2,1690	4,0000e-3	0,18	3,9100	Cmca	298	2010
INALEP	177,7	2,1080	2,1080	0,0000	0,00	2,7800	P21/c	298	2010
ISECIT	175,0	2,1730	2,1640	9,0000e-3	0,42	3,4600	P21	173	2011
IXIROX	176,2	2,2000	2,1900	0,0100	0,46	2,4700	Pbcn	173	2009
IXIRUD	174,2	2,1330	2,1410	-8,0000e-3	-0,37	4,6300	Pbcn	173	2009
IZEYAP	180,0	2,1410	2,1410	0,0000	0,00	1,7800	Pnnm	133	2016
IZEYUJ	180,0	2,1470	2,1470	0,0000	0,00	3,0000	P-1	133	2016
IZIJUY	180,0	2,1720	2,1720	0,0000	0,00	2,1100	P-1	133	2016
IZILOU	170,7	2,1390	2,1430	-4,0000e-3	-0,19	3,3000	Pna21	133	2016
JABSAH	176,7	2,1350	2,1210	0,0140	0,66	6,1900	P213	293	2003
JABSEL	173,7	2,1660	2,1660	0,0000	0,00	4,9300	P-1	293	2003
JAGRIT	177,1	2,1370	2,1410	-4,0000e-3	-0,19	4,3900	P-1	293	2003
JAGRIT	179,1	2,1390	2,1390	0,0000	0,00	4,3900	P-1	293	2003
JAJQEQ	171,7	2,1450	2,1450	0,0000	0,00	2,1100	C2/c	173	1998
JAJQOA	170,3	2,1350	2,1410	-6,0000e-3	-0,28	3,6200	P-1	173	1998
JAYBOB	172,9	2,1430	2,1430	0,0000	0,00	4,5500	C2/c	292	2005
JEFCUV	180,0	2,1250	2,1250	0,0000	0,00	4,3900	P-1	173	2016
JOKWEL	171,0	2,1520	2,1390	0,0130	0,61	6,5400	P-1	295	1992
JOKWEL	170,5	2,1570	2,1520	5,0000e-3	0,23	6,5400	P-1	295	1992
KAJGOT	175,4	2,1490	2,1480	1,0000e-3	0,05	3,2400	P-1	120	2010
KAJGUZ	173,5	2,2040	2,1830	0,0210	0,96	6,1700	Pmc21	173	2010
KAJGUZ	174,2	2,1750	2,1690	6,0000e-3	0,28	6,1700	Pmc21	173	2010
KALPAP	175,2	2,1800	2,1800	0,0000	0,00	5,6500	Pnma	293	2005
KAYSEJ	175,6	2,1140	2,1230	-9,0000e-3	-0,42	5,5800	C2/c	298	2004
KEJRIC	175,0	2,1220	2,1280	-6,0000e-3	-0,28	3,1200	P-1	113	2012
KIDYOM	178,5	2,1700	2,1010	0,0690	3,28	3,7000	P1	295	2007
KIDYOM01	179,8	2,1270	2,1270	0,0000	0,00	0,0000	P-1	295	2009
KIDZIH	179,3	2,0760	2,1180	-0,0420	-1,98	4,2100	P1	295	2007
KIDZIH01	179,6	2,1170	2,1360	-0,0190	-0,89	0,0000	P-1	295	2009
KIJREC	180,0	2,1540	2,1540	0,0000	0,00	3,7800	P-1	293	2013
KOLHIC	171,2	2,1690	2,1690	0,0000	0,00	2,9000	C2/c	295	1991
KULDUS	177,9	2,1220	2,1220	0,0000	0,00	10,2800	C2/m	123	2014
KULDUS	174,4	2,1600	2,1600	0,0000	0,00	10,2800	C2/m	123	2014
KULFAA	176,1	2,1760	2,1760	0,0000	0,00	6,9700	P-421c	173	2014
KULFAA	174,9	2,1400	2,1630	-0,0230	-1,06	6,9700	P-421c	173	2014
KULFAA	177,0	2,1350	2,1350	0,0000	0,00	6,9700	P-421c	173	2014
KUXWUW	171,2	2,1490	2,1280	0,0210	0,99	4,9400	P-1	296	2010
KUXXEH	172,9	2,1220	2,1240	-2,0000e-3	-0,09	2,5900	P1	296	2010
KUXXEH	177,5	2,1290	2,1320	-3,0000e-3	-0,14	2,5900	P1	296	2010
KUYNOI	180,0	2,1360	2,1360	0,0000	0,00	2,1400	P-1	113	2010
LAFPUE	180,0	2,1130	2,1130	0,0000	0,00	4,5500	P-1	293	2004
LALZIK	171,6	2,1070	2,1090	-2,0000e-3	-0,09	3,5700	I41	100	2016
LALZIK	170,4	2,1100	2,1070	3,0000e-3	0,14	3,5700	I41	100	2016
LALZIK	171,5	2,1100	2,1170	-7,0000e-3	-0,33	3,5700	I41	100	2016
LALZIK	171,6	2,1120	2,1190	-7,0000e-3	-0,33	3,5700	I41	100	2016
LALZIK	170,9	2,1130	2,1110	2,0000e-3	0,09	3,5700	I41	100	2016
LALZIK	170,6	2,1140	2,1200	-6,0000e-3	-0,28	3,5700	I41	100	2016
LALZUW	180,0	2,1140	2,1140	0,0000	0,00	4,8800	P21/c	100	2016
LALZUW	180,0	2,1160	2,1160	0,0000	0,00	4,8800	P21/c	100	2016
LAZYAP	180,0	2,1260	2,1260	0,0000	0,00	7,9600	C2/m	293	2017
LAZYAP	180,0	2,1560	2,1560	0,0000	0,00	7,9600	C2/m	293	2017
LETGEZ	177,5	2,1630	2,1730	-0,0100	-0,46	5,5200	Pbca	296	2017
LIVKEI	180,0	2,1320	2,1320	0,0000	0,00	9,4800	P-1	173	2014
LIVKEI	179,3	2,1500	2,1350	0,0150	0,70	9,4800	P-1	173	2014
LIWNIP	177,9	2,1510	2,1520	-1,0000e-3	-0,05	3,7200	R-3c	173	2007
LUKZAU	176,9	2,1330	2,1280	5,0000e-3	0,23	5,3500	P-1	173	2015
LUKZIC	174,8	2,1430	2,1300	0,0130	0,61	2,0300	P21/c	173	2015
LULNIR	176,0	2,1380	2,1370	1,0000e-3	0,05	3,0800	P21/n	170	2015
LULNOX	177,0	2,1310	2,1320	-1,0000e-3	-0,05	2,9400	P21/n	170	2015
MADYUM	171,3	2,1610	2,1700	-9,0000e-3	-0,41	6,9400	P65	173	2004
MADZAT	174,4	2,1860	2,1720	0,0140	0,64	7,0600	P21/n	173	2004
MAHNIT	178,2	2,1260	2,1260	0,0000	0,00	5,5200	Cmca	298	2004

MATFIY	174,7	2,1200	2,1210	-1,0000e-3	-0,05	6,5700	P-1	293	2012
MAXKAX	172,7	2,1540	2,1660	-0,0120	-0,55	2,5300	C2	203	2000
MIGQEY	173,3	2,1850	2,1850	0,0000	0,00	4,9300	P21/n	168	2002
MIGQEY	172,2	2,1810	2,1660	0,0150	0,69	4,9300	P21/n	168	2002
MIGQEY	172,4	2,2000	2,1930	7,0000e-3	0,32	4,9300	P21/n	168	2002
MIRGEZ	180,0	2,1640	2,1640	0,0000	0,00	4,7200	C2/c	293	2001
MOLKEF	175,8	2,1580	2,1620	-4,0000e-3	-0,19	3,5200	P-1	120	2013
MOLKEF	176,8	2,1490	2,1530	-4,0000e-3	-0,19	3,5200	P-1	120	2013
MOMZIY	174,6	2,1280	2,1280	0,0000	0,00	8,8000	P21/c	173	2009
MOMZUK	178,3	2,1340	2,1480	-0,0140	-0,65	8,2500	P21/c	173	2009
MOWNUI	180,0	2,1600	2,1600	0,0000	0,00	5,1900	C2/c	293	2009
MOYKIU	180,0	2,1200	2,1200	0,0000	0,00	3,5200	P-1	168	2002
MUBXEN	174,2	2,1470	2,1440	3,0000e-3	0,14	4,5700	P-1	173	2009
MUTMOD	174,2	2,1640	2,1610	3,0000e-3	0,14	3,6100	P-1	293	2003
NABYOF	180,0	2,1300	2,1300	0,0000	0,00	2,3700	P-1	293	2004
NABYOF	180,0	2,1320	2,1320	0,0000	0,00	2,3700	P-1	293	2004
NEGQOI	180,0	2,1380	2,1380	0,0000	0,00	1,6500	P-1	113	2017
NEGXIJ	174,6	2,1710	2,4890	-0,3180	-12,78	18,6800	C2221	90	2017
NEGYAC	173,8	2,0960	2,2270	-0,1310	-5,88	9,9700	P43	100	2017
NEGYAC	176,0	2,1760	2,2660	-0,0900	-3,97	9,9700	P43	100	2017
NENROP	177,6	2,1290	2,1190	0,0100	0,47	5,2300	P21/c	298	2012
NEVRAI	173,8	2,1260	2,1330	-7,0000e-3	-0,33	4,5700	Pbcn	150	2007
NEVREM	174,8	2,1270	2,1230	4,0000e-3	0,19	3,1600	Pbcn	150	2007
NEVRIQ	178,1	2,1290	2,1290	0,0000	0,00	5,3300	P42/nnm	298	2007
NIGHIX	177,9	2,1600	2,1560	4,0000e-3	0,19	4,6100	P-1	123	2017
NIGHIX	178,1	2,1600	2,1590	1,0000e-3	0,05	4,6100	P-1	123	2017
NISFUQ	176,1	2,1440	2,1430	1,0000e-3	0,05	4,2800	P-1	173	1998
NISJAA	171,0	2,1540	2,1520	2,0000e-3	0,09	2,6900	P-1	173	1998
NISNEI	174,9	2,1600	2,1650	-5,0000e-3	-0,23	2,8600	P-1	173	1998
NOMBID	177,6	2,1460	2,1590	-0,0130	-0,60	8,0400	P21/c	150	2019
NOMBID	178,3	2,1640	2,1560	8,0000e-3	0,37	8,0400	P21/c	150	2019
NOMBID	174,9	2,1540	2,1650	-0,0110	-0,51	8,0400	P21/c	150	2019
NOMBID	178,7	2,1510	2,1470	4,0000e-3	0,19	8,0400	P21/c	150	2019
NUNSEW	176,4	2,1430	2,1370	6,0000e-3	0,28	5,8200	P-1	295	2015
NUNSUM	180,0	2,1380	2,1380	0,0000	0,00	6,0900	P21/c	295	2015
NUVBUC	176,0	2,1530	2,1390	0,0140	0,65	16,1000	P21/c	298	2010
NUVCAJ	173,1	2,1600	2,1450	0,0150	0,70	8,8000	P21/m	298	2010
OBEWOK	171,3	2,1560	2,1540	2,0000e-3	0,09	7,7800	P21/c	200	2016
OBEWUQ	176,6	2,1490	2,1340	0,0150	0,70	9,8700	P21/n	200	2016
OFALUE	170,1	2,1540	2,1500	4,0000e-3	0,19	4,1700	P21/c	293	2013
OHAHOW	170,0	2,1860	2,1930	-7,0000e-3	-0,32	5,1800	P-1	100	2015
OJAFOW	180,0	2,1040	2,1040	0,0000	0,00	10,3600	C2/c	293	2015
OJAROI	172,4	2,1310	2,1340	-3,0000e-3	-0,14	5,9500	P-1	296	2015
OKUGOQ	176,4	2,1470	2,1450	2,0000e-3	0,09	3,7700	P-1	293	2001
OPUCUY	173,3	2,1950	2,1690	0,0260	1,20	3,8600	Cc	293	2011
OPUDAF	173,8	2,1850	2,1700	0,0150	0,69	3,7600	Cc	293	2011
OQINUY	176,4	2,1220	2,1100	0,0120	0,57	4,5600	P21/n	293	2011
OQINUY01	180,0	2,1350	2,1350	0,0000	0,00	4,1500	C2/c	293	2011
OQINUY02	180,0	2,1400	2,1400	0,0000	0,00	6,3000	P-1	293	2011
OSASEH	171,3	2,1460	2,1450	1,0000e-3	0,05	3,5000	P-1	90	2011
OSEWIT	174,3	2,1530	2,1290	0,0240	1,13	6,8900	P-1	293	2011
OSEWUF	172,9	2,1620	2,1610	1,0000e-3	0,05	3,8800	C2/c	293	2011
OTUVEG	174,6	2,1360	2,1440	-8,0000e-3	-0,37	6,0100	P-1	298	2016
OXUQAB	172,6	2,1340	2,1340	0,0000	0,00	8,5600	P-1	100	2016
PALSED	175,4	2,1520	2,1540	-2,0000e-3	-0,09	4,8200	P-1	296	2017
PAPSOQ	170,7	2,1240	2,1290	-5,0000e-3	-0,23	5,9000	P-1	293	2011
PASBES	180,0	2,1610	2,1610	0,0000	0,00	4,4000	C2/c	200	2012
PASBIW	171,9	2,1430	2,1440	-1,0000e-3	-0,05	2,3500	P-1	200	2012
PAVKIH	180,0	2,1650	2,1650	0,0000	0,00	10,3300	P21/c	150	2005
PAVKIH	176,4	2,1260	2,1100	0,0160	0,76	10,3300	P21/c	150	2005
PAVKIH	171,3	2,1740	2,1250	0,0490	2,31	10,3300	P21/c	150	2005
PEMGUL	178,5	2,1350	2,1440	-9,0000e-3	-0,42	12,0300	P21/n	173	2013
PEMHEW	178,9	2,0880	2,1010	-0,0130	-0,62	9,5200	P21/c	173	2013
PICVOO	172,3	2,1420	2,1420	0,0000	0,00	4,6000	C2/c	153	2013
PIGPAZ	170,9	2,1490	2,1410	8,0000e-3	0,37	4,2900	C2/c	100	2018
PIHTUY	174,2	2,1360	2,1460	-0,0100	-0,47	8,6800	P-1	298	2018
PIZJOZ	180,0	2,1690	2,1690	0,0000	0,00	4,1600	P-1	100	2014
PIZJOZ	180,0	2,2240	2,2240	0,0000	0,00	4,1600	P-1	100	2014
PIZKUF	180,0	2,1190	2,1190	0,0000	0,00	4,6600	P-1	273	2008
PIZKUF	180,0	2,1090	2,1090	0,0000	0,00	4,6600	P-1	273	2008
POCPOM	180,0	2,1660	2,1660	0,0000	0,00	6,7000	P-1	295	1994
POGCIZ	176,2	2,1620	2,1790	-0,0170	-0,78	7,9400	P6522	90	2014
POGDAS	176,2	2,1530	2,1250	0,0280	1,32	9,7700	P6522	90	2014
POKYIY	180,0	2,1820	2,1820	0,0000	0,00	4,7000	P-1	223	2008
PURXUW	174,4	2,1450	2,1450	0,0000	0,00	4,6000	P21/n	150	2010
PURYAD	172,2	2,1420	2,1470	-5,0000e-3	-0,23	4,3000	P-1	150	2010
PURYUX	175,7	2,1410	2,1410	0,0000	0,00	3,2000	P-1	150	2010
QAFQIY	178,1	2,1790	2,1700	9,0000e-3	0,41	2,3600	P-1	173	2003

QAGTUN	180,0	2,1350	2,1350	0,0000	0,00	2,6800	C2/c	173	1999
QANXOV	175,9	2,2140	2,2170	-3,0000e-3	-0,14	5,7600	P-1	150	2017
QANXUB	175,9	2,1520	2,1400	0,0120	0,56	2,1900	P21	150	2017
QANZOX	178,4	2,1330	2,1380	-5,0000e-3	-0,23	9,4600	P-1	150	2017
QANZOX	177,4	2,1450	2,1650	-0,0200	-0,92	9,4600	P-1	150	2017
QAPPIH	180,0	2,1170	2,1170	0,0000	0,00	2,9800	P-1	150	2005
QARNOM	178,4	2,1370	2,1320	5,0000e-3	0,23	7,6000	P-1	213	2000
QAWQEL	177,6	2,1300	2,1300	0,0000	0,00	2,2400	P21/n	293	2005
QEKBIS	175,0	2,1270	2,1180	9,0000e-3	0,42	5,8200	P-1	200	2006
QIBHIT	173,5	2,1560	2,1560	0,0000	0,00	3,7100	P21/n	150	2007
QIBHIT01	170,7	2,1540	2,1530	1,0000e-3	0,05	4,1400	P21/c	150	2007
QIBHIT01	171,5	2,1830	2,1790	4,0000e-3	0,18	4,1400	P21/c	150	2007
QIWROG	174,8	2,1730	2,1710	2,0000e-3	0,09	9,2700	P-1	173	2019
QIWSUN	170,9	2,1370	2,1480	-0,0110	-0,51	14,7300	P-1	173	2019
QIYGUB	173,9	2,1500	2,1530	-3,0000e-3	-0,14	7,3100	P-1	173	2008
QOKBEZ	174,0	2,1390	2,1460	-7,0000e-3	-0,33	5,3000	C2/c	298	2014
QQQBUV01	180,0	2,1280	2,1280	0,0000	0,00	3,8000	C2/c	293	2005
QUDMIM	171,3	2,1900	2,1810	9,0000e-3	0,41	5,5900	P21/c	293	2009
QUPJIV01	170,7	2,1560	2,1590	-3,0000e-3	-0,14	2,9800	P21/n	150	2010
QUPKAO	174,7	2,1490	2,1380	0,0110	0,51	4,3400	P-1	290	2010
QUPKAO01	172,4	2,1430	2,1340	9,0000e-3	0,42	2,8100	P-1	190	2010
QUPKUI	170,2	2,1660	2,1560	0,0100	0,46	4,1800	P21/n	290	2010
RADWAV	176,3	2,1450	2,1440	1,0000e-3	0,05	9,1000	P21/n	100	2004
RAKGIU	177,2	2,1250	2,1290	-4,0000e-3	-0,19	4,7500	P21/c	296	2005
RAKGIU	171,1	2,1270	2,1340	-7,0000e-3	-0,33	4,7500	P21/c	296	2005
RANHAQ	180,0	2,1850	2,1850	0,0000	0,00	4,2600	P21/c	294	2005
RASGOI	174,6	2,1100	2,1130	-3,0000e-3	-0,14	3,4700	P-1	173	2005
RAWTOZ	180,0	2,1520	2,1520	0,0000	0,00	4,8000	P-1	293	2005
RAYWUK	172,5	2,1400	2,1380	2,0000e-3	0,09	5,1000	P-1	293	2005
RAYXOF	173,9	2,1620	2,1620	0,0000	0,00	3,2000	Pbcn	293	2005
RAYXOF01	176,7	2,1270	2,1410	-0,0140	-0,65	4,1000	P61	153	2005
RAYZAT	180,0	2,1300	2,1300	0,0000	0,00	3,9000	P-1	293	2005
RAYZAT	180,0	2,1380	2,1380	0,0000	0,00	3,9000	P-1	293	2005
RAYZEX	174,3	2,1420	2,1480	-6,0000e-3	-0,28	3,8000	P-1	293	2005
RAYZOH	175,4	2,1730	2,1730	0,0000	0,00	5,3000	C2/c	293	2005
RAZBEA	175,2	2,1230	2,1330	-0,0100	-0,47	5,0000	P21/c	293	2005
REHJEV	180,0	2,1210	2,1210	0,0000	0,00	5,3900	C2/c	293	2012
REKHOE	170,0	2,2040	2,1920	0,0120	0,55	11,0500	P-1	295	1997
RENNUU	179,0	2,1270	2,1470	-0,0200	-0,93	10,6000	P21/c	111	2006
RIBZIM	180,0	2,1560	2,1560	0,0000	0,00	3,5000	Fddd	295	2007
RICZAH	177,9	2,1090	2,1050	4,0000e-3	0,19	4,6600	C2/c	298	2018
RIDHAQ	174,9	2,1530	2,1420	0,0110	0,51	6,1300	P21/c	298	2018
RIDHEU	178,0	2,1620	2,1650	-3,0000e-3	-0,14	7,7100	Cmca	298	2018
RIRPOZ	180,0	2,1270	2,1270	0,0000	0,00	2,0500	C2/c	120	2014
RIRQEQ	173,7	2,2460	2,2600	-0,0140	-0,62	2,6600	P-1	120	2014
RIRQEQ	177,3	2,2820	2,2770	5,0000e-3	0,22	2,6600	P-1	120	2014
RISDII	179,3	2,1600	2,1620	-2,0000e-3	-0,09	3,5400	P21/c	220	2014
RIYNEU	172,0	2,1540	2,1430	0,0110	0,51	8,6000	C2/c	290	2014
ROPBIJ	171,8	2,1350	2,1530	-0,0180	-0,84	3,5100	P21	293	2014
ROPBIJ	172,0	2,1360	2,1340	2,0000e-3	0,09	3,5100	P21	293	2014
SADWOL	178,1	2,1560	2,1640	-8,0000e-3	-0,37	3,6000	P21/n	296	2012
SAJKEU	180,0	2,2000	2,2000	0,0000	0,00	7,0900	P-1	193	2004
SARPEH	174,5	2,1340	2,1350	-1,0000e-3	-0,05	5,0000	P-1	123	2005
SARPEH	174,3	2,1430	2,1550	-0,0120	-0,56	5,0000	P-1	123	2005
SARPIL	180,0	2,1870	2,1870	0,0000	0,00	3,1100	C2/c	123	2005
SEDJAN	171,5	2,1470	2,1300	0,0170	0,80	3,9300	P-1	123	2006
SEDJAN	172,3	2,1200	2,1270	-7,0000e-3	-0,33	3,9300	P-1	123	2006
SEDJER	175,2	2,1510	2,1220	0,0290	1,37	11,3800	P21/c	123	2006
SEDJER	172,5	2,1680	2,1080	0,0600	2,85	11,3800	P21/c	123	2006
SEDJIV	180,0	2,1210	2,1210	0,0000	0,00	5,8200	P-1	123	2006
SEHBEO	180,0	2,1910	2,1910	0,0000	0,00	2,5600	P-1	296	2012
SEHBIS	180,0	2,1590	2,1590	0,0000	0,00	3,2700	C2/c	296	2012
SEHBOY	173,6	2,1720	2,1780	-6,0000e-3	-0,28	4,4500	P-1	296	2012
SEHBUE	174,1	2,1390	2,1390	0,0000	0,00	4,0100	P-1	296	2012
SEHCAL	174,6	2,1410	2,1410	0,0000	0,00	3,7100	P-1	296	2012
SESSEP	174,6	2,1330	2,1340	-1,0000e-3	-0,05	4,0200	P21/n	295	2006
SIKKIH	180,0	2,1320	2,1320	0,0000	0,00	6,8200	P21/c	153	2007
SOMQAM	174,3	2,1440	2,1480	-4,0000e-3	-0,19	1,8400	P-1	173	1998
SOMQEQ	177,3	2,1470	2,1410	6,0000e-3	0,28	4,8100	P21/c	173	1998
SOMQIU	173,4	2,1590	2,1540	5,0000e-3	0,23	2,4500	P21/c	173	1998
SUHCIJ	177,4	2,1060	2,1040	2,0000e-3	0,10	4,4100	P-1	100	2014
SUHCOP	180,0	2,1640	2,1640	0,0000	0,00	4,5000	P-1	100	2014
SUHCOP	180,0	2,2040	2,2040	0,0000	0,00	4,5000	P-1	100	2014
SUPZUA	171,8	2,1330	2,1440	-0,0110	-0,51	5,8900	P-1	293	2015
TAGNIA	171,9	2,2780	2,2780	0,0000	0,00	4,1500	P2/c	291	2010
TAGNOG	171,9	2,2670	2,2670	0,0000	0,00	2,7100	P2/c	291	2010
TEBXUU	178,5	2,1920	2,1790	0,0130	0,60	9,5200	P21/c	273	2005
TEBXUU	172,6	2,2070	2,2040	3,0000e-3	0,14	9,5200	P21/c	273	2005

TEBYEF	172,3	2,1460	2,2060	-0,0600	-2,72	5,7300	Pn	273	2005
TEBYEF	176,1	2,1790	2,1400	0,0390	1,82	5,7300	Pn	273	2005
TEPFUR	172,2	2,1430	2,1530	-0,0100	-0,46	10,5300	Ibam	123	2013
TEPFUR	174,0	2,1540	2,1540	0,0000	0,00	10,5300	Ibam	123	2013
TEPFUR	178,0	2,1320	2,1320	0,0000	0,00	10,5300	Ibam	123	2013
TEPFUR	174,4	2,1580	2,1580	0,0000	0,00	10,5300	Ibam	123	2013
TEPFUR	175,4	2,1260	2,1260	0,0000	0,00	10,5300	Ibam	123	2013
TEPGAY	173,0	2,1390	2,1390	0,0000	0,00	8,4300	P42/mbc	298	2013
TEPGAY	171,2	2,1600	2,1600	0,0000	0,00	8,4300	P42/mbc	298	2013
TEPWES	174,7	2,1270	2,1410	-0,0140	-0,65	4,1300	P21/c	200	2013
TEQRIR	172,7	2,1620	2,1800	-0,0180	-0,83	4,8800	P21/c	168	2006
TERMOT	173,1	2,1300	2,1300	0,0000	0,00	4,7800	C2/c	293	2006
TEWPIW	171,7	2,1120	2,1450	-0,0330	-1,54	6,1900	P1	298	2012
TIJFOH	175,0	2,1360	2,1330	3,0000e-3	0,14	6,3000	P-1	293	2001
TIJFOH	180,0	2,1410	2,1410	0,0000	0,00	6,3000	P-1	293	2001
TIJFOH	180,0	2,1760	2,1760	0,0000	0,00	6,3000	P-1	293	2001
TOGXAR	179,1	2,1420	2,1320	0,0100	0,47	5,2000	Pbcn	294	2019
TOHJUX	173,4	2,1800	2,1600	0,0200	0,93	7,7200	P-1	173	2014
TOLBUS	178,2	2,1300	2,1370	-7,0000e-3	-0,33	2,4600	P-1	100	2008
TOLCAZ	178,7	2,1330	2,1400	-7,0000e-3	-0,33	2,1200	P-1	100	2008
TOLCIH	177,1	2,1070	2,1040	3,0000e-3	0,14	2,7800	P-1	100	2008
TOLDAA	177,9	2,1370	2,1310	6,0000e-3	0,28	5,5500	P-1	100	2008
TORWAZ	178,2	2,1380	2,1300	8,0000e-3	0,38	2,8300	P-1	100	2008
TORWAZ01	178,6	2,1380	2,1310	7,0000e-3	0,33	2,8900	P-1	100	2008
UBONEE	177,0	2,1520	2,1540	-2,0000e-3	-0,09	3,6600	P21/n	173	2001
UFALEV	180,0	2,1930	2,1930	0,0000	0,00	10,1100	Pbca	293	2018
UGADAI	180,0	2,1120	2,1120	0,0000	0,00	3,5300	C2/c	100	2008
UGADAI	176,0	2,1410	2,1420	-1,0000e-3	-0,05	3,5300	C2/c	100	2008
UGEKOH	176,3	2,1310	2,1310	0,0000	0,00	7,8500	Ibca	293	2009
UGEKUN	173,1	2,1260	2,1260	0,0000	0,00	8,0200	Ibca	293	2009
UHEXOV	178,4	2,1510	2,1490	2,0000e-3	0,09	3,4900	R-3c	173	2009
UHEXUB	179,4	2,1470	2,1490	-2,0000e-3	-0,09	4,1600	R-3c	173	2009
UHEYAI	172,0	2,1840	2,1840	0,0000	0,00	2,3100	C2/c	173	2009
UHEYEM	176,1	2,1360	2,1350	1,0000e-3	0,05	3,7900	P-1	173	2009
ULAGET	170,9	2,1300	2,1360	-6,0000e-3	-0,28	2,5200	P-1	150	2003
ULOHOT	174,2	2,1830	2,1790	4,0000e-3	0,18	3,5500	P212121	113	2011
ULOJUB	178,1	2,1540	2,1620	-8,0000e-3	-0,37	4,5300	P21	113	2011
ULOJUB	175,4	2,1190	2,1190	0,0000	0,00	4,5300	P21	113	2011
UNABIW	175,9	2,1260	2,1260	0,0000	0,00	7,3500	C2/m	173	2016
UNABIW	176,1	2,1550	2,1550	0,0000	0,00	7,3500	C2/m	173	2016
UNURAY	175,2	2,1450	2,1490	-4,0000e-3	-0,19	4,7900	R-3c	293	2016
UNUREC	178,6	2,1580	2,1580	0,0000	0,00	4,9900	Pnma	100	2016
UNURIG	178,3	2,2390	2,2390	0,0000	0,00	6,6300	C2/c	173	2016
UTAFAX	174,0	2,2000	2,1540	0,0460	2,14	10,3700	Cc	150	2011
UTAFAX	174,6	2,1900	2,1310	0,0590	2,77	10,3700	Cc	150	2011
UTAFAX	176,9	2,1400	2,1070	0,0330	1,57	10,3700	Cc	150	2011
UTAFAX	173,4	2,1520	2,1340	0,0180	0,84	10,3700	Cc	150	2011
UWIHUE	180,0	2,1330	2,1330	0,0000	0,00	8,7300	C2/m	123	2011
UWIHUE	179,6	2,1800	2,1800	0,0000	0,00	8,7300	C2/m	123	2011
UWIKUH	178,0	2,1530	2,1690	-0,0160	-0,74	6,1500	C2	293	2011
UWIKUH	178,0	2,1570	2,1610	-4,0000e-3	-0,19	6,1500	C2	293	2011
UWIKUH01	178,1	2,1550	2,1660	-0,0110	-0,51	0,0000	Fdd2	293	2014
UXIWIJ	176,8	2,1420	2,1420	0,0000	0,00	3,8700	Fdd2	293	2016
VAPDIB	178,1	2,1650	2,1650	0,0000	0,00	4,0200	Pccn	296	2012
VARKIL	170,6	2,1470	2,1490	-2,0000e-3	-0,09	5,3300	P21/n	295	2017
VATXUL	171,7	2,1960	2,1870	9,0000e-3	0,41	5,7600	C2/c	296	2012
VATYAS	174,4	2,2160	2,1990	0,0170	0,77	5,6100	C2/c	296	2012
VATYEW	172,9	2,2220	2,2080	0,0140	0,63	3,9000	C2/c	296	2012
VAZFUY	174,9	2,1180	2,1210	-3,0000e-3	-0,14	3,2000	P-1	295	2005
VEFVAF	177,5	2,1730	2,1770	-4,0000e-3	-0,18	9,2900	P21	296	2012
VEFVAF	172,6	2,1890	2,1880	1,0000e-3	0,05	9,2900	P21	296	2012
VETGUX	180,0	2,1760	2,1760	0,0000	0,00	4,1500	C2/c	293	2006
VIGDEW	178,3	2,1470	2,1300	0,0170	0,80	6,2300	P21/n	298	2012
VIKFUT	180,0	2,1170	2,1170	0,0000	0,00	11,8900	P-1	120	2018
VIKGEE	180,0	2,1450	2,1450	0,0000	0,00	7,3000	P-1	168	2018
VINZID	180,0	2,1420	2,1420	0,0000	0,00	9,6600	P-1	293	2013
VINZID	180,0	2,1970	2,1970	0,0000	0,00	9,6600	P-1	293	2013
VIRDAC	178,8	2,1310	2,1080	0,0230	1,09	9,0900	P-1	153	2007
VODCUN	174,2	2,1100	2,1140	-4,0000e-3	-0,19	5,9100	P21/a	293	2008
VONBEH	180,0	2,1580	2,1580	0,0000	0,00	7,5100	P-1	297	2014
VUDKAI	180,0	2,0860	2,0860	0,0000	0,00	8,1500	P21/c	173	2015
VUDKAI	180,0	2,0980	2,0980	0,0000	0,00	8,1500	P21/c	173	2015
VULVUU	175,6	2,1280	2,1280	0,0000	0,00	4,5700	Cmc21	293	2009
VUVGUQ	172,1	2,1290	2,1390	-0,0100	-0,47	4,1000	P-1	298	2015
WATKOU	172,0	2,1600	2,1390	0,0210	0,98	7,2900	P-1	296	2017
WATKUA	175,5	2,1620	2,1440	0,0180	0,84	7,5600	P-1	296	2017
WAZNIW	170,5	2,1840	2,2010	-0,0170	-0,77	5,3800	P-1	298	2012
WEBNEZ	180,0	2,1370	2,1370	0,0000	0,00	5,8200	P-1	298	2014

WEGBUI	177,5	2,1240	2,1220	2,0000e-3	0,09	9,2300	C2	98	2017
WEYDAI	170,1	2,1710	2,1800	-9,0000e-3	-0,41	7,6100	P21/c	273	2018
WIHWER	171,3	2,1480	2,1340	0,0140	0,66	5,5300	P21/c	295	2013
WITREY	178,8	2,1600	2,1600	0,0000	0,00	10,4100	Ibam	120	2014
WIWKUK	173,8	2,1570	2,1570	0,0000	0,00	8,7100	C2	123	2014
WOHKAG	176,4	2,1380	2,1380	0,0000	0,00	2,3900	C2/c	173	2008
WOHKAG01	176,4	2,1300	2,1300	0,0000	0,00	2,1000	C2/c	173	2018
WOLSAS	176,0	2,2050	2,1690	0,0360	1,66	5,7900	I-4	150	2008
WUFMIT	176,2	2,1270	2,1340	-7,0000e-3	-0,33	5,8800	P-1	293	2002
WUFMOZ	178,0	2,1590	2,1270	0,0320	1,50	6,3700	P21/c	293	2002
WUFMOZ	176,0	2,1370	2,1310	6,0000e-3	0,28	6,3700	P21/c	293	2002
XAZGIR	180,0	2,1390	2,1390	0,0000	0,00	6,3500	P21/c	150	2011
XECZUB01	171,0	2,1750	2,1680	7,0000e-3	0,32	2,7100	P-1	173	2007
XEDZOV	173,1	2,1400	2,1370	3,0000e-3	0,14	3,0600	P21/c	295	2000
XEDZUB	172,5	2,1380	2,1450	-7,0000e-3	-0,33	5,4700	P21/c	295	2000
XEFBAL	172,8	2,1480	2,1500	-2,0000e-3	-0,09	3,7200	P21/c	295	2000
XEFBEP	172,6	2,1350	2,1240	0,0110	0,52	3,5200	P21/n	295	2000
XEFDAO	175,6	2,2400	2,2280	0,0120	0,54	3,5700	Cc	293	2006
XEKQOV	171,0	2,1510	2,1510	0,0000	0,00	3,4500	C2/c	296	2013
XICSEK	175,4	2,1500	2,1510	-1,0000e-3	-0,05	4,4300	P-1	293	2016
XIDCOF	170,3	2,1490	2,1610	-0,0120	-0,56	5,9300	P-1	293	2016
XIQPAO	178,0	2,1270	2,1320	-5,0000e-3	-0,23	3,4400	Cc	173	2001
XITNIZ	180,0	2,1570	2,1570	0,0000	0,00	6,4000	P21/c	294	2014
XOBSAI	172,8	2,1240	2,1250	-1,0000e-3	-0,05	5,0400	P21/a	293	2002
XOBSAI	171,4	2,1460	2,1290	0,0170	0,80	5,0400	P21/a	293	2002
XOLTAT	176,7	2,1480	2,1680	-0,0200	-0,92	4,9200	P21	293	2002
XOPREB	177,4	2,1560	2,1590	-3,0000e-3	-0,14	6,6000	P21/c	296	2014
XUJSAX	179,1	2,1490	2,2150	-0,0660	-2,98	4,7900	P1	298	2009
XUKXIL	174,7	2,1050	2,1090	-4,0000e-3	-0,19	5,0700	C2/c	150	2009
YARHOQ	173,7	2,1240	2,1260	-2,0000e-3	-0,09	2,5300	P-1	100	2012
YARHUW	173,5	2,1290	2,1300	-1,0000e-3	-0,05	2,6400	P-1	100	2012
YARJAE	172,1	2,1320	2,1220	0,0100	0,47	6,5400	P21/m	100	2012
YECTIK	173,7	2,1480	2,1530	-5,0000e-3	-0,23	5,2800	P-1	150	2006
YECTOQ	172,0	2,1650	2,1610	4,0000e-3	0,19	6,8200	P-1	150	2006
YEWGEP	180,0	2,1280	2,1280	0,0000	0,00	4,8300	P-1	200	2018
YIHYUL	176,0	2,1190	2,1240	-5,0000e-3	-0,24	3,3900	P-1	293	2013
YIHYUL01	176,0	2,1190	2,1240	-5,0000e-3	-0,24	3,3900	P-1	293	2014
YIQKUH	178,4	2,1850	2,1680	0,0170	0,78	6,9300	P21/n	298	2018
YIQLOB	178,9	2,1630	2,1330	0,0300	1,41	4,4700	C2/c	203	2010
YIQLUH	177,4	2,1520	2,1540	-2,0000e-3	-0,09	5,6200	P21/c	293	2010
YIRYIK	172,8	2,1710	2,1800	-9,0000e-3	-0,41	1,5500	P-1	110	2018
YIWJUK	171,2	2,1500	2,1500	0,0000	0,00	2,8300	Fddd	295	2008
YOFDUU	171,6	2,1350	2,1440	-9,0000e-3	-0,42	2,2900	P21/c	93	2014
YOTWEL	180,0	2,0100	2,0100	0,0000	0,00	7,8700	P-1	293	2014
YOTWIP	171,7	2,1620	2,1730	-0,0110	-0,51	4,5400	P-1	293	2014
YUCNIU	170,7	2,1590	2,1590	0,0000	0,00	3,2700	Cmc21	153	2009
YUDQUK	172,0	2,2280	2,2280	0,0000	0,00	4,0800	P2/c	291	2009
ZAXRUO	173,4	2,1530	2,1560	-3,0000e-3	-0,14	2,7400	P21/c	173	2016
ZEDFOG	174,7	2,1680	2,1630	5,0000e-3	0,23	8,8900	P21/a	93	2017
ZUJPUR	177,8	2,1280	2,1420	-0,0140	-0,65	7,4200	C2/c	294	2014
ZUJQAY	176,1	2,1040	2,1160	-0,0120	-0,57	7,7900	C2/c	294	2014

Table S19. N-Ag-N Bond angles and N-Ag bond distances in the 1740 related X-ray structures available in the CSD. Only close to linear (180° ± 10°), nonpolymeric structures not having additional metal coordination are considered.

Ref. code	Angle	N(1)-I (Å)	N(2)-I (Å)	Δ(N-I)	%	R	SpGr	Temp	Pub. Year
CUCTUS	173,6	2,0570	2,0940	11,5900	Cc	100	2020		
CUCTUS	174,3	2,0840	2,2150	11,5900	Cc	100	2020		
CUCTUS	174,3	2,1200	2,1150	11,5900	Cc	100	2020		
CUCTUS	174,8	2,1560	2,1100	11,5900	Cc	100	2020		
FOZMOZ	170,6	2,0690	2,1220	6,6300	P21	293	2019		
FOZMOZ	178,7	2,0740	2,1150	6,6300	P21	293	2019		
FOZMOZ	177,6	2,0870	2,0820	6,6300	P21	293	2019		
FOZMOZ	172,3	2,1280	2,1330	6,6300	P21	293	2019		
FOZMOZ	178,2	2,1110	2,1160	6,6300	P21	293	2019		
FOZMOZ	174,4	2,1160	2,1030	6,6300	P21	293	2019		
FOZMOZ	173,5	2,1170	2,0900	6,6300	P21	293	2019		
FOZMOZ	177,0	2,0770	2,0890	6,6300	P21	293	2019		
FOZMOZ	174,9	2,0850	2,0670	6,6300	P21	293	2019		
FOZMOZ	178,0	2,0990	2,1040	6,6300	P21	293	2019		
FOZMOZ	176,6	2,1380	2,1130	6,6300	P21	293	2019		
FOZMOZ01	178,8	2,0620	2,0710	7,0100	P21	100	2019		
FOZMOZ01	177,2	2,1030	2,1160	7,0100	P21	100	2019		
FOZMOZ01	178,4	2,1300	2,1220	7,0100	P21	100	2019		
FOZMOZ01	176,0	2,0940	2,0960	7,0100	P21	100	2019		
FOZMOZ01	173,7	2,1020	2,1060	7,0100	P21	100	2019		
FOZMOZ01	172,6	2,1250	2,1250	7,0100	P21	100	2019		
FOZMOZ01	176,8	2,1210	2,1370	7,0100	P21	100	2019		

FOZMOZ01	172,3	2,1370	2,1620	7,0100	P21	100	2019
FOZMOZ01	174,1	2,0660	2,0590	7,0100	P21	100	2019
FOZMOZ01	177,5	2,0930	2,1150	7,0100	P21	100	2019
FOZMOZ01	177,1	2,1170	2,1140	7,0100	P21	100	2019
FOZMOZ02	179,2	2,0820	2,1030	4,4000	P21	100	2019
FOZMOZ02	171,8	2,0920	2,0880	4,4000	P21	100	2019
FOZMOZ02	177,7	2,1060	2,0950	4,4000	P21	100	2019
FOZMOZ02	178,2	2,1220	2,0980	4,4000	P21	100	2019
FOZMOZ02	175,1	2,0720	2,0890	4,4000	P21	100	2019
FOZMOZ02	176,5	2,0990	2,0950	4,4000	P21	100	2019
FOZMOZ02	172,3	2,1220	2,1330	4,4000	P21	100	2019
FOZMOZ02	177,2	2,0850	2,0900	4,4000	P21	100	2019
FOZMOZ02	176,7	2,0880	2,1150	4,4000	P21	100	2019
FOZMOZ02	174,3	2,0910	2,0970	4,4000	P21	100	2019
FOZMOZ02	173,5	2,1090	2,1280	4,4000	P21	100	2019
FOZMOZ03	177,5	2,0820	2,0910	4,3600	P21	100	2019
FOZMOZ03	172,3	2,1200	2,1360	4,3600	P21	100	2019
FOZMOZ03	179,1	2,0860	2,0980	4,3600	P21	100	2019
FOZMOZ03	174,1	2,0950	2,1010	4,3600	P21	100	2019
FOZMOZ03	177,8	2,0960	2,1040	4,3600	P21	100	2019
FOZMOZ03	175,4	2,0760	2,0780	4,3600	P21	100	2019
FOZMOZ03	171,6	2,0820	2,1030	4,3600	P21	100	2019
FOZMOZ03	176,8	2,1130	2,0980	4,3600	P21	100	2019
FOZMOZ03	173,5	2,1140	2,1090	4,3600	P21	100	2019
FOZMOZ03	176,8	2,0950	2,1050	4,3600	P21	100	2019
FOZMOZ03	178,2	2,1240	2,1010	4,3600	P21	100	2019
FOZMOZ04	177,6	2,1030	2,0810	6,7500	P21	100	2019
FOZMOZ04	177,2	2,1290	2,1040	6,7500	P21	100	2019
FOZMOZ04	179,3	2,0750	2,0760	6,7500	P21	100	2019
FOZMOZ04	177,8	2,0860	2,1100	6,7500	P21	100	2019
FOZMOZ04	172,4	2,1200	2,0910	6,7500	P21	100	2019
FOZMOZ04	175,0	2,0910	2,0570	6,7500	P21	100	2019
FOZMOZ04	176,4	2,1080	2,1500	6,7500	P21	100	2019
FOZMOZ04	173,4	2,1360	2,1120	6,7500	P21	100	2019
FOZMOZ04	178,1	2,1430	2,0900	6,7500	P21	100	2019
FOZMOZ04	176,3	2,0720	2,1140	6,7500	P21	100	2019
FOZMOZ04	173,1	2,1440	2,1440	6,7500	P21	100	2019
FOZMOZ05	174,4	2,0980	2,1010	3,8900	P21	100	2019
FOZMOZ05	173,2	2,1090	2,1260	3,8900	P21	100	2019
FOZMOZ05	171,7	2,0680	2,0880	3,8900	P21	100	2019
FOZMOZ05	177,5	2,0850	2,1060	3,8900	P21	100	2019
FOZMOZ05	178,4	2,0800	2,0860	3,8900	P21	100	2019
FOZMOZ05	175,6	2,0820	2,0820	3,8900	P21	100	2019
FOZMOZ05	177,1	2,0950	2,0800	3,8900	P21	100	2019
FOZMOZ05	176,6	2,0950	2,0840	3,8900	P21	100	2019
FOZMOZ05	172,5	2,1300	2,1190	3,8900	P21	100	2019
FOZMOZ05	177,0	2,1010	2,1000	3,8900	P21	100	2019
FOZMOZ05	177,9	2,1080	2,0940	3,8900	P21	100	2019
JOXZOO	170,9	2,1370	2,1210	9,3500	P1	100	2019
JOXZOO	171,3	2,1020	2,1190	9,3500	P1	100	2019
KOSTIY	178,3	2,1100	2,1120	6,9700	P21/n	115	2019
KOSTIY	178,9	2,1160	2,1080	6,9700	P21/n	115	2019
KOXYEE	175,9	2,0800	2,0760	5,4500	P21/c	298	2019
KOXYEE	177,9	2,1030	2,1030	5,4500	P21/c	298	2019
KOXYEE	179,0	2,0810	2,0900	5,4500	P21/c	298	2019
KOXZAB	176,5	2,1100	2,1100	3,3000	C2/c	298	2019
MOXLIX	175,3	2,1100	2,1120	4,4000	P-1	220	2019
MOXLIX	174,8	2,0890	2,1000	4,4000	P-1	220	2019
MOXLIX	178,7	2,0980	2,0970	4,4000	P-1	220	2019
MOXLUJ	176,4	2,1350	2,1370	4,9900	P21/n	220	2019
MOXLUJ	176,5	2,0990	2,0940	4,9900	P21/n	220	2019
MOXLUJ	176,8	2,0970	2,0930	4,9900	P21/n	220	2019
MOXMAQ	177,8	2,1100	2,0980	2,3700	C2/c	220	2019
MOXMAQ	178,5	2,0910	2,0910	2,3700	C2/c	220	2019
ROWCAK	175,5	2,1040	2,1310	8,6900	P-1	250	2019
ROYKUO	178,5	2,0770	2,0700	4,6300	P-1	100	2016
ROYKUO	179,6	2,0690	2,0770	4,6300	P-1	100	2016
ROYKUO	178,0	2,0660	2,0740	4,6300	P-1	100	2016
ROYSAC	173,8	2,1080	2,1610	15,1100	I432	93	2019
TOSJIX	178,0	2,1010	2,0990	6,4300	P21/c	298	2019
TOSJIX	174,9	2,1000	2,1000	6,4300	P21/c	298	2019
TOSJIX	174,2	2,0960	2,0890	6,4300	P21/c	298	2019
TOSJUJ	176,9	2,1190	2,0760	4,4300	P1	100	2019
TOSJUJ	178,1	2,1250	2,0850	4,4300	P1	100	2019
TOSJUJ	177,1	2,1360	2,0850	4,4300	P1	100	2019
TOSJUJ	176,4	2,0810	2,1110	4,4300	P1	100	2019
TOSJUJ	175,6	2,1000	2,1090	4,4300	P1	100	2019
TOSJUJ	176,0	2,1010	2,0980	4,4300	P1	100	2019

TOSJUJ	177,6	2,0590	2,0800	4,4300	P1	100	2019
TOSJUJ	177,6	2,1030	2,1270	4,4300	P1	100	2019
TOSJUJ	178,2	2,1080	2,0790	4,4300	P1	100	2019
TOSKAQ	171,4	2,0850	2,0980	5,5100	P21/c	298	2019
TOSKAQ	177,7	2,0950	2,0930	5,5100	P21/c	298	2019
TOSKAQ	178,6	2,0840	2,0830	5,5100	P21/c	298	2019
TOSKAQ	174,6	2,0880	2,0800	5,5100	P21/c	298	2019
TOSKAQ	174,8	2,0990	2,0980	5,5100	P21/c	298	2019
VUCSUK	177,9	2,1520	2,1520	4,5900	C2/c	150	2020
WOWZOA	176,8	2,0910	2,0900	3,6600	P21/n	120	2019
ZUGKOE	179,7	2,1270	2,1290	3,6200	P21/c	93	2020
ZUGZOT	176,0	2,1340	2,1470	2,7200	P-1	180	2020
ZUGZUZ	176,4	2,1270	2,1290	2,5700	C2/c	180	2020
ABEMUR	171,8	2,1390	2,1370	6,5200	P-1	293	2011
ABOREQ	172,2	2,1500	2,1470	2,5100	P-1	296	2011
ACAKUN	173,5	2,1750	2,1760	6,6000	P21/n	301	2017
ACALEY	174,9	2,1810	2,1760	6,4100	P21/n	301	2017
ACUREV	173,9	2,1560	2,1550	2,6200	P-1	293	2001
ADAHOD	174,7	2,0850	2,0780	4,2000	P21/c	294	2006
ADIXUH	171,6	2,0350	2,0440	8,6400	Pbcn	293	2007
ADIXUH	171,2	2,0620	2,0620	8,6400	Pbcn	293	2007
ADIXUH	170,2	2,0930	2,0910	8,6400	Pbcn	293	2007
ADIYAO	180,0	2,1160	2,1160	6,8900	P21/n	293	2007
ADIYAO	170,6	2,1210	2,1000	6,8900	P21/n	293	2007
ADIYAO	170,5	2,0880	2,1040	6,8900	P21/n	293	2007
ADUHUF	180,0	2,0570	2,0570	7,1500	P-1	293	2016
ADUHUF	180,0	2,1140	2,1140	7,1500	P-1	293	2016
ADUJAN	180,0	2,1150	2,1150	7,0300	P-1	293	2016
ADUJAN	176,0	2,1170	2,0970	7,0300	P-1	293	2016
AFATOQ	175,5	2,1180	2,1190	4,0000	P-1	213	2002
AFATOQ	174,4	2,1280	2,1290	4,0000	P-1	213	2002
AGEHOJ	174,1	2,0880	2,1060	4,3800	P21/c	193	2002
AGEHOJ	170,5	2,1770	2,1830	4,3800	P21/c	193	2002
AGEHOJ	171,0	2,1700	2,1960	4,3800	P21/c	193	2002
AGEHUP	171,0	2,1440	2,1610	4,1600	Pca21	293	2002
AGEHUP	171,7	2,1520	2,1700	4,1600	Pca21	293	2002
AGEHUP	173,5	2,1030	2,1130	4,1600	Pca21	293	2002
AGEHUP	170,5	2,1420	2,1420	4,1600	Pca21	293	2002
AGEHUP	173,5	2,0970	2,0910	4,1600	Pca21	293	2002
AGEHUP	171,2	2,1480	2,1490	4,1600	Pca21	293	2002
AGEJAX	172,7	2,1120	2,1020	7,2100	P21/c	173	2002
AGEJAX	171,8	2,1850	2,1640	7,2100	P21/c	173	2002
AGIMHN01	172,1	2,1180	2,1320	3,9000	P212121	295	1971
AGIMHN02	172,8	2,1150	2,1200	2,1000	P212121	150	2006
AGIMHN03	172,6	2,1120	2,1160	2,3000	P212121	298	2007
AGMTHY	180,0	2,0810	2,0810	2,8000	C2/c	295	1979
AGPYNO	172,7	2,1620	2,1620	11,8000	C2/c	295	1970
AGPYNO02	173,9	2,1760	2,1760	1,7900	I2/a	173	2015
AHIDIE	180,0	2,1730	2,1730	4,8100	C2/c	223	2002
AHIDIE	173,1	2,1910	2,1940	4,8100	C2/c	223	2002
AHUCIR	174,9	2,0850	2,0750	3,2800	P-1	133	2015
AHUCIR	177,1	2,1250	2,1290	3,2800	P-1	133	2015
AHUCIR	178,3	2,0950	2,0850	3,2800	P-1	133	2015
AHUCIR	173,9	2,0770	2,0830	3,2800	P-1	133	2015
AHUCIR	176,5	2,0860	2,0880	3,2800	P-1	133	2015
AJIBUR	178,3	2,0900	2,0910	2,4300	P21/n	295	2009
AJUKOH	180,0	2,1800	2,1800	3,8900	P-1	298	2014
AKEDAX	177,3	2,1900	2,1800	2,9000	P-1	110	2016
AKUCEO	180,0	2,1930	2,1930	5,0000	C2/c	193	2003
AKUGIW	173,1	2,0960	2,0860	4,9900	P21/n	200	2003
ALIROC	177,2	2,0990	2,0990	6,3400	P321	200	2003
ALIRUI	173,8	2,1040	2,1040	7,2200	Cmcm	293	2003
ALIRUI	173,7	2,0260	2,0260	7,2200	Cmcm	293	2003
AMOVIJ	180,0	2,1480	2,1480	5,3600	Pbam	296	2016
APIRAU	173,6	2,1070	2,1030	2,9700	P21/n	100	2012
AQAVOD	173,4	2,1450	2,1510	2,8200	P21/m	293	2004
AQUZIX	179,7	2,0810	2,0770	5,7700	P-1	296	2016
ARECOQ	180,0	2,1420	2,1420	3,3000	P21/n	293	2010
ASAVAS	173,4	2,1670	2,1750	4,5400	Pn	133	2011
ASIQOK	179,4	2,1360	2,1390	4,4400	P-1	293	2016
ASIQUQ	176,7	2,1220	2,1140	4,4800	C2/c	293	2016
ASOWIO	180,0	2,1240	2,1240	3,7000	P-1	298	2004
ATEHOY	180,0	2,1490	2,1470	7,2400	C2/c	293	2016
ATEHOY	180,0	2,1850	2,1730	7,2400	C2/c	293	2016
ATOLEA	173,5	2,1140	2,1180	3,3400	P21/n	293	2003
ATOXOX	176,8	2,1030	2,0880	4,2400	P21/a	100	2011
ATOXOX	173,6	2,0930	2,0820	4,2400	P21/a	100	2011
ATOXUD	172,2	2,1230	2,1230	9,9100	P6/m	113	2011

ATOXUD	170,5	2,1410	2,1410	9,9100	P6/m	113	2011
ATOXUD	172,3	2,1650	2,1650	9,9100	P6/m	113	2011
ATOYAK	174,2	2,1190	2,1250	9,2300	P21/m	293	2011
ATOYAK	172,9	2,1300	2,1520	9,2300	P21/m	293	2011
AWAWUS	172,4	2,0650	2,0810	3,8700	Fd-3c	100	2016
AWUTAP	179,0	2,1750	2,1610	14,2100	P-1	150	2016
AXUDUS	172,5	2,1070	2,1140	5,8400	P21/n	200	2004
AYICEQ	174,6	2,1570	2,1500	3,7900	P21/n	150	2004
AYICIU	179,6	2,1040	2,1160	5,9300	P1	150	2004
AYICOA	180,0	2,1420	2,1420	7,2000	P21/n	150	2004
AYOGUQ	180,0	2,1250	2,1250	2,9400	P-1	293	2004
AYOGUQ	180,0	2,1320	2,1320	2,9400	P-1	293	2004
AYOHAX	180,0	2,1340	2,1340	3,0500	P21/c	293	2004
AYUXUN	171,0	2,1150	2,1130	7,2900	C2/c	293	2004
BACROP	173,2	2,0550	2,0540	2,3100	C2/c	293	2016
BACROP	174,0	2,0490	2,0490	2,3100	C2/c	293	2016
BADVAG	180,0	2,1220	2,1220	5,4200	P-1	293	2015
BAFBES	180,0	2,1010	2,1010	3,6100	P-1	293	2015
BAFDIW	171,8	2,1050	2,1050	5,1000	C2/c	200	2002
BAFDIW	173,1	2,1460	2,1520	5,1000	C2/c	200	2002
BAFDIW	171,1	2,1120	2,1040	5,1000	C2/c	200	2002
BAFDIW	171,2	2,0890	2,0770	5,1000	C2/c	200	2002
BAFDIW	172,4	2,1010	2,1040	5,1000	C2/c	200	2002
BAFDOC	170,1	2,1640	2,1660	7,4200	P21/n	200	2002
BAFDOC	173,0	2,1260	2,1170	7,4200	P21/n	200	2002
BALHIH	180,0	2,1100	2,1100	3,9200	C2/c	173	2011
BALJAB	180,0	2,1380	2,1380	3,2200	C2/c	173	2011
BARJEM	171,5	2,0970	2,1020	9,7400	P21/c	293	2017
BARJEM	175,2	2,0310	2,0750	9,7400	P21/c	293	2017
BARJEM	170,4	2,1250	2,1240	9,7400	P21/c	293	2017
BARJEM	172,7	2,0780	2,1430	9,7400	P21/c	293	2017
BAXLET	171,5	2,0700	2,0770	6,1400	C2/c	123	2012
BAXLET	176,2	2,0550	2,0660	6,1400	C2/c	123	2012
BAXLET	170,8	2,0880	2,0920	6,1400	C2/c	123	2012
BAXLET	176,6	2,0700	2,0620	6,1400	C2/c	123	2012
BESLES	180,0	2,0700	2,0700	7,2300	P-1	150	2013
BESLES	180,0	2,1090	2,1090	7,2300	P-1	150	2013
BEWVOR	180,0	2,1320	2,1320	6,1600	P-1	296	2018
BEWWUW	171,1	2,1740	2,1520	4,6900	R-3	293	2004
BIPFOX	170,9	2,1470	2,1800	3,6600	P-1	293	2014
BIPGAK	170,3	2,1760	2,1520	3,2500	P-1	293	2014
BIQVUU	180,0	2,1410	2,1410	3,3300	P21/c	293	2013
BIRVOQ	174,8	2,0440	2,0390	4,9600	P21/c	150	2018
BISTUU	171,8	2,1620	2,1680	3,4700	P-1	150	2014
BIXHAU	180,0	2,1410	2,1410	12,9200	P-1	93	2018
BOBKIP	172,9	2,1270	2,1340	4,8700	P21/c	150	2019
BOBKOV	180,0	2,1380	2,1380	3,2400	P21/n	150	2019
BOCFIL	173,3	2,1300	2,1530	6,6100	C2/c	100	2019
BOCQEQ	176,5	2,0870	2,1160	6,3900	P-1	293	2008
BOCQEQ	177,6	2,0970	2,0990	6,3900	P-1	293	2008
BOCQEQ	177,6	2,1180	2,0790	6,3900	P-1	293	2008
BOCQIU	177,9	2,1250	2,1140	6,5800	P63/m	293	2008
BOQVEK	180,0	2,1770	2,1770	12,7700	P21/c	173	2014
BOSNON	173,6	2,0930	2,0980	2,4300	P21/n	100	2009
BOSNON	171,8	2,0980	2,1010	2,4300	P21/n	100	2009
BOSNON	176,4	2,1170	2,1190	2,4300	P21/n	100	2009
BOTQAD	178,6	2,1920	2,2080	2,8500	P-1	153	2009
BUDDUA	172,6	2,1290	2,1440	4,0300	Pbc21	120	2009
BUDDUA	173,9	2,1300	2,1750	4,0300	Pbc21	120	2009
BURVER	170,8	2,1310	2,1270	2,8000	C2/c	100	2015
BUTGAZ	177,8	2,1240	2,1340	5,0800	P21/c	273	2009
CABMAU	170,2	2,1200	2,1200	6,5600	P-3c1	293	2001
CABMUO	180,0	2,1740	2,1740	6,9100	P-1	293	2001
CACDIV	175,2	2,0710	2,0810	5,9800	P21/n	293	2010
CAGWUF	175,8	2,1510	2,1330	11,1900	Pnma	298	2017
CAGWUF	173,0	2,1370	2,1370	11,1900	Pnma	298	2017
CAGWUF	178,3	2,1570	2,1570	11,1900	Pnma	298	2017
CAGWUF	171,3	2,1460	2,1460	11,1900	Pnma	298	2017
CAGXAM	179,1	2,1060	2,1060	9,7000	Pnna	298	2017
CAHMUW	176,4	2,2450	2,1980	12,7300	Pbcn	100	2016
CAHMUW	177,0	2,2510	2,1210	12,7300	Pbcn	100	2016
CAHMUW	174,8	2,0920	2,1500	12,7300	Pbcn	100	2016
CAHMUW	172,3	2,1450	2,1130	12,7300	Pbcn	100	2016
CALHUT	175,1	2,1280	2,1240	5,5100	P-1	173	2002
CALJAB	172,7	2,1320	2,1300	4,5800	P-1	173	2002
CALJEF	177,8	2,1360	2,1350	6,0200	P-1	173	2002
CANTOB	176,9	2,1390	2,1390	5,5300	C2221	293	2004
CANTOB	173,6	2,1760	2,1690	5,5300	C2221	293	2004

CASQAO	175,0	2,1470	2,1380	5,7000	P-1	295	1999	
CASXAW	180,0	2,1540	2,1540	4,9100	P-1	293	2005	
CAWNAS	173,2	2,0420	2,0580	9,2500	P-1	80	2017	
CAWNAS	177,0	2,0480	2,0490	9,2500	P-1	80	2017	
CAWNAS	173,2	2,0530	2,0240	9,2500	P-1	80	2017	
CAWNIA	177,7	2,0540	2,0510	3,4700	P-1	100	2017	
CAWNIA	175,8	2,0430	2,0430	3,4700	P-1	100	2017	
CAWNIA	171,6	2,0550	2,0420	3,4700	P-1	100	2017	
CAWNIA	174,5	2,0500	2,0450	3,4700	P-1	100	2017	
CAWNOG	177,1	2,0570	2,0520	4,0200	P-1	100	2017	
CAWNOG	177,5	2,0420	2,0450	4,0200	P-1	100	2017	
CAWNOG	177,7	2,0480	2,0460	4,0200	P-1	100	2017	
CAWNUM	175,5	2,0510	2,0520	4,7800	P-1	100	2017	
CAWNUM	176,2	2,0370	2,0400	4,7800	P-1	100	2017	
CAWNUM	173,5	2,0450	2,0490	4,7800	P-1	100	2017	
CAWPAU	170,1	2,0930	2,0410	8,6100	P-1	100	2017	
CAWPAU	175,2	2,0360	2,0680	8,6100	P-1	100	2017	
CEGDUO	172,4	2,0820	2,0820	2,4900	Pnna	123	2006	
CEGDUO	176,2	2,0710	2,0750	2,4900	Pnna	123	2006	
CEGFAW	175,0	2,0690	2,0760	4,2300	P21/c	123	2006	
CEGFAW	176,8	2,0820	2,0920	4,2300	P21/c	123	2006	
CEGFAW	173,5	2,0940	2,0890	4,2300	P21/c	123	2006	
CEGFEA	180,0	2,0670	2,0670	2,6000	P21/n	293	2006	
CEGFEA02	180,0	2,0670	2,0670	3,0200	P21/n	300	2012	
CEGFEA03	180,0	2,0680	2,0680	2,8400	P21/n	250	2012	
CEGFEA04	180,0	2,0740	2,0740	2,5300	P21/n	200	2012	
CEGFEA05	180,0	2,0680	2,0680	2,5600	P21/n	150	2012	
CEGFEA06	180,0	2,0670	2,0670	2,5100	P21/n	100	2012	
CEGFEA07	180,0	2,0720	2,0720	2,4800	P21/n	293	2012	
CEGFEA08	180,0	2,0620	2,0620	8,2100	P21/n	293	2012	
CEGFEA09	180,0	2,0500	2,0500	8,9100	P21/n	293	2012	
CEGFEA10	180,0	2,0490	2,0490	7,2800	P21/n	293	2012	
CEGFEA11	180,0	2,0440	2,0440	7,7200	P21/n	293	2012	
CEGFEA12	180,0	2,0480	2,0480	7,5100	P21/n	293	2012	
CEGFEA13	180,0	2,0430	2,0430	6,0100	P21/n	293	2012	
CEGFEA14	180,0	2,0370	2,0370	6,1500	P21/n	293	2012	
CEJDED	171,9	2,1210	2,1130	4,9900	P-1	123	2017	
CENFIM	176,5	2,0960	2,0950	5,7700	P-1	298	2013	
CENFIM	178,4	2,1160	2,0970	5,7700	P-1	298	2013	
CENFIM	177,2	2,1470	2,1460	5,7700	P-1	298	2013	
CENFOS	171,7	2,1380	2,1160	5,2600	P-1	298	2013	
CENFOS	176,7	2,0980	2,1300	5,2600	P-1	298	2013	
CENFOS	172,3	2,1160	2,1330	5,2600	P-1	298	2013	
CENGAF	176,8	2,1170	2,1220	5,9500	P21	298	2013	
CENGAF	170,1	2,1320	2,1230	5,9500	P21	298	2013	
CENGAF	176,0	2,0540	2,0850	5,9500	P21	298	2013	
CENGAF	178,4	2,1160	2,1100	5,9500	P21	298	2013	
CENGAF	176,3	2,1100	2,1230	5,9500	P21	298	2013	
CENGAF	176,3	2,1210	2,0940	5,9500	P21	298	2013	
CENGAF01	177,3	2,0770	2,0830	3,9500	P21	203	2016	
CENGAF01	175,7	2,1050	2,0980	3,9500	P21	203	2016	
CENGAF01	176,1	2,0870	2,0760	3,9500	P21	203	2016	
CENGAF01	175,6	2,0930	2,1160	3,9500	P21	203	2016	
CENGAF01	178,1	2,0840	2,0790	3,9500	P21	203	2016	
CENGAF01	170,6	2,1120	2,1040	3,9500	P21	203	2016	
CENGEJ	177,7	2,0900	2,0900	4,4000	P-1	298	2013	
CENGEJ	177,2	2,0910	2,0810	4,4000	P-1	298	2013	
CENGEJ	179,7	2,0920	2,1050	4,4000	P-1	298	2013	
CENGIN	176,3	2,0850	2,0750	5,2900	P-1	298	2013	
CENGIN	178,8	2,1140	2,0840	5,2900	P-1	298	2013	
CENGIN	175,5	2,0610	2,0920	5,2900	P-1	298	2013	
CENGIN	177,1	2,0860	2,0730	5,2900	P-1	298	2013	
CENGIN	177,8	2,0780	2,0960	5,2900	P-1	298	2013	
CENGIN	174,5	2,0850	2,0940	5,2900	P-1	298	2013	
CENGOT	178,5	2,1260	2,1210	3,4900	P-1	298	2013	
CEPGOT	178,5	2,2260	2,2110	9,7300	Pcna	295	1999	
CETJER	175,7	2,1500	2,1390	4,4400	P212121		293	2005
CETJER	172,9	2,1180	2,1190	4,4400	P212121		293	2005
CEYMIC	179,2	2,0640	2,0730	3,0000	Cmc21		295	1984
CEYMIC	179,3	2,0760	2,0850	3,0000	Cmc21		295	1984
CEZBUE	175,1	2,1470	2,1540	6,6000	P-1	295	1984	
CEZGAP	180,0	2,0870	2,0870	9,7000	C2/c	295	1984	
CEZPOO	175,9	2,1310	2,1330	6,3900	P-1	295	2013	
CEZPUU	172,6	2,1530	2,1590	6,3700	C2/c	295	2013	
CIPDEM	173,7	2,1240	2,1230	4,5300	P-1	293	2014	
CIPDEM	180,0	2,1010	2,1010	4,5300	P-1	293	2014	
CIZNIK	172,2	2,0900	2,0900	8,0200	P-1	296	2014	
CIZNIK	170,2	2,0650	2,1140	8,0200	P-1	296	2014	

COFQAR	175,8	2,0780	2,0770	3,5600	P-1	298	2014
COFQAR	173,8	2,0920	2,0960	3,5600	P-1	298	2014
COFQAR	173,7	2,0850	2,0910	3,5600	P-1	298	2014
COFQEV	175,9	2,0820	2,0900	6,3100	Pna21	298	2014
COFQEV	175,6	2,0960	2,1130	6,3100	Pna21	298	2014
COFQEV	177,7	2,0850	2,0840	6,3100	Pna21	298	2014
COFQEV01	177,2	2,0870	2,0900	3,9000	Pna21	201	2014
COFQEV01	177,0	2,0940	2,1020	3,9000	Pna21	201	2014
COFQEV01	176,3	2,0890	2,0900	3,9000	Pna21	201	2014
COFQIZ	174,8	2,1020	2,1070	6,1000	Pna21	298	2014
COFQIZ	175,4	2,0870	2,0820	6,1000	Pna21	298	2014
COFQIZ	178,1	2,0900	2,0880	6,1000	Pna21	298	2014
COFQOF	174,8	2,0880	2,0900	6,1500	P21/c	298	2014
COFQOF	174,7	2,0750	2,0790	6,1500	P21/c	298	2014
COFQUL	175,9	2,0850	2,0770	3,7000	P-1	298	2014
COFQUL	174,6	2,0670	2,0720	3,7000	P-1	298	2014
COFQUL	172,3	2,0680	2,0590	3,7000	P-1	298	2014
COFQUL	174,0	2,0820	2,0920	3,7000	P-1	298	2014
COFQUL	175,9	2,0890	2,0870	3,7000	P-1	298	2014
COJFUD	174,3	2,1330	2,1490	13,4500	P-1	93	2008
COJFUD	177,0	2,1350	2,1220	13,4500	P-1	93	2008
COJFUD	174,2	2,0930	2,1130	13,4500	P-1	93	2008
COJFUD	171,9	2,1370	2,1220	13,4500	P-1	93	2008
COJFUD	174,3	2,1340	2,1410	13,4500	P-1	93	2008
COJFUD	174,3	2,1320	2,1330	13,4500	P-1	93	2008
COJFUD	170,2	2,1190	2,1190	13,4500	P-1	93	2008
COJFUD	171,6	2,0670	2,0720	13,4500	P-1	93	2008
COJPUP	176,1	2,1280	2,1240	4,7900	P21/c	293	2019
CONQEC	178,5	2,1590	2,2000	7,0800	P1	293	2009
CONQIG	177,7	2,1510	2,2050	4,7000	P1	293	2009
COPVAH	175,5	2,0980	2,1290	6,7300	P-1	293	2019
CORRAE	180,0	2,1940	2,1940	9,7700	P21/c	293	2014
COTBOC	180,0	2,1060	2,1060	2,7000	C2/c	295	1984
COVBEU	178,5	2,0880	2,0870	2,6000	Pnma	295	1984
COXBAU	180,0	2,0480	2,0480	5,2300	C2/c	298	2014
COXBEY	180,0	2,0500	2,0500	6,1500	C2/c	298	2014
COXBIC	180,0	2,0930	2,0930	5,6100	P-1	293	2014
COXBIC	175,3	2,0690	2,0710	5,6100	P-1	293	2014
CRETAG	177,2	2,1000	2,1000	3,4000	P2/c	295	1981
CUTNEL	176,1	2,1340	2,1410	2,7400	P-1	296	2010
CUWNIS	178,9	2,1330	2,1400	5,8000	P21/c	223	2010
CUWNIS	173,4	2,1440	2,1440	5,8000	P21/c	223	2010
CUWNOY	174,7	2,1790	2,1910	4,2000	P-1	223	2010
CUXRES	174,4	2,1220	2,1220	3,3100	C2/c	295	2000
CUXRES01	173,4	2,1130	2,1370	5,4600	C2/c	173	2006
CUXRES01	173,1	2,1250	2,1250	5,4600	C2/c	173	2006
CUYXIE	176,8	2,1500	2,1670	3,6600	P21/c	298	2010
CUZFIO	172,9	2,0960	2,0920	1,6900	P-1	100	2016
CUZKEP	176,3	2,1100	2,1120	2,4700	P-1	120	2016
CUZKIT	174,0	2,1170	2,1290	3,4100	Pbca	120	2016
DACKAV	176,3	2,1230	2,1280	3,9000	P21/c	296	2011
DACKAV	177,8	2,1280	2,1280	3,9000	P21/c	296	2011
DACYEO	179,4	2,0710	2,0710	2,5100	Pn	130	2017
DACYEO	173,8	2,0760	2,0680	2,5100	Pn	130	2017
DAPZEA	175,7	2,1390	2,1150	6,0200	P21/n	293	2005
DATJOZ	180,0	2,1790	2,1790	4,4300	P-1	296	2012
DATJOZ	174,3	2,1610	2,1590	4,4300	P-1	296	2012
DAWMIY	178,6	2,0890	2,0890	2,5700	C2/m	100	2005
DAXHIU	171,9	2,1580	2,1580	3,0600	C2/c	273	2005
DAXTAY	170,1	2,0940	2,0980	3,6400	P-1	293	2005
DAXTEC	176,3	2,0760	2,0880	3,9700	P-1	293	2005
DAXTIG	174,3	2,1090	2,1160	4,2500	P-1	298	2005
DAXTOM	174,7	2,0980	2,0980	3,4800	P-1	298	2005
DAXZEK	171,0	2,1260	2,1320	7,5900	P-1	293	2016
DAXZIO	171,8	2,1250	2,1220	8,9000	P-1	293	2016
DAYZIM	180,0	2,1670	2,1670	4,1000	I41/acd	295	1985
DAZFIU	175,5	2,0890	2,0890	2,6700	C2/m	100	2005
DAZGIV	177,7	2,0850	2,0810	2,8100	P-1	100	2005
DAZGIV	174,7	2,0950	2,0940	2,8100	P-1	100	2005
DAZGIV	172,0	2,0960	2,0960	2,8100	P-1	100	2005
DEBGOI	179,2	2,1380	2,1360	9,2400	P21/c	293	2012
DEBGOI	179,2	2,1220	2,1520	9,2400	P21/c	293	2012
DEHDIE	178,8	2,0970	2,0930	1,9300	P-1	150	2006
DEHDOK	180,0	2,1260	2,1260	2,1800	Pnma	150	2006
DEHZUO	180,0	2,1050	2,1050	7,4100	P-1	296	2017
DELJIP	180,0	2,1440	2,1440	9,7200	P-1	293	2009
DELJIP	180,0	2,1640	2,1640	9,7200	P-1	293	2009
DEWLUP	178,2	2,1590	2,1650	5,1700	P-1	293	2018

DEWLUP	180,0	2,1470	2,1470	5,1700	P-1	293	2018
DEWTIJ	174,8	2,1440	2,1360	2,2300	P21/c	100	2007
DEZMUS	180,0	2,1070	2,1070	6,4100	P21/c	298	2013
DICFEB	175,4	2,1040	2,1010	2,6700	P21/c	120	2007
DICFEB	176,0	2,1070	2,1060	2,6700	P21/c	120	2007
DICFEB	176,3	2,0990	2,1000	2,6700	P21/c	120	2007
DICFIF	176,3	2,0810	2,0870	2,2300	C2/c	100	2007
DICFIF	176,9	2,1000	2,0950	2,2300	C2/c	100	2007
DICFIF	178,0	2,1090	2,1140	2,2300	C2/c	100	2007
DICFOL	177,0	2,0950	2,0950	2,7100	Pnma	100	2007
DICFOL	177,8	2,0980	2,0960	2,7100	Pnma	100	2007
DICFUR	175,7	2,0930	2,0950	1,6600	R3c	100	2007
DICQAJ	177,4	2,0950	2,0950	6,8800	C2/c	153	2012
DICQAJ	174,7	2,0980	2,0980	6,8800	C2/c	153	2012
DICQEN	171,5	2,1020	2,1020	11,5500	C2/c	153	2012
DICQEN	179,4	2,0990	2,0990	11,5500	C2/c	153	2012
DIDZOH	170,9	2,1410	2,1220	3,4500	P-1	293	2012
DIMJOY	173,1	2,1120	2,1120	5,3000	P21/n	203	1999
DITCEO	173,7	2,1570	2,1490	3,3000	I-4	295	1985
DOHKIX	180,0	2,2230	2,2230	3,4600	P-1	296	2019
DOHXOP	170,3	2,1720	2,1690	5,9000	P21/n	293	2014
DOHXUV	172,6	2,1170	2,1270	4,1700	C2/c	100	2014
DOJCOW	179,2	2,0920	2,0780	3,0600	Pna21	190	2014
DOJCOW	173,4	2,0640	2,0720	3,0600	Pna21	190	2014
DOJCUC	172,8	2,0770	2,0610	5,1700	P21/c	190	2014
DOKJOD	180,0	2,0950	2,0950	7,5400	P21/n	123	2008
DOKJOD	180,0	2,1290	2,1290	7,5400	P21/n	123	2008
DONMIF	174,3	2,1220	2,1310	3,8500	P-1	100	2019
DONMIF	174,3	2,1320	2,1320	3,8500	P-1	100	2019
DOQHEY	174,8	2,1540	2,1680	5,2900	P21/n	173	2015
DUCGOY	180,0	2,1230	2,1230	2,7300	P-1	293	2009
DUHHIY	172,6	2,1690	2,1590	6,0300	P-1	173	2009
DUPMIL	180,0	2,0900	2,0900	2,9300	P-1	293	2010
DUPMIL	180,0	2,0950	2,0950	2,9300	P-1	293	2010
DUPMIL	175,9	2,1040	2,1060	2,9300	P-1	293	2010
DUQTOZ	173,4	2,1460	2,1520	2,5600	P-1	296	2010
EBAHIA	179,0	2,1150	2,1260	6,3800	R-3	100	2011
EBAJAV	170,3	2,1480	2,1600	2,9000	P-1	173	2016
EBISIT	180,0	2,0890	2,0890	6,0100	P-1	293	2011
EBITOZ	175,3	2,1020	2,1020	3,6200	P21/c	200	2004
ECALIG	173,5	2,1510	2,1520	2,0200	P-1	133	2016
ECENUW	176,8	2,1640	2,1630	4,0600	Cc	296	2006
ECEPAE	177,6	2,1680	2,1700	3,8200	Cc	223	2006
ECEPEI	172,9	2,1730	2,1670	8,0700	P21/n	296	2006
ECEPEI	170,4	2,1310	2,1470	8,0700	P21/n	296	2006
ECOYOM	178,0	2,0990	2,1120	4,6200	P-1	293	2012
ECOYOM	180,0	2,1780	2,1780	4,6200	P-1	293	2012
EDEREK	173,5	2,1160	2,1190	5,0600	P21/n	293	2000
EFAWOY	173,2	2,1100	2,1020	3,8300	P-1	100	2008
EFAZUH	171,0	2,0900	2,0830	4,4100	C2/c	296	2008
EFAZUH	172,4	2,0830	2,0790	4,4100	C2/c	296	2008
EFIWUL	171,4	2,1960	2,1990	4,2500	P21/c	298	2002
EHIHEK	171,0	2,1420	2,1400	6,2700	P-1	293	2016
EHIHIO	170,4	2,1320	2,1370	5,0200	P-1	293	2016
EJIMUG	173,0	2,2280	2,2000	6,7800	C2/c	296	2011
EKOSOM	176,5	2,1240	2,1240	8,5500	P4/mnc	173	2003
EKOVAB	172,4	2,1130	2,0980	6,4300	P42/n	294	2003
EKOVAB	176,1	2,1170	2,1140	6,4300	P42/n	294	2003
ELAVET	170,9	2,1220	2,1380	6,7800	P-1	296	2011
ELAVET	170,8	2,1430	2,1390	6,7800	P-1	296	2011
ELEVEW	180,0	2,1340	2,1340	3,4500	P21/c	200	2003
ELIQOG	174,6	2,1070	2,1160	2,1700	P21/n	120	2010
EMAHEG	180,0	2,0750	2,0750	4,9000	P-1	293	2011
EMEJOW	177,3	2,1190	2,1270	5,8300	P-1	295	2010
EMEJOW	172,3	2,1360	2,1340	5,8300	P-1	295	2010
EMOVIM	173,9	2,1630	2,1620	3,8800	P-1	173	2011
ENETAU	170,6	2,1530	2,1260	4,8600	P21/n	296	2016
ENEYEC	180,0	2,1770	2,1770	8,4400	P-1	173	2010
ENEYOM	180,0	2,1370	2,1370	4,2600	P21/n	173	2010
EPUSUE	174,5	2,1110	2,1550	8,7400	P-1	293	2011
EPUSUE	177,8	2,0630	2,0830	8,7400	P-1	293	2011
EPUSUE	173,5	2,0870	2,1130	8,7400	P-1	293	2011
EPUSUE	175,4	2,1230	2,0980	8,7400	P-1	293	2011
EPUTAL	175,6	2,0860	2,1550	9,5100	P21/c	293	2011
EPUTAL	173,8	2,0600	2,0720	9,5100	P21/c	293	2011
EPUTAL	177,7	2,0960	2,0990	9,5100	P21/c	293	2011
EPUTEP	174,8	2,0700	2,0680	3,0400	P21/n	123	2011
EPUTEP	178,2	2,0740	2,0710	3,0400	P21/n	123	2011

EPUTEP	175,8	2,0770	2,0760	3,0400	P21/n	123	2011
EPUTIT	176,9	2,0770	2,0710	5,4700	P-1	293	2011
EPUTIT	178,1	2,0740	2,0650	5,4700	P-1	293	2011
EPUTIT	172,3	2,0690	2,0750	5,4700	P-1	293	2011
EPUTIT	175,8	2,0840	2,0820	5,4700	P-1	293	2011
EPUTOZ	173,8	2,0960	2,0630	7,5900	Pna21	293	2011
EPUTOZ	175,9	2,1020	2,0610	7,5900	Pna21	293	2011
EPUTOZ	170,7	2,1060	2,0800	7,5900	Pna21	293	2011
EPUTOZ	170,3	2,0720	2,0580	7,5900	Pna21	293	2011
EPUTOZ	176,8	2,1430	2,0420	7,5900	Pna21	293	2011
EPUTOZ	178,5	2,1130	2,0920	7,5900	Pna21	293	2011
EPUTOZ	172,5	2,1110	2,0910	7,5900	Pna21	293	2011
EPUTOZ	176,8	2,0920	2,1310	7,5900	Pna21	293	2011
EPUTOZ	173,1	2,0940	2,1040	7,5900	Pna21	293	2011
ERIKEW	177,0	2,0780	2,0790	2,9000	P-1	293	2011
ESARUL	175,4	2,1360	2,1470	4,1700	P21/n	293	2004
ESEMEU	177,5	2,0970	2,0930	4,3600	P21/n	293	2004
ESETOL	180,0	2,1620	2,1620	3,3100	P-1	293	2004
ESEYIK	171,7	2,1600	2,1770	8,6300	C2/c	298	2004
ESEYIK01	171,1	2,1560	2,1420	5,0500	P-1	298	2005
ESEYIK01	173,3	2,1630	2,1760	5,0500	P-1	298	2005
ESEYOQ	175,6	2,1200	2,1110	8,3100	C2/c	293	2004
ESEYUW	177,7	2,1460	2,1470	5,5600	P21/c	298	2004
ETOQIN	176,3	2,2230	2,2230	7,3000	C2	293	2004
ETOQIN	175,4	2,1970	2,1860	7,3000	C2	293	2004
ETUFOQ	172,2	2,1600	2,1600	1,9200	C2/c	213	2016
ETUXOH	177,0	2,1180	2,1180	4,4400	C2/c	298	2011
EWIZAN	174,8	2,1040	2,1080	1,9400	P21/c	100	2016
EWOVUJ	172,8	2,0900	2,0860	3,4800	P212121	100	2016
EXOXIZ	170,6	2,0630	2,0670	5,4400	Cc	293	2011
EXOXIZ	176,3	2,0910	2,0770	5,4400	Cc	293	2011
EXOXIZ	175,8	2,0780	2,0840	5,4400	Cc	293	2011
EYAQAW	175,3	2,1270	2,1510	6,7400	P-1	293	2004
EYEXEM	177,9	2,0840	2,0840	3,1000	P21/c	293	2011
EYOWOF	179,1	2,1350	2,1290	6,2300	Pna21	298	2011
EYOWUL	177,1	2,0870	2,0830	6,8300	P21/c	298	2011
EYUQAR	173,8	2,1040	2,1140	5,3600	P-1	298	2011
EZARUT	171,7	2,1360	2,1360	4,6300	Cmca	296	2011
EZASAA	174,2	2,1390	2,1460	4,3400	P21/n	296	2011
EZASEE	172,0	2,1500	2,1450	3,9600	P-1	296	2011
EZECAO	171,2	2,1250	2,1350	2,8300	P21/n	173	2011
EZUKOZ	180,0	2,2120	2,2120	5,6600	P-1	120	2004
EZUKOZ01	180,0	2,2120	2,2120	5,6600	P-1	120	2005
EZUKUF	174,5	2,0940	2,0940	3,0700	P-421c	150	2004
EZUKUF01	174,6	2,0930	2,0930	2,8800	P-421c	150	2005
FACMAY	175,2	2,1610	2,1520	2,8100	P-1	296	1999
FACTAH	180,0	2,1730	2,1730	2,7500	C2/c	296	2015
FAGLIL	180,0	2,0910	2,0910	7,1300	P-1	173	2016
FAPZUS	175,4	2,1340	2,1020	5,4500	P-1	173	2004
FAQWIF	180,0	2,1530	2,1530	3,9000	P21/c	298	2012
FARHUE	172,1	2,1730	2,1600	4,5800	P21/n	301	2017
FEGFII	175,5	2,1750	2,1640	2,7900	P21/c	200	2013
FELFOS	175,0	2,1310	2,1270	3,1600	C2/c	120	2005
FEPBUZ	180,0	2,1340	2,1340	5,7800	C2/c	90	2013
FEPLIY	178,8	2,1130	2,1130	6,8800	P-1	120	2017
FEPLIY	174,7	2,0980	2,1060	6,8800	P-1	120	2017
FEPLIY	176,1	2,1080	2,0910	6,8800	P-1	120	2017
FEPMAR	171,1	2,1200	2,1090	4,3000	P21/c	120	2017
FEPMAR	178,4	2,0970	2,0950	4,3000	P21/c	120	2017
FEPMAR	176,5	2,1190	2,1200	4,3000	P21/c	120	2017
FEPMEV	173,4	2,0960	2,1050	5,5200	P21	120	2017
FEPMEV	175,2	2,1030	2,0890	5,5200	P21	120	2017
FEPMEV	178,4	2,0690	2,0880	5,5200	P21	120	2017
FEPMEV	177,6	2,0980	2,0960	5,5200	P21	120	2017
FEPMEV	177,5	2,0920	2,1000	5,5200	P21	120	2017
FEPMEV	173,4	2,1020	2,1170	5,5200	P21	120	2017
FEVFOD	172,5	2,0910	2,0790	5,1300	P21/c	293	2013
FEVFOD	174,1	2,0790	2,0800	5,1300	P21/c	293	2013
FEVFOD	174,7	2,0930	2,0870	5,1300	P21/c	293	2013
FEYPOP	171,5	2,1460	2,1440	4,0700	I41/a	293	2005
FEYQAC	178,6	2,1120	2,1060	6,0800	P-1	293	2005
FIDGEF	179,1	2,0920	2,0740	3,9800	Fdd2	293	2005
FIDGEF	177,5	2,1070	2,0970	3,9800	Fdd2	293	2005
FIDGEF	174,5	2,1420	2,1330	3,9800	Fdd2	293	2005
FIDGEF	175,5	2,1460	2,1170	3,9800	Fdd2	293	2005
FIFYOJ	171,9	2,1350	2,1310	2,5600	C2/c	100	2005
FIGWID	180,0	2,1310	2,1310	8,4700	P-1	293	2013
FIGWID	171,3	2,1390	2,1240	8,4700	P-1	293	2013

FIGWOJ	170,7	2,1320	2,1390	9,1300	P-1	173	2013
FIGWOJ	180,0	2,1350	2,1350	9,1300	P-1	173	2013
FIGWUP	180,0	2,1260	2,1260	6,0400	P-1	173	2013
FIHJOX	176,7	2,1720	2,1770	3,1800	P21/c	293	2013
FIHJUD	176,6	2,1630	2,1560	5,7500	P21/c	100	2013
FINQOK	175,6	2,1430	2,1470	8,0300	P-1	298	2013
FINQUQ	174,3	2,1320	2,1260	3,0800	P-1	298	2013
FIRRAC	177,3	2,1700	2,1780	9,8800	P-1	173	2018
FIRREF	179,9	2,0890	2,0920	3,2400	R-3	291	2014
FIRROQ	178,8	2,1870	2,1890	7,0000	P-1	203	2018
FIRRUW	177,0	2,1780	2,1670	7,5500	P-1	203	2018
FIRSAD	175,9	2,1920	2,1590	12,6100	P-1	203	2018
FISDIV01	171,8	2,0810	2,0980	2,8300	C2/c	203	2016
FISDIV01	170,9	2,0920	2,1020	2,8300	C2/c	203	2016
FISDIV01	171,7	2,0740	2,0800	2,8300	C2/c	203	2016
FIWJAZ	180,0	2,1220	2,1220	1,9700	P21/n	150	2019
FIZCIB	177,7	2,1300	2,1250	4,7300	P2/c	150	2005
FIZCOH	170,3	2,1100	2,1280	6,9400	C2/c	150	2005
FIZCOH	173,0	2,1430	2,1490	6,9400	C2/c	150	2005
FOBYIG	171,7	2,1170	2,1160	3,5900	P-1	293	2014
FOCGIO	174,5	2,1140	2,1260	8,8200	C2/c	150	2005
FOCGIO	176,1	2,1130	2,1300	8,8200	C2/c	150	2005
FONRUW	172,4	2,1730	2,1720	3,9800	P-1	293	2009
FONSAD	176,9	2,1340	2,1300	4,6600	P21/c	298	2009
FOPJUP	175,3	2,1460	2,1460	4,1000	P-1	295	1987
FOPPIK	180,0	2,1420	2,1420	6,6700	P-1	293	2009
FOPPIK	172,7	2,1070	2,1580	6,6700	P-1	293	2009
FORGUQ	171,7	2,1380	2,1710	7,1900	P-1	293	2015
FORKUT	180,0	2,1190	2,1190	4,2100	P-1	293	2009
FORKUT	180,0	2,1460	2,1460	4,2100	P-1	293	2009
FORKUT01	180,0	2,1190	2,1190	4,4100	P-1	293	2009
FORKUT01	180,0	2,1320	2,1320	4,4100	P-1	293	2009
FORLAA	178,6	2,0980	2,0870	8,4800	P21/c	293	2009
FOVTER	174,1	2,1190	2,1340	3,4200	P21/n	100	2014
FUFVOT	175,4	2,1670	2,1670	9,6700	C2/c	296	2015
FUFVUZ	176,2	2,1670	2,1670	5,4900	C2/c	293	2015
FUFWAG	176,5	2,1590	2,1590	5,3100	C2/c	293	2015
FUFWEK	176,7	2,1640	2,1640	5,4400	C2/c	293	2015
FUGPAZ	173,3	2,1080	2,1000	3,9000	C2/c	298	2009
FUJLEC	177,4	2,1510	2,1460	10,7700	P2/c	150	2010
FUJLOM	180,0	2,1080	2,1080	5,4600	P21/c	150	2010
FUWXIE	174,7	2,0880	2,0870	6,0000	P21/n	295	1988
FUWXIE	175,6	2,0760	2,0760	6,0000	P21/n	295	1988
FUWXIE	176,3	2,0810	2,0860	6,0000	P21/n	295	1988
GAFJEE	177,5	2,0810	2,0840	4,4100	C2/m	211	2010
GAFJEE	178,8	2,0930	2,0850	4,4100	C2/m	211	2010
GAFJEE	179,1	2,0840	2,0950	4,4100	C2/m	211	2010
GAFJII	175,2	2,0740	2,0600	4,6300	P21/n	218	2010
GAFJII	175,1	2,0710	2,0590	4,6300	P21/n	218	2010
GAHVUJ	179,5	2,1050	2,1050	3,8900	P2/c	100	2016
GAJQUF	178,5	2,0940	2,0980	3,0500	P21/c	295	2010
GAJWUL	170,6	2,2770	2,2470	7,7700	P-1	293	2010
GAKNEM	175,2	2,1550	2,1510	6,7200	P21/c	293	2004
GALCUT	174,1	2,1550	2,1550	7,7100	Cmc21	296	2012
GAMXIC	173,2	2,0960	2,1230	6,4000	P-1	153	2005
GAMXIC	172,9	2,1120	2,1090	6,4000	P-1	153	2005
GAQVUQ	170,8	2,1120	2,1180	4,7000	P21/c	293	2005
GAXGIY	177,8	2,1730	2,1730	5,9100	I41/a	173	2017
GAXJIB	175,4	2,1620	2,1640	4,7200	C2	173	2017
GEFDEB	177,5	2,1070	2,1240	4,6300	C2/c	293	2006
GEGYAU	170,5	2,1150	2,1280	3,7400	P21/c	296	2012
GEKYOM	174,2	2,1540	2,1420	2,8500	P-1	295	2011
GERQOJ	180,0	2,4140	2,4140	3,8100	R32	295	1997
GEXFOG	171,1	2,1520	2,0740	7,2100	P-1	293	2013
GEXGAT	175,5	2,1230	2,1320	5,5600	C2/c	293	2013
GEXGAT	174,3	2,1360	2,1380	5,5600	C2/c	293	2013
GIHFUA	178,7	2,0950	2,0950	6,5500	Cc	153	2013
GIHFUA	173,6	2,0930	2,0550	6,5500	Cc	153	2013
GIHFUA	175,5	2,0640	2,0770	6,5500	Cc	153	2013
GIHFUA	177,4	2,0680	2,1040	6,5500	Cc	153	2013
GIHFUA	174,1	2,1030	2,1250	6,5500	Cc	153	2013
GIHFUA	173,1	2,0960	2,1080	6,5500	Cc	153	2013
GIHGAH	176,8	2,0890	2,0730	9,3400	P-1	153	2013
GIHGAH	175,4	2,0830	2,0740	9,3400	P-1	153	2013
GIHGAH	177,5	2,0810	2,0860	9,3400	P-1	153	2013
GIHGAH	175,1	2,0750	2,0890	9,3400	P-1	153	2013
GIHGAH	175,8	2,0790	2,0810	9,3400	P-1	153	2013
GIHGAH	171,5	2,1000	2,0920	9,3400	P-1	153	2013

GIHXOL	175,4	2,1460	2,1520	3,0400	P-1	173	2007
GIKJAN	174,6	2,1120	2,1210	4,2000	P-1	298	2013
GIQGAP	178,3	2,0950	2,0950	7,2100	P6322	173	2007
GIQGET	179,5	2,0680	2,0680	8,1100	P6322	150	2007
GIYZOG	174,4	2,0910	2,0950	4,2600	P21/c	296	2018
GOBNER	173,0	2,1540	2,1800	7,5300	P21/c	294	2008
GOBNER	176,8	2,1680	2,1180	7,5300	P21/c	294	2008
GOBQEU01	180,0	2,1800	2,1800	4,3400	P-1	153	2011
GOBQEU01	180,0	2,2000	2,2000	4,3400	P-1	153	2011
GOLNIF	171,9	2,1300	2,1270	5,5500	P21/n	100	2009
GOSGUS	171,6	2,0970	2,0940	4,7400	P-1	200	2015
GUCYIO	180,0	2,1070	2,1070	4,6700	P-1	100	2015
GUCYIO	177,7	2,1110	2,1100	4,6700	P-1	100	2015
GUCYIO	180,0	2,1130	2,1130	4,6700	P-1	100	2015
GUCYIO	178,6	2,1140	2,1130	4,6700	P-1	100	2015
GUCYIO	176,0	2,1150	2,1200	4,6700	P-1	100	2015
GUCZAH	174,2	2,1250	2,1150	4,5900	P-1	100	2015
GUCZAH	175,9	2,1390	2,1260	4,5900	P-1	100	2015
GUCZEL	172,8	2,1280	2,1240	2,7900	P-1	100	2015
GUCZEL	176,3	2,1520	2,0890	2,7900	P-1	100	2015
GUCZIP	178,4	2,1480	2,1480	2,9300	P-1	100	2015
GUCZIP	177,3	2,1750	2,1780	2,9300	P-1	100	2015
GUCZOV	175,7	2,1380	2,1090	2,1500	C2/c	100	2015
GUDBAJ	170,8	2,1860	2,1680	5,3100	P-1	295	2009
GUDBEN	172,3	2,1700	2,1780	3,1000	P-1	224	2009
GUDCEO	175,6	2,2010	2,2010	6,5800	C2/c	224	2009
GUFYAJ	180,0	2,1530	2,1530	2,8200	P-1	173	2015
GUJQIN	174,6	2,0880	2,1060	4,0400	P21/c	100	2015
GUJQIN	173,9	2,1150	2,1070	4,0400	P21/c	100	2015
GUJQOT	174,8	2,0990	2,0910	6,1000	P-1	100	2015
GUJQUZ	170,3	2,1450	2,1160	3,9300	Pna21	100	2015
GUJQUZ	170,2	2,1050	2,1250	3,9300	Pna21	100	2015
GUJQUZ	171,0	2,1060	2,1300	3,9300	Pna21	100	2015
GUJQUZ	171,2	2,1500	2,1230	3,9300	Pna21	100	2015
GUKXOA	180,0	2,1840	2,1840	8,4600	C2/m	293	2009
GUPPOW	173,0	2,1210	2,1170	3,5100	P1	295	2000
GUPPOW01	171,4	2,1340	2,1350	5,9300	P-1	294	2001
GUPPOW02	171,3	2,1340	2,1310	0,0000	P-1	295	2005
GUPYOH	170,6	2,1070	2,1070	4,7500	C2/c	293	2015
GUXTOJ	172,9	2,1610	2,1580	4,6500	P-1	150	2009
HAKVIB	176,7	2,1120	2,1120	4,4100	P63/mmc	174	2016
HAMLAJ	180,0	2,1250	2,1250	5,9700	P-1	293	2004
HAPFEL	175,1	2,0810	2,0920	7,8600	P21/n	133	2012
HAPFEL	172,3	2,0740	2,0920	7,8600	P21/n	133	2012
HAPFEL	178,2	2,0740	2,0750	7,8600	P21/n	133	2012
HAPFUB	173,8	2,0740	2,0870	2,8600	P-1	100	2012
HAPFUB	171,3	2,1010	2,0980	2,8600	P-1	100	2012
HAPFUB	173,9	2,1030	2,0950	2,8600	P-1	100	2012
HAXQAY	179,1	2,3080	2,3070	3,2000	P-1	200	1999
HAXQAY	178,7	2,3130	2,3170	3,2000	P-1	200	1999
HAYWEL	177,3	2,1170	2,1220	5,8200	P-1	293	2009
HAYWEL	174,7	2,1280	2,1270	5,8200	P-1	293	2009
HAYXOW	170,8	2,1680	2,1540	3,9500	P-1	293	2009
HAYXUC	176,5	2,1310	2,1360	4,2000	P-1	293	2009
HAZBER	174,1	2,1740	2,1660	9,2400	P2/c	293	2009
HEFFII	170,2	2,1320	2,1330	4,1200	P-1	120	2006
HEFFII	173,3	2,1480	2,1470	4,1200	P-1	120	2006
HEFFOO	178,9	2,1090	2,1140	5,8700	Cc	120	2006
HEFFOO	178,0	2,1320	2,1250	5,8700	Cc	120	2006
HEKKUD	179,0	2,1140	2,1170	3,4000	Pbca	295	1994
HEQTOO	174,8	2,1570	2,1520	2,7000	P21/n	143	2013
HETQEC	175,8	2,1030	2,1110	5,3000	P21/c	173	1998
HEXLUS	180,0	2,1110	2,1110	4,6300	P-1	294	2007
HICHIL	177,8	2,0770	2,0750	2,3100	P-1	100	2007
HICHIL	178,0	2,0840	2,0790	2,3100	P-1	100	2007
HICHIL	178,6	2,0760	2,0790	2,3100	P-1	100	2007
HICHOR	171,8	2,0940	2,0850	2,7500	P21/n	293	2007
HICHOR	173,0	2,0930	2,0860	2,7500	P21/n	293	2007
HICHUX	176,4	2,0840	2,0920	5,9000	P21/c	293	2007
HICHUX	179,3	2,0900	2,0940	5,9000	P21/c	293	2007
HICHUX	174,1	2,0840	2,0870	5,9000	P21/c	293	2007
HICJAF	177,1	2,1010	2,1020	2,1400	P21/c	100	2007
HICJAF	175,2	2,0980	2,1020	2,1400	P21/c	100	2007
HICJAF	177,3	2,1100	2,1090	2,1400	P21/c	100	2007
HICJEJ	172,7	2,0750	2,0820	2,9800	P-1	100	2007
HICJEJ	179,3	2,0870	2,0890	2,9800	P-1	100	2007
HICJEJ	172,2	2,0960	2,0940	2,9800	P-1	100	2007
HICJEJ	178,1	2,0990	2,0900	2,9800	P-1	100	2007

HICJEJ	175,3	2,0800	2,0750	2,9800	P-1	100	2007
HICJEJ	173,1	2,0810	2,0860	2,9800	P-1	100	2007
HICJEJ	177,7	2,0920	2,0890	2,9800	P-1	100	2007
HICJEJ	170,9	2,0960	2,0960	2,9800	P-1	100	2007
HICJEJ	172,9	2,1140	2,1060	2,9800	P-1	100	2007
HICJEJ	174,2	2,0840	2,0790	2,9800	P-1	100	2007
HICJEJ	172,5	2,0900	2,0970	2,9800	P-1	100	2007
HICJEJ	174,9	2,0930	2,0970	2,9800	P-1	100	2007
HICJEJ	170,4	2,1000	2,0980	2,9800	P-1	100	2007
HICJEJ	171,4	2,1020	2,1080	2,9800	P-1	100	2007
HIPQEE	178,0	2,0780	2,0780	2,5300	P-1	100	2013
HIPQEE	173,6	2,1300	2,1280	2,5300	P-1	100	2013
HIPQEE	172,6	2,1040	2,1010	2,5300	P-1	100	2013
HIQHIZ	179,1	2,1710	2,1730	3,1200	P21/n	294	2007
HIQRUV	176,5	2,1030	2,1020	3,5700	C2/c	293	2007
HIWHEB	176,3	2,1000	2,1220	3,8200	P-1	193	2007
HIZPEN	176,9	2,1100	2,1100	3,1000	P-1	100	2014
HIZPIR	177,4	2,0950	2,0970	5,7300	P21/c	100	2014
HIZPOX	179,0	2,0950	2,0970	3,7200	P-1	100	2014
HIZPUD	176,7	2,0820	2,0840	4,5900	P-1	173	2014
HIZQAK	176,0	2,0850	2,0880	4,1200	P-1	100	2014
HORWAN	180,0	2,1820	2,1820	3,2800	C2/c	100	2009
HORWER	175,7	2,1720	2,1660	3,7800	P21/c	200	2009
HOVJOT	173,6	2,1470	2,1550	4,0800	P-1	296	2014
HOVZOJ	175,7	2,0820	2,1190	2,8200	P-1	130	2015
HOYZEC	175,2	2,0970	2,0970	9,5200	C2/c	190	2014
HOYZEC	177,4	2,1290	2,1390	9,5200	C2/c	190	2014
HOYZIG	172,0	2,1240	2,1190	3,5000	P-1	100	2014
HUBWIL	176,6	2,1080	2,1080	5,5400	C2/c	120	2009
HUHBER	176,4	2,1370	2,1340	5,7300	P-1	293	2002
HUHFIB	177,8	2,1190	2,1010	4,4400	P2/c	293	2015
HUHFIB	170,0	2,1360	2,1270	4,4400	P2/c	293	2015
HUTSOE	180,0	2,1620	2,1620	4,4900	P-1	293	2003
HUTSUK	180,0	2,1710	2,1710	4,3700	C2/c	298	2003
HUWCAE	173,0	2,1110	2,1170	3,7000	P21/c	223	2009
HUWCIM	173,9	2,1780	2,1770	3,5500	C2/c	296	2009
IBANUX	180,0	2,1540	2,1540	1,8300	C2/c	133	2016
IBARIP	180,0	2,1080	2,1080	2,8100	C2/c	293	2014
IBAXAN	170,4	2,1460	2,1430	3,0900	P-1	133	2016
IDUPUV	176,6	2,0870	2,0780	6,4900	P-1	200	2018
IGOQEB	172,2	2,1590	2,1530	4,0200	P-1	100	2009
IGUPOP	172,5	2,1660	2,1790	2,8700	P-1	168	2002
IHONIC	173,0	2,1000	2,1160	2,7400	P-1	296	2003
IHONIC	175,4	2,1060	2,1240	2,7400	P-1	296	2003
IHONUO	177,0	2,1090	2,1100	4,3400	P-1	295	2003
IHONUO	176,2	2,1080	2,0900	4,3400	P-1	295	2003
IHONUO	176,1	2,0920	2,1080	4,3400	P-1	295	2003
IHONUO	174,5	2,0880	2,0970	4,3400	P-1	295	2003
IKOXAH	180,0	2,1420	2,1420	3,4000	P-1	100	2003
ILAYOL	174,5	2,0870	2,0920	2,4400	P21/n	296	2016
ILUJOO	177,0	2,1300	2,1380	6,9500	P21/n	120	2003
IMIHAO	174,1	2,0650	2,0740	2,4700	P21/c	140	2011
IMIQOL	174,6	2,2340	2,2420	9,2700	P21/n	293	2010
INAKUE	174,3	2,1730	2,1690	3,9100	Cmca	298	2010
INALEP	177,7	2,1080	2,1080	2,7800	P21/c	298	2010
IQIGUK	170,9	2,1050	2,1100	3,1700	P21/c	293	2003
ISAHUH	180,0	2,1010	2,1010	4,5000	P-1	100	2016
ISAJAP	174,5	2,0710	2,0600	5,7000	P-1	100	2016
ISAJET	172,4	2,0630	2,0690	5,3100	P-1	100	2016
ISAJET	175,1	2,0740	2,0790	5,3100	P-1	100	2016
ISAJIX	177,0	2,0600	2,0570	5,3900	P-1	100	2016
ISAJIX	172,8	2,0600	2,0690	5,3900	P-1	100	2016
ISECIT	175,0	2,1730	2,1640	3,4600	P21	173	2011
IWOSAQ	180,0	2,1360	2,1360	3,0900	P21/c	296	2016
IXIROX	176,2	2,2000	2,1900	2,4700	Pbcn	173	2009
IXIRUD	174,2	2,1330	2,1410	4,6300	Pbcn	173	2009
IXIVOA	180,0	2,0960	2,0960	4,8900	P-1	293	2004
IXODOO	174,5	2,1250	2,1280	3,9400	P-4n2	293	2004
IZEYAP	180,0	2,1410	2,1410	1,7800	Pnnm	133	2016
IZEYUJ	180,0	2,1470	2,1470	3,0000	P-1	133	2016
IZIJUY	180,0	2,1720	2,1720	2,1100	P-1	133	2016
IZILOU	170,7	2,1390	2,1430	3,3000	Pna21	133	2016
JABSAH	176,7	2,1350	2,1210	6,1900	P213	293	2003
JABSEL	173,7	2,1660	2,1660	4,9300	P-1	293	2003
JAGRIT	177,1	2,1370	2,1410	4,3900	P-1	293	2003
JAGRIT	179,1	2,1390	2,1390	4,3900	P-1	293	2003
JAJQEQ	171,7	2,1450	2,1450	2,1100	C2/c	173	1998
JAJQOA	170,3	2,1350	2,1410	3,6200	P-1	173	1998

JAVWAE	173,6	2,0950	2,0810	6,2200	R-3	223	1999
JAVWAE	172,5	2,0780	2,0830	6,2200	R-3	223	1999
JAVWEI	173,8	2,0840	2,0830	4,7600	R-3	223	1999
JAVWEI	172,8	2,0880	2,0940	4,7600	R-3	223	1999
JAXKOL	180,0	2,1080	2,1080	2,3800	C2/c	133	2017
JAYBOB	172,9	2,1430	2,1430	4,5500	C2/c	292	2005
JEFCUV	180,0	2,1250	2,1250	4,3900	P-1	173	2016
JEJVIG	171,2	2,1170	2,1370	10,1500	P21/n	150	2017
JEJVIG	171,7	2,1270	2,1280	10,1500	P21/n	150	2017
JEJVIG	173,9	2,0580	2,1100	10,1500	P21/n	150	2017
JEJVIG	172,9	1,9970	2,0820	10,1500	P21/n	150	2017
JEJVOM	174,7	2,1380	2,1380	8,0100	C2/m	293	2017
JEJVUS	173,1	2,4800	2,4800	4,7600	Immm	150	2017
JEJWAZ	170,5	2,1010	2,1010	2,8200	Immm	150	2017
JEJWED	172,9	2,1120	2,1120	4,3600	Immm	150	2017
JEJWIH	173,0	2,0810	2,0810	4,3800	Immm	150	2017
JEJWON	170,7	2,0670	2,1410	10,0800	P-1	293	2017
JEJWON	170,8	2,1530	2,0720	10,0800	P-1	293	2017
JEJWUT	170,5	2,1150	2,1150	5,7600	Immm	150	2017
JELXIK	177,5	2,0940	2,0830	3,5800	P21/c	298	2016
JEWKUU	172,1	2,1040	2,1120	10,9400	P-1	296	2018
JEWKUU	171,9	2,1010	2,1150	10,9400	P-1	296	2018
JEWKUU	172,2	2,1260	2,1030	10,9400	P-1	296	2018
JEWXAN	176,7	2,0310	2,1000	6,8300	P-1	296	2018
JIQDOF	175,8	2,1490	2,1500	4,1600	P2/c	293	2018
JIQDOF	174,9	2,1480	2,1530	4,1600	P2/c	293	2018
JIYYOH	176,4	2,0590	2,0130	5,0200	P1	293	2014
JIYYOH	174,9	2,0740	2,1640	5,0200	P1	293	2014
JIYYOH	173,3	2,0860	2,0180	5,0200	P1	293	2014
JIYYOH	174,1	2,1900	2,0910	5,0200	P1	293	2014
JOKWEL	171,0	2,1520	2,1390	6,5400	P-1	295	1992
JOKWEL	170,5	2,1570	2,1520	6,5400	P-1	295	1992
JUCGEV	172,9	2,1140	2,1090	2,0400	P-1	296	2015
JUPFOP	178,1	2,2550	2,2400	4,8700	Pbcn	295	1999
JUSJAK	174,0	2,0550	2,0750	3,2800	P212121	293	2015
JUSJAK	173,0	2,0700	2,0990	3,2800	P212121	293	2015
KAJGOT	175,4	2,1490	2,1480	3,2400	P-1	120	2010
KAJGUZ	174,2	2,1750	2,1690	6,1700	Pmc21	173	2010
KAJGUZ	173,5	2,1830	2,2040	6,1700	Pmc21	173	2010
KALPAP	175,2	2,1800	2,1800	5,6500	Pnma	293	2005
KANYEG	174,1	2,1210	2,1110	6,5300	P21/c	298	2016
KAPFIS	170,4	2,0540	2,0310	8,5500	P21/c	293	2011
KAPGIS	170,1	2,1360	2,1320	3,8200	P21/n	293	2005
KAXTOU	180,0	2,1240	2,1240	6,5700	P-1	293	2012
KAXTUA	176,7	2,1080	2,1140	3,6700	P-1	293	2012
KAYSEJ	175,6	2,1140	2,1230	5,5800	C2/c	298	2004
KEHJUE	174,0	2,0940	2,0990	4,4300	P-1	100	2012
KEHJUE	174,0	2,0980	2,0920	4,4300	P-1	100	2012
KEHKAL	177,3	2,0980	2,1060	6,7800	P21/c	100	2012
KEHKEP	177,9	2,0890	2,0940	5,0400	P21/c	100	2012
KEHKIT	177,0	2,1000	2,0910	4,4300	P21/c	298	2012
KEJRIC	175,0	2,1220	2,1280	3,1200	P-1	113	2012
KELZAE	174,7	2,0730	2,0820	4,8200	P21/n	100	2012
KELZAE	173,2	2,1140	2,1000	4,8200	P21/n	100	2012
KELZAE	179,9	2,0670	2,0820	4,8200	P21/n	100	2012
KELZAE	178,2	2,0990	2,0890	4,8200	P21/n	100	2012
KELZAE	174,4	2,0990	2,0980	4,8200	P21/n	100	2012
KELZEI	174,8	2,0950	2,0880	4,1800	P-1	100	2012
KELZEI	176,6	2,1000	2,0970	4,1800	P-1	100	2012
KELZEI	175,7	2,0970	2,0880	4,1800	P-1	100	2012
KEMVIJ	176,7	2,0960	2,1040	2,3300	P-1	150	2012
KETLAZ	176,3	2,2310	2,2220	4,0700	Pccn	200	2017
KEWNIL	176,1	2,1650	2,1430	7,3200	Cmcm	293	2013
KEWNOR	172,3	2,1390	2,1060	7,9000	P-1	298	2013
KEXQUZ	175,2	2,0910	2,0910	4,6500	P-1	295	2000
KEXSIP	178,4	2,0910	2,0880	5,9400	P21/c	180	2000
KIDYOM	178,5	2,1700	2,1010	3,7000	P1	295	2007
KIDYOM01	179,8	2,1270	2,1270	0,0000	P-1	295	2009
KIDZIH	179,3	2,0760	2,1180	4,2100	P1	295	2007
KIDZIH01	179,6	2,1170	2,1360	0,0000	P-1	295	2009
KIJREC	180,0	2,1540	2,1540	3,7800	P-1	293	2013
KIRWIS	175,6	2,1740	2,1080	8,2200	P-1	150	2007
KIRXAL	170,9	2,1420	2,1360	6,2000	Pca21	150	2007
KIRXAL	171,6	2,1580	2,1320	6,2000	Pca21	150	2007
KIRXEP	176,5	2,1250	2,1300	3,7400	P-1	150	2007
KIRXEP	175,1	2,1550	2,1480	3,7400	P-1	150	2007
KISNAE	178,3	2,0820	2,0720	6,4800	P-1	200	2019
KISNAE	175,6	2,0760	2,0730	6,4800	P-1	200	2019

KISNAE	177,7	2,0520	2,0720	6,4800	P-1	200	2019
KITMAD	174,4	2,0910	2,0870	3,1800	Pna21	100	2014
KIXKOT	172,1	2,1540	2,1570	9,6900	P-1	293	2013
KOHZOY	173,4	2,1430	2,1490	7,5400	P21/c	298	2014
KOHZOY	170,8	2,1330	2,1320	7,5400	P21/c	298	2014
KOLHIC	171,2	2,1690	2,1690	2,9000	C2/c	295	1991
KONXOC	180,0	2,1370	2,1370	4,5700	P-1	140	2014
KULDUS	177,9	2,1220	2,1220	10,2800	C2/m	123	2014
KULDUS	174,4	2,1600	2,1600	10,2800	C2/m	123	2014
KULFAA	176,1	2,1760	2,1760	6,9700	P-421c	173	2014
KULFAA	174,9	2,1400	2,1630	6,9700	P-421c	173	2014
KULFAA	177,0	2,1350	2,1350	6,9700	P-421c	173	2014
KUNRIW	177,9	2,0720	2,0720	6,7800	P6322	150	2007
KUNROC	177,6	2,0500	2,0500	7,5700	P6322	150	2007
KUXWUW	171,2	2,1490	2,1280	4,9400	P-1	296	2010
KUXXEH	172,9	2,1220	2,1240	2,5900	P1	296	2010
KUXXEH	177,5	2,1290	2,1320	2,5900	P1	296	2010
KUYNOI	180,0	2,1360	2,1360	2,1400	P-1	113	2010
LAFNIQ	180,0	2,1120	2,1120	4,3600	P-1	293	2004
LAFPUE	180,0	2,1130	2,1130	4,5500	P-1	293	2004
LAHCII	180,0	2,0680	2,0680	4,1200	P-1	150	2010
LALDOT	170,9	2,2050	2,2000	3,5300	P21/n	298	2010
LALZIK	171,6	2,1070	2,1090	3,5700	I41	100	2016
LALZIK	170,4	2,1100	2,1070	3,5700	I41	100	2016
LALZIK	171,5	2,1100	2,1170	3,5700	I41	100	2016
LALZIK	171,6	2,1120	2,1190	3,5700	I41	100	2016
LALZIK	170,9	2,1130	2,1110	3,5700	I41	100	2016
LALZIK	170,6	2,1140	2,1200	3,5700	I41	100	2016
LALZUW	180,0	2,1140	2,1140	4,8800	P21/c	100	2016
LALZUW	180,0	2,1160	2,1160	4,8800	P21/c	100	2016
LAXCAR	175,4	2,1360	2,1380	4,2000	P-1	293	2017
LAZYAP	180,0	2,1260	2,1260	7,9600	C2/m	293	2017
LAZYAP	180,0	2,1560	2,1560	7,9600	C2/m	293	2017
LEDHUY	172,6	2,0940	2,0960	2,0200	P21/c	100	2006
LEDJAG	178,0	2,0990	2,0860	1,7800	P-1	100	2006
LEDXOI	174,4	2,1310	2,1320	5,3000	P21/c	273	2006
LEDXOI	174,4	2,1110	2,1310	5,3000	P21/c	273	2006
LETGEZ	177,5	2,1730	2,1630	5,5200	Pbca	296	2017
LEXDIE	180,0	2,1830	2,1830	7,4300	P-1	293	2018
LEXFEC	178,1	2,2060	2,1880	6,2700	P-1	293	2018
LEXFEC	171,9	2,2000	2,1920	6,2700	P-1	293	2018
LIBWOJ	173,7	2,0840	2,0850	4,2200	Pna21	293	2007
LICDAE	176,3	2,1070	2,1080	5,0000	P21/c	296	2012
LIFQEX	172,8	2,1040	2,1250	5,2200	P21/n	293	2007
LIFQUN	170,7	2,1990	2,1840	2,6100	P-1	293	2007
LIFREY	180,0	2,2060	2,2060	3,1600	P-1	293	2007
LIFRIC	173,4	2,1000	2,1100	5,9900	P-1	293	2007
LIFRIC	170,2	2,0840	2,0940	5,9900	P-1	293	2007
LIFRIC	174,5	2,0850	2,0850	5,9900	P-1	293	2007
LILQON	170,5	2,1070	2,1070	3,9600	C2/c	293	2007
LINPII	180,0	2,1670	2,1710	3,4000	C2/c	293	2007
LIVFUT	174,6	2,0330	2,0450	5,0400	P1	291	2014
LIVFUT	174,8	2,0530	2,1150	5,0400	P1	291	2014
LIVFUT	172,9	2,0570	2,1080	5,0400	P1	291	2014
LIVFUT	171,9	2,0690	2,0670	5,0400	P1	291	2014
LIVGAA	177,7	2,0750	2,0750	4,8400	P6522	296	2014
LIVGEE	174,8	2,0330	2,0960	5,7000	P1	291	2014
LIVGEE	174,9	2,0520	2,0780	5,7000	P1	291	2014
LIVGEE	171,7	2,0870	2,0630	5,7000	P1	291	2014
LIVGEE	175,8	2,0900	2,0990	5,7000	P1	291	2014
LIVGII	177,5	2,0760	2,0760	4,6000	P6122	296	2014
LIVKEI	180,0	2,1320	2,1320	9,4800	P-1	173	2014
LIVKEI	179,3	2,1500	2,1350	9,4800	P-1	173	2014
LIWNIP	177,9	2,1510	2,1520	3,7200	R-3c	173	2007
LOLTUC	177,8	2,0890	2,0890	6,5200	C2/c	296	2008
LONBIA	172,8	2,0980	2,1290	6,7100	P21	293	2008
LONBIA	179,1	2,1280	2,1410	6,7100	P21	293	2008
LONBIA	172,7	2,0970	2,0870	6,7100	P21	293	2008
LONBIA	179,6	2,1070	2,1280	6,7100	P21	293	2008
LONBIA	175,0	2,0910	2,1040	6,7100	P21	293	2008
LONBIA	174,2	2,1130	2,1170	6,7100	P21	293	2008
LOZQOG	173,7	2,2110	2,2100	3,8500	P21/n	168	2002
LOZQOG	171,7	2,2260	2,2160	3,8500	P21/n	168	2002
LOZQOG	170,2	2,2240	2,1660	3,8500	P21/n	168	2002
LOZQOG	173,0	2,2310	2,1870	3,8500	P21/n	168	2002
LUDROR	173,0	2,0950	2,0810	8,0100	P42/n	173	2002
LUDROR	177,0	2,0900	2,0860	8,0100	P42/n	173	2002
LUFYIV	172,5	2,3070	2,3080	6,6400	R-3c	123	2009

LUHTOZ	170,5	2,1610	2,1480	3,3700	P-1	296	2015
LUKZAU	176,9	2,1330	2,1280	5,3500	P-1	173	2015
LUKZIC	174,8	2,1430	2,1300	2,0300	P21/c	173	2015
LULNIR	176,0	2,1380	2,1370	3,0800	P21/n	170	2015
LULNOX	177,0	2,1310	2,1320	2,9400	P21/n	170	2015
LUTPUL	175,9	2,1160	2,0930	3,4000	P-1	173	2003
LUXPUR	172,4	2,1310	2,1310	3,5600	Ibca	296	2015
MADYUM	171,3	2,1610	2,1700	6,9400	P65	173	2004
MADZAT	174,4	2,1860	2,1720	7,0600	P21/n	173	2004
MAGLOY	170,9	2,0720	2,0770	3,7400	P21/n	93	2016
MAGMEP	173,7	2,0920	2,0940	4,1600	P21/n	93	2016
MAHNIT	178,2	2,1260	2,1260	5,5200	Cmca	298	2004
MARCOX	180,0	2,2020	2,2020	3,6100	Pbcn	295	2000
MARGIW	180,0	2,0710	2,0710	5,7400	C2/c	163	2005
MARGOC	173,4	2,0850	2,0860	3,8500	P-1	203	2005
MARGUI	180,0	2,0890	2,0890	5,5800	P2/n	173	2005
MARHAP	173,7	2,0740	2,0700	3,8200	P21/c	100	2005
MARHET	171,4	2,1020	2,1030	4,8100	P-1	203	2005
MARHIX	180,0	2,0710	2,0710	3,8500	P-1	183	2005
MARHOD	174,0	2,0660	2,0860	6,2300	P21/c	100	2005
MARHUJ	174,1	2,1020	2,0990	3,1300	P-1	100	2005
MATFIY	174,7	2,1200	2,1210	6,5700	P-1	293	2012
MAXKAX	172,7	2,1540	2,1660	2,5300	C2	203	2000
MECJIO	176,9	2,0690	2,0750	5,8900	P-1	293	2006
MEKDIS	175,2	2,2450	2,1990	14,7200	P2/c	173	2017
MEKFUE	180,0	2,0850	2,0850	4,7000	C2/c	293	2006
MEXGIH	180,0	2,1660	2,1660	5,4400	C2/c	293	2013
MHPXAG	173,7	2,1280	2,1260	6,2000	P-1	295	1980
MHPXAG	172,2	2,1300	2,1210	6,2000	P-1	295	1980
MIDHUD	178,3	2,0780	2,0720	4,9200	P-1	293	2007
MIDHUD	180,0	2,0930	2,0930	4,9200	P-1	293	2007
MIDJAL	175,5	2,0830	2,0940	5,8800	C2/c	293	2007
MIDJAL	172,6	2,0650	2,0890	5,8800	C2/c	293	2007
MIGQEY	173,3	2,1850	2,1850	4,9300	P21/n	168	2002
MIGQEY	172,2	2,1810	2,1660	4,9300	P21/n	168	2002
MIGQEY	172,4	2,2000	2,1930	4,9300	P21/n	168	2002
MINZUE	175,2	2,2630	2,2440	6,0100	P-1	173	2002
MIRGEZ	180,0	2,1640	2,1640	4,7200	C2/c	293	2001
MIVWEU	172,4	2,0710	2,0720	5,4800	I41/a	153	2008
MIVWEU	170,8	2,0830	2,0900	5,4800	I41/a	153	2008
MIVWEU	176,1	2,0670	2,0620	5,4800	I41/a	153	2008
MIVWEU	176,3	2,0660	2,0710	5,4800	I41/a	153	2008
MIVWEU	174,0	2,0920	2,0870	5,4800	I41/a	153	2008
MIVWEU	175,3	2,0410	2,0420	5,4800	I41/a	153	2008
MIVWEU	172,0	2,0640	2,0610	5,4800	I41/a	153	2008
MIVWEU	170,2	2,0550	2,0570	5,4800	I41/a	153	2008
MIVWEU	177,1	2,0610	2,0570	5,4800	I41/a	153	2008
MIVWEU	175,7	2,0550	2,0500	5,4800	I41/a	153	2008
MIVWEU	178,8	2,0730	2,0660	5,4800	I41/a	153	2008
MIVWEU	177,5	2,0580	2,0510	5,4800	I41/a	153	2008
MIVWEU	171,4	2,0840	2,0860	5,4800	I41/a	153	2008
MIVWEU	171,5	2,0730	2,0720	5,4800	I41/a	153	2008
MIVWEU	171,1	2,0780	2,0790	5,4800	I41/a	153	2008
MIVWEU	170,2	2,0720	2,0820	5,4800	I41/a	153	2008
MIVWEU	171,1	2,0720	2,0730	5,4800	I41/a	153	2008
MIVWEU	172,8	2,0980	2,1000	5,4800	I41/a	153	2008
MIVWEU	177,0	2,0680	2,0750	5,4800	I41/a	153	2008
MIVWEU	173,3	2,0640	2,0580	5,4800	I41/a	153	2008
MIYGOR	177,6	1,9560	2,0560	16,6400	I-42d	123	2008
MIYGOR	170,6	2,0180	2,0830	16,6400	I-42d	123	2008
MIYGOR	171,3	2,0980	2,1500	16,6400	I-42d	123	2008
MIYGOR	174,7	2,0710	2,0450	16,6400	I-42d	123	2008
MIYGOR	176,6	2,0140	1,9880	16,6400	I-42d	123	2008
MIYGOR	171,2	2,0190	2,0850	16,6400	I-42d	123	2008
MIYGOR	172,0	1,9810	2,1210	16,6400	I-42d	123	2008
MIYHAE	176,1	2,0400	2,0410	4,9400	Ccca	213	2008
MIYHAE	174,3	2,0710	2,0720	4,9400	Ccca	213	2008
MIYHAE	176,7	2,0540	2,0610	4,9400	Ccca	213	2008
MIYHAE	175,9	2,0400	2,0450	4,9400	Ccca	213	2008
MIYHAE	176,7	2,0690	2,0690	4,9400	Ccca	213	2008
MIYHAE	170,9	2,0530	2,0570	4,9400	Ccca	213	2008
MIYHAE	174,2	2,0590	2,0590	4,9400	Ccca	213	2008
MOBJIW	180,0	2,1700	2,1700	4,4500	P-1	150	2002
MOBJIW	180,0	2,1890	2,1890	4,4500	P-1	150	2002
MODKOG	175,9	2,1030	2,0990	4,3800	P-1	293	2008
MODKOG	179,0	2,0910	2,0910	4,3800	P-1	293	2008
MOHBER	173,9	2,1280	2,1330	3,0900	R-3	273	2008
MOHBER01	173,4	2,1330	2,1320	2,4100	R-3	293	2016

MOHHOH	180,0	2,2010	2,2010	3,3100	P-1	213	2008	
MOLKEF	176,8	2,1530	2,1490	3,5200	P-1	120	2013	
MOLKEF	175,8	2,1580	2,1620	3,5200	P-1	120	2013	
MOMZIY	174,6	2,1280	2,1280	8,8000	P21/c	173	2009	
MOMZUK	178,3	2,1340	2,1480	8,2500	P21/c	173	2009	
MOWNUI	180,0	2,1600	2,1600	5,1900	C2/c	293	2009	
MOYKIU	180,0	2,1200	2,1200	3,5200	P-1	168	2002	
MUBXEN	174,2	2,1470	2,1440	4,5700	P-1	173	2009	
MUBYUE	176,8	2,1210	2,1050	4,3200	P-1	293	2009	
MUBYUE	172,6	2,0890	2,0910	4,3200	P-1	293	2009	
MUTMOD	174,2	2,1640	2,1610	3,6100	P-1	293	2003	
NABYOF	180,0	2,1300	2,1300	2,3700	P-1	293	2004	
NABYOF	180,0	2,1320	2,1320	2,3700	P-1	293	2004	
NAGLOY	171,9	2,0800	2,0750	4,5200	P-1	173	2010	
NAGLUE	180,0	2,0900	2,0900	4,5600	P-1	173	2010	
NAGMAL	180,0	2,0980	2,0980	3,0200	P-1	173	2010	
NAJXOO	178,5		5,9400	I4/mcm	296	2016		
NEGQOI	180,0	2,1380	2,1380	1,6500	P-1	113	2017	
NEGXIJ	174,6		18,6800	C2221	90	2017		
NEGYAC	173,8	2,0960	2,2270	9,9700	P43	100	2017	
NEGYAC	176,0	2,1760	2,2660	9,9700	P43	100	2017	
NEKWUV	172,3	2,1370	2,1370	4,4900	C2/c	160	1998	
NENRAZ	180,0	2,1120	2,1120	3,1700	P-1	200	2001	
NENRIH	178,2	2,1140	2,1120	3,5100	P21/n	200	2001	
NENROP	177,6	2,1290	2,1190	5,2300	P21/c	298	2012	
NEPSEJ	180,0	2,1350	2,1350	2,9400	P-1	293	2018	
NEVRAI	173,8	2,1260	2,1330	4,5700	Pbcn	150	2007	
NEVREM	174,8	2,1270	2,1230	3,1600	Pbcn	150	2007	
NEVRIQ	178,1	2,1290	2,1290	5,3300	P42/nnm		298	2007
NEXXOE	176,5	2,1160	2,1160	7,6900	C2/m	292	2006	
NIGHIX	177,9	2,1600	2,1560	4,6100	P-1	123	2017	
NIGHIX	178,1	2,1600	2,1590	4,6100	P-1	123	2017	
NISFUQ	176,1	2,1440	2,1430	4,2800	P-1	173	1998	
NISJAA	171,0	2,1540	2,1520	2,6900	P-1	173	1998	
NISNEI	174,9	2,1600	2,1650	2,8600	P-1	173	1998	
NITXUK	171,3	2,1010	2,1190	4,2100	R3	93	2007	
NIVZID	179,8	2,1330	2,1300	5,0600	P21/n	187	2014	
NOHMOP	175,7	2,0860	2,0830	3,0800	P-1	120	2019	
NOMBID	178,7	2,1510	2,1470	8,0400	P21/c	150	2019	
NOMBID	174,9	2,1540	2,1650	8,0400	P21/c	150	2019	
NOMBID	177,6	2,1460	2,1590	8,0400	P21/c	150	2019	
NOMBID	178,3	2,1560	2,1640	8,0400	P21/c	150	2019	
NOWGIR	170,6	2,1800	2,1560	5,4500	Cc	293	2015	
NUGFUS	174,0	2,0570	2,0570	5,7100	I41	100	2015	
NUGFUS	174,9	1,9680	2,0940	5,7100	I41	100	2015	
NULTIX	170,2	2,1530	2,1330	4,3000	I2/m	295	1997	
NULTIX	178,9	2,1140	2,1140	4,3000	I2/m	295	1997	
NUNSEW	176,4	2,1430	2,1370	5,8200	P-1	295	2015	
NUNSUM	180,0	2,1380	2,1380	6,0900	P21/c	295	2015	
NUVBUC	176,0	2,1390	2,1530	16,1000	P21/c	298	2010	
NUVCAJ	173,1	2,1600	2,1450	8,8000	P21/m298		2010	
NUVGUH	177,0	2,0990	2,0680	7,6800	Pnna	298	2010	
NUYQIJ	174,1	2,0960	2,0980	5,0000	P-1	100	2015	
NUYQOP	175,1	2,1070	2,1040	5,7500	P-1	100	2015	
NUYQUV	172,1	2,0960	2,1050	5,7500	P-1	100	2015	
NUYQUV	174,0	2,0960	2,0980	5,7500	P-1	100	2015	
NUYRAC	172,6	2,0950	2,1000	5,0400	P-1	100	2015	
NUYRAC	174,1	2,0970	2,0940	5,0400	P-1	100	2015	
NUYREG	172,4	2,0960	2,0850	6,6200	P-1	100	2015	
NUYREG	173,7	2,0860	2,0960	6,6200	P-1	100	2015	
NUYRIK	178,7	2,0960	2,1060	3,8800	P-1	100	2015	
OBEWOK	171,3	2,1560	2,1540	7,7800	P21/c	200	2016	
OBEWUQ	176,6	2,1490	2,1340	9,8700	P21/n	200	2016	
OBIHUD	171,9	2,1500	2,1630	5,1800	P21/c	150	2004	
OBIHUD	172,6	2,1360	2,1410	5,1800	P21/c	150	2004	
OBIHUD	174,3	2,1140	2,1260	5,1800	P21/c	150	2004	
OBIJAL	171,7	2,1230	2,1480	6,7800	P21/c	150	2004	
OBIJAL	171,5	2,1400	2,1410	6,7800	P21/c	150	2004	
OBIWEC	170,1	2,1290	2,1180	4,3400	P-1	296	2004	
OCIKUG	179,2	2,1120	2,1260	7,5600	P-1	293	2001	
OCIKUG01	179,2	2,1120	2,1260	7,5700	P-1	293	2001	
OCIKUG02	178,4	2,1000	2,1030	2,7500	P-1	100	2011	
OFABOP	173,5	2,1330	2,1330	5,7500	C2/c	295	2018	
OFABOP	174,2	2,1430	2,1190	5,7500	C2/c	295	2018	
OFABOP	172,1	2,1040	2,1040	5,7500	C2/c	295	2018	
OFALUE	170,1	2,1540	2,1500	4,1700	P21/c	293	2013	
OGAQEU	170,5	2,1460	2,1560	9,7600	C2/c	153	2013	
OGINAU	176,8	2,1170	2,1330	3,8400	Pca21	150	2009	

OGINAU	177,1	2,1530	2,0850	3,8400	Pca21	150	2009
OGINAU	174,0	2,1190	2,0760	3,8400	Pca21	150	2009
OGINAU	174,3	2,1230	2,0970	3,8400	Pca21	150	2009
OGOLIF	171,7	2,2040	2,2080	4,8600	P-1	293	2002
OHAHOW	170,0	2,1860	2,1930	5,1800	P-1	100	2015
OJAFOW	180,0	2,1040	2,1040	10,3600	C2/c	293	2015
OJAROI	172,4	2,1310	2,1340	5,9500	P-1	296	2015
OKEXUY	170,8	2,0760	2,0880	8,8600	C2/c	293	2010
OKEXUY	172,1	2,1110	2,1130	8,8600	C2/c	293	2010
OKUGOQ	176,4	2,1470	2,1450	3,7700	P-1	293	2001
OLALOE	173,1	2,1360	2,1510	5,3800	P21/n	100	2016
OLIVOW	173,7	2,1050	2,1240	4,3700	P-1	293	2016
OMADAH	176,1	2,1650	2,1460	6,0100	Pna21	93	2003
OMADAH	176,5	2,1830	2,1840	6,0100	Pna21	93	2003
OMADAH	173,7	2,1690	2,1860	6,0100	Pna21	93	2003
OPUCUY	173,3	2,1950	2,1690	3,8600	Cc	293	2011
OPUDAF	173,8	2,1850	2,1700	3,7600	Cc	293	2011
OPUHUD	171,0	2,0930	2,1020	5,1700	P21/c	293	2011
OQAGIX	175,2	2,1120	2,1110	3,4500	P-1	296	2011
OQINUY	176,4	2,1220	2,1100	4,5600	P21/n	293	2011
OQINUY01	180,0	2,1350	2,1350	4,1500	C2/c	293	2011
OQINUY02	180,0	2,1400	2,1400	6,3000	P-1	293	2011
OSASEH	171,3	2,1460	2,1450	3,5000	P-1	90	2011
OSEWIT	174,3	2,1530	2,1290	6,8900	P-1	293	2011
OSEWUF	172,9	2,1620	2,1610	3,8800	C2/c	293	2011
OTUVEG	174,6	2,1360	2,1440	6,0100	P-1	298	2016
OXUQAB	172,6	2,1340	2,1340	8,5600	P-1	100	2016
OZIZII	172,9	2,1480	2,1620	2,7800	P-1	133	2016
PALSED	175,4	2,1520	2,1540	4,8200	P-1	296	2017
PAPSOQ	170,7	2,1240	2,1290	5,9000	P-1	293	2011
PASBES	180,0	2,1610	2,1610	4,4000	C2/c	200	2012
PASBIW	171,9	2,1430	2,1440	2,3500	P-1	200	2012
PAVKIH	180,0	2,1650	2,1650	10,3300	P21/c	150	2005
PAVKIH	176,4	2,1260	2,1100	10,3300	P21/c	150	2005
PAVKIH	171,3	2,1740	2,1250	10,3300	P21/c	150	2005
PAWBEV	174,1	2,1420	2,1260	4,4400	P21/c	293	2005
PAWBEV	176,8	2,1430	2,1490	4,4400	P21/c	293	2005
PAWMEG	180,0	2,0870	2,0870	5,0000	P-1	293	2005
PAXQIO	177,3	2,1790	2,1710	3,8600	P-1	173	1998
PAXQIO01	177,3	2,1790	2,1710	3,8600	P-1	173	1998
PAXQOU01	176,0	2,1970	2,2080	5,0300	P-1	173	1998
PAXQOU01	180,0	2,1660	2,1660	5,0300	P-1	173	1998
PEJROM	176,4	2,0990	2,0850	6,6600	P-1	295	2006
PEJROM	170,3	2,0820	2,1000	6,6600	P-1	295	2006
PEJRUS	174,5	2,1060	2,1060	2,7500	P-1	296	2006
PEKWOR	176,3	2,1760	2,1880	4,4000	P-1	178	1993
PELBIU	174,2	2,2020	2,2030	6,1600	P-1	173	2017
PEMGUL	178,5	2,1350	2,1440	12,0300	P21/n	173	2013
PEMHEW	178,9	2,0880	2,1010	9,5200	P21/c	173	2013
PEPZUG	180,0	2,1040	2,1040	4,1200	P-1	190	2006
PEWTIU	180,0	2,1110	2,1110	7,6100	P-1	295	1993
PEXQOZ	178,7	2,1650	2,1680	4,2700	P-1	293	2007
PEXQOZ	176,0	2,1590	2,1680	4,2700	P-1	293	2007
PIBFOY	178,2	2,1160	2,1160	6,0900	C2/m	298	2018
PIBFOY	179,5	2,0950	2,0950	6,0900	C2/m	298	2018
PIBFOY	171,0	2,1460	2,1460	6,0900	C2/m	298	2018
PICVOO	172,3	2,1420	2,1420	4,6000	C2/c	153	2013
PIGPAZ	170,9	2,1490	2,1410	4,2900	C2/c	100	2018
PIHTUY	174,2	2,1360	2,1460	8,6800	P-1	298	2018
PIVJOV	177,7	2,1130	2,1060	3,7900	Pbca	100	2013
PIVJOV	178,5	2,1050	2,0980	3,7900	Pbca	100	2013
PIVJOV	178,2	2,1050	2,1050	3,7900	Pbca	100	2013
PIVJUB	176,8	2,1110	2,1130	3,1300	Pbca	100	2013
PIVJUB	178,9	2,1150	2,1030	3,1300	Pbca	100	2013
PIVJUB	178,6	2,1050	2,1020	3,1300	Pbca	100	2013
PIZJOZ	180,0	2,1690	2,1690	4,1600	P-1	100	2014
PIZJOZ	180,0	2,2240	2,2240	4,1600	P-1	100	2014
PIZKUF	180,0	2,1090	2,1090	4,6600	P-1	273	2008
PIZKUF	180,0	2,1190	2,1190	4,6600	P-1	273	2008
POBZOY	178,0	2,0540	2,0540	2,0100	Fddd	100	2019
POCPOM	180,0	2,1660	2,1660	6,7000	P-1	295	1994
POGCIZ	176,2	2,1620	2,1790	7,9400	P6522	90	2014
POGDAS	176,2	2,1530	2,1250	9,7700	P6522	90	2014
POHPUY	173,5	2,0910	2,0940	3,9000	P-1	293	2008
POKHAA	180,0	2,1530	2,1530	4,5700	P21/n	120	2014
POKHAA	180,0	2,1630	2,1630	4,5700	P21/n	120	2014
POKHII	180,0	2,1740	2,1740	1,3700	P-1	120	2014
POKHII	180,0	2,1810	2,1810	1,3700	P-1	120	2014

POKYIY	180,0	2,1820	2,1820	4,7000	P-1	223	2008
POVCUZ	174,9	2,0620	2,1390	2,3100	C2/c	100	2009
POVCUZ	177,4	2,0650	2,1250	2,3100	C2/c	100	2009
POVDIO	175,3	2,1250	2,0590	5,0800	P-1	100	2009
POVDIO	175,2	2,1350	2,0640	5,0800	P-1	100	2009
PUHVOE	172,0	2,1130	2,1200	4,1400	P21/n	293	2010
PUMQUL	174,8	2,0760	2,0830	6,0400	P-1	153	2015
PUMQUL	173,9	2,0930	2,0640	6,0400	P-1	153	2015
PUMQUL	176,9	2,0950	2,0910	6,0400	P-1	153	2015
PUMRAS	180,0	2,1440	2,1440	8,2700	P-1	293	2015
PUMRAS	176,2	2,1010	2,0760	8,2700	P-1	293	2015
PUNHOX	180,0	2,1650	2,1650	3,0400	Pbcn	296	2015
PURLAR	172,5	2,1560	2,1700	5,2100	R-3	296	2015
PURLAR	175,6	2,1110	2,1320	5,2100	R-3	296	2015
PURXUW	174,4	2,1450	2,1450	4,6000	P21/n	150	2010
PURYAD	172,2	2,1420	2,1470	4,3000	P-1	150	2010
PURYUX	175,7	2,1410	2,1410	3,2000	P-1	150	2010
PUYQAD	177,9	2,1270	2,1170	5,0000	P2/c	293	2015
PUYQAD	171,7	2,1300	2,1280	5,0000	P2/c	293	2015
PUYQAD	178,7	2,1570	2,1570	5,0000	P2/c	293	2015
PUYQEH	175,1	2,1350	2,1320	3,9800	P2/c	293	2015
QAFQIY	178,1	2,1790	2,1700	2,3600	P-1	173	2003
QAFXIH	180,0	2,0970	2,0970	11,5200	P-1	175	2016
QAGTUN	180,0	2,1350	2,1350	2,6800	C2/c	173	1999
QANXOV	175,9	2,2140	2,2170	5,7600	P-1	150	2017
QANXUB	175,9	2,1520	2,1400	2,1900	P21	150	2017
QANZOX	177,4	2,1450	2,1650	9,4600	P-1	150	2017
QANZOX	178,4	2,1380	2,1330	9,4600	P-1	150	2017
QAPPIH	180,0	2,1170	2,1170	2,9800	P-1	150	2005
QARNOM	178,4	2,1370	2,1320	7,6000	P-1	213	2000
QARSAE	176,5	2,2120	2,2190	3,0500	P-1	293	2005
QATZUI	174,6	2,2060	2,2060	8,6100	Cmca	293	2012
QAWQEL	177,6	2,1300	2,1300	2,2400	P21/n	293	2005
QAZCUP	180,0	2,1710	2,1710	4,5400	P21/n	295	2000
QEJJEX	173,7	2,1000	2,0950	5,1400	P21/n	298	2017
QEJJEX	176,4	2,1190	2,1060	5,1400	P21/n	298	2017
QEJJEX	174,5	2,0900	2,0920	5,1400	P21/n	298	2017
QEJJEX	174,9	2,1030	2,1020	5,1400	P21/n	298	2017
QEJJIB	173,6	2,0820	2,0960	6,3000	C2/c	298	2017
QEJJIB	174,0	2,0920	2,0970	6,3000	C2/c	298	2017
QEJJIB	176,8	2,0830	2,0840	6,3000	C2/c	298	2017
QEJJIB	172,2	2,0830	2,0860	6,3000	C2/c	298	2017
QEJJIB	172,5	2,0670	2,0590	6,3000	C2/c	298	2017
QEJJIB	174,6	2,0790	2,0720	6,3000	C2/c	298	2017
QEJJIB	172,4	2,0570	2,0710	6,3000	C2/c	298	2017
QEJJIB	176,9	2,0860	2,0680	6,3000	C2/c	298	2017
QEJJIB	177,0	2,0990	2,0910	6,3000	C2/c	298	2017
QEJJOH	179,7	2,0830	2,0870	3,6600	P21/c	298	2017
QEJJOH	178,4	2,0780	2,0810	3,6600	P21/c	298	2017
QEJJOH	179,3	2,0720	2,0750	3,6600	P21/c	298	2017
QEJJOH	179,1	2,0650	2,0630	3,6600	P21/c	298	2017
QEKBIS	175,0	2,1270	2,1180	5,8200	P-1	200	2006
QEMJEY	173,9	2,1580	2,1720	5,7000	P2/c	295	2006
QEZHEJ	175,4	2,0650	2,0590	4,8200	C2/c	299	2007
QEZHEJ	176,7	2,1030	2,0970	4,8200	C2/c	299	2007
QEZHEJ	179,3	2,0800	2,0740	4,8200	C2/c	299	2007
QIBHIT	173,5	2,1560	2,1560	3,7100	P21/n	150	2007
QIBHIT01	170,7	2,1540	2,1530	4,1400	P21/c	150	2007
QIBHIT01	171,5	2,1830	2,1790	4,1400	P21/c	150	2007
QIFQAA	176,5	2,0920	2,0940	5,0100	P3121	151	2018
QIFQAA	176,8	2,0960	2,0950	5,0100	P3121	151	2018
QIFQAA	170,4	2,0970	2,0900	5,0100	P3121	151	2018
QIFVEI	176,6	2,0720	2,1370	6,9800	P-1	296	2012
QIFVEI	178,6	2,1420	2,0710	6,9800	P-1	296	2012
QIFVEI	177,2	2,1430	2,1460	6,9800	P-1	296	2012
QIPXEU	175,4	2,1140	2,1140	3,5100	C2/c	298	2014
QIPXUK	175,4	2,0720	2,0900	6,8800	P-1	298	2014
QIPYEV	180,0	2,1860	2,1860	4,5900	P-1	293	2014
QIPYIZ	180,0	2,1170	2,1170	5,2400	P21/c	298	2014
QIPYOF	178,9	2,0830	2,1030	8,8000	P-1	298	2014
QIWROG	174,8	2,1730	2,1710	9,2700	P-1	173	2019
QIWSUN	170,9	2,1370	2,1480	14,7300	P-1	173	2019
QIWYIG	174,7	2,1560	2,1560	2,9200	C2/c	293	2013
QIXHAH	176,7	2,1090	2,1100	2,5900	P-1	293	2008
QIYGUB	173,9	2,1500	2,1530	7,3100	P-1	173	2008
QOKBEZ	174,0	2,1390	2,1460	5,3000	C2/c	298	2014
QOTDEK	177,3	2,1230	2,1210	3,3400	P-1	150	2015
QOTDIO	179,4	2,1210	2,1310	8,6800	P-1	296	2015

QOXSOM	172,8	2,0500	2,0610	3,0400	C2/c	296	2009
QQQBUV01	180,0	2,1280	2,1280	3,8000	C2/c	293	2005
QUDMIM	171,3	2,1900	2,1810	5,5900	P21/c	293	2009
QUPJIV01	170,7	2,1560	2,1590	2,9800	P21/n	150	2010
QUPKAO	174,7	2,1490	2,1380	4,3400	P-1	290	2010
QUPKAO01	172,4	2,1340	2,1430	2,8100	P-1	190	2010
QUPKUI	170,2	2,1660	2,1560	4,1800	P21/n	290	2010
QUQBIO	180,0	2,0660	2,0660	2,6200	P21/c	173	2010
QUQBOU	180,0	2,0660	2,0660	3,3300	P21/c	296	2010
QUQBUA	180,0	2,0580	2,0580	2,8600	P21/c	293	2010
QUQCAH	180,0	2,0560	2,0560	5,9200	P21/c	293	2010
QUQCEL	180,0	2,0600	2,0600	6,3000	P21/c	298	2010
QUQCIP	180,0	2,0560	2,0560	3,5600	P21/c	298	2010
QUQCOV	180,0	2,0600	2,0600	2,1300	P21/c	298	2010
RABVOF01	175,8	2,0690	2,0640	2,5200	P21/c	294	2006
RABVOF01	176,6	2,0740	2,0730	2,5200	P21/c	294	2006
RABVOF02	173,5	2,0710	2,0700	2,7200	Pbca	294	2006
RABVOF03	176,6	2,0810	2,0730	3,1500	P212121	293	2005
RABVOF03	176,0	2,0720	2,0610	3,1500	P212121	293	2005
RABVOF03	171,1	2,0760	2,0640	3,1500	P212121	293	2005
RADWAV	176,3	2,1450	2,1440	9,1000	P21/n	100	2004
RAFRUL	170,1	2,2170	2,2280	7,1800	Pa3	295	1997
RAKGIU	171,1	2,1270	2,1340	4,7500	P21/c	296	2005
RAKGIU	177,2	2,1290	2,1250	4,7500	P21/c	296	2005
RANHAQ	180,0	2,1850	2,1850	4,2600	P21/c	294	2005
RANZOV	180,0	2,1700	2,1700	3,9800	C2/c	295	2000
RASGOI	174,6	2,1100	2,1130	3,4700	P-1	173	2005
RAWNAE	170,6	2,1210	2,0890	3,5800	P21/c	150	1996
RAWTOZ	180,0	2,1520	2,1520	4,8000	P-1	293	2005
RAYWUK	172,5	2,1400	2,1380	5,1000	P-1	293	2005
RAYXOF	173,9	2,1620	2,1620	3,2000	Pbcn	293	2005
RAYXOF01	176,7	2,1270	2,1410	4,1000	P61	153	2005
RAYYIA	178,0	2,1550	2,1590	3,8000	P212121	293	2005
RAYYOG	180,0	2,1450	2,1450	2,8000	P-1	293	2005
RAYYOG	180,0	2,1670	2,1670	2,8000	P-1	293	2005
RAYZAT	180,0	2,1300	2,1300	3,9000	P-1	293	2005
RAYZAT	180,0	2,1380	2,1380	3,9000	P-1	293	2005
RAYZEX	174,3	2,1420	2,1480	3,8000	P-1	293	2005
RAYZOH	175,4	2,1730	2,1730	5,3000	C2/c	293	2005
RAZBEA	175,2	2,1230	2,1330	5,0000	P21/c	293	2005
REBYAB	174,2	2,1040	2,1060	3,2300	R-3c	296	2017
REBYAB	176,7	2,1150	2,1170	3,2300	R-3c	296	2017
RECJAL	177,4	2,0970	2,1010	3,1000	Pca21	150	2006
RECJAL	173,6	2,0910	2,1010	3,1000	Pca21	150	2006
RECJIT	173,0	2,1000	2,1060	3,9100	Pbca	150	2006
RECJOZ	170,4	2,1630	2,1600	3,6700	P21/n	150	2006
RECTUP	180,0	2,1150	2,1150	9,3700	C2/c	120	2006
REHJEV	180,0	2,1210	2,1210	5,3900	C2/c	293	2012
REKHOE	170,0	2,2040	2,1920	11,0500	P-1	295	1997
RENNUU	179,0	2,1270	2,1470	10,6000	P21/c	111	2006
REPGAW	180,0	2,0870	2,0870	4,8800	P-1	293	2013
RESFEB	178,9	2,1830	2,1830	5,0100	Pnna	298	2006
REWWUO	176,3	2,0930	2,1000	6,0900	P-1	100	2017
REWWUO	175,6	2,0870	2,0990	6,0900	P-1	100	2017
REWWUO	177,2	2,0810	2,0960	6,0900	P-1	100	2017
REYRUL	171,0	2,1170	2,1280	2,5000	P21/c	133	2018
RIBZIM	180,0	2,1560	2,1560	3,5000	Fddd	295	2007
RICZAH	177,9	2,1090	2,1050	4,6600	C2/c	298	2018
RIDHAQ	174,9	2,1530	2,1420	6,1300	P21/c	298	2018
RIDHEU	178,0	2,1620	2,1650	7,7100	Cmca	298	2018
RIMPUB	179,4	2,1840	2,1830	7,8400	P-1	100	2018
RIRPOZ	180,0	2,1270	2,1270	2,0500	C2/c	120	2014
RIRQEQ	177,3	2,2820	2,2770	2,6600	P-1	120	2014
RIRQEQ	173,7	2,2460	2,2600	2,6600	P-1	120	2014
RISDII	179,3	2,1620	2,1600	3,5400	P21/c	220	2014
RITLIS	178,0	2,0740	2,0430	8,7400	P-1	100	2017
RITLIS	176,2	2,0710	2,0990	8,7400	P-1	100	2017
RITLIS	172,8	2,1230	2,1570	8,7400	P-1	100	2017
RIYNEU	172,0	2,1540	2,1430	8,6000	C2/c	290	2014
RIYXIG	180,0	2,2220	2,2220	4,5300	Pmnn	295	1997
ROFYUJ	180,0	2,1610	2,1610	3,2900	P21/n	296	2019
ROHKIK	176,6	2,1310	2,1330	3,7200	P-1	293	2014
ROJCUO	173,3	2,0730	2,0860	4,5000	P21/n	200	1997
ROPBIJ	171,8	2,1350	2,1530	3,5100	P21	293	2014
ROPBIJ	172,0	2,1360	2,1340	3,5100	P21	293	2014
RUBMAD	180,0	2,1810	2,1810	3,3800	C2/c	293	2009
RUBMAD01	180,0	2,1750	2,1750	4,4800	C2/c	223	2013
SACQEV	180,0	2,1220	2,1220	4,8200	P-1	100	2016

SACQOF	172,3	2,1130	2,0990	7,7600	P21/c 173	2016
SACQUL	176,4	2,0930	2,0940	6,4100	P-1 173	2016
SACREW	174,4	2,0960	2,0980	3,3100	P-1 173	2016
SADWOL	178,1	2,1560	2,1640	3,6000	P21/n 296	2012
SAJKEU	180,0	2,2000	2,2000	7,0900	P-1 193	2004
SAQDEW	170,5	2,1170	2,1260	4,0500	C2/c 293	2017
SAQJEB	180,0	2,1190	2,1190	3,9200	P-1 273	2012
SAQJEB	180,0	2,1270	2,1270	3,9200	P-1 273	2012
SARPEH	174,5	2,1340	2,1350	5,0000	P-1 123	2005
SARPEH	174,3	2,1430	2,1550	5,0000	P-1 123	2005
SARPIL	180,0	2,1870	2,1870	3,1100	C2/c 123	2005
SAXLUA	179,6	2,1360	2,1360	4,1100	Fddd 298	2012
SAXLUA	179,9	2,1210	2,1210	4,1100	Fddd 298	2012
SEDJAN	172,3	2,1200	2,1270	3,9300	P-1 123	2006
SEDJAN	171,5	2,1470	2,1300	3,9300	P-1 123	2006
SEDJER	175,2	2,1510	2,1220	11,3800	P21/c 123	2006
SEDJER	172,5	2,1680	2,1080	11,3800	P21/c 123	2006
SEDJIV	180,0	2,1210	2,1210	5,8200	P-1 123	2006
SEHBEO	180,0	2,1910	2,1910	2,5600	P-1 296	2012
SEHBIS	180,0	2,1590	2,1590	3,2700	C2/c 296	2012
SEHBOY	173,6	2,1720	2,1780	4,4500	P-1 296	2012
SEHBUE	174,1	2,1390	2,1390	4,0100	P-1 296	2012
SEHCAL	174,6	2,1410	2,1410	3,7100	P-1 296	2012
SERVIU	174,0	2,1000	2,1090	3,4000	Pn21a 295	1986
SESSEP	174,6	2,1330	2,1340	4,0200	P21/n 295	2006
SEZCAE	170,1	2,0740	2,1180	7,5600	P-1 296	2018
SEZCAE	180,0	2,0650	2,0650	7,5600	P-1 296	2018
SIDROO	171,0	2,2260	2,0580	5,1300	P21/c 293	2013
SIKKIH	180,0	2,1320	2,1320	6,8200	P21/c 153	2007
SISYAV	178,5	2,1040	2,1120	4,5800	P-1 293	2008
SOJCOM	173,5	2,1060	2,0960	2,5100	P-1 120	2019
SOJCOM	176,6	2,0800	2,0830	2,5100	P-1 120	2019
SOJCUS	175,9	2,0760	2,0810	4,3800	P-1 120	2019
SOJCUS	171,7	2,0850	2,0860	4,3800	P-1 120	2019
SOJCUS	178,5	2,0790	2,0830	4,3800	P-1 120	2019
SOJCUS	170,9	2,0960	2,0970	4,3800	P-1 120	2019
SOJCUS	174,3	2,0850	2,0860	4,3800	P-1 120	2019
SOJCUS	178,5	2,0880	2,0900	4,3800	P-1 120	2019
SOKBEA	170,5	2,1030	2,1070	5,6200	P-1 133	2008
SOKBEA	171,3	2,0930	2,0920	5,6200	P-1 133	2008
SOKBEA	170,9	2,1060	2,1010	5,6200	P-1 133	2008
SOKBEA	171,2	2,0860	2,0870	5,6200	P-1 133	2008
SOMQAM	174,3	2,1440	2,1480	1,8400	P-1 173	1998
SOMQEQ	177,3	2,1470	2,1410	4,8100	P21/c 173	1998
SOMQIU	173,4	2,1590	2,1540	2,4500	P21/c 173	1998
SOXKIC	180,0	2,1660	2,1660	1,5300	P-1 150	2019
SUFTUK	170,3	2,1650	2,1390	7,3200	P21/c 293	2015
SUFTUK	171,7	2,1580	2,1540	7,3200	P21/c 293	2015
SUFTUK	170,9	2,1650	2,1730	7,3200	P21/c 293	2015
SUFTUK	175,3	2,1590	2,1260	7,3200	P21/c 293	2015
SUFTUK	177,1	2,1500	2,1530	7,3200	P21/c 293	2015
SUFTUK	175,7	2,1460	2,1520	7,3200	P21/c 293	2015
SUFTUK	179,2	2,1450	2,1370	7,3200	P21/c 293	2015
SUHCIJ	177,4	2,1060	2,1040	4,4100	P-1 100	2014
SUHCOP	180,0	2,1640	2,1640	4,5000	P-1 100	2014
SUHCOP	180,0	2,2040	2,2040	4,5000	P-1 100	2014
SUHJEL	180,0	2,1220	2,1220	3,1700	P-1 100	2010
SUPZUA	171,8	2,1330	2,1440	5,8900	P-1 293	2015
SUQWOS	170,6	2,1390	2,1500	3,7500	P-1 293	2015
SUSTAC	172,4	2,0970	2,0970	4,3100	C2/c 293	2010
SUXFUO	170,9	2,1190	2,1440	5,5600	P21/c 295	2016
TACYUT	175,1	2,1330	2,1370	4,9500	C2/c 200	2010
TAGNEW	177,2	2,1130	2,1290	3,3400	Pbca 293	2010
TAGNIA	171,9	2,2780	2,2780	4,1500	P2/c 291	2010
TAGNOG	171,9	2,2670	2,2670	2,7100	P2/c 291	2010
TAPLIH	179,0	2,1060	2,1050	2,7000	P-1 100	2011
TAPLUT	179,0	2,0870	2,0910	5,5000	P-1 100	2011
TARRAI	171,3	2,0990	2,1140	4,4700	P-1 293	2017
TARZAN	176,9	2,0930	2,1550	7,6000	P21/n 295	1996
TARZAN	178,1	2,2160	2,1300	7,6000	P21/n 295	1996
TARZAN01	178,6	2,1470	2,1420	5,7000	P2/n 295	1996
TARZAN01	178,1	2,1490	2,1540	5,7000	P2/n 295	1996
TARZAN02	178,8	2,1440	2,1450	3,9000	C2/m 295	1996
TARZIV	177,4	2,1270	2,1320	5,5000	C2/c 295	1996
TARZIV	177,9	2,1640	2,1840	5,5000	C2/c 295	1996
TASFEA	173,4	2,0880	2,0840	4,7000	P21/c 100	2012
TASFOK	170,6	2,1080	2,1250	2,2800	P-1 100	2012
TASFUQ	171,7	2,0950	2,0970	3,6700	C2/c 100	2012

TASGAX	172,3	2,1090	2,1070	4,1500	P-1	100	2012
TASHEC	177,8	2,1130	2,1420	4,0400	P-1	100	2012
TASPEK	177,3	2,1190	2,1110	6,6900	P-1	150	2012
TEBXUU	178,5	2,1920	2,1790	9,5200	P21/c	273	2005
TEBXUU	172,6	2,2040	2,2070	9,5200	P21/c	273	2005
TEBYEF	176,1	2,1400	2,1790	5,7300	Pn	273	2005
TEBYEF	172,3	2,1460	2,2060	5,7300	Pn	273	2005
TENDAU	180,0	2,2420	2,2420	8,2300	P-1	296	2017
TEPFUR	174,0	2,1540	2,1540	10,5300	Ibam	123	2013
TEPFUR	178,0	2,1320	2,1320	10,5300	Ibam	123	2013
TEPFUR	172,2	2,1530	2,1430	10,5300	Ibam	123	2013
TEPFUR	174,4	2,1580	2,1580	10,5300	Ibam	123	2013
TEPFUR	175,4	2,1260	2,1260	10,5300	Ibam	123	2013
TEPGAY	173,0	2,1390	2,1390	8,4300	P42/mbc	298	2013
TEPGAY	171,2	2,1600	2,1600	8,4300	P42/mbc	298	2013
TEPWES	174,7	2,1270	2,1410	4,1300	P21/c	200	2013
TEQRIR	172,7	2,1800	2,1620	4,8800	P21/c	168	2006
TERMOT	173,1	2,1300	2,1300	4,7800	C2/c	293	2006
TEVROE	174,0	2,1000	2,0850	2,5700	P21/c	93	2018
TEVROE	171,9	2,0750	2,0950	2,5700	P21/c	93	2018
TEVRUK	174,0	2,1050	2,0930	5,7300	Pna21	93	2018
TEVRUK	170,2	2,1180	2,1070	5,7300	Pna21	93	2018
TEWPIW	171,7	2,1120	2,1450	6,1900	P1	298	2012
TIGHEY	176,2	2,1090	2,0990	1,5300	P21212	293	2013
TIJFOH	175,0	2,1360	2,1330	6,3000	P-1	293	2001
TIJFOH	180,0	2,1410	2,1410	6,3000	P-1	293	2001
TIJFOH	180,0	2,1760	2,1760	6,3000	P-1	293	2001
TISHIN	177,7	2,1110	2,0990	5,7800	Fdd2	294	2007
TIVXIG	178,8	2,0860	2,0860	6,6100	C2/m	292	2008
TIVXIG	170,9	2,1420	2,1420	6,6100	C2/m	292	2008
TIVXIG	178,4	2,1140	2,1140	6,6100	C2/m	292	2008
TIZCIQ	178,4	2,0790	2,0850	3,0800	P-1	133	2014
TIZCIQ	179,5	2,0670	2,0750	3,0800	P-1	133	2014
TIZCIQ	177,6	2,0790	2,0880	3,0800	P-1	133	2014
TIZCOW	179,6	2,0750	2,0760	4,3900	P-1	133	2014
TIZCOW	177,3	2,0910	2,0850	4,3900	P-1	133	2014
TIZCOW	177,0	2,0740	2,0860	4,3900	P-1	133	2014
TODYOD	174,7	2,0740	2,1090	9,0400	P32	100	2019
TODYOD	177,3	2,1140	2,0790	9,0400	P32	100	2019
TODYOD	173,3	2,1380	2,1060	9,0400	P32	100	2019
TOGXAR	179,1	2,1320	2,1420	5,2000	Pbcn	294	2019
TOHJUX	173,4	2,1600	2,1800	7,7200	P-1	173	2014
TOLBUS	178,2	2,1300	2,1370	2,4600	P-1	100	2008
TOLCAZ	178,7	2,1330	2,1400	2,1200	P-1	100	2008
TOLCIH	177,1	2,1070	2,1040	2,7800	P-1	100	2008
TOLDAA	177,9	2,1370	2,1310	5,5500	P-1	100	2008
TORWAZ	178,2	2,1380	2,1300	2,8300	P-1	100	2008
TORWAZ01	178,6	2,1380	2,1310	2,8900	P-1	100	2008
TUTQEF	177,4	2,0980	2,0970	6,7200	P21/c	296	2010
UBOGEX	175,4	2,1090	2,1090	3,4600	C2/c	297	2001
UBOGEX	172,9	2,1170	2,1170	3,4600	C2/c	297	2001
UBOGIB	178,9	2,0890	2,0760	3,4100	P-1	297	2001
UBOGIB	174,9	2,0870	2,0890	3,4100	P-1	297	2001
UBOGIB	175,8	2,1120	2,1120	3,4100	P-1	297	2001
UBONEE	177,0	2,1520	2,1540	3,6600	P21/n	173	2001
UBUKUA	171,4	2,0990	2,0990	4,0500	Pccn	100	2017
UBULAH	170,1	2,1420	2,1490	4,6700	P21/n	295	2017
UCOHUP	177,1	2,1220	2,1560	5,7000	P21	293	2001
UFALEV	180,0	2,1930	2,1930	10,1100	Pbca	293	2018
UGADAI	180,0	2,1120	2,1120	3,5300	C2/c	100	2008
UGADAI	176,0	2,1410	2,1420	3,5300	C2/c	100	2008
UGEKOH	176,3	2,1310	2,1310	7,8500	Ibca	293	2009
UGEKUN	173,1	2,1260	2,1260	8,0200	Ibca	293	2009
UHEXOV	178,4	2,1510	2,1490	3,4900	R-3c	173	2009
UHEXUB	179,4	2,1470	2,1490	4,1600	R-3c	173	2009
UHEYAI	172,0	2,1840	2,1840	2,3100	C2/c	173	2009
UHEYEM	176,1	2,1360	2,1350	3,7900	P-1	173	2009
UKAVIL	172,4	2,0590	2,0610	3,4700	P-1	150	2003
UKAVIL	170,6	2,0650	2,0640	3,4700	P-1	150	2003
UKOVOG	173,5	2,1370	2,1240	6,0900	Pca21	293	2008
UKOVOG	173,7	2,1600	2,1510	6,0900	Pca21	293	2008
ULAGET	170,9	2,1300	2,1360	2,5200	P-1	150	2003
ULOHOT	174,2	2,1830	2,1790	3,5500	P212121	113	2011
ULOJUB	178,1	2,1540	2,1620	4,5300	P21	113	2011
ULOJUB	175,4	2,1190	2,1190	4,5300	P21	113	2011
UMOCII	173,7	2,1400	2,1300	5,7100	P-1	293	2003
UMOCII	177,8	2,1450	2,1360	5,7100	P-1	293	2003
UMODOQ	170,9	2,1270	2,1310	6,9800	C2/c	173	2011

UMOFAE	171,9	2,1320	2,1530	3,3600	P-1	173	2011
UMOFIM	171,0	2,1460	2,1680	4,4900	P-1	173	2011
UMOFIM01	171,2	2,1210	2,1410	3,6600	P21/n	173	2011
UMOFUY	174,7	2,1170	2,1280	3,8600	P21/c	173	2011
UMOFUY	171,8	2,1170	2,1150	3,8600	P21/c	173	2011
UNABIW	175,9	2,1260	2,1260	7,3500	C2/m	173	2016
UNABIW	176,1	2,1550	2,1550	7,3500	C2/m	173	2016
UNOKAK	170,3	2,1170	2,1180	3,9800	P-1	293	2011
UNONER	176,5	2,1000	2,1020	4,2300	P21/c	100	2011
UNURAY	175,2	2,1450	2,1490	4,7900	R-3c	293	2016
UNUREC	178,6	2,1580	2,1580	4,9900	Pnma	100	2016
UNURIG	178,3	2,2390	2,2390	6,6300	C2/c	173	2016
UTAFAX	174,0	2,2000	2,1540	10,3700	Cc	150	2011
UTAFAX	174,6	2,1900	2,1310	10,3700	Cc	150	2011
UTAFAX	176,9	2,1400	2,1070	10,3700	Cc	150	2011
UTAFAX	173,4	2,1520	2,1340	10,3700	Cc	150	2011
UWIHUE	180,0	2,1330	2,1330	8,7300	C2/m	123	2011
UWIHUE	179,6	2,1800	2,1800	8,7300	C2/m	123	2011
UWIKUH	178,0	2,1530	2,1690	6,1500	C2	293	2011
UWIKUH	178,0	2,1570	2,1610	6,1500	C2	293	2011
UWIKUH01	178,1	2,1550	2,1660	0,0000	Fdd2	293	2014
UWULII	170,6	2,1050	2,1020	4,3800	Pna21	100	2011
UWULII	172,2	2,1190	2,1180	4,3800	Pna21	100	2011
UWULII	172,1	2,1220	2,1230	4,3800	Pna21	100	2011
UWULOO	175,9	2,0980	2,0940	4,8600	P-1	100	2011
UWULOO	176,7	2,0930	2,0950	4,8600	P-1	100	2011
UWULOO	179,0	2,1180	2,1140	4,8600	P-1	100	2011
UXIWIJ	176,8	2,1420	2,1420	3,8700	Fdd2	293	2016
UYEKEP	173,4	2,1550	2,1510	3,4600	Pbca	295	2011
VAHYUA	175,3	2,0800	2,0730	4,5100	C2	293	2010
VAHYUA	174,7	2,0430	2,0550	4,5100	C2	293	2010
VAPDIB	178,1	2,1650	2,1650	4,0200	Pccn	296	2012
VARKIL	170,6	2,1470	2,1490	5,3300	P21/n	295	2017
VATXUL	171,7	2,1960	2,1870	5,7600	C2/c	296	2012
VATYAS	174,4	2,2160	2,1990	5,6100	C2/c	296	2012
VATYEW	172,9	2,2220	2,2080	3,9000	C2/c	296	2012
VAYWEZ	180,0	2,1720	2,1720	3,4200	P21/c	293	2012
VAYWEZ	172,5	2,1490	2,1290	3,4200	P21/c	293	2012
VAYWID	180,0	2,1150	2,1150	4,2100	P21/c	293	2012
VAYWID	174,3	2,1220	2,1490	4,2100	P21/c	293	2012
VAZFUY	174,9	2,1180	2,1210	3,2000	P-1	295	2005
VECXOQ	177,8	2,1040	2,1020	4,2600	P21/c	173	1998
VECXOQ	176,4	2,1060	2,1040	4,2600	P21/c	173	1998
VECXOQ	179,1	2,1340	2,1300	4,2600	P21/c	173	1998
VEFHEU	174,5	2,1550	2,1550	4,0300	C2/c	298	2006
VEFVAF	177,5	2,1730	2,1770	9,2900	P21	296	2012
VEFVAF	172,6	2,1890	2,1880	9,2900	P21	296	2012
VEJSUZ	172,0	2,4300	2,4180	2,7500	P21/c	200	2006
VEJTEK	170,8	2,6390	2,5810	3,2400	P21/n	200	2006
VERWEX	175,3	2,1570	2,1490	6,7700	Pc	100	2018
VERWEX	179,0	2,1770	2,1470	6,7700	Pc	100	2018
VETGUX	180,0	2,1760	2,1760	4,1500	C2/c	293	2006
VIGDEW	178,3	2,1470	2,1300	6,2300	P21/n	298	2012
VIGNAB	180,0	2,2030	2,2030	6,9100	P-1	200	2007
VIKFUT	180,0	2,1170	2,1170	11,8900	P-1	120	2018
VIKGEE	180,0	2,1450	2,1450	7,3000	P-1	168	2018
VINVAR	174,2	2,1260	2,1200	2,4700	P-1	293	2013
VINVIZ	180,0	2,1010	2,1010	5,2600	P-1	100	2013
VINZID	180,0	2,1420	2,1420	9,6600	P-1	293	2013
VINZID	180,0	2,1970	2,1970	9,6600	P-1	293	2013
VIRDAC	178,8	2,1310	2,1080	9,0900	P-1	153	2007
VIZBAH	180,0	2,1240	2,1240	2,5000	P-1	223	1991
VIZBAH	180,0	2,1270	2,1270	2,5000	P-1	223	1991
VODCUN	174,2	2,1100	2,1140	5,9100	P21/a	293	2008
VOFQEO	180,0	2,0930	2,0930	7,2600	C2/c	296	2013
VOFQEO	178,3	2,0980	2,0940	7,2600	C2/c	296	2013
VOHMOX	180,0	2,1590	2,1590	7,1700	P-1	293	2019
VOLKAL	174,2	2,1130	2,1220	2,9600	P2/c	100	2019
VOLWEB	175,0	2,0750	2,0740	7,9300	P-1	296	2019
VOLWIF	180,0	2,1210	2,1210	7,2300	P21/c	296	2019
VONBEH	180,0	2,1580	2,1580	7,5100	P-1	297	2014
VUCQUG	174,6	2,1530	2,1190	2,6000	Pbca	150	2009
VUCRAN	173,8	2,1360	2,1070	2,4600	P-1	150	2009
VUDKAI	180,0	2,0860	2,0860	8,1500	P21/c	173	2015
VUDKAI	180,0	2,0980	2,0980	8,1500	P21/c	173	2015
VULVUU	175,6	2,1280	2,1280	4,5700	Cmc21	293	2009
VUVGUQ	172,1	2,1290	2,1390	4,1000	P-1	298	2015
WAFWAD	173,8	2,1280	2,1160	7,9500	P-1	296	2010

WAFWEH	180,0	2,0960	2,0960	4,4800	P21/c	296	2010
WAFWEH	180,0	2,1190	2,1190	4,4800	P21/c	296	2010
WAGHUK	174,0	2,1420	2,1360	4,7100	P-1	293	2016
WATKOU	172,0	2,1600	2,1390	7,2900	P-1	296	2017
WATKUA	175,5	2,1620	2,1440	7,5600	P-1	296	2017
WAZLUG	172,5	2,1390	2,1010	6,8100	P21/c	293	2012
WAZNIW	170,5	2,1840	2,2010	5,3800	P-1	298	2012
WEBNEZ	180,0	2,1370	2,1370	5,8200	P-1	298	2014
WEGBUI	177,5	2,1220	2,1240	9,2300	C2	98	2017
WEHTAH	178,2	2,1100	2,1100	5,5700	P321	170	2017
WEJTUC	178,6	2,0750	2,0950	5,0300	P21/n	296	2012
WEJVAK	170,9	2,0860	2,1090	5,9900	P-1	296	2012
WEJVEO	173,2	2,0850	2,0810	8,6700	P-1	296	2012
WEXJUG	171,6	2,1120	2,1060	3,3800	P-1	298	2013
WEYDAI	170,1	2,1710	2,1800	7,6100	P21/c	273	2018
WIFBAP	180,0	2,1750	2,1750	2,8100	C2/c	133	2007
WIHWER	171,3	2,1480	2,1340	5,5300	P21/c	295	2013
WIMGIL	175,3	2,1000	2,1020	3,4700	P21/n	293	2018
WIMTAO	178,4	2,0840	2,0850	7,9300	I41/acd	293	2007
WINZUP	176,4	2,1220	2,1220	4,5900	C2221	293	2007
WITREY	178,8	2,1600	2,1600	10,4100	Ibam	120	2014
WITSEZ	175,4	2,1490	2,1490	6,0100	P2/c	291	2014
WIVHAK	176,8	2,1260	2,1220	5,2500	C2/c	295	2000
WIWKUK	173,8	2,1570	2,1570	8,7100	C2	123	2014
WOGQUF	175,9	2,1400	2,1360	2,0100	P-1	293	2008
WOHKAG	176,4	2,1380	2,1380	2,3900	C2/c	173	2008
WOHKAG01	176,4	2,1300	2,1300	2,1000	C2/c	173	2018
WOLSAS	176,0	2,2050	2,1690	5,7900	I-4	150	2008
WOLVEA	175,2	2,1960	2,1940	8,0900	P-1	153	2014
WUFMIT	176,2	2,1270	2,1340	5,8800	P-1	293	2002
WUFMOZ	178,0	2,1590	2,1270	6,3700	P21/c	293	2002
WUFMOZ	176,0	2,1370	2,1310	6,3700	P21/c	293	2002
XABJIV	176,8	2,0890	2,1050	8,1100	P-1	298	2010
XABJIV	174,9	2,1060	2,1000	8,1100	P-1	298	2010
XAVNUD	174,9	2,0890	2,0960	5,9200	Pa-3	153	2000
XAVNUD	173,6	2,0840	2,0830	5,9200	Pa-3	153	2000
XAVNUD	175,4	2,0920	2,1070	5,9200	Pa-3	153	2000
XAVNUD	176,1	2,0900	2,1010	5,9200	Pa-3	153	2000
XAZFEJ	180,0	2,2480	2,2480	3,8600	Pnnm	295	2000
XAZGIR	180,0	2,1390	2,1390	6,3500	P21/c	150	2011
XECZUB01	171,0	2,1750	2,1680	2,7100	P-1	173	2007
XEDZOV	173,1	2,1400	2,1370	3,0600	P21/c	295	2000
XEDZUB	172,5	2,1380	2,1450	5,4700	P21/c	295	2000
XEFBAL	172,8	2,1480	2,1500	3,7200	P21/c	295	2000
XEFBEP	172,6	2,1350	2,1240	3,5200	P21/n	295	2000
XEFDAO	175,6	2,2400	2,2280	3,5700	Cc	293	2006
XEGKOK	170,7	2,1310	2,1310	1,7000	C2	150	2006
XEKQOV	171,0	2,1510	2,1510	3,4500	C2/c	296	2013
XELXER	178,4	2,0820	2,0820	3,6800	C2/c	295	2000
XELXER	178,6	2,0760	2,0660	3,6800	C2/c	295	2000
XELXER	175,6	2,0840	2,0850	3,6800	C2/c	295	2000
XICGEW	171,8	2,1880	2,1720	2,9900	P-1	293	2007
XICGIC	171,5	2,1370	2,0810	7,0200	P-1	293	2018
XICGIC	175,1	2,0900	2,0780	7,0200	P-1	293	2018
XICSEK	175,4	2,1500	2,1510	4,4300	P-1	293	2016
XIDCOF	170,3	2,1610	2,1490	5,9300	P-1	293	2016
XIHNAG	174,5	2,0690	2,0650	7,5800	P-1	293	2018
XIHNAG	172,6	2,1060	2,0900	7,5800	P-1	293	2018
XINRER	180,0	2,3110	2,3110	5,0800	P-1	293	2002
XINRER	173,9	2,1490	2,1720	5,0800	P-1	293	2002
XIQPAO	178,0	2,1270	2,1320	3,4400	Cc	173	2001
XIRCIL	176,4	2,0980	2,0910	4,1200	P21	293	2007
XITJES	180,0	2,1460	2,1460	1,9800	P21/c	150	2019
XITNIZ	180,0	2,1570	2,1570	6,4000	P21/c	294	2014
XOBSAI	172,8	2,1240	2,1250	5,0400	P21/a	293	2002
XOBSAI	171,4	2,1460	2,1290	5,0400	P21/a	293	2002
XOGJUA	177,9	2,0500	2,0500	4,4000	C2	298	2014
XOHKOW	174,0	2,1070	2,1070	2,4500	C2/c	293	2014
XOKFOU	171,1	2,0830	2,0830	4,4800	C2/c	298	2014
XOKFUA	172,5	2,1000	2,1090	2,6500	P21/n	293	2014
XOKGEL	173,0	2,0580	2,0580	5,4800	C2/c	293	2014
XOKTAT	175,2	2,0730	2,0750	3,8700	P-1	293	2008
XOKTAT	173,8	2,1120	2,1090	3,8700	P-1	293	2008
XOLTAT	176,7	2,1480	2,1680	4,9200	P21	293	2002
XOMVON	177,3	2,0620	2,0590	1,4800	P-1	178	2019
XOMVON	178,4	2,0690	2,0720	1,4800	P-1	178	2019
XOMVON	177,8	2,0630	2,0600	1,4800	P-1	178	2019
XOMWAA	177,4	2,0690	2,0690	5,7600	P21/c	178	2019

XOMWAA	177,0	2,0570	2,0470	5,7600	P21/c	178	2019
XOMWAA	178,4	2,0750	2,0810	5,7600	P21/c	178	2019
XOPREB	177,4	2,1560	2,1590	6,6000	P21/c	296	2014
XUBBAY	180,0	2,1740	2,1740	3,3900	C2/c	293	2009
XUFSAU	174,9	2,1100	2,1120	1,8300	P21/c	100	2015
XUGMIX	171,4	2,1110	2,0990	6,1500	C2/c	293	2015
XUGMIX	175,2	2,1150	2,1150	6,1500	C2/c	293	2015
XUJSAX	179,1	2,1490	2,2150	4,7900	P1	298	2009
XUKWEG	180,0	2,0930	2,0930	5,1700	P-1	298	2009
XUKXIL	174,7	2,1050	2,1090	5,0700	C2/c	150	2009
XUNPEC	178,5	2,1070	2,1190	5,5400	P21/n	223	2009
XUNPEC	176,8	2,0850	2,0810	5,5400	P21/n	223	2009
XUNPEC	174,9	2,1140	2,1180	5,5400	P21/n	223	2009
XUPPOO	177,9	2,1390	2,1190	9,5400	P63/m	93	2010
YADFOA	176,2	2,0810	2,0810	6,4500	C2/c	293	2011
YALKIH	175,0	2,0980	2,0990	4,2700	P-1	293	2011
YARHOQ	173,7	2,1240	2,1260	2,5300	P-1	100	2012
YARHUW	173,5	2,1290	2,1300	2,6400	P-1	100	2012
YARJAE	172,1	2,1320	2,1220	6,5400	P21/m	100	2012
YASJIN	173,9	2,2500	2,2460	4,8600	P-1	293	2012
YASKAG	177,5	2,2180	2,2300	8,8200	P-1	296	2012
YASMEL	171,5	2,1460	2,1500	1,8400	P-1	100	2005
YECTIK	173,7	2,1480	2,1530	5,2800	P-1	150	2006
YECTOQ	172,0	2,1650	2,1610	6,8200	P-1	150	2006
YEGNAA	175,8	2,1520	2,1470	3,8800	P21/n	150	2006
YEKQOV	177,7	2,0530	2,0480	3,1900	P21/c	100	2006
YEKQOV	172,9	2,0610	2,0560	3,1900	P21/c	100	2006
YEKQOV01	174,8	2,0810	2,0870	3,6100	P-1	214	2010
YEKQOV01	174,7	2,0840	2,0720	3,6100	P-1	214	2010
YEKQOV01	170,1	2,0620	2,0680	3,6100	P-1	214	2010
YEKQOV01	172,3	2,0550	2,0450	3,6100	P-1	214	2010
YEKQOV01	175,3	2,0690	2,0550	3,6100	P-1	214	2010
YEKQUB01	173,0	2,0710	2,0580	9,4500	I41/a	208	2016
YEKQUB01	171,0	2,0800	2,0870	9,4500	I41/a	208	2016
YEKQUB01	171,9	2,0950	2,0830	9,4500	I41/a	208	2016
YEKRAI	178,2	2,1050	2,1280	3,6900	P21	100	2006
YEKRAI	173,9	2,1220	2,1190	3,6900	P21	100	2006
YEKRAI	170,5	2,0980	2,1090	3,6900	P21	100	2006
YEKRAI	174,2	2,1150	2,1100	3,6900	P21	100	2006
YEKRAI	175,0	2,1010	2,0720	3,6900	P21	100	2006
YEKRAI	171,3	2,1210	2,1110	3,6900	P21	100	2006
YEVQUM	175,0	2,0370	2,0450	7,9900	I41/a	153	2007
YEVQUM	178,9	2,0700	2,0650	7,9900	I41/a	153	2007
YEVQUM	170,2	2,0470	2,0650	7,9900	I41/a	153	2007
YEVQUM	172,4	2,0690	2,0680	7,9900	I41/a	153	2007
YEVQUM	174,0	2,0570	2,0370	7,9900	I41/a	153	2007
YEVQUM	173,8	2,0400	2,0450	7,9900	I41/a	153	2007
YEVQUM	178,0	2,0660	2,0450	7,9900	I41/a	153	2007
YEVQUM	174,9	2,0440	2,0740	7,9900	I41/a	153	2007
YEVQUM	171,5	2,0810	2,0690	7,9900	I41/a	153	2007
YEVQUM	174,8	2,0650	2,0420	7,9900	I41/a	153	2007
YEVQUM	171,7	2,0830	2,0630	7,9900	I41/a	153	2007
YEVQUM	170,0	2,0800	2,0740	7,9900	I41/a	153	2007
YEVQUM	176,4	2,0460	2,0410	7,9900	I41/a	153	2007
YEVQUM	177,8	2,0600	2,0450	7,9900	I41/a	153	2007
YEVQUM	176,2	2,0680	2,0610	7,9900	I41/a	153	2007
YEVQUM	171,6	2,0880	2,0780	7,9900	I41/a	153	2007
YEWGEP	180,0	2,1280	2,1280	4,8300	P-1	200	2018
YEYVOO	172,4	2,0730	2,0640	7,7700	I41/a	153	2007
YEYVOO	173,0	2,0420	2,0830	7,7700	I41/a	153	2007
YEYVOO	171,4	2,0660	2,0720	7,7700	I41/a	153	2007
YEYVOO	172,3	2,0810	2,0660	7,7700	I41/a	153	2007
YEYVOO	170,5	2,0680	2,0810	7,7700	I41/a	153	2007
YEYVOO	173,2	2,0780	2,0760	7,7700	I41/a	153	2007
YEYVOO	176,3	2,0360	2,0350	7,7700	I41/a	153	2007
YEYVOO	177,9	2,0260	2,0600	7,7700	I41/a	153	2007
YEYVOO	178,6	2,0490	2,0370	7,7700	I41/a	153	2007
YEYVOO	172,4	2,0670	2,0440	7,7700	I41/a	153	2007
YEYVOO	175,1	2,0590	2,0500	7,7700	I41/a	153	2007
YEYVOO	176,6	2,0560	2,0530	7,7700	I41/a	153	2007
YEYVOO	174,0	2,0420	2,0570	7,7700	I41/a	153	2007
YEYVOO	177,4	2,0600	2,0870	7,7700	I41/a	153	2007
YEYVOO	171,3	2,0390	2,0760	7,7700	I41/a	153	2007
YEYVOO	175,7	2,0630	2,0800	7,7700	I41/a	153	2007
YEYVOO	172,9	2,0540	2,0570	7,7700	I41/a	153	2007
YIBZAM	176,1	2,1170	2,1080	6,8500	P-1	180	2013
YIBZAM	176,8	2,0860	2,0910	6,8500	P-1	180	2013
YIBZEQ	176,0	2,0830	2,0890	3,1000	P-1	180	2013

YIHYUL	176,0	2,1190	2,1240	3,3900	P-1	293	2013
YIHYUL01	176,0	2,1190	2,1240	3,3900	P-1	293	2014
YIQKUH	178,4	2,1680	2,1850	6,9300	P21/n	298	2018
YIQLOB	178,9	2,1630	2,1330	4,4700	C2/c	203	2010
YIQLUH	177,4	2,1520	2,1540	5,6200	P21/c	293	2010
YIRYIK	172,8	2,1710	2,1800	1,5500	P-1	110	2018
YIWJUK	171,2	2,1500	2,1500	2,8300	Fddd	295	2008
YOFDUU	171,6	2,1350	2,1440	2,2900	P21/c	93	2014
YOHKEN	176,6	2,0870	2,0520	10,0800	Pbcm	293	2014
YOHKEN	171,7	2,1210	2,1250	10,0800	Pbcm	293	2014
YOSXEK	174,1	2,1220	2,1270	4,4400	P-1	292	2009
YOTWEL	180,0	2,0100	2,0100	7,8700	P-1	293	2014
YOTWIP	171,7	2,1620	2,1730	4,5400	P-1	293	2014
YUCNIU	170,7	2,1590	2,1590	3,2700	Cmc21	153	2009
YUCYAX	174,6	2,1710	2,1470	3,6900	P21/n	120	2009
YUDQUK	172,0	2,2280	2,2280	4,0800	P2/c	291	2009
YULSIJ	170,8	2,1180	2,1250	3,5800	P21/n	293	2015
YULSIJ	172,7	2,0920	2,0860	3,5800	P21/n	293	2015
ZAWJIQ	180,0	2,0990	2,0990	3,8000	P-1	173	1995
ZAWKOZ	170,6	2,1400	2,1360	2,5900	P-1	100	2012
ZAXRUO	173,4	2,1530	2,1560	2,7400	P21/c	173	2016
ZAZYAD	173,7	2,1220	2,1350	3,4200	P-1	296	2017
ZAZYAD	174,0	2,1240	2,1370	3,4200	P-1	296	2017
ZECXEM	179,5	2,1010	2,1340	3,2600	C2/c	187	2012
ZECXIQ	180,0	2,0530	2,0530	3,3500	P-1	187	2012
ZECXIQ	170,2	2,1100	2,1080	3,3500	P-1	187	2012
ZECXUC	175,3	2,1000	2,1290	3,8300	P-1	187	2012
ZECXUC	170,1	2,1270	2,1200	3,8300	P-1	187	2012
ZECYAJ	174,9	2,1160	2,1260	3,1300	P-1	187	2012
ZEDFOG	174,7	2,1630	2,1680	8,8900	P21/a	93	2017
ZEHNAD	178,5	2,1190	2,1360	2,6000	P-1	298	2012
ZEHNOR	177,3	2,1100	2,1480	3,5000	P-1	298	2012
ZENBUS	172,6	2,1390	2,1450	4,0000	R-3	296	2017
ZEVXEF	170,7	2,1580	2,1580	11,0700	Pnna	150	2013
ZEVXEF	171,8	2,0890	2,0890	11,0700	Pnna	150	2013
ZIQCAF	175,6	2,0920	2,0960	4,2500	P21/n	296	2013
ZOZBEX	178,0	2,0770	2,0920	6,4800	P21/n	113	2015
ZUJPUR	177,8	2,1280	2,1420	7,4200	C2/c	294	2014
ZUJQAY	176,1	2,1040	2,1160	7,7900	C2/c	294	2014
ZULJOH	176,9	2,1340	2,1210	4,7000	P-1	296	2015
ZULJUN	178,6	2,1730	2,1340	4,4200	P21/c	296	2015
ZUWVEU	177,0	2,1470	2,1420	6,2500	P21/n	100	2016

Table S20. N-Au-N Bond angles and N-Au bond distances in the related structures X-ray structures available in the CSD. Only close to linear (180° ± 10°), nonpolymeric structures not having additional metal coordination are considered.

Ref. code	Angle	N(1)-Au (Å)	N(2)-Au (Å)	Δ(N-Au)	%	R	SpGr	Temp	Pub. Year
AXENUO	179,4	2,0230	2,0230	0,0000	0,0000	1,7600	Pbcn	100	2014
AXENUO01	177,2	2,0250	2,0250	0,0000	0,0000	1,5300	P-1	100	2014
BUVTUI	178,9	2,0390	2,0500	-0,0110	-0,5366	3,8800	P21/n	200	2010
DOGRIB	175,3	2,0560	2,0610	-5,0000e-3	-0,2426	7,9400	Pbca	93	2008
GOGDIQ	180,0	2,0300	2,0300	0,0000	0,0000	4,3800	C2/m	298	2008
KANJIS	173,8	2,0080	2,0350	-0,0270	-1,3268	3,7100	P21/n	173	2000
PIZJOY	177,7	2,0220	2,0220	0,0000	0,0000	2,7200	C2/c	273	2008
PIZJUE	180,0	2,0070	2,0070	0,0000	0,0000	3,4800	P-1	273	2008
PIZJUE	177,1	2,0070	2,0120	-5,0000e-3	-0,2485	3,4800	P-1	273	2008
PIZJUE	180,0	2,0110	2,0110	0,0000	0,0000	3,4800	P-1	273	2008
PIZKEP	180,0	2,0060	2,0060	0,0000	0,0000	7,5300	P-1	296	2008
PIZKEP	180,0	2,0210	2,0210	0,0000	0,0000	7,5300	P-1	296	2008
PIZKEP	180,0	2,0680	2,0680	0,0000	0,0000	7,5300	P-1	296	2008
PIZKEP	180,0	2,0540	2,0540	0,0000	0,0000	7,5300	P-1	296	2008
PIZKEP01	180,0	2,0160	2,0160	0,0000	0,0000	2,2600	P21/n	100	2013
PIZKEP01	180,0	2,0220	2,0220	0,0000	0,0000	2,2600	P21/n	100	2013
PIZKEP02	180,0	2,0140	2,0140	0,0000	0,0000	3,5000	P21/n	300	2013
PIZKEP02	180,0	2,0190	2,0190	0,0000	0,0000	3,5000	P21/n	300	2013
PIZKEP03	180,0	2,0130	2,0130	0,0000	0,0000	3,1200	P21/n	200	2013
PIZKEP03	180,0	2,0330	2,0330	0,0000	0,0000	3,1200	P21/n	200	2013
PIZKIT	178,0	2,0120	2,0170	-5,0000e-3	-0,2479	5,0800	P-1	298	2008
PIZKIT	177,7	2,0150	2,0100	5,0000e-3	0,2488	5,0800	P-1	298	2008
PIZKIT	176,2	2,0170	2,0170	0,0000	0,0000	5,0800	P-1	298	2008
PIZKOZ	180,0	2,2090	2,2090	0,0000	0,0000	2,2600	P21/n	273	2008
RILZEU	177,3	2,0140	2,0260	-0,0120	-0,5923	2,8000	C2/c	100	2018
RILZEU	175,3	2,0310	2,0310	0,0000	0,0000	2,8000	C2/c	100	2018
RILZEU01	175,4	2,0220	2,0360	-0,0140	-0,6876	2,5600	Cc	100	2018
RILZEU01	176,1	2,0230	2,0210	2,0000e-3	0,0990	2,5600	Cc	100	2018
UJOQAN	176,2	2,0220	2,0190	3,0000e-3	0,1486	3,5500	Ccca	173	2016
ZINTIB	180,0	2,0190	2,0190	0,0000	0,0000	2,4600	P21/n	100	2013
ZINTIB	180,0	2,0260	2,0260	0,0000	0,0000	2,4600	P21/n	100	2013

Table S21. N-Au-N Bond angles and N-Au bond distances in the related structures X-ray structures available in the CSD. Only close to linear (180° ± 10°), nonpolymeric structures not having additional metal coordination are considered.

Ref. code	Angle	N(1)-Au (Å)	N(2)-Au (Å)	Δ(N-Au)	%	R	SpGr	Temp	Pub. Year
DUDTUU	174,32	1,9940	2,0190	-0,0250	-1,2382	7,3700	P2/c	298	2019
DUDVAC	173,62	2,0060	2,0080	-2,0000e-3	-0,0996	3,8200	P-1	296	2019
JUDSOQ01	178,80	1,9970	1,9720	0,0250	1,2677	6,1400	P-1	296	2019
JUDSOQ01	177,51	1,9900	1,9900	0,0000	0,0000	6,1400	P-1	296	2019
JUDSOQ01	177,34	1,9930	1,9850	8,0000e-3	0,4030	6,1400	P-1	296	2019
MOXMEU	179,13	2,0010	2,0020	-1,0000e-3	-0,0500	1,5200	C2/c	220	2019
MOXMEU	179,80	2,0040	2,0040	0,0000	0,0000	1,5200	C2/c	220	2019
XUHFOY	179,58	1,9790	1,9920	-0,0130	-0,6526	4,1300	P21/n	296	2019
XUHFOY	177,96	1,9980	1,9940	4,0000e-3	0,2006	4,1300	P21/n	296	2019
XUHFOY	177,76	1,9900	1,9920	-2,0000e-3	-0,1004	4,1300	P21/n	296	2019
XUHVII	176,98	2,0320	1,9550	0,0770	3,9386	5,0500	P-1	296	2019
XUHVII	176,44	2,0240	1,9620	0,0620	3,1600	5,0500	P-1	296	2019
XUHVII	177,84	1,9830	2,0030	-0,0200	-0,9985	5,0500	P-1	296	2019
AHAHAS	179,15	1,9910	1,9950	-4,0000e-3	-0,2005	4,9900	P21/n	298	2002
AHAHEW	178,69	1,9910	1,9740	0,0170	0,8612	3,9900	P-1	298	2002
AHAHEW	179,27	1,9740	1,9680	6,0000e-3	0,3049	3,9900	P-1	298	2002
AHAHEW	176,07	1,9820	1,9910	-9,0000e-3	-0,4520	3,9900	P-1	298	2002
AHAHEW	179,64	2,0230	1,9950	0,0280	1,4035	3,9900	P-1	298	2002
AHAHIA	179,43	1,9790	1,9560	0,0230	1,1759	3,8700	P-1	298	2002
AHAHOG	178,35	1,9940	2,0010	-7,0000e-3	-0,3498	4,4200	Pca21	298	2002
AHAHOG	178,45	2,0080	1,9970	0,0110	0,5508	4,4200	Pca21	298	2002
ATOWEL	174,21	2,0080	1,9630	0,0450	2,2924	8,4200	P-1	173	2004
ATOWEL	178,60	2,0000	2,0400	-0,0400	-1,9608	8,4200	P-1	173	2004
ATOWEL	175,29	1,9980	1,9740	0,0240	1,2158	8,4200	P-1	173	2004
AWAWOM	174,08	1,9930	2,0060	-0,0130	-0,6481	2,5700	Fd-3c	100	2016
AXENUO	179,40	2,0230	2,0230	0,0000	0,0000	1,7600	Pbcn	100	2014
AXENUO01	177,21	2,0250	2,0250	0,0000	0,0000	1,5300	P-1	100	2014
BAQYOH	180,00	2,0410	2,0410	0,0000	0,0000	2,8700	C2/c	173	1999
BUVTUI	178,93	2,0390	2,0500	-0,0110	-0,5366	3,8800	P21/n	200	2010
CAQTUJ	179,58	2,0520	2,0520	0,0000	0,0000	3,0700	C2/c	173	1999
CAQTUJ	175,01	2,0560	2,0520	4,0000e-3	0,1949	3,0700	C2/c	173	1999
CENFUY	178,83	2,0010	1,9970	4,0000e-3	0,2003	2,5400	C2/c	298	2013
CENFUY	178,11	2,0090	2,0030	6,0000e-3	0,2996	2,5400	C2/c	298	2013
CENFUY	179,53	2,0160	2,0010	0,0150	0,7496	2,5400	C2/c	298	2013
CILFUA	174,28	2,0000	1,9970	3,0000e-3	0,1502	5,0000	P21/c	293	2013
CILFUA	174,36	2,0070	1,9810	0,0260	1,3125	5,0000	P21/c	293	2013
CILFUA	179,39	2,0120	2,0000	0,0120	0,6000	5,0000	P21/c	293	2013
CILFUA	177,84	2,0210	1,9890	0,0320	1,6088	5,0000	P21/c	293	2013
CILFUA	178,57	1,9990	1,9810	0,0180	0,9086	5,0000	P21/c	293	2013
CILFUA	177,60	2,0050	2,0070	-2,0000e-3	-0,0997	5,0000	P21/c	293	2013
CILFUA01	179,74	2,0060	2,0080	-2,0000e-3	-0,0996	3,8700	C2/c	293	2013
CILFUA01	173,32	2,0010	1,9860	0,0150	0,7553	3,8700	C2/c	293	2013
CILFUA01	178,09	1,9920	1,9800	0,0120	0,6061	3,8700	C2/c	293	2013
CILFUA02	173,24	2,0000	1,9940	6,0000e-3	0,3009	3,2600	C2/c	100	2013
CILFUA02	179,68	2,0030	1,9980	5,0000e-3	0,2503	3,2600	C2/c	100	2013
CILFUA02	178,85	1,9980	2,0120	-0,0140	-0,6958	3,2600	C2/c	100	2013
CILFUA03	173,88	1,9880	1,9970	-9,0000e-3	-0,4507	4,8400	P21/c	100	2013
CILFUA03	173,95	2,0030	2,0040	-1,0000e-3	-0,0499	4,8400	P21/c	100	2013
CILFUA03	179,20	2,0060	2,0050	1,0000e-3	0,0499	4,8400	P21/c	100	2013
CILFUA03	178,00	2,0120	1,9840	0,0280	1,4113	4,8400	P21/c	100	2013
CILFUA03	177,06	1,9900	1,9830	7,0000e-3	0,3530	4,8400	P21/c	100	2013
CILFUA03	177,87	2,0110	1,9400	0,0710	3,6598	4,8400	P21/c	100	2013
COFRAS	178,22	2,0060	2,0080	-2,0000e-3	-0,0996	4,1000	C2/c	298	2014
COFRAS	179,21	1,9980	2,0050	-7,0000e-3	-0,3491	4,1000	C2/c	298	2014
COFRAS	179,16	2,0000	2,0090	-9,0000e-3	-0,4480	4,1000	C2/c	298	2014
COFREW	175,38	2,0440	2,0030	0,0410	2,0469	9,3800	P21/c	298	2014
COFREW	177,67	2,0150	2,0140	1,0000e-3	0,0497	9,3800	P21/c	298	2014
COFREW	177,97	2,0340	2,0250	9,0000e-3	0,4444	9,3800	P21/c	298	2014
COHFIO	179,54	1,9100	1,8870	0,0230	1,2189	6,6000	P-1	295	1984
COHFIO	178,67	1,9330	1,9480	-0,0150	-0,7700	6,6000	P-1	295	1984
COHFIO	178,12	1,9550	1,9330	0,0220	1,1381	6,6000	P-1	295	1984
COHFIO01	179,89	1,9950	1,9950	0,0000	0,0000	2,0400	P-1	100	2005
COHFIO01	179,46	1,9950	1,9900	5,0000e-3	0,2513	2,0400	P-1	100	2005
COHFIO01	179,47	2,0030	2,0010	2,0000e-3	0,1000	2,0400	P-1	100	2005
DAXQOJ	176,93	2,0010	1,9950	6,0000e-3	0,3008	2,5500	P-1	100	2005
DAXQOJ	176,32	2,0090	1,9860	0,0230	1,1581	2,5500	P-1	100	2005
DAXQOJ	176,04	1,9960	2,0010	-5,0000e-3	-0,2499	2,5500	P-1	100	2005
DAXQOJ	176,86	1,9990	2,0020	-3,0000e-3	-0,1499	2,5500	P-1	100	2005
DAXQOJ	175,69	2,0050	2,0160	-0,0110	-0,5456	2,5500	P-1	100	2005
DAXQOJ	177,11	2,0060	2,0050	1,0000e-3	0,0499	2,5500	P-1	100	2005
DOGRIB	175,28	2,0560	2,0610	-5,0000e-3	-0,2426	7,9400	Pbca	93	2008
DOHVIG	176,29	1,9980	1,9930	5,0000e-3	0,2509	1,8900	P-1	143	2008

DOHVOM	178,01	2,0140	2,0100	4,0000e-3	0,1990	4,5400	Fdd2	143	2008
DOHVUS	176,59	2,0010	1,9990	2,0000e-3	0,1001	2,1800	P21/n	143	2008
DUHQAY	177,21	2,0520	2,0550	-3,0000e-3	-0,1460	2,1000	P-1	143	2000
DUHQEC	176,43	2,0440	2,0290	0,0150	0,7393	4,3000	Pna21	173	2000
DUHQUS	179,30	2,1030	2,0980	5,0000e-3	0,2383	7,3700	P-1	143	2000
DUHQUS01	179,58	2,1050	2,1060	-1,0000e-3	-0,0475	6,7300	C2/m	143	2003
EHUCER	180,00	2,0030	2,0030	0,0000	0,0000	3,0300	C2/c	173	2016
EWOKEI	175,68	2,0170	2,0260	-9,0000e-3	-0,4442	4,7400	P21	123	2016
EWOKEI	175,78	2,0240	2,0310	-7,0000e-3	-0,3447	4,7400	P21	123	2016
FEJJAF10	174,69	2,0610	2,0290	0,0320	1,5771	7,8000	P21/n	295	1988
FEJJAF10	178,91	2,1350	2,1570	-0,0220	-1,0199	7,8000	P21/n	295	1988
FEJJAF10	175,54	2,0020	2,0220	-0,0200	-0,9891	7,8000	P21/n	295	1988
FEJJAF10	179,34	2,0560	2,0670	-0,0110	-0,5322	7,8000	P21/n	295	1988
FEJJAF10	176,65	2,0320	2,0580	-0,0260	-1,2634	7,8000	P21/n	295	1988
FEJJAF10	175,88	2,0510	2,0430	8,0000e-3	0,3916	7,8000	P21/n	295	1988
FIMSEB	180,00	2,0560	2,0560	0,0000	0,0000	2,2500	P-1	100	2013
FIYQUA	178,26	2,0210	2,0160	5,0000e-3	0,2480	3,4800	Cc	100	2005
FIYQUA	178,81	1,9880	1,9660	0,0220	1,1190	3,4800	Cc	100	2005
FIYQUA	178,52	2,0000	2,0070	-7,0000e-3	-0,3488	3,4800	Cc	100	2005
FORLEE	179,34	2,0170	2,0200	-3,0000e-3	-0,1485	4,2300	Pna21	296	2009
FORLII	177,17	1,9880	1,9920	-4,0000e-3	-0,2008	9,7900	P21/c	296	2009
FORLOO	177,76	1,9880	1,9980	-0,0100	-0,5005	3,5700	P21/c	293	2009
FORLOO	178,07	2,0010	2,0050	-4,0000e-3	-0,1995	3,5700	P21/c	293	2009
FUWXOK	179,53	1,9760	1,9760	0,0000	0,0000	3,5000	R-3c	295	1988
FUWXOK01	179,94	1,9930	1,9930	0,0000	0,0000	1,5100	R-3c	100	2014
FUWXOK02	179,91	1,9950	1,9950	0,0000	0,0000	1,4900	R-3c	120	2014
FUWXOK03	179,90	1,9950	1,9950	0,0000	0,0000	1,5700	R-3c	150	2014
FUWXOK04	179,94	1,9950	1,9950	0,0000	0,0000	1,6600	R-3c	180	2014
FUWXOK05	179,91	1,9950	1,9950	0,0000	0,0000	1,7100	R-3c	210	2014
FUWXOK06	179,95	1,9960	1,9960	0,0000	0,0000	1,7700	R-3c	240	2014
FUWXOK07	179,95	1,9930	1,9930	0,0000	0,0000	1,8600	R-3c	270	2014
FUWXOK08	179,83	1,9930	1,9940	-1,0000e-3	-0,0502	2,6400	R-3c	293	2014
FUWXOK09	179,72	1,9940	1,9950	-1,0000e-3	-0,0501	3,3800	R-3c	293	2014
FUWXOK10	179,72	1,9840	1,9850	-1,0000e-3	-0,0504	3,9900	R-3c	293	2014
FUWXOK11	179,95	1,9900	1,9910	-1,0000e-3	-0,0502	3,0000	R-3c	293	2014
FUWXOK12	179,92	1,9910	1,9920	-1,0000e-3	-0,0502	2,9500	R-3c	293	2014
FUWXOK13	179,73	1,9830	1,9830	0,0000	0,0000	3,2300	R-3c	293	2014
FUWXOK14	179,81	1,9820	1,9830	-1,0000e-3	-0,0504	3,2000	R-3c	293	2014
FUWXOK15	179,89	1,9790	1,9800	-1,0000e-3	-0,0505	2,8600	R-3c	293	2014
FUXDIN	177,29	2,0040	1,9920	0,0120	0,6024	4,5600	P21/n	100	2014
FUXDIN	175,66	1,9960	2,0170	-0,0210	-1,0412	4,5600	P21/n	100	2014
FUXDIN	175,44	1,9980	1,9990	-1,0000e-3	-0,0500	4,5600	P21/n	100	2014
FUXDIN	178,06	2,0040	2,0070	-3,0000e-3	-0,1495	4,5600	P21/n	100	2014
FUXDIN	175,18	2,0130	1,9990	0,0140	0,7004	4,5600	P21/n	100	2014
FUXDIN	174,32	2,0020	1,9980	4,0000e-3	0,2002	4,5600	P21/n	100	2014
FUXDOT	171,62	2,0090	2,0120	-3,0000e-3	-0,1491	5,8600	C2/c	100	2014
FUXDOT	176,73	2,0040	2,0070	-3,0000e-3	-0,1495	5,8600	C2/c	100	2014
FUXDOT	177,81	2,0160	1,9960	0,0200	1,0020	5,8600	C2/c	100	2014
GAFJOO	176,27	2,0060	1,9790	0,0270	1,3643	9,7200	P-1	212	2010
GAFJOO	174,89	2,0460	2,0110	0,0350	1,7404	9,7200	P-1	212	2010
GAFJOO	175,65	2,0130	2,0230	-0,0100	-0,4943	9,7200	P-1	212	2010
GAFJOO	174,67	2,0410	2,0040	0,0370	1,8463	9,7200	P-1	212	2010
GAFJOO	175,46	1,9890	2,0080	-0,0190	-0,9462	9,7200	P-1	212	2010
GAFJOO	174,33	2,0020	2,0080	-6,0000e-3	-0,2988	9,7200	P-1	212	2010
GAFJUU	178,90	1,9490	1,9970	-0,0480	-2,4036	6,1000	I41/a	218	2010
GAFKAB	178,77	2,0270	2,0370	-0,0100	-0,4909	10,6700	P-1	184	2010
GAFKAB	177,64	2,0350	2,0150	0,0200	0,9926	10,6700	P-1	184	2010
GAFKAB	178,06	2,0520	1,9880	0,0640	3,2193	10,6700	P-1	184	2010
GAFKAB	179,65	2,0310	2,0070	0,0240	1,1958	10,6700	P-1	184	2010
GEXJUO	178,97	1,9970	2,0140	-0,0170	-0,8441	3,9800	P21/n	295	1988
GEXJUO	178,20	1,9830	1,9950	-0,0120	-0,6015	3,9800	P21/n	295	1988
GIJTAV	174,94	2,0300	2,0330	-3,0000e-3	-0,1476	3,2000	P21/c	100	2007
GIJTAV	175,93	2,0360	2,0290	7,0000e-3	0,3450	3,2000	P21/c	100	2007
GIJTEZ	175,17	2,0270	2,0250	2,0000e-3	0,0988	2,6200	P21/n	100	2007
GOGDIQ	180,00	2,0300	2,0300	0,0000	0,0000	4,3800	C2/m	298	2008
GOGFEN	179,04	2,0500	2,0550	-5,0000e-3	-0,2433	3,0200	C2/c	143	1998
GOMWEL	177,61	2,0560	2,0590	-3,0000e-3	-0,1457	6,2200	P-1	299	2009
HAPGAI	179,14	1,9630	2,0400	-0,0770	-3,7745	9,9700	Pbca	100	2012
HAPGAI	178,29	1,9800	1,9710	9,0000e-3	0,4566	9,9700	Pbca	100	2012
HAPGAI	179,42	2,0060	1,9420	0,0640	3,2956	9,9700	Pbca	100	2012
HAPGEM	175,39	2,0180	2,0170	1,0000e-3	0,0496	7,4900	P-1	100	2012
HAPGEM	179,02	2,0060	2,0050	1,0000e-3	0,0499	7,4900	P-1	100	2012
HAPGEM	175,57	2,0060	2,0080	-2,0000e-3	-0,0996	7,4900	P-1	100	2012
HEMTID	175,49	1,9860	1,9920	-6,0000e-3	-0,3012	2,5300	C2/c	95	2006
HEMTID	177,07	1,9930	2,0020	-9,0000e-3	-0,4496	2,5300	C2/c	95	2006
HEMTID	176,77	2,0010	1,9950	6,0000e-3	0,3008	2,5300	C2/c	95	2006
HEMTID01	175,81	1,9730	1,9700	3,0000e-3	0,1523	7,3800	C2/c	293	2006
HEMTID01	175,51	2,0150	2,0320	-0,0170	-0,8366	7,3800	C2/c	293	2006

HEMTID01	172,91	1,9670	1,9630	4,0000e-3	0,2038	7,3800	C2/c	293	2006
HEMTID02	175,95	2,0030	2,0080	-5,0000e-3	-0,2490	3,9200	C2/c	296	2016
HEMTID02	176,19	2,0030	1,9990	4,0000e-3	0,2001	3,9200	C2/c	296	2016
HEMTID02	176,41	2,0060	2,0010	5,0000e-3	0,2499	3,9200	C2/c	296	2016
HEMTID03	176,33	1,9990	2,0040	-5,0000e-3	-0,2495	5,5700	C2/c	293	2016
HEMTID03	176,57	1,9860	1,9870	-1,0000e-3	-0,0503	5,5700	C2/c	293	2016
HEMTID03	177,26	1,9680	2,0070	-0,0390	-1,9432	5,5700	C2/c	293	2016
HEMTID04	176,10	1,9890	1,9890	0,0000	0,0000	5,4900	C2/c	293	2016
HEMTID04	177,38	1,9550	2,0060	-0,0510	-2,5424	5,4900	C2/c	293	2016
HEMTID04	177,16	1,9870	1,9050	0,0820	4,3045	5,4900	C2/c	293	2016
HEMTID05	175,76	2,0400	2,0050	0,0350	1,7456	4,6600	C2/c	293	2016
HEMTID05	176,28	1,9350	1,9920	-0,0570	-2,8614	4,6600	C2/c	293	2016
HEMTID05	177,06	2,0080	1,9980	0,0100	0,5005	4,6600	C2/c	293	2016
HEMTID06	175,63	1,9840	1,9740	0,0100	0,5066	4,0800	C2/c	293	2016
HEMTID06	176,66	1,9730	1,9810	-8,0000e-3	-0,4038	4,0800	C2/c	293	2016
HEMTID06	177,15	1,9490	1,9820	-0,0330	-1,6650	4,0800	C2/c	293	2016
HEMTID07	176,20	1,9650	1,9850	-0,0200	-1,0076	5,6400	P21/n	293	2016
HEMTID07	175,47	1,9660	1,9900	-0,0240	-1,2060	5,6400	P21/n	293	2016
HEMTID07	176,00	1,9750	1,9960	-0,0210	-1,0521	5,6400	P21/n	293	2016
HEMTID07	177,28	2,0020	1,9950	7,0000e-3	0,3509	5,6400	P21/n	293	2016
HEMTID07	176,03	1,9710	1,9770	-6,0000e-3	-0,3035	5,6400	P21/n	293	2016
HEMTID07	177,28	1,9870	1,9800	7,0000e-3	0,3535	5,6400	P21/n	293	2016
HEMTID08	172,57	1,9510	2,0000	-0,0490	-2,4500	4,9500	C2/c	293	2016
HEMTID08	176,42	1,9780	1,9790	-1,0000e-3	-0,0505	4,9500	C2/c	293	2016
HEMTID08	174,86	1,9960	1,9930	3,0000e-3	0,1505	4,9500	C2/c	293	2016
HEMTID08	175,98	1,9960	1,9830	0,0130	0,6556	4,9500	C2/c	293	2016
HEMTID08	176,95	1,9980	2,0020	-4,0000e-3	-0,1998	4,9500	C2/c	293	2016
HEMTID08	174,87	2,0000	1,9880	0,0120	0,6036	4,9500	C2/c	293	2016
HEMTID08	174,14	1,9820	1,9970	-0,0150	-0,7511	4,9500	C2/c	293	2016
HEMTID08	175,57	1,9840	1,9910	-7,0000e-3	-0,3516	4,9500	C2/c	293	2016
HEMTID08	177,51	1,9970	2,0020	-5,0000e-3	-0,2498	4,9500	C2/c	293	2016
HEMTID09	177,45	1,9770	1,9770	0,0000	0,0000	6,9300	P21/n	293	2016
HEMTID09	172,12	1,9850	1,9890	-4,0000e-3	-0,2011	6,9300	P21/n	293	2016
HEMTID09	177,22	2,0020	1,9940	8,0000e-3	0,4012	6,9300	P21/n	293	2016
HEMTID09	179,20	2,0150	1,9940	0,0210	1,0532	6,9300	P21/n	293	2016
HEMTID09	174,91	1,9440	2,0120	-0,0680	-3,3797	6,9300	P21/n	293	2016
HEMTID09	176,46	1,9790	2,0140	-0,0350	-1,7378	6,9300	P21/n	293	2016
HEMTID10	176,02	1,9860	1,9840	2,0000e-3	0,1008	3,4600	C2/c	296	2016
HEMTID10	175,68	1,9950	1,9810	0,0140	0,7067	3,4600	C2/c	296	2016
HEMTID10	176,13	1,9910	1,9840	7,0000e-3	0,3528	3,4600	C2/c	296	2016
HEMTID11	176,68	1,9960	1,9680	0,0280	1,4228	4,8100	C2/c	296	2016
HEMTID11	178,68	2,0000	1,9860	0,0140	0,7049	4,8100	C2/c	296	2016
HEMTID11	176,29	2,0040	2,0020	2,0000e-3	0,0999	4,8100	C2/c	296	2016
HEMTID12	176,83	2,0070	1,9850	0,0220	1,1083	3,8300	C2/c	296	2016
HEMTID12	176,89	2,0090	2,0050	4,0000e-3	0,1995	3,8300	C2/c	296	2016
HEMTID12	174,63	2,0040	1,9700	0,0340	1,7259	3,8300	C2/c	296	2016
HEMTID13	177,38	2,0050	1,9910	0,0140	0,7032	4,3200	C2/c	296	2016
HEMTID13	175,54	2,0030	1,9920	0,0110	0,5522	4,3200	C2/c	296	2016
HEMTID13	173,03	2,0190	1,9760	0,0430	2,1761	4,3200	C2/c	296	2016
HEMTID14	176,99	2,0130	1,9760	0,0370	1,8725	5,1900	C2/c	296	2016
HEMTID14	177,60	1,9930	1,9930	0,0000	0,0000	5,1900	C2/c	296	2016
HEMTID14	176,43	1,9920	1,9870	5,0000e-3	0,2516	5,1900	C2/c	296	2016
HEMTID15	178,35	2,0010	1,9970	4,0000e-3	0,2003	6,7200	C2/c	296	2016
HEMTID15	177,30	1,9990	2,0120	-0,0130	-0,6461	6,7200	C2/c	296	2016
HEMTID15	174,57	1,9820	2,0120	-0,0300	-1,4911	6,7200	C2/c	296	2016
HEMTID16	172,85	1,9890	1,9920	-3,0000e-3	-0,1506	5,4000	C2/c	296	2016
HEMTID16	176,66	1,9950	2,0100	-0,0150	-0,7463	5,4000	C2/c	296	2016
HEMTID16	176,07	1,9720	1,9800	-8,0000e-3	-0,4040	5,4000	C2/c	296	2016
HEMTID17	173,67	1,9780	1,9930	-0,0150	-0,7526	6,1100	C2/c	296	2016
HEMTID17	172,33	1,9810	2,0280	-0,0470	-2,3176	6,1100	C2/c	296	2016
HEMTID17	176,96	1,9760	2,0050	-0,0290	-1,4464	6,1100	C2/c	296	2016
HEMTID18	170,34	1,8870	1,9510	-0,0640	-3,2804	13,2000	P21/c	293	2016
HEMTID18	174,92	1,9740	2,0150	-0,0410	-2,0347	13,2000	P21/c	293	2016
HEMTID18	176,59	1,9790	2,0010	-0,0220	-1,0995	13,2000	P21/c	293	2016
HEMTID18	175,60	2,0120	1,9660	0,0460	2,3398	13,2000	P21/c	293	2016
HEMTID18	177,14	1,8920	2,0440	-0,1520	-7,4364	13,2000	P21/c	293	2016
HEMTID18	177,22	1,9550	2,0010	-0,0460	-2,2989	13,2000	P21/c	293	2016
HEMTID18	174,26	2,0400	2,0080	0,0320	1,5936	13,2000	P21/c	293	2016
HEMTID18	174,17	2,0750	1,9600	0,1150	5,8673	13,2000	P21/c	293	2016
HEMTID18	170,87	1,9000	1,9280	-0,0280	-1,4523	13,2000	P21/c	293	2016
HEMTID18	174,65	1,9580	1,9600	-2,0000e-3	-0,1020	13,2000	P21/c	293	2016
HEMTID18	178,10	1,9940	2,0480	-0,0540	-2,6367	13,2000	P21/c	293	2016
HEMTID18	177,37	1,9950	1,9880	7,0000e-3	0,3521	13,2000	P21/c	293	2016
HOHJOF	179,30	1,9880	1,9880	0,0000	0,0000	9,4100	C2/m	90	2014
HOHJOF	178,26	2,0220	2,0320	-0,0100	-0,4921	9,4100	C2/m	90	2014
HOHJUL	178,08	1,9990	2,0230	-0,0240	-1,1864	4,2000	Pnma	90	2014
HOHJUL	179,20	2,0110	2,0110	0,0000	0,0000	4,2000	Pnma	90	2014
IMIJAQ	177,73	1,9970	2,0030	-6,0000e-3	-0,2996	1,7400	P-1	140	2011

JOSNOW	178,21	2,0140	1,9850	0,0290	1,4610	4,8900	Pna21	210	2014
JOSNOW	179,13	1,9980	2,0060	-8,0000e-3	-0,3988	4,8900	Pna21	210	2014
JOSNOW	175,85	2,0020	2,0190	-0,0170	-0,8420	4,8900	Pna21	210	2014
JOSNOW01	178,43	2,0290	1,9990	0,0300	1,5008	4,6600	Pna21	100	2014
JOSNOW01	178,79	2,0180	2,0320	-0,0140	-0,6890	4,6600	Pna21	100	2014
JOSNOW01	176,99	2,0290	2,0130	0,0160	0,7948	4,6600	Pna21	100	2014
JOSNOW02	178,79	1,9870	1,9360	0,0510	2,6343	4,0300	Pna21	100	2014
JOSNOW02	178,44	1,9950	2,0000	-5,0000e-3	-0,2500	4,0300	Pna21	100	2014
JOSNOW02	177,74	1,9940	2,0180	-0,0240	-1,1893	4,0300	Pna21	100	2014
JOSNOW03	177,94	2,0030	1,9810	0,0220	1,1106	4,6800	Pna21	150	2014
JOSNOW03	179,02	1,9960	2,0120	-0,0160	-0,7952	4,6800	Pna21	150	2014
JOSNOW03	175,96	2,0080	2,0330	-0,0250	-1,2297	4,6800	Pna21	150	2014
JOSNOW04	178,25	2,0050	1,9920	0,0130	0,6526	4,9300	Pna21	120	2014
JOSNOW04	178,69	1,9880	2,0050	-0,0170	-0,8479	4,9300	Pna21	120	2014
JOSNOW04	176,83	2,0090	2,0320	-0,0230	-1,1319	4,9300	Pna21	120	2014
JOSNOW05	178,63	2,0160	1,9980	0,0180	0,9009	3,7900	Pna21	100	2014
JOSNOW05	179,24	1,9930	2,0000	-7,0000e-3	-0,3500	3,7900	Pna21	100	2014
JOSNOW05	177,05	2,0040	2,0170	-0,0130	-0,6445	3,7900	Pna21	100	2014
JOSNOW06	178,43	2,0290	1,9990	0,0300	1,5008	4,6600	Pna21	100	2014
JOSNOW06	178,79	2,0180	2,0320	-0,0140	-0,6890	4,6600	Pna21	100	2014
JOSNOW06	176,99	2,0290	2,0130	0,0160	0,7948	4,6600	Pna21	100	2014
JOSNOW07	178,17	2,0040	2,0050	-1,0000e-3	-0,0499	3,0700	Pna21	270	2014
JOSNOW07	178,66	2,0080	2,0130	-5,0000e-3	-0,2484	3,0700	Pna21	270	2014
JOSNOW07	176,76	2,0100	2,0070	3,0000e-3	0,1495	3,0700	Pna21	270	2014
JOSNOW08	178,18	2,0130	2,0020	0,0110	0,5495	3,1100	Pna21	240	2014
JOSNOW08	178,72	2,0180	2,0080	0,0100	0,4980	3,1100	Pna21	240	2014
JOSNOW08	176,34	2,0100	2,0170	-7,0000e-3	-0,3471	3,1100	Pna21	240	2014
JOSNUC	178,75	2,0140	1,9880	0,0260	1,3078	3,5900	Pna21	180	2014
JOSNUC	179,23	1,9910	2,0070	-0,0160	-0,7972	3,5900	Pna21	180	2014
JOSNUC	177,19	2,0090	2,0030	6,0000e-3	0,2996	3,5900	Pna21	180	2014
JUDSOQ	177,05	1,9760	1,9670	9,0000e-3	0,4575	5,5000	P-1	295	1998
JUDSOQ	176,87	1,9840	1,9690	0,0150	0,7618	5,5000	P-1	295	1998
JUDSOQ	178,47	1,9770	1,9940	-0,0170	-0,8526	5,5000	P-1	295	1998
KANJIS	173,82	2,0080	2,0350	-0,0270	-1,3268	3,7100	P21/n	173	2000
KEDVOH	170,57	2,0520	2,0470	5,0000e-3	0,2443	3,1900	P-1	200	2017
KESDOC	173,14	2,0240	2,0240	0,0000	0,0000	2,7400	C2/c	100	2006
KIGLOB	176,60	2,0300	2,0480	-0,0180	-0,8789	5,8000	P21/n	295	1990
KIGLOB	177,39	2,0070	1,9810	0,0260	1,3125	5,8000	P21/n	295	1990
KISMUX	178,93	2,0030	2,0180	-0,0150	-0,7433	7,5300	P-1	293	2019
KISMUX	177,23	1,9890	2,0030	-0,0140	-0,6990	7,5300	P-1	293	2019
KISMUX	179,28	1,9110	1,9810	-0,0700	-3,5336	7,5300	P-1	293	2019
LALPOD	178,80	2,0100	1,9990	0,0110	0,5503	3,3000	P21/n	295	1993
LIYBUR	175,41	2,0260	2,0140	0,0120	0,5958	3,3500	P-1	293	2007
LIYBUR	174,39	2,0170	2,0150	2,0000e-3	0,0993	3,3500	P-1	293	2007
LIYBUR	172,83	2,0300	2,0170	0,0130	0,6445	3,3500	P-1	293	2007
LIYBUR	172,27	2,0300	2,0170	0,0130	0,6445	3,3500	P-1	293	2007
LIYCAY	176,56	1,9800	2,0220	-0,0420	-2,0772	10,7200	P-1	110	2007
LIYCAY	177,02	1,9720	2,0050	-0,0330	-1,6459	10,7200	P-1	110	2007
LIYCAY	176,58	2,0350	1,9980	0,0370	1,8519	10,7200	P-1	110	2007
LIYCAY	175,12	1,9950	1,9930	2,0000e-3	0,1004	10,7200	P-1	110	2007
LIYCAY	177,22	2,0390	2,0110	0,0280	1,3923	10,7200	P-1	110	2007
LIYCAY	177,40	2,0300	1,9950	0,0350	1,7544	10,7200	P-1	110	2007
LIYCAY	177,68	2,0370	2,0250	0,0120	0,5926	10,7200	P-1	110	2007
LIYCAY	176,56	1,9850	2,0170	-0,0320	-1,5865	10,7200	P-1	110	2007
MAGLUE	176,25	1,9800	1,9890	-9,0000e-3	-0,4525	4,3500	P-1	293	2016
MAGLUE	175,74	1,9980	2,0070	-9,0000e-3	-0,4484	4,3500	P-1	293	2016
MAGMAL	175,80	1,9920	1,9930	-1,0000e-3	-0,0502	2,0600	P21/c	93	2016
MAGMAL	174,30	1,9850	2,0040	-0,0190	-0,9481	2,0600	P21/c	93	2016
MAGMIT	171,49	1,9970	1,9950	2,0000e-3	0,1003	1,8300	P-1	93	2016
MUTKUH	176,87	2,0010	2,0140	-0,0130	-0,6455	2,1600	P21/c	298	2003
MUTKUH	178,87	2,0070	2,0100	-3,0000e-3	-0,1493	2,1600	P21/c	298	2003
MUTKUH	177,43	1,9910	2,0000	-9,0000e-3	-0,4500	2,1600	P21/c	298	2003
MUTKUH01	177,83	1,9940	1,9950	-1,0000e-3	-0,0501	2,8200	P21/c	100	2014
MUTKUH02	178,01	1,9840	1,9950	-0,0110	-0,5514	2,2700	P21/c	293	2014
MUTKUH02	175,97	1,9680	1,9840	-0,0160	-0,8065	2,2700	P21/c	293	2014
MUTKUH02	178,25	1,9900	2,0060	-0,0160	-0,7976	2,2700	P21/c	293	2014
MUTKUH03	177,77	1,9850	2,0020	-0,0170	-0,8492	2,6400	P21/c	293	2014
MUTKUH03	177,99	2,0020	1,9950	7,0000e-3	0,3509	2,6400	P21/c	293	2014
MUTKUH03	175,19	1,9450	1,9880	-0,0430	-2,1630	2,6400	P21/c	293	2014
MUTKUH04	177,83	2,0030	1,9980	5,0000e-3	0,2503	2,9000	P21/c	293	2014
MUTKUH04	178,04	1,9770	2,0000	-0,0230	-1,1500	2,9000	P21/c	293	2014
MUTKUH04	174,97	1,9400	1,9950	-0,0550	-2,7569	2,9000	P21/c	293	2014
MUTKUH05	178,33	1,9860	1,9880	-2,0000e-3	-0,1006	2,3100	P21/c	293	2014
MUTKUH05	177,60	1,9780	2,0320	-0,0540	-2,6575	2,3100	P21/c	293	2014
MUTKUH05	175,58	1,9610	1,9660	-5,0000e-3	-0,2543	2,3100	P21/c	293	2014
MUTKUH06	178,49	1,9900	1,9770	0,0130	0,6576	2,1700	P21/c	293	2014
MUTKUH06	176,67	1,9470	2,0330	-0,0860	-4,2302	2,1700	P21/c	293	2014
MUTKUH06	176,11	1,9850	1,9660	0,0190	0,9664	2,1700	P21/c	293	2014

MUTKUH07	179,14	1,9820	1,9760	6,0000e-3	0,3036	2,0400	P21/c	293	2014
MUTKUH07	177,84	1,9720	2,0040	-0,0320	-1,5968	2,0400	P21/c	293	2014
MUTKUH07	174,74	1,9760	1,9900	-0,0140	-0,7035	2,0400	P21/c	293	2014
MUTKUH08	178,47	1,9800	1,9790	1,0000e-3	0,0505	2,1000	P21/c	293	2014
MUTKUH08	178,02	1,9750	1,9780	-3,0000e-3	-0,1517	2,1000	P21/c	293	2014
MUTKUH08	174,63	1,9770	1,9890	-0,0120	-0,6033	2,1000	P21/c	293	2014
MUTKUH09	178,01	2,0010	2,0060	-5,0000e-3	-0,2493	4,1100	P21/c	180	2014
MUTKUH09	178,56	1,9960	2,0070	-0,0110	-0,5481	4,1100	P21/c	180	2014
MUTKUH09	176,21	2,0080	1,9970	0,0110	0,5508	4,1100	P21/c	180	2014
MUTKUH10	178,01	2,0070	2,0060	1,0000e-3	0,0499	3,8300	P21/c	210	2014
MUTKUH10	178,35	2,0050	2,0080	-3,0000e-3	-0,1494	3,8300	P21/c	210	2014
MUTKUH10	176,87	2,0010	2,0110	-0,0100	-0,4973	3,8300	P21/c	210	2014
MUTKUH11	177,41	1,9910	1,9990	-8,0000e-3	-0,4002	4,1700	P21/c	240	2014
MUTKUH11	178,82	2,0110	2,0110	0,0000	0,0000	4,1700	P21/c	240	2014
MUTKUH11	176,41	2,0010	2,0060	-5,0000e-3	-0,2493	4,1700	P21/c	240	2014
MUTKUH12	177,85	1,9910	1,9880	3,0000e-3	0,1509	4,3500	P21/c	270	2014
MUTKUH12	178,56	2,0020	1,9660	0,0360	1,8311	4,3500	P21/c	270	2014
MUTKUH12	176,80	1,9910	2,0350	-0,0440	-2,1622	4,3500	P21/c	270	2014
MUTKUH13	177,82	1,9890	1,9960	-7,0000e-3	-0,3507	4,8400	P21/c	293	2014
MUTKUH13	178,99	1,9950	2,0060	-0,0110	-0,5484	4,8400	P21/c	293	2014
MUTKUH13	176,61	2,0090	2,0080	1,0000e-3	0,0498	4,8400	P21/c	293	2014
MUTKUH14	177,98	1,9950	2,0050	-0,0100	-0,4988	3,7500	P21/c	100	2014
MUTKUH14	178,94	1,9870	2,0140	-0,0270	-1,3406	3,7500	P21/c	100	2014
MUTKUH14	176,63	2,0040	2,0120	-8,0000e-3	-0,3976	3,7500	P21/c	100	2014
MUTKUH15	178,29	1,9970	1,9760	0,0210	1,0628	4,0900	P21/c	120	2014
MUTKUH15	178,72	2,0130	2,0040	9,0000e-3	0,4491	4,0900	P21/c	120	2014
MUTKUH15	176,62	2,0090	2,0100	-1,0000e-3	-0,0498	4,0900	P21/c	120	2014
MUTKUH16	178,02	1,9970	2,0020	-5,0000e-3	-0,2498	3,9800	P21/c	150	2014
MUTKUH16	178,72	1,9910	2,0030	-0,0120	-0,5991	3,9800	P21/c	150	2014
MUTKUH16	176,19	2,0070	2,0030	4,0000e-3	0,1997	3,9800	P21/c	150	2014
MUTLAO	178,40	1,9120	2,0040	-0,0920	-4,5908	5,4500	P-1	298	2003
MUTLAO	178,10	1,9350	1,9810	-0,0460	-2,3221	5,4500	P-1	298	2003
MUTLAO	176,83	1,9510	1,9940	-0,0430	-2,1565	5,4500	P-1	298	2003
MUTLAO	179,48	1,9590	2,0010	-0,0420	-2,0990	5,4500	P-1	298	2003
MUTLAO	177,33	1,9610	1,9670	-6,0000e-3	-0,3050	5,4500	P-1	298	2003
MUTLAO	177,77	1,9710	2,0000	-0,0290	-1,4500	5,4500	P-1	298	2003
MUTLAO	178,13	1,9820	1,9820	0,0000	0,0000	5,4500	P-1	298	2003
MUTLAO	177,70	1,9830	1,9530	0,0300	1,5361	5,4500	P-1	298	2003
MUTLAO	178,52	1,9900	2,0510	-0,0610	-2,9742	5,4500	P-1	298	2003
MUTLAO	177,55	2,0010	1,9630	0,0380	1,9358	5,4500	P-1	298	2003
MUTLAO	175,86	2,0050	2,0330	-0,0280	-1,3773	5,4500	P-1	298	2003
MUTLAO	177,59	2,0100	2,0150	-5,0000e-3	-0,2481	5,4500	P-1	298	2003
MUTLAO	179,36	2,0140	2,0050	9,0000e-3	0,4489	5,4500	P-1	298	2003
MUTLAO	179,83	2,0180	2,0850	-0,0670	-3,2134	5,4500	P-1	298	2003
MUTLAO	175,02	2,0320	1,9480	0,0840	4,3121	5,4500	P-1	298	2003
MUTLAO	174,26	1,9620	2,0100	-0,0480	-2,3881	5,4500	P-1	298	2003
MUTLAO	178,58	1,9720	2,0510	-0,0790	-3,8518	5,4500	P-1	298	2003
MUTLAO	176,27	1,9790	1,9720	7,0000e-3	0,3550	5,4500	P-1	298	2003
MUTLAO	177,51	1,9830	2,0180	-0,0350	-1,7344	5,4500	P-1	298	2003
MUTLAO	177,91	1,9870	1,9770	0,0100	0,5058	5,4500	P-1	298	2003
MUTLAO	174,75	1,9870	2,0660	-0,0790	-3,8238	5,4500	P-1	298	2003
MUTLAO	176,69	1,9900	2,0240	-0,0340	-1,6798	5,4500	P-1	298	2003
MUTLAO	176,34	1,9940	1,9740	0,0200	1,0132	5,4500	P-1	298	2003
MUTLAO	177,19	1,9950	1,9920	3,0000e-3	0,1506	5,4500	P-1	298	2003
MUTLAO	172,69	1,9990	2,0440	-0,0450	-2,2016	5,4500	P-1	298	2003
MUTLAO	177,70	2,0020	1,9870	0,0150	0,7549	5,4500	P-1	298	2003
MUTLAO	176,92	2,0070	1,9910	0,0160	0,8036	5,4500	P-1	298	2003
MUTLAO	177,32	2,0120	2,0070	5,0000e-3	0,2491	5,4500	P-1	298	2003
MUTLAO	176,80	2,0160	1,9940	0,0220	1,1033	5,4500	P-1	298	2003
MUTLAO	176,39	2,0570	2,0170	0,0400	1,9831	5,4500	P-1	298	2003
MUTLAO	178,03	1,9260	2,0230	-0,0970	-4,7949	5,4500	P-1	298	2003
MUTLAO	178,11	1,9460	1,9940	-0,0480	-2,4072	5,4500	P-1	298	2003
MUTLAO	178,21	1,9540	1,9670	-0,0130	-0,6609	5,4500	P-1	298	2003
MUTLAO	179,23	1,9560	1,9780	-0,0220	-1,1122	5,4500	P-1	298	2003
MUTLAO	177,69	1,9710	2,0190	-0,0480	-2,3774	5,4500	P-1	298	2003
MUTLAO	176,77	1,9930	1,9510	0,0420	2,1527	5,4500	P-1	298	2003
MUTLAO	175,62	1,9940	1,9390	0,0550	2,8365	5,4500	P-1	298	2003
MUTLAO	178,39	1,9950	2,0130	-0,0180	-0,8942	5,4500	P-1	298	2003
MUTLAO	178,81	1,9970	1,9680	0,0290	1,4736	5,4500	P-1	298	2003
MUTLAO	176,95	2,0000	2,0680	-0,0680	-3,2882	5,4500	P-1	298	2003
MUTLAO	177,67	2,0040	2,0220	-0,0180	-0,8902	5,4500	P-1	298	2003
MUTLAO	175,67	2,0220	1,9980	0,0240	1,2012	5,4500	P-1	298	2003
MUTLAO	175,10	2,0320	1,9990	0,0330	1,6508	5,4500	P-1	298	2003
MUTLAO	176,63	2,0360	1,9790	0,0570	2,8802	5,4500	P-1	298	2003
MUTLAO	176,53	2,0580	2,0340	0,0240	1,1799	5,4500	P-1	298	2003
MUTLES	175,99	2,0120	2,0500	-0,0380	-1,8537	3,8400	Pna21	298	2003
MUTLES	178,27	2,0160	2,0060	0,0100	0,4985	3,8400	Pna21	298	2003
MUTLES	178,66	2,0020	2,0220	-0,0200	-0,9891	3,8400	Pna21	298	2003

NAPYUY	180,00	2,0410	2,0410	0,0000	0,0000	2,4900	C2/c	173	1997
NUGXES	174,16	2,0170	2,0270	-0,0100	-0,4933	5,7700	P21/c	295	1998
NUGXES	177,20	2,0330	2,0110	0,0220	1,0940	5,7700	P21/c	295	1998
NUGXES	176,40	2,0030	2,0140	-0,0110	-0,5462	5,7700	P21/c	295	1998
NUGXES	174,88	2,0720	2,0520	0,0200	0,9747	5,7700	P21/c	295	1998
NUGXES	176,39	1,9950	1,9990	-4,0000e-3	-0,2001	5,7700	P21/c	295	1998
NUGXES	175,40	2,0180	1,9860	0,0320	1,6113	5,7700	P21/c	295	1998
OBIWAY	176,24	2,0190	2,0220	-3,0000e-3	-0,1484	4,8300	P21/n	296	2004
OFABEF	173,93	2,0420	2,0450	-3,0000e-3	-0,1467	2,6800	P21/c	296	2018
OFABEF	177,34	2,0450	2,0440	1,0000e-3	0,0489	2,6800	P21/c	296	2018
OFABEF	174,16	2,0390	2,0460	-7,0000e-3	-0,3421	2,6800	P21/c	296	2018
OFABEF	178,25	2,0290	2,0300	-1,0000e-3	-0,0493	2,6800	P21/c	296	2018
OFABIJ	179,82	2,0370	2,0370	0,0000	0,0000	4,5500	C2/c	296	2018
OFABIJ	178,79	2,0730	2,0680	5,0000e-3	0,2418	4,5500	C2/c	296	2018
OFABIJ	179,25	2,0350	2,0350	0,0000	0,0000	4,5500	C2/c	296	2018
OKALER	173,57	1,9940	2,0070	-0,0130	-0,6477	2,8200	C2/c	298	2003
OKALER	178,76	2,0280	2,0280	0,0000	0,0000	2,8200	C2/c	298	2003
OKALER	179,33	2,0060	2,0170	-0,0110	-0,5454	2,8200	C2/c	298	2003
OKALER	176,66	2,0130	1,9980	0,0150	0,7508	2,8200	C2/c	298	2003
OKALIV	173,80	2,0000	2,0110	-0,0110	-0,5470	3,7200	P2/n	298	2003
OKALIV	172,82	2,0180	2,0030	0,0150	0,7489	3,7200	P2/n	298	2003
OKALIV	177,62	2,0060	2,0220	-0,0160	-0,7913	3,7200	P2/n	298	2003
OKALIV	178,27	2,0170	2,0160	1,0000e-3	0,0496	3,7200	P2/n	298	2003
OKALIV	174,07	2,0100	2,0030	7,0000e-3	0,3495	3,7200	P2/n	298	2003
OKALIV	174,07	2,0190	2,0090	0,0100	0,4978	3,7200	P2/n	298	2003
OKALIV	177,99	2,0190	2,0110	8,0000e-3	0,3978	3,7200	P2/n	298	2003
OKALIV	178,03	2,0210	2,0240	-3,0000e-3	-0,1482	3,7200	P2/n	298	2003
OZIKIT	172,93	1,9960	1,9960	0,0000	0,0000	5,6100	P21	100	2011
OZIKIT	171,14	2,0010	1,9890	0,0120	0,6033	5,6100	P21	100	2011
OZIKIT	173,01	2,0080	2,0020	6,0000e-3	0,2997	5,6100	P21	100	2011
PAQTAD	178,20	2,0330	2,0340	-1,0000e-3	-0,0492	4,9200	P-1	173	2005
PEJSAZ	177,31	1,9840	1,9900	-6,0000e-3	-0,3015	6,8300	P-1	296	2006
PEJSAZ	176,26	2,0070	2,0070	0,0000	0,0000	6,8300	P-1	296	2006
PEJSAZ	172,49	1,9860	1,9920	-6,0000e-3	-0,3012	6,8300	P-1	296	2006
PEJSAZ	172,64	2,0090	1,9990	0,0100	0,5003	6,8300	P-1	296	2006
PEJSED	176,88	2,0010	2,0450	-0,0440	-2,1516	8,8400	P21/a	297	2006
PEJSED	174,67	2,0240	2,0100	0,0140	0,6965	8,8400	P21/a	297	2006
PEMSEG	175,86	2,0130	2,0290	-0,0160	-0,7886	3,7300	P-1	296	2006
PEMSEG	175,61	2,0130	2,0360	-0,0230	-1,1297	3,7300	P-1	296	2006
PIZJOY	177,75	2,0220	2,0220	0,0000	0,0000	2,7200	C2/c	273	2008
PIZJUE	180,00	2,0070	2,0070	0,0000	0,0000	3,4800	P-1	273	2008
PIZJUE	177,06	2,0070	2,0120	-5,0000e-3	-0,2485	3,4800	P-1	273	2008
PIZJUE	180,00	2,0110	2,0110	0,0000	0,0000	3,4800	P-1	273	2008
PIZKEP	180,00	2,0060	2,0060	0,0000	0,0000	7,5300	P-1	296	2008
PIZKEP	180,00	2,0210	2,0210	0,0000	0,0000	7,5300	P-1	296	2008
PIZKEP	180,00	2,0540	2,0540	0,0000	0,0000	7,5300	P-1	296	2008
PIZKEP	180,00	2,0680	2,0680	0,0000	0,0000	7,5300	P-1	296	2008
PIZKEP01	180,00	2,0160	2,0160	0,0000	0,0000	2,2600	P21/n	100	2013
PIZKEP01	180,00	2,0220	2,0220	0,0000	0,0000	2,2600	P21/n	100	2013
PIZKEP02	180,00	2,0140	2,0140	0,0000	0,0000	3,5000	P21/n	300	2013
PIZKEP02	180,00	2,0190	2,0190	0,0000	0,0000	3,5000	P21/n	300	2013
PIZKEP03	180,00	2,0130	2,0130	0,0000	0,0000	3,1200	P21/n	200	2013
PIZKEP03	180,00	2,0330	2,0330	0,0000	0,0000	3,1200	P21/n	200	2013
PIZKIT	177,99	2,0120	2,0170	-5,0000e-3	-0,2479	5,0800	P-1	298	2008
PIZKIT	177,75	2,0150	2,0100	5,0000e-3	0,2488	5,0800	P-1	298	2008
PIZKIT	176,23	2,0170	2,0170	0,0000	0,0000	5,0800	P-1	298	2008
PIZKOZ	180,00	2,2090	2,2090	0,0000	0,0000	2,2600	P21/n	273	2008
POVDAG	170,93	2,0620	2,0110	0,0510	2,5361	3,9400	P-1	100	2009
POVDAG	173,69	2,0730	2,0060	0,0670	3,3400	3,9400	P-1	100	2009
QOXSEC	176,60	2,0030	2,0020	1,0000e-3	0,0500	3,9600	C2/c	296	2009
QOXSEC	176,78	1,9960	1,9810	0,0150	0,7572	3,9600	C2/c	296	2009
QOXSIG	177,95	1,8920	1,9110	-0,0190	-0,9942	5,1900	C2/c	296	2009
QOXSOM	174,87	2,0380	2,0400	-2,0000e-3	-0,0980	3,0400	C2/c	296	2009
QURRIF	176,85	1,9960	2,0020	-6,0000e-3	-0,2997	2,6300	Pnma	100	2010
QURROL	176,05	1,9960	2,0100	-0,0140	-0,6965	4,0800	Pnma	100	2010
QURRUR	175,35	2,0060	2,0000	6,0000e-3	0,3000	4,0000	Pnma	100	2010
QURSAY	177,44	1,9930	2,0040	-0,0110	-0,5489	3,8800	Pnma	100	2010
REXXEX	175,54	1,9420	1,9680	-0,0260	-1,3211	5,3300	P212121	295	1996
REXXEX	177,59	1,9440	1,9390	5,0000e-3	0,2579	5,3300	P212121	295	1996
RILZEU	177,30	2,0140	2,0260	-0,0120	-0,5923	2,8000	C2/c	100	2018
RILZEU	175,26	2,0310	2,0310	0,0000	0,0000	2,8000	C2/c	100	2018
RILZEU01	175,35	2,0220	2,0360	-0,0140	-0,6876	2,5600	Cc	100	2018
RILZEU01	176,08	2,0230	2,0210	2,0000e-3	0,0990	2,5600	Cc	100	2018
SMHYAN	180,00	1,7950	1,7950	0,0000	0,0000	4,3000	P-1	295	1978
SMHYAN	180,00	2,0700	2,0700	0,0000	0,0000	4,3000	P-1	295	1978
UDACAG	178,56	2,0140	2,0090	5,0000e-3	0,2489	3,4500	P21/n	133	2016
UFEBIR	180,00	2,0070	2,0070	0,0000	0,0000	3,1700	P-1	295	2008
UFEBOX	179,16	2,0020	2,0030	-1,0000e-3	-0,0499	4,7000	P-1	100	2008

UHEGIZ	176,40	2,0020	2,0050	-3,0000e-3	-0,1496	7,3300	P-1	173	2015
UJOQAN	176,20	2,0220	2,0190	3,0000e-3	0,1486	3,5500	Ccca	173	2016
XACQOK	180,00	2,0100	2,0100	0,0000	0,0000	5,3100	P-1	200	2016
XACRAX	177,00	1,9930	1,9900	3,0000e-3	0,1508	3,2300	P21/m	200	2016
XACRAX	175,56	2,0060	1,9920	0,0140	0,7028	3,2300	P21/m	200	2016
XACREB	178,98	1,9920	1,9980	-6,0000e-3	-0,3003	3,1400	P-1	200	2016
XACREB	177,19	2,0070	2,0040	3,0000e-3	0,1497	3,1400	P-1	200	2016
XACRIF	180,00	1,9950	1,9950	0,0000	0,0000	2,8800	P21/n	200	2016
XOMVUT	178,09	2,0000	1,9960	4,0000e-3	0,2004	1,2800	P-1	178	2019
XOMVUT	178,62	2,0050	2,0090	-4,0000e-3	-0,1991	1,2800	P-1	178	2019
XOMVUT	178,85	2,0000	2,0010	-1,0000e-3	-0,0500	1,2800	P-1	178	2019
XOMWEE	179,32	2,0140	1,9840	0,0300	1,5121	6,0700	P21/c	140	2019
XOMWEE	178,72	1,9760	1,9690	7,0000e-3	0,3555	6,0700	P21/c	140	2019
XOMWEE	177,66	1,9860	1,9910	-5,0000e-3	-0,2511	6,0700	P21/c	140	2019
YOSRII	179,73	2,0220	2,0390	-0,0170	-0,8337	4,3700	Pnma	299	2009
YOSROO	177,75	2,0480	1,9710	0,0770	3,9066	3,6200	P21	293	2009
YOSROO	176,87	2,0150	2,0090	6,0000e-3	0,2987	3,6200	P21	293	2009
YOSROO	178,85	1,9890	1,9990	-0,0100	-0,5003	3,6200	P21	293	2009
YOSROO	178,09	2,0090	1,9590	0,0500	2,5523	3,6200	P21	293	2009
YOSRUU	178,74	1,9930	2,0010	-8,0000e-3	-0,3998	4,2900	P21/c	298	2009
YOSRUU	179,72	1,9970	1,9920	5,0000e-3	0,2510	4,2900	P21/c	298	2009
YOSRUU	177,09	1,9940	1,9810	0,0130	0,6562	4,2900	P21/c	298	2009
YOSRUU01	179,68	2,0030	2,0050	-2,0000e-3	-0,0998	4,3200	P21/c	100	2014
YOSRUU01	179,03	1,9780	1,9780	0,0000	0,0000	4,3200	P21/c	100	2014
YOSRUU01	176,82	2,0010	2,0010	0,0000	0,0000	4,3200	P21/c	100	2014
YOSRUU02	179,70	1,9900	2,0010	-0,0110	-0,5497	4,4200	P21/c	120	2014
YOSRUU02	179,22	1,9740	1,9740	0,0000	0,0000	4,4200	P21/c	120	2014
YOSRUU02	176,61	2,0040	2,0050	-1,0000e-3	-0,0499	4,4200	P21/c	120	2014
YOSRUU03	179,87	2,0040	1,9970	7,0000e-3	0,3505	4,5400	P21/c	150	2014
YOSRUU03	179,09	1,9770	1,9770	0,0000	0,0000	4,5400	P21/c	150	2014
YOSRUU03	176,75	2,0020	2,0080	-6,0000e-3	-0,2988	4,5400	P21/c	150	2014
YOSRUU04	179,80	2,0080	2,0050	3,0000e-3	0,1496	4,7300	P21/c	180	2014
YOSRUU04	179,21	1,9830	1,9870	-4,0000e-3	-0,2013	4,7300	P21/c	180	2014
YOSRUU04	176,67	2,0130	1,9940	0,0190	0,9529	4,7300	P21/c	180	2014
YOSRUU05	179,86	2,0000	1,9930	7,0000e-3	0,3512	4,9100	P21/c	210	2014
YOSRUU05	178,82	1,9780	1,9770	1,0000e-3	0,0506	4,9100	P21/c	210	2014
YOSRUU05	176,63	2,0110	2,0030	8,0000e-3	0,3994	4,9100	P21/c	210	2014
YOSRUU06	179,41	2,0020	1,9940	8,0000e-3	0,4012	5,0100	P21/c	240	2014
YOSRUU06	178,97	1,9790	1,9710	8,0000e-3	0,4059	5,0100	P21/c	240	2014
YOSRUU06	176,81	2,0050	2,0050	0,0000	0,0000	5,0100	P21/c	240	2014
YOSRUU07	179,49	1,9980	1,9900	8,0000e-3	0,4020	4,5300	P21/c	270	2014
YOSRUU07	179,00	1,9830	1,9660	0,0170	0,8647	4,5300	P21/c	270	2014
YOSRUU07	176,39	2,0180	2,0020	0,0160	0,7992	4,5300	P21/c	270	2014
YOSRUU08	179,69	1,9940	1,9940	0,0000	0,0000	3,2000	P21/c	293	2014
YOSRUU08	178,54	1,9750	1,9840	-9,0000e-3	-0,4536	3,2000	P21/c	293	2014
YOSRUU08	176,81	1,9970	1,9900	7,0000e-3	0,3518	3,2000	P21/c	293	2014
YOSRUU09	178,72	1,9900	2,0090	-0,0190	-0,9457	3,2700	P21/c	293	2014
YOSRUU09	178,51	1,9670	1,9690	-2,0000e-3	-0,1016	3,2700	P21/c	293	2014
YOSRUU09	176,84	2,0070	1,9870	0,0200	1,0065	3,2700	P21/c	293	2014
YOSRUU10	179,57	1,9830	1,9800	3,0000e-3	0,1515	3,4000	P21/c	293	2014
YOSRUU10	177,71	1,9430	1,9780	-0,0350	-1,7695	3,4000	P21/c	293	2014
YOSRUU10	177,31	2,0120	1,9640	0,0480	2,4440	3,4000	P21/c	293	2014
YOSRUU11	177,75	1,9550	1,9750	-0,0200	-1,0127	3,6500	P21/c	293	2014
YOSRUU11	177,91	1,9720	2,0160	-0,0440	-2,1825	3,6500	P21/c	293	2014
YOSRUU11	176,31	2,0190	1,9810	0,0380	1,9182	3,6500	P21/c	293	2014
YOSRUU12	179,58	1,9710	1,9690	2,0000e-3	0,1016	4,7200	P21/c	293	2014
YOSRUU12	177,70	1,9940	1,9960	-2,0000e-3	-0,1002	4,7200	P21/c	293	2014
YOSRUU12	175,78	1,9810	1,9860	-5,0000e-3	-0,2518	4,7200	P21/c	293	2014
YOSRUU13	177,83	1,9790	1,9790	0,0000	0,0000	4,4000	P21/c	293	2014
YOSRUU13	177,97	1,9220	1,9580	-0,0360	-1,8386	4,4000	P21/c	293	2014
YOSRUU13	175,10	1,9870	1,9680	0,0190	0,9654	4,4000	P21/c	293	2014
YOSRUU14	177,84	1,9740	1,9510	0,0230	1,1789	2,9800	P21/c	293	2014
YOSRUU14	175,64	2,0000	1,9890	0,0110	0,5530	2,9800	P21/c	293	2014
YOSRUU14	174,48	1,9590	1,9600	-1,0000e-3	-0,0510	2,9800	P21/c	293	2014
YOSRUU15	178,65	1,9520	1,9670	-0,0150	-0,7626	4,2600	P21/c	293	2014
YOSRUU15	176,63	1,9970	2,0030	-6,0000e-3	-0,2996	4,2600	P21/c	293	2014
YOSRUU15	174,66	1,9400	1,9520	-0,0120	-0,6148	4,2600	P21/c	293	2014
YOSSEF	179,14	2,0200	1,9950	0,0250	1,2531	3,9600	P21/n	299	2009
YOSSEF01	178,95	2,0270	2,0270	0,0000	0,0000	2,9300	Pbcn	299	2009
YOSSIJ	177,02	1,9830	1,9690	0,0140	0,7110	3,3800	P21/c	293	2009
YOSSIJ	177,81	2,0020	1,9720	0,0300	1,5213	3,3800	P21/c	293	2009
ZINTIB	180,00	2,0190	2,0190	0,0000	0,0000	2,4600	P21/n	100	2013
ZINTIB	180,00	2,0260	2,0260	0,0000	0,0000	2,4600	P21/n	100	2013

Table S22. N-X-N Bond angles and N-X bond distances in the related structures X-ray structures of Hg(II), Cd(II), Te(III), Er(III), Zn(II), Gd(III), Mn(II), Fe(II), Ni(II), Cr(II), and Rh(I) complexes available in the CSD. Only close to linear (180° ± 10°), nonpolymeric structures not having additional metal coordination are considered.

Ref. code	Angle	N(1)-X (Å)	N(2)-X (Å)	Δ(N-Au)	%	R	SpGr	Temp	Pub. Year
ACABAH	180,0	2,0870	2,0920	-5,0000e-3	-0,2390	1,7800	C2/c	293	2001
ACABAH	180,0	2,1130	2,1130	0,0000	0,0000	1,7800	C2/c	293	2001
CEWLAS	180,0	2,1470	2,1470	0,0000	0,0000	3,7100	P21/c	293	2006
FMTPHG	180,0	2,0160	2,0160	0,0000	0,0000	2,9000	C2/c	295	1979
GEGWOE	176,3	2,0580	2,0380	0,0200	0,9814	4,4000	P21/c	295	1988
GEGWUK	180,0	2,0210	2,0210	0,0000	0,0000	3,5000	P21/c	295	1988
GEGWUK10	178,7	2,0300	2,0180	0,0120	0,5946	3,5000	P21/c	295	1989
JEJGUA	180,0	2,0270	2,0270	0,0000	0,0000	3,2000	P21/n	295	1990
JEZLOP	174,6	1,9750	2,0500	-0,0750	-3,6585	7,2000	P-1	295	1989
JIBXIB	177,0	2,0330	2,0100	0,0230	1,1443	4,7000	P21/c	295	1988
JINLEY	175,1	1,8190	1,8260	-7,0000e-3	-0,3834	6,0400	P-1	200	2007
JINLEY	173,8	1,8230	1,8280	-5,0000e-3	-0,2735	6,0400	P-1	200	2007
JINLEY	172,2	1,8140	1,8120	2,0000e-3	0,1104	6,0400	P-1	200	2007
JINLEY	175,8	1,8220	1,8170	5,0000e-3	0,2752	6,0400	P-1	200	2007
JUBSUU	179,4	2,0410	2,0310	0,0100	0,4924	3,7000	P21/n	295	1992
MAPDEM	172,9	2,1420	2,1560	-0,0140	-0,6494	8,0000	Pcmn	295	1999
MGSACC10	175,4	2,0290	2,0580	-0,0290	-1,4091	6,2000	P-1	295	1982
MOMSUD	173,0	2,0370	2,0410	-4,0000e-3	-0,1960	4,5100	P21/n	200	2008
MTYMHG10	180,0	2,0350	2,0350	0,0000	0,0000	7,7000	P21/a	295	1974
OMODUQ	180,0	2,1070	2,1070	0,0000	0,0000	5,8100	P-1	150	2011
OMODUQ	180,0	2,1170	2,1170	0,0000	0,0000	5,8100	P-1	150	2011
PUTPUP	179,4	1,8220	1,8180	4,0000e-3	0,2200	3,5500	P21/n	185	1998
QETWIW	172,8	2,3120	2,3100	2,0000e-3	0,0866	4,2400	P-1	293	2007
RAJSIE	180,0	2,0000	2,0000	0,0000	0,0000	2,1000	P21/n	173	2001
SISJOT	177,7	2,0390	2,0310	8,0000e-3	0,3939	4,7000	P21/n	295	1991
VEJGAU	175,7	2,0250	2,0240	1,0000e-3	0,0494	2,8400	P21/n	123	2012
VUSCER	172,2	1,9820	2,0200	-0,0380	-1,8812	5,3000	P21/n	295	1992
WAJDAM	171,4	1,8400	1,8470	-7,0000e-3	-0,3790	4,5000	C2/c	295	1992
WEZXUU	180,0	1,9790	1,9790	0,0000	0,0000	5,8000	P-1	295	1993
WUQLIE	180,0	2,0380	2,0380	0,0000	0,0000	3,2000	P-1	85	2010
YAXFOS	180,0	2,0680	2,0680	0,0000	0,0000	5,1000	P21/c	295	1993
YITVIH	180,0	2,1490	2,1490	0,0000	0,0000	3,5500	C2/c	170	2008
ZIRNIZ	173,5	2,0600	2,0600	0,0000	0,0000	3,0200	C2/c	200	2014
AHOHEL	178,6	2,0440	2,0400	4,0000e-3	0,1961	4,4200	P21/c	150	2009
AHOHIP	177,9	1,9720	1,9760	-4,0000e-3	-0,2024	4,6000	P21/c	150	2009
IFIZUU	179,6	1,8750	1,8750	0,0000	0,0000	6,5700	C2/c	173	2013
IFIZUU	178,9	1,8640	1,8640	0,0000	0,0000	6,5700	C2/c	173	2013
IFIZUU	178,3	1,8600	1,8610	-1,0000e-3	-0,0537	6,5700	C2/c	173	2013
IFOBEM	179,3	1,8630	1,8630	0,0000	0,0000	5,4000	C2/c	173	2013
IFOBEM	176,0	1,8560	1,8560	0,0000	0,0000	5,4000	C2/c	173	2013
IFOBEM	177,9	1,8400	1,8670	-0,0270	-1,4462	5,4000	C2/c	173	2013
SAGJES	180,0	1,8560	1,8560	0,0000	0,0000	7,4000	P2/c	293	2016
SAGJIW	173,6	1,7920	1,7910	1,0000e-3	0,0558	4,4500	P212121	293	2016
TEYDIN	175,4	2,2090	2,2090	0,0000	0,0000	5,8700	C2/c	293	2018
TIFPEF	177,8	2,0450	2,0450	0,0000	0,0000	7,3400	P-421c	200	2012
TIFPIJ	178,0	2,0200	2,0310	-0,0110	-0,5416	7,2000	P21/c	200	2012
VAJFES	171,6	2,9450	2,9370	8,0000e-3	0,2724	5,0500	C2/c	123	2003
YUPFIA	179,0	1,8780	1,8600	0,0180	0,9677	7,1400	C2/c	173	2015
YUPFIA	179,0	1,8610	1,8850	-0,0240	-1,2732	7,1400	C2/c	173	2015
YUPMAZ	180,0	1,8640	1,8640	0,0000	0,0000	9,2400	C2/c	173	2015
YUPMAZ	180,0	1,9040	1,9040	0,0000	0,0000	9,2400	C2/c	173	2015
YUVDUQ	180,0	1,9860	1,9860	0,0000	0,0000	1,9300	I41/a	100	2015
ZEKYET	178,2	2,0960	2,0880	8,0000e-3	0,3831	6,5100	P21/c	130	1995
ZOLFOX	178,2	2,0960	2,0850	0,0110	0,5276	6,5900	P21/c	130	2014

7. References

1. Bruker AXS Inc: Madison, Wisconsin, USA, 2007.
2. Bruker AXS Inc: Madison, Wisconsin, USA, 2019.
3. Sheldrick, G. M., Shelxt-Integrated Space-Group and Crystal-Structure Determination. *Acta Cryst. Sect. A* **2015**, *71*, 3-8.
4. Sheldrick, G. M., Crystal Structure Refinement with Shelxl. *Acta Cryst. Sect. C* **2015**, *71*, 3-8.
5. Dolomanov, O. V.; Bourhis, L. J.; Gildea, R. J.; Howard, J. A. K.; Puschmann, H., Olex2: A Complete Structure Solution, Refinement and Analysis Program. *J. Appl. Chem.* **2009**, *42*, 339-341.
6. Parsons, S.; Flack, H., Precise Absolute-Structure Determination in Light-Atom Crystals. *Acta Cryst. Sect. A* **2004**, *60*, S61-S61.
7. Flack, H. D., On Enantiomorph-Polarity Estimation. *Acta Crystallogr. A* **1983**, *39*, 876-881.
8. Carlsson, A.-C. C.; Gräfenstein, J.; Budnjo, A.; Laurila, J. L.; Bergquist, J.; Karim, A.; Kleinmaier, R.; Brath, U.; Erdelyi, M., Symmetric Halogen Bonding Is Preferred in Solution. *J. Am. Chem. Soc.* **2012**, *134*, 5706-15.
9. Bedin, M.; Karim, A.; Reitti, M.; Carlsson, A. C.; Topic, F.; Cetina, M.; Pan, F.; Havel, V.; Al-Ameri, F.; Sindelar, V.; Rissanen, K.; Grafenstein, J.; Erdelyi, M., Counterion Influence on the N-I-N Halogen Bond. *Chem. Sci.* **2015**, *6*, 3746-3756.
10. Carlsson, A.-C. C.; Mehmeti, K.; Uhrbom, M.; Karim, A.; Bedin, M.; Puttreddy, R.; Kleinmaier, R.; Neverov, A. A.; Nekoueishahraki, B.; Gräfenstein, J.; Rissanen, K.; Erdélyi, M., Substituent Effects on the $[N-I-N]^+$ Halogen Bond. *J. Am. Chem. Soc.* **2016**, *138*, 9853-9863.
11. Karim, A.; Reitti, M.; Carlsson, A.-C. C.; Gräfenstein, J.; Erdélyi, M., The Nature of $[N-Cl-N]^+$ and $[N-F-N]^+$ Halogen Bonds in Solution. *Chem. Sci.* **2014**, *5*, 3226-3233.
12. Carlsson, A.-C. C.; Gräfenstein, J.; Laurila, J. L.; Bergquist, J.; Erdélyi, M., Symmetry of $[N-X-N]^+$ Halogen Bonds in Solution. *Chem. Commun.* **2012**, *48*, 1458-1460.
